# Development of structurally extended benzosiloxaboroles – synthesis and *in vitro* biological evaluation†

**DOI:** 10.1039/d1ra04127d

**Published:** 2021-07-20

**Authors:** P. Pacholak, J. Krajewska, P. Wińska, J. Dunikowska, U. Gogowska, J. Mierzejewska, K. Durka, K. Woźniak, A. E. Laudy, S. Luliński

**Affiliations:** Faculty of Chemistry, Warsaw University of Technology Noakowskiego 3 00-664 Warsaw Poland sergiusz.lulinski@pw.edu.pl; University of Warsaw, Faculty of Chemistry Pasteura 1 02-093 Warsaw Poland; Department of Pharmaceutical Microbiology, Medical University of Warsaw Oczki 3 02-007 Warsaw Poland alaudy@wp.pl

## Abstract

The synthesis of potassium 6-hydroxy-7-chloro-1,1-dimethyl-3,3-difluorobenzo-1,2,3-siloxaborolate 5b from readily available 4-bromo-2-chlorophenol was developed. This compound proved useful in various derivatizations resulting in a wide range of *O*-functionalized benzosiloxaboroles. Reactions of 5b with selected substituted benzoyl chlorides gave rise to a series of respective derivatives with 6-benzoate side groups attached to the benzosiloxaborole core. Furthermore, treatment of 5b with substituted benzenesufonyl chlorides afforded several benzosiloxaboroles bearing functionalized benzenesulfonate moieties at the 6 position. The synthesis of related chloropyridine-2-yloxy substituted benzosiloxaboroles was accomplished by a standard approach involving silylation/boronation of appropriate heterodiaryl ethers. Investigation of biological activity of obtained compounds revealed that some benzoate and most benzenesulfonate derivatives exhibit high activity against Gram-positive cocci such as methicillin-sensitive *Staphylococcus aureus* ATCC 6538P as well as methicillin-resistant *S. aureus* ATCC 43300 with the MIC values in the range of 0.39–3.12 mg L^−1^. Some benzenesulfonate derivatives showed also potent activity against *Enterococcus faecalis* ATCC 29212 and *E. faecium* ATCC 6057 with MIC = 6.25 mg L^−1^. Importantly, for the most promising cocci-active benzenesulfonate derivatives the obtained MIC values were far below the cytotoxicity limit determined with respect to human normal lung fibroblasts (MRC-5). For those derivatives, the obtained IC_50_ values were higher than 12.3 mg L^−1^. The results of antimicrobial activity and cytotoxicity indicate that the tested compounds can be considered as potential antibacterial agents.

## Abstract 2

The synthesis of potassium 6-hydroxy-7-chloro-1,1-dimethyl-3,3-difluorobenzo-1,2,3-siloxaborolate 5b from readily available 4-bromo-2-chlorophenol was developed.

## Graphical abstract

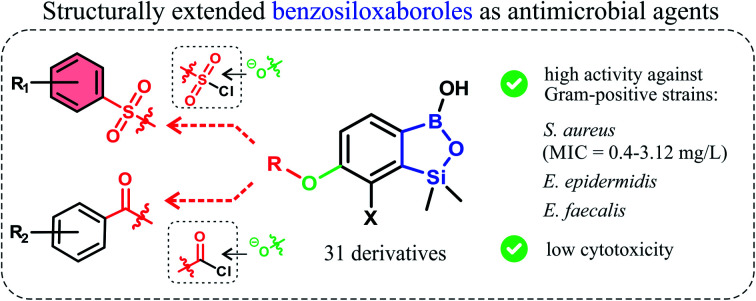

## 1. Introduction

**Fig. 1:**
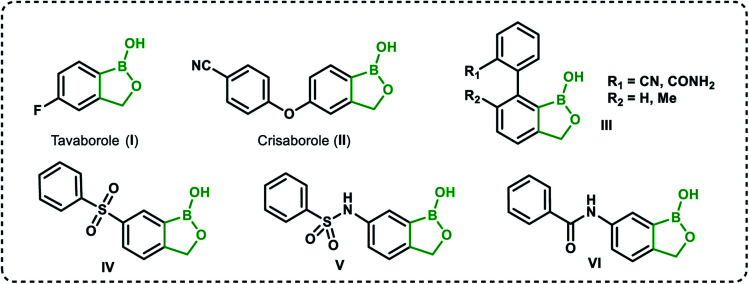
Examples of biologically active benzoxaboroles.

Recently, organoboron compounds have attracted increased attention as a subject of studies in the area of medicinal chemistry.^1–3^ Numerous compounds were found to exhibit biological activity, mostly as anti-cancer, anti-inflammatory and anti-microbial agents. From the structural point of view, selected classes of organoboranes seem to be especially suitable for such applications. These include boron-rich cluster compounds, *i.e.*, carborane derivatives considered for the use in Boron Neutron Capture Therapy.^4^ There are also numerous examples of biologically active boronated peptide derivatives.^5–7^ Currently, arylboronic acids are highly popular synthetic reagents which found also applications in other fields, *e.g.*, as potent receptors of saccharides and other diols with a special emphasis on nucleosides and catechol derivatives.^8^ Their antimicrobial activity was recognized in the 1930's.^9^ Benzoxaboroles are a specific class of cyclic arylboronic hemiesters which were obtained the 1950's.^10,11^ However, they became highly popular only 50 years later when their potent antimicrobial activity was discovered.^12^ Extensive studies resulted in the preparation of numerous derivatives which were further evaluated from the point of view of medicinal chemistry.^13–15^ Those efforts have already met with success as two benzoxaboroles including antifungal agent Tavaborole I (trade name Kerydin) (Fig. 1), and anti-inflammatory Crisaborole II (trade name Eucrisa) (Fig. 1), were approved by FDA for the clinical use.^16^ The mechanism of action of benzoxaboroles relies on their physicochemical specifity based on enhanced electron-deficient character of the boron atom. In fact, benzoxaboroles are stronger Lewis acids than the corresponding arylboronic acids.^17^ In general, the boron centre plays the key role in the binding to the biological targets through formation of strong covalent bonds. However, the binding can be substantially enhanced by additional interactions occurring with participation of various functional groups or larger structural fragments. Thus, a specific activity can be achieved by proper structural design, therefore intensive efforts resulted in elaboration of synthetic protocols for the preparation of thousands of functionalized benzoxaboroles.^15^ For example, 7-(cyanophenyl) benzoxaboroles III (Fig. 1) showed antituberculosis potency^18^ whereas 3-aminomethyl derivatives were active against Gram-negative bacteria.^19^ In another work, the series of compounds bearing substituted phenyl groups connected to benzoxaborole core at the 6 position through various linkages (Fig. 1) were prepared and tested as potential antitrypanosomal agents.^20^ It was demonstrated that compounds with sulfone IV, sulfonamide V and amide VI linkages were most promising, and therefore it was concluded that their improved activity is connected to enhanced hydrogen-bond-acceptor character of these linkage groups.

**Fig. 2:**
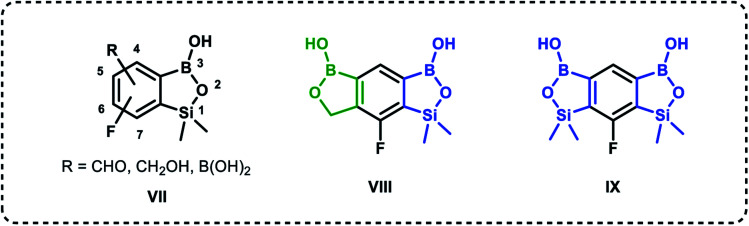
Examples of functionalized benzosiloxaboroles.^26^ Position numbering scheme is additionally provided for the general structure VII (note that it is different between benzoxa- and benzosiloxaboroles).

Based on the concept of bioisosterism,^21^ we decided to prepare some benzoxaborole congeners. Thus, we developed synthetic routes to pyridoxaboroles^22^ where the benzene rings replaced with the pyridine one. However, we have put our major efforts to benzosiloxaboroles^23,24^ where the silicon atom serves as the bioisostere of the carbon atom in the oxaborole ring. Despite the close analogy resulting from the location of carbon and silicon in the same group of periodic table, the chemical properties of those elements are quite different. From the point of view of biological activity it is important to note that Lewis acidity of the boron atom is increased when comparing benzosiloxaboroles to benzoxaboroles which may be attributed to increased π-acceptor ability of silicon *vs.* saturated carbon atom.^25^ In addition, one can expect that lipophilicity will be increased when the methylene group is replaced with the larger SiMe_2_ fragment. As a consequence, antimicrobial activity of respective benzoxa- and benzosiloxaboroles is different. We have already succeeded in preparation and comprehensive characterization of various functionalized benzosiloxaboroles VII (Fig. 2). It was found that simple fluorinated benzosiloxaboroles are potent antifungal agents whereas other diboron derivatives VIII–IX were identified as inhibitors of KPC-2 β-lactamase.^26^ We have also observed that replacement of fluorines with chlorines at 6 and 7 positions was beneficial for antibacterial activity. Therefore, we decided to check whether introduction of larger substituents adjacent to chlorine will further enhance antibacterial potency. To some extent, this concept was inspired by the fact that diverse biological activity of benzoxaboroles is observed or improved due to attachment of various pendant aryl substituents as demonstrated by examples shown in Fig. 1. Thus, in this work we report new family of structurally expanded benzosiloxaboroles with a special focus on derivatives with arylsulfonate side groups which showed the most promising antibacterial activity, especially towards various strains of *Staphylococcus aureus*. Clinical strains of methicillin-resistant *S. aureus* have been a serious problem in both hospital and open treatment for many years. *S. aureus* MRSA strains are resistant to almost all β-lactams and often resistant to antibiotics of other classes. Recently, an increase in the number of isolates resistant to one of the newer group of antibiotics, *i.e.*, glycopeptides, has been observed.^27^ Therefore, it is necessary to search for new groups of compounds active against these bacteria, preferably with a new mechanism of action.

## 2. Results and discussion

### 2.1. Synthesis

**Scheme 1:**
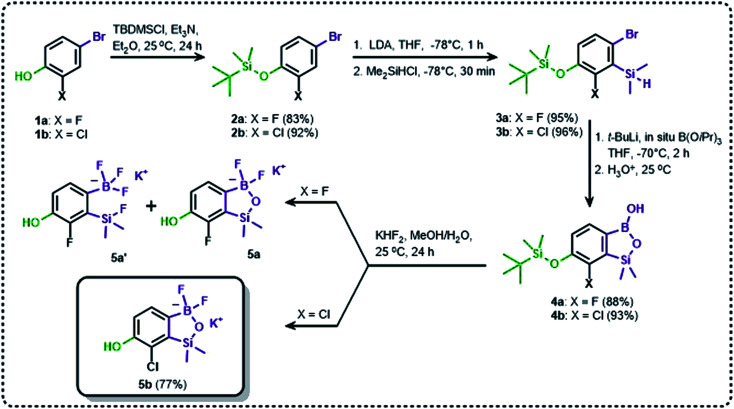
Synthesis of hydroxy-substituted benzosiloxa(difluoro)borolates 5a–5b.

The general synthetic approach to final targeted benzosiloxaboroles started with inexpensive 4-bromo-2-halophenols 1a–1b (Scheme 1). The hydroxyl groups were protected with chloro(*tert*-butyl)dimethylsilane (TBDMSCl) and the resulting silyl ethers 2a–2b were subjected to deprotonation with LDA in THF at −78 °C followed by trapping of corresponding aryllithium intermediates with Me_2_Si(H)Cl in accordance with a general protocol reported by us previously.^23^ The reactions occurred regioselectively at the position between two halogens in accordance with a strong cumulated *ortho*-acidifying effect of those two substituents.^28,29^ The functionalized arylsilanes 3a–3b were converted to respective benzosiloxaboroles 4a–4b after some optimization of reaction conditions. Thus, the most effective approach involved Br/Li exchange with *t*-BuLi in THF at −78 °C followed by immediate trapping with B(O*i*Pr)_3_ present in a reaction mixture (“*in situ* quench” technique^30^). The hydrolysis effected with water resulted in cleavage of Si–H bond which occurs rapidly under alkaline conditions due to *ortho*-assistance of the anionic boronate group.^31^ The benzosiloxaboroles 4a–4b bearing silyloxy groups at the 6-position have been obtained in good yields as white solids soluble in common organic solvents (Scheme 1). In a subsequent step, 4a–4b were subjected to deprotection of OTBDMS groups with KHF_2_ in MeOH/H_2_O. Unfortunately, in the case of 4a, the reaction resulted in a mixture of potassium salts of benzosiloxaborolate and aryltrifluoroborate anions 5a, 5a′, respectively; the latter product formed due to subsequent cleavage of siloxaborole ring in 5a. The attempts to isolate the desired product 5a in a pure form were unsuccessful. In contrast, the salt 5b was formed selectively as it was not prone to subsequent ring opening. It was isolated as a non-stoichiometric DMF solvate as this solvent was used for the final extraction of 5b from a crude product containing substantial amounts of inorganic fluoride salts. The solvent could not be quantitatively removed even by prolonged heating under reduced pressure (10^−3^ mbar). However, the presence of DMF does not disturb subsequent derivatization of 5b as it was also carried out using this solvent.

**Scheme 2:**
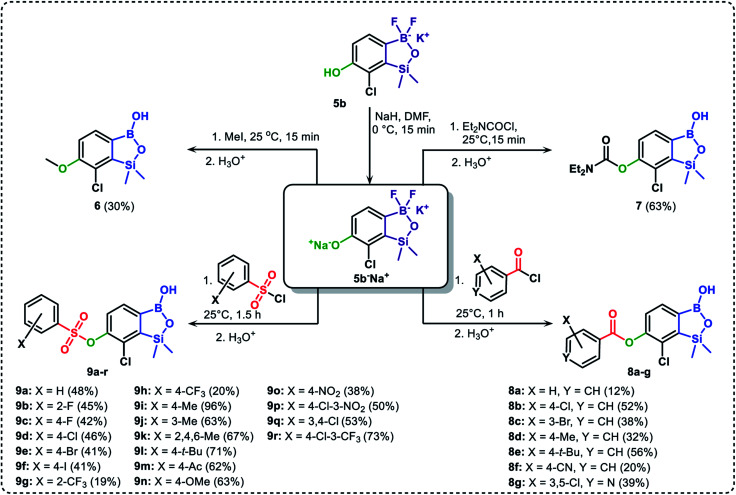
Synthesis of functionalized benzosiloxaboroles 6, 7, 8a–8g, 9a–9r.

The presence of free hydroxyl group in 5b was utilized in various derivatization reactions through initial generation of anionic phenolate species. Various bases including K_2_CO_3_/acetone, NaOH/EtOH and DIPEA (Hünig's base)/THF were tested but they proved ineffective which can be attributed to the poor solubility or degradation of 5b under such conditions. Finally, the use of sodium hydride in anhydrous DMF gave satisfactory results allowing for clean and effective deprotonation of the 6-OH group. Subsequent nucleophilic substitution reactions with MeI, Et_2_NCOCl, benzoyl, and benzenesulfonyl chlorides as electrophilic partners proceeded smoothly under mild conditions (temperature range of 0–25 °C) giving rise to a series of functionalized benzosiloxaboroles 6, 7, 8a–8g, and 9a–9r, respectively (Scheme 2). In addition, we attempted to use the mixture 5a/5a′ using the protocol developed for derivatization of 5b but the results were not satisfactory as we were unable to isolate 7-fluoro analogues of aforementioned products.

**Scheme 3:**
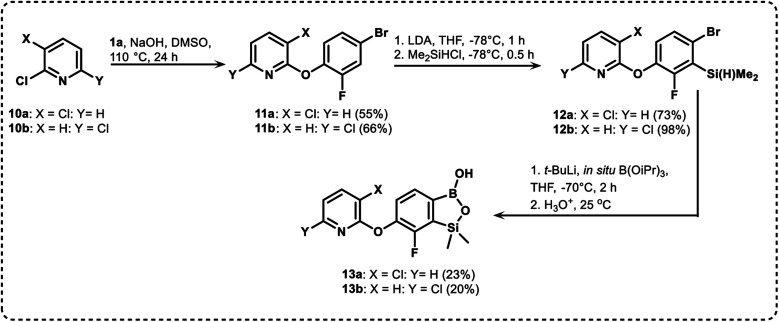
Synthesis of chloropyridin-2-yloxy substituted benzosiloxaboroles 13a–13b.

We have also used dichloropyridines 10a–10b as electrophiles in order to attach the pyridine ring through the ether linkage. Unfortunately, the reactions did not proceed under conditions described above whereas at higher temperatures a tarry mixture was obtained indicating that degradation of starting materials occurred during heating. Thus, we have changed the reactions sequence leading to targeted products 13a–13b (Scheme 3). In the first step, 10a–10b were subjected to S_N_2Ar reactions with the phenolate anion generated from 1b using NaOH/DMSO at 100 °C.^32^ The obtained halogenated phenoxypyridines 11a–11b were converted to respective dimethylsilyl derivatives 12a–12b followed by final transformation to benzosiloxaboroles 13a–13b; both steps were carried out using a protocol described for preparation of 4a–4b from 2a–2b.

### 2.2. Compound characterization

**Fig. 3:**
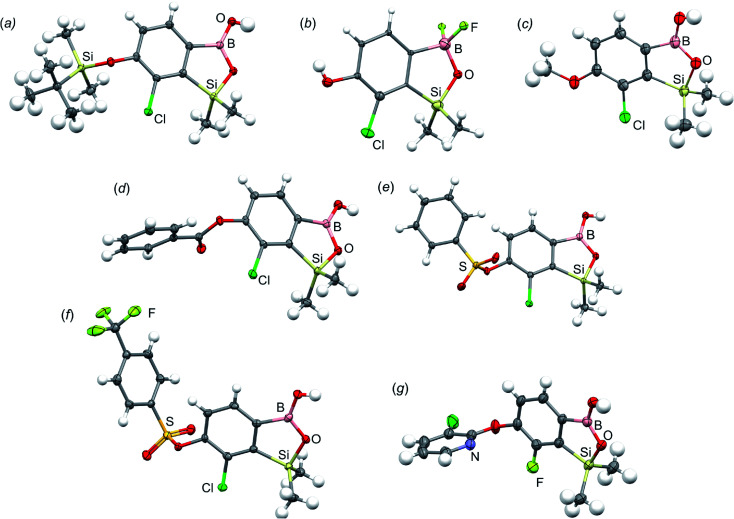
Molecular structures of (a) 4b, (b) 5b, (c) 6, (d) 8a, (e) 9a, (f) 9h and (g) 13a. Thermal motions given as ADPs at the 50% probability level. In the case of the potassium salt 5b only the organoboron anion is presented.

All final benzosiloxaboroles were obtained and fully characterized by multinuclear NMR spectroscopy and HRMS analysis. The ^11^B NMR spectrum of the salt 5b in DMSO-*d*_6_ showed a broadened resonance at 5.5 ppm consistent with the presence of tetracoordinate boron atom whereas the respective ^19^F NMR spectrum showed a signal at −133.60 ppm indicating the attachment of fluorides. In addition, X-ray diffraction analysis of the salt 5b confirmed the tetrahedral arrangement of the boron atom (Fig. 3b) whereas the geometry of the entire boracyclic ring in the benzosiloxaborolate anion is slightly different than that in neutral benzosiloxaboroles,^23^ mainly due to elongation of the B–O distance. The structural formulation of selected benzosiloxaboroles 4b, 6, 8a, 9a, 9h and 13a was also confirmed by single-crystal X-ray diffraction analyses (Fig. 3a and c–g). The metric features of five-membered boracyclic rings in all studied structures are similar to those found previously in analogous compounds (Table S3, see ESI†).^23^ In most cases, the molecules tend to form centrosymmetric dimers due to formation of intermolecular hydrogen bonds between BOH groups (Fig. S84, ESI†). Exceptionally, in the case of 13b, the dimer is formed by O–H⋯N hydrogen bonds between B(OH) hydroxyl group and pyridine nitrogen atom (Fig. S85, ESI†). Furthermore, the acidity (p*K*_a_ values) of selected derivatives was determined by potentiometric titration with 0.05 M aq. NaOH in water/methanol solution (1 : 2). The results (see Table 1) indicate that the benzenesulfonate derivatives (9a, 9c, 9k, 9o) exhibit the highest acidity in the studied series (p*K*_a_ in the range 5.4–6.1) which depends to some extent on the structure of the pendant aryl substituent. This is in agreement with the strong electron-withdrawing effect of the benzenesulfonate group. The benzoate derivatives are slightly weaker acids (p*K*_a_ in the range 6.4–6.6) whereas acidity of 4b (p*K*_a_ = 7.6) is decreased due to strong electron-donating character of TBDMSO group. Overall, the obtained p*K*_a_ values indicate that the most of studied compounds tend strongly to exist as corresponding anions under standard physiological conditions (pH = 7.4), which should enhance their solubility.

**Table 1:** Acidity (p*K*_a_ values) of some obtained benzosiloxaboroles^a^

	4b	8a	8b	9a	9b	9c	9d	9k	9n	9o	13a
p*K*_a_	7.6	6.6	6.4	5.6	5.6	5.4	5.4	6.1	6.0	5.8	6.7

a Determined by potentiometric titration with 0.05 M NaOH in MeOH/H_2_O (2 : 1).

### 2.3. Antibacterial activity

It has been shown recently, that sulfonamide-substituted benzoxaboroles have high activity against *S. aureus* including methicillin-resistant *S. aureus* ATCC BAA-1762 strain.^33^ The MIC values from 0.4 to 6.25 mg L^−1^ were obtained for the most active compounds. Besides, we have previously presented the antibacterial activity, also against Gram-positive cocci, of the several benzosiloxaboroles.^26^ In this study we have investigated antimicrobial activity of the following groups of the newly synthesized benzosiloxaboroles: Group I (TBDMSO derivatives 4a–4b), Group II (benzoyloxy derivatives 8a–8g), Group III (benzenesulfonyloxy 9a–9r) and Group IV (chloropyridin-2-yloxy derivatives 13a–13b). All obtained data for the new derivatives of benzosiloxaboroles and the reference agents are presented in the ESI in Tables S4–S6†. In general, the compounds from the Group III (9a–9r) showed the highest antibacterial activity towards cocci of the *Staphylococcus* genus, especially *S. aureus*. It is worth to underline, that study was carried out with methicillin-sensitive *S. aureus* ATCC 6538P and methicillin-resistant *S. aureus* ATCC 43300. 14 out of 16 well soluble compounds from the Group III showed the high activity against methicillin-sensitive as well as methicillin-resistant *S. aureus* strains, with the MIC values in the range of 0.39–3.12 mg L^−1^ (Table 2, for full data set see Table S4†).

**Table 2:** The MIC values of selected new compounds against standard Gram-positive strains^a^

Compound	MIC [mg L^−1^]				
	*S. aureus* ATCC 6538P MSSA	*S. aureus* ATCC 43300 MRSA	*S. epidermidis* ATCC 12228	*E. faecalis* ATCC 29212	*E. faecium* ATCC 6057
6	50	50	50	200	50
7	12.5	12.5	50	200	200
8a	12.5	25	25	50	50
8f	12.5	12.5	12.5	50	50
8g	100	100	100	400	400
9a	**1.56**	**1.56**	12.5	50	50
9b	**3.12**	**3.12**	12.5	50	50
9c	**3.12**	**3.12**	12.5	50	50
9d	**0.78**	**1.56**	**3.12**	12.5	12.5
9e	**1.56**	**1.56**	6.25	12.5	25
9g	**1.56**	**1.56**	6.25	25	25
9h	**0.39**	**1.56**	**3.12**	25	25
9i	**1.56**	**1.56**	6.25	25	25
9j	**1.56**	**1.56**	6.25	25	25
9k	**0.78**	**1.56**	**3.12**	6.25	6.25
9m	**3.12**	**3.12**	6.25	50	25
9n	**1.56**	**1.56**	12.5	50	50
9o	**1.56**	**3.12**	**0.78**	50	12.5
9p	**3.12**	**3.12**	**3.12**	25	25
9q	**0.78**	**0.78**	**3.12**	6.25	6.25
9r	**0.39**	**0.39**	**3.12**	6.25	6.25
13a	25	25	25	100	50
13b	25	50	25	50	50
**LIN** b	1	2	1	2	2

a The highest activity indicated by the low MIC values (≤3.12 mg L^−1^) is shown in boldface.

b LIN, linezolid was used as a reference agent active against Gram-positive bacteria.

Interestingly, compounds 9k, 9q and 9r showed relatively high activity also against other Gram-positive cocci such as *Enterococcus faecalis* ATCC 29212 and *E. faecium* ATCC 6057, with the MIC value of 6.25 mg L^−1^ (Tables 2 and S4†). The activity of new groups of compounds against *Enterococcus* sp. is rarely observed. It is worth emphasizing that *E. faecalis* and *E. faecium* used in our research belong to two species of the genus *Enterococcus* responsible for frequent human infections, including nosocomial infections.^34^ Compounds from the remaining three groups (4a–4b, 8a–8g and 13a–13b) showed lower activity against Gram-positive bacteria as the MIC range was 12.5–400 mg L^−1^ whilst diameters of the growth inhibition zones ranged from 18–24 mm (Tables 2 and S4†). Thus, the substitution of benzosiloxaboroles with benzenesulfonate substituents is necessary to achieve high activity against staphylococci and enterococci. In this study, linezolid – one of the relatively new group of antibacterial drugs belonging to the oxazolidinones, was used as the reference substance. The indications for linezolid treatment are infections caused by multi-drug resistant cocci including both methicillin-resistant staphylococci and glycopeptide-resistant enterococci strains.^27,34^ We have found that five compounds from the Group III were more active than linezolid against MSSA and MRSA strains. The obtained MIC range of these compounds (9d, 9h, 9k, 9q and 9r) was 0.39–0.78 mg L^−1^ for the MSSA strain (linezolid: MIC = 1 mg L^−1^) and 0.39–1.56 mg L^−1^ for the MRSA strain (linezolid: MIC = 2 mg L^−1^) (Table 2). The high activity of these compounds is due to the presence of chloro or trifluoromethyl groups at the *para* position or two such groups at the *meta* and *para* positions of the benzenesulfonate substituent. Also the presence of three methyl groups at the 2,4,6 positions of the benzenesulfonate substituent results in the high activity of 9k.

Contrary to staphylococci, no potency of the obtained sulfonate-substituted benzosiloxaboroles comparable to linezolid was observed against enterococci. In the case of nine derivatives of the parent compound 9a, the activity against *E. faecalis* and *E. faecium* increased from 2- to 8-fold indicative of positive effect of substituents at the benzenesulfonate scaffold. The analysis of the relationship between the activity and the structure of the tested compounds revealed that the presence of two Cl (9q) or Cl and CF_3_ groups (9r) as well as the presence of three Me groups (9k) is necessary to achieve the highest activity against enterococci. However, the activity of these compounds was still 3-fold weaker than that of linezolid.

Examining the antibacterial activity of new compounds, the minimum bactericidal concentration (MBC) can be determined after establishing the MIC value. For most compounds of Groups I, II and IV, the MBC values were high ≥ 200 mg L^−1^. Interestingly, in the case of the tested compounds from Group III, a paradoxical growth effect was observed during the determination of bactericidal activity. This so-called Eagle effect has previously been reported for several antibiotics, such as some β-lactams, glycopeptides, aminoglycosides, quinolones and polymyxins.^35^ This phenomenon was first published for *S. aureus.*^36^ According to the EUCAST and CLSI definitions, the MBC value is the lowest concentration of a agent that kills 99.9% of bacteria.^37,38^ For 11 out of 16 well soluble compounds (9a, 9c, 9d, 9g, 9i, 9j, 9m, 9n, 9p–9r) the two MBC values for both *S. aureus* strains were observed (Table S4†). Following the CLSI guidelines,^38^ the results were read as the low MBC values in range 0.78–12.5 mg L^−1^ for *S. aureus* MSSA and 1.56–25 mg L^−1^ for *S. aureus* MRSA. However unusually, on the plates with samples taken from the wells containing progressively increasing the agent concentrations (from 2- to 4-fold over the first MBC values), a significant increase in the number of growing colonies, as a paradoxical growth effect, was observed. Finally the second MBC value (in the range 25–400 mg L^−1^) was obtained. So far, the mechanisms causing paradoxical bacterial growth with increasing concentrations of antibiotics are not fully elucidated. However, there have also been several *in vivo* studies in animal models to support the occurrence of the Eagle effect.^35^ In addition, two case reports of the Eagle effect observation during the treatment of human bacterial infections have been described. The reduction in the doses of antibiotics resulted in therapeutic success and correlated with a reduction of the bacteria survived in the bloodstream.^35^

Overall, all the studied groups of the newly synthesized benzosiloxaboroles showed no significant activity against Gram-negative rods (Table S5†). Only a few compounds from the Group II and III of benzosiloxaboroles derivatives showed weak activity against *Stenotrophomonas maltophilia* strains (MICs 200–400 mg L^−1^) and *Bordetella bronchiseptica* (MICs 50–400 mg L^−1^). As in our previous publications, we have investigated the contribution of efflux pumps to the resistance of Gram-negative bacilli to the new synthesized compounds.^23,26^ We used the well-known RND efflux pump inhibitor, Phe-Arg-β-naphthylamide (PAβN).^39,40^ It inhibits the activity of efflux systems found in all Gram-negative rods, like *Escherichia coli, Klebsiella pneumoniae, Enterobacter* sp., *Proteus mirabilis*, *Pseudomonas aeruginosa, S. maltophilia* and *Acinetobacter baumannii.*^39–43^ According to the recent publications, we have used lower concentration of PAβN, *i.e.*, 20 mg L^−1^ because the destabilization of bacterial cell covers was observed at higher concentration of this inhibitor.^44–46^ In order to minimize the influence of PAβN on cell covers, the tests were conducted also in the presence of 1 mM MgSO_4_.^44^ Only in the case of six compounds, we showed a significant (4-fold) decrease in the MIC value of the studied compound in the presence of PAβN. These results confirm the lack of activity of the tested benzosiloxaborole derivatives against Gram-negative rods.

### 2.4. Antifungal activity

Inspired by our previous report about high activity of few benzosiloxaborole derivatives against yeast-like fungi, *Candida tropicalis* and *C. guilliermondii*,^23,26^ we have investigated the activity of the newly synthesized benzosiloxaboroles against 5 species of *Candida* and *Saccharomyces cerevisiae* ATCC 9763. For all but one compounds, the antifungal activity determination was performed using the disk diffusion method as well as by an evaluation of the MIC and MFC values. The results of antifungal activity of the newly synthesized benzosiloxaboroles agents are presented in the ESI (Table S6†). The collection strains of *Candida* species which most commonly cause infections in humans were selected for the study. In most cases, they are responsible for opportunistic infections. However, they can cause also nosocomial infections, including severe infections, and cause death, mainly of immunocompromised patients.^47,48^ Compounds from the Group II demonstrated the highest activity against all tested *Candida* species, especially against *C. krusei* and *C. tropicalis*. The presence of methyl group at the *para* position of the phenyl ring (compound 8d) led to 2–4-fold increase in the activity against all tested *Candida* strains. The lowest MIC values 3.12–6.25 mg L^−1^ were observed for two strains of *C. tropicalis*. Other studied benzosiloxaboroles showed relatively high activity only against *S. cerevisiae*, a species that is not clinically significant.

### 2.5. Cytotoxic activity

To evaluate the cytotoxic effect of the tested compounds, MTT-based assay was performed. Human normal lung fibroblasts MRC-5 were treated with the newly synthesized compounds at the concentrations range of 0.78 to 50 mg L^−1^ for 72 h. IC_50_ values describing half inhibitory concentrations of each tested compound were calculated and summarized in Table 3. The representative plots demonstrating sigmoidal dose response curves for the tested compounds were shown in the ESI (Fig. S86–S91†). The obtained IC_50_ values for compounds 9a–9r, 8a–8g, 13a–13b are in the range of 3.19 to above 50 mg L^−1^, with the lowest value for the 4-chloro-3-nitrobenzenesulfonate derivative 9p, and the highest values for compounds 9a, 9g, 5b, 8f and 8g.

**Table 3:** The viability of human normal lung fibroblasts, MRC-5 after 72 h treatment with the tested compounds. Linezolid was used as a reference. The concentration (IC_50_) that causes a response half way between the maximal (top) response and the maximally inhibited (bottom) response was calculated using MTT-based assay data and an equation *Y* = bottom + (top − bottom)/(1 + 10^((log^ ^IC_50_ − *X*) × HillSlope)^)

Compound	IC_50_ [mg L^−1^]	Compound	IC_50_ [mg L^−1^]
9a	>50	5b	>50
9c	49.13 ± 8.93	7	32.04 ± 3.76
9d	24.96 ± 3.37	8a	>50
9e	24.12 ± 9.82	8b	21.67 ± 2.72
9f	25.89 ± 2.00	8d	13.31 ± 3.09
9g	>50	8e	14.30 ± 1.96
9h	21.95 ± 2.24	8f	>50
9i	24.85 ± 4.23	8g	>50
9j	25.00 ± 4.74	13a	40.46 ± 2.70
9k	15.64 ± 3.47	13b	30.91 ± 2.31
9m	27.52 ± 3.61	Linezolid	>50
9n	29.43 ± 5.76		
9o	25.74 ± 3.56		
9p	3.19 ± 1.07		
9q	16.83 ± 3.89		
9r	12.30 ± 4.51		

## 3. Conclusions

In conclusion, simple (MeO) and more structurally extended moieties (Et_2_NCO_2_, ArCO_2_, ArSO_3_, Py-2-O) were successfully installed onto the benzosiloxaborole scaffold using modular approaches based in most cases on the functionalization of the newly obtained potassium 6-hydroxy-7-chloro-1,1-dimethyl-3,3-difluorobenzo-1,2,3-siloxaborolate 5b. It seems that the elaborated protocols can be directly adapted for the synthesis of other benzosiloxaboroles with various substitution patterns. Comprehensive characterization of antimicrobial activity of obtained compounds was performed. Most importantly, selected derivatives showed high activity against Gram-positive cocci from *Staphylococcus* and *Enterococcus* genera with the MIC values for as low as 0.39–0.78 mg L^−1^ and 6.25 mg L^−1^ respectively. SAR analysis comprising derivatives bearing ArCO_2_, ArSO_3_, Py-2-O moieties clearly indicates that the presence of –SO_2_–O– linker between two aromatic units is essential for achieving high antibacterial potency as the MIC values (determined for *S. aureus*) for benzenesulfonate derivatives (Group III) were typically in the range of 0.39–3.12 mg L^−1^. In contrast, compounds from the Group II and IV were significantly less active as the corresponding MIC values were equal to 12.5 mg L^−1^ at best. It should be stressed that the cytotoxicity tests performed for human lung fibroblasts revealed that the IC_50_ values are much higher than MIC values for the majority of analyzed compounds. Importantly, for the most promising cocci-active benzenesulfonate derivatives (except for 9p agent), the obtained MIC values were not cytotoxic towards human normal lung fibroblasts (MRC-5) with IC_50_ values exceeding 12.3 mg L^−1^. To summarize, benzenesulfonate substituted benzosiloxaboroles can be considered as potential candidates for the treatment of infections caused by Gram-positive bacterial pathogens and therefore, extended studies on the topic by our research group are currently in progress.

## 4. Experimental section

### 4.1. General comments

Solvents used for reactions were dried by heating to reflux with sodium/benzophenone and distilled under argon. Starting materials including halogenated phenols (*tert*-butyl)dimethylsilyl chloride (TBDMSCl), alkyllithiums, diisopropylamine, trialkyl borates, chlorodimethylsilane as well as other reagents were used as received without further purification. In the ^13^C NMR spectra the resonances of boron-bound carbon atoms were not observed in most cases as a result of their broadening by a quadrupolar boron nucleus. ^1^H, and ^13^C NMR chemical shifts are given relative to TMS using residual solvent resonances. For signal assignments purposes in selected ^1^H NMR data, Ar^B^, Ar^C^, Ar^S^ stand for boronated benzene, benzoate and benzenesulfonyl fragments, respectively. ^11^B and ^19^F NMR chemical shifts are given relative to BF_3_·Et_2_O and CFCl_3_, respectively. Crystallographic Information Files (CIFs) have been deposited with the Cambridge Crystallographic Data Centre as supplementary publications no. 2068345 (4b), 2068347 (5b), 2069477 (6), 2068346 (8a), 2068348 (9a), 2077619 (9h) and 2068349 (13a). Relevant crystallographic data is provided in Table S1†.

### 4.2. Synthesis

#### 4.2.1 (2a) 4-Bromo-2-fluoro-1-(*tert*-butyldimethylsilyloxy)benzene


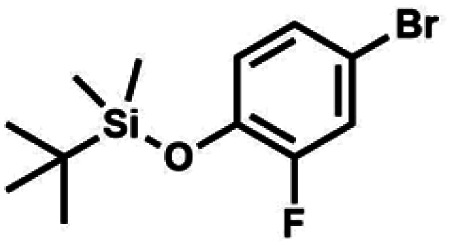
A suspension of NaH in mineral oil (60%, 6.30 g, 157 mmol, 3.0 eq.) under argon atmosphere was washed twice with anhydrous hexane (20 mL) and anhydrous THF (20 mL) was added. The mixture was stirred for 10 min and cooled to 0 °C and solution of 4-bromo-2-fluorophenol (10.0 g, 53.0 mmol, 1.0 eq.) in anhydrous THF (50 mL) was added for 20 min. After *ca.* 15 min stirring in 0 °C it was warmed to room temperature and solution of TBDMSCl (8.4 g, 55.0 mmol, 1.1 eq.) in Et_2_O (30 mL) was added to the white suspension for 10 min. It was stirred for another 2 h at room temperature, then it was evaporated to dryness under reduced pressure and the residue was subjected to a simple distillation under reduced pressure. The product was obtained as a yellow liquid, bp 70 °C/5 10^−3^ mbar. Yield 13.4 g (83%). ^1^H NMR (400 MHz, CDCl_3_) *δ* 7.22 (dd, *J* = 10.1, 2.4 Hz, 1H, Ar), 7.12 (ddd, *J* = 8.6, 2.4, 1.5 Hz, 1H, Ar), 6.80 (t, *J* = 8.7 Hz, 1H, Ar), 1.01 (s, 9H, *t*-Bu), 0.20 (d, *J* = 1.2 Hz, 6H, SiMe_2_) ppm. ^13^C NMR (101 MHz, CDCl_3_) *δ* 154.20 (d, *J* = 249.2 Hz), 142.8 (d, *J* = 12.4 Hz), 127.4 (d, *J* = 3.9 Hz), 123.4 (d, *J* = 2.5 Hz), 119.9 (d, *J* = 22.1 Hz), 112.8 (d, *J* = 8.2 Hz), 25.5, 18.3, −4.7 (d, *J* = 1.8 Hz) ppm. Anal. calcd for C_12_H_18_BrFOSi (305.26): C 47.22, H 5.94; found C 47.27, H 5.88.

#### 4.2.2 (2b) 4-Bromo-2-chloro-1-(*tert*-butyldimethylsilyloxy)benzene


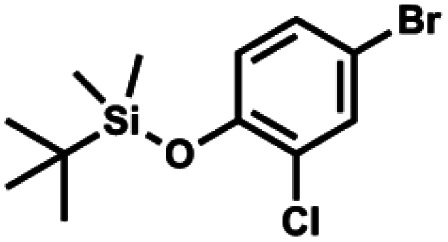
A solution of 4-bromo-2-chlorophenol (51.86 g, 0.250 mol, 1.0 eq.), TBDMSCl (41.45 g, 0.275 mol, 1.1 eq.) and Et_3_N (52.3 mL, 0.375 mol, 1.5 eq.) in Et_2_O (300 mL) was stirred under argon atmosphere for 24 h at room temperature. Obtained white suspension was evaporated to dryness under reduced pressure. The residue was triturated with heptane (400 mL) followed by filtration under reduced pressure. The yellow filtrate was evaporated under reduced pressure and the residue was subjected to a fractional distillation under reduced pressure. The product was obtained as a yellow liquid, bp 106–115 °C/5 10^−3^ mbar. Yield 74.3 g (92%). ^1^H NMR (400 MHz, CDCl_3_) *δ* 7.48 (d, *J* = 2.5 Hz, 1H, Ar), 7.23 (dd, *J* = 8.7, 2.5 Hz, 1H, Ar), 6.76 (d, *J* = 8.6 Hz, 1H, Ar), 1.03 (s, 9H, *t*-Bu), 0.22 (s, 6H, SiMe_2_) ppm. ^13^C NMR (101 MHz, CDCl_3_) *δ* 151.1, 132.8, 130.6, 126.9, 122.0, 113.4, 25.8, 18.4, −4.2 ppm. Anal. calcd for C_12_H_18_BrClOSi (321.71): C 44.80, H 5.64; found C 44.69, H 5.53.

#### 4.2.3 (3a) 4-Bromo-2-fluoro-3-(dimethylsilyl)-1-(*tert*-butyldimethylsilyloxy)benzene


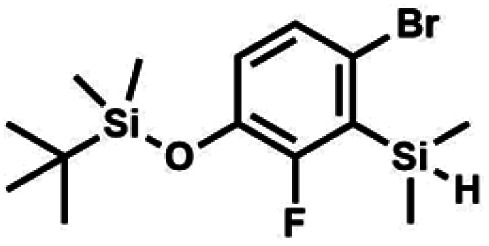
This compound was obtained using the protocol described for 3b using 2a (12.2 g, 40.0 mmol, 1.0 eq.), *n*-BuLi (11 M, 4 mL, 44.0 mmol, 1.1 eq.), *i*Pr_2_NH (6.2 mL, 44.0 mmol, 1.1 eq.) and Me_2_SiHCl (4.8 mL, 44.0 mmol, 1.1 eq.) as the starting materials. It was obtained as a white powder. bp 95–100 °C. Yield 13.7 g (95%). ^1^H NMR (300 MHz, CDCl_3_) *δ* 7.19 (dd, *J* = 8.5, 1.4 Hz, 1H, Ar), 6.79 (dd, *J* = 9.2, 8.5 Hz, 1H, Ar), 4.79–4.73 (m, 1H, SiH), 1.01 (d, *J* = 0.5 Hz, 9H, *t*-Bu), 0.46 (dd, *J* = 3.9, 1.8 Hz, 6H, Si(H)
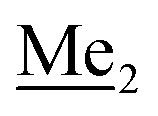
), 0.20 (d, *J* = 1.1 Hz, 6H, Si(*t*-Bu)
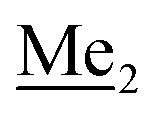
) ppm. ^13^C NMR (101 MHz, CDCl_3_) *δ* 158.50 (d, *J* = 243.5 Hz), 142.50 (d, *J* = 16.6 Hz), 129.0 (d, *J* = 3.7 Hz), 126.5 (d, *J* = 30.0 Hz), 124.4 (d, *J* = 3.0 Hz), 120.30 (d, *J* = 10.9 Hz), 25.6, 18.4, −3.1 (d, *J* = 4.3 Hz), −4.7 (d, *J* = 1.9 Hz) ppm. ^19^F NMR (282 MHz, CDCl_3_) *δ* −114.09 (t, *J* = 7.1 Hz) ppm. Anal. calcd for C_14_H_24_BrFOSi_2_ (363.42): C 46.27, H 6.66; found C 46.12, H 6.58.

#### 4.2.4 (3b) 4-Bromo-2-chloro-3-(dimethylsilyl)-1-(*tert*-butyldimethylsiloxy)benzene


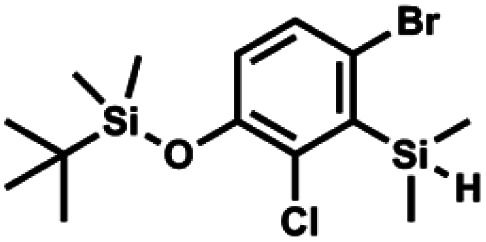
A solution of 2b (74.30 g, 231.00 mmol, 1.0 eq.) in Et_2_O (70 mL) was added dropwise at −75 °C for 15 min to a stirred solution of LDA, freshly prepared from diisopropylamine (35.6 mL, 254.1 mmol, 1.1 eq.) and *n-*BuLi (2.5 M, 101.64 mL, 254.10 mmol, 1.1 eq.) in THF (400 mL). The solution turned lucid yellow. After *ca.* 1.25 h stirring at −75 °C chlorodimethylsilane (30.8 mL, 277.2 mmol, 1.2 eq.) was added slowly for 15 min and the formation of thick slurry was observed. It was stirred for another 15 min at −75 °C, then it was allowed to warm to room temperature. The obtained white suspension was evaporated to dryness under reduced pressure. The residue was triturated with heptane (200 mL) followed by filtration. The yellow filtrate was evaporated under reduced pressure and the residue was subjected to a simple distillation under reduced pressure. The product was obtained as a yellow liquid, bp 125–130 °C (5 10^−3^ mbar). Yield 84.3 g (96%). ^1^H NMR (400 MHz, CDCl_3_) *δ* 7.31 (d, *J* = 8.6 Hz, 1H, Ar), 6.73 (d, *J* = 8.5 Hz, 1H, Ar), 5.02 (sept, *J* = 3.9 Hz, 1H, SiH), 1.02 (s, 9H, *t*-Bu), 0.49 (d, *J* = 4.0 Hz, 6H, Si(H)
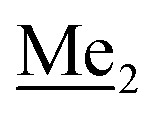
), 0.22 (s, 6H, Si(*t*-Bu)
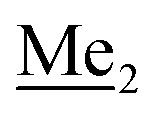
) ppm. ^13^C NMR (101 MHz, CDCl_3_) *δ* 150.7, 138.8, 133.3, 132.2, 122.4, 121.4, 25.6, 18.3, −2.6, −4.4 ppm. Anal. calcd for C_14_H_24_BrClOSi_2_ (379.87): C 44.27, H 6.37; found C 44.19, H 6.32.

#### 4.2.5 (4a) 6-(*tert*-Butyldimethylsilyloxy)-7-fluoro-1,1-dimethyl-3-hydroxybenzo-1,2,3-siloxaborole


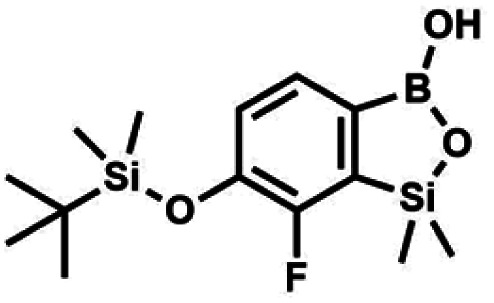
This compound was obtained using the protocol described for 4b using 3a (12.1 g, 33.0 mmol, 1.0 eq.), *t*-BuLi (1.7 M, 39 mL, 66.0 mmol, 2.0 eq.) and B(O*i*Pr)_3_ (14.0 mL, 50.0 mmol, 1.5 eq.) as the starting materials. It was obtained as a white powder. mp 84 °C. Yield 9.5 g (88%). ^1^H NMR (300 MHz, CDCl_3_) *δ* 7.43 (dd, *J* = 7.7, 0.6 Hz, 1H, Ar), 7.01 (t, *J* = 7.8 Hz, 1H, Ar), 1.01 (s, 9H, *t*-Bu), 0.48 (s, 6H, OSiMe_2_), 0.22 (d, *J* = 1.0 Hz, 6H, Si (*t*-Bu)
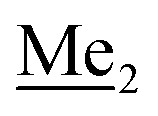
) ppm. ^13^C NMR (101 MHz, CDCl_3_) *δ* 155.4 (d, *J* = 243.4 Hz), 145.4 (d, *J* = 14.9 Hz), 136.2 (d, *J* = 28.5 Hz), 128.5 (d, *J* = 3.1 Hz), 125.0 (d, *J* = 1.4 Hz), 25.5, 18.3, −0.7, −4.7 (d, *J* = 1.9 Hz). ^11^B NMR (96 MHz, CDCl_3_) *δ* 30.5 ppm. ^19^F NMR (282 MHz, CDCl_3_) *δ* −123.40 (d, *J* = 7.9 Hz) ppm. HRMS (ESI, positive ion mode): calcd for C_14_H_25_BFO_3_Si_2_^+^ [MH]^+^ 327.1414; found 327.1416.

#### 4.2.6 (4b) 6-(*tert*-Butyldimethylsilyloxy)-7-chloro-1,1-dimethyl-3-hydroxybenzo-1,2,3-siloxaborole


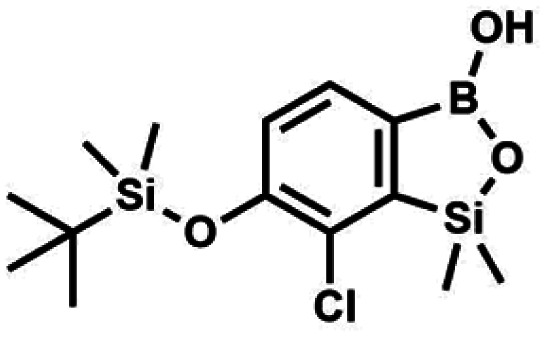
A solution of *t*-BuLi (1.9 M, 150 mL, 285.0 mmol, 2.1 eq.) was added dropwise at −75 °C for 30 min to a stirred solution of 3b (51.55 g, 135.71 mmol, 1.0 eq.) and B(O*i*Pr)_3_ (27.7 mL, 162.9 mmol, 1.2 eq.) in anhydrous THF (400 mL) under argon atmosphere. After *ca.* 30 min stirring at −75 °C a thick slurry was formed. It was stirred for another 1.5 h at −75 °C and warmed to −10 °C, quenched with water and stirred at room temperature until evolution of H_2_ ceased. 1.5 M aq. H_2_SO_4_ was dropped to reach the pH = 2–3. Et_2_O (150 mL) and brine (50 mL) were added. The aqueous phase was separated followed by extraction with Et_2_O (2 × 100 mL). The extracts were added to the organic phase and dried with anhydrous MgSO_4_. Then it was concentrated under reduced pressure. An oily residue was mixed with water and hexane resulting in the formation of a white slurry. The white solid was filtered, washed several times with water and dried *in vacuo*, to give the product, mp 84–85 °C. Yield 43.3 g (93%). ^1^H NMR (400 MHz, CDCl_3_) *δ* 7.56 (d, *J* = 7.8 Hz, 1H, Ar), 6.96 (d, *J* = 7.8 Hz, 1H, Ar), 1.04 (s, 9H, *t*-Bu), 0.49 (s, 6H, OSiMe_2_), 0.26 (s, 6H, Si (*t*-Bu)
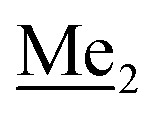
) ppm. ^13^C NMR (101 MHz, CDCl_3_) *δ* 153.7, 151.9, 131.1, 128.2, 122.7, 25.7, 18.4, −1.4, −4.2 ppm. HRMS (ESI, positive ion mode): calcd for C_14_H_25_BClO_3_Si_2_^+^ [MH]^+^ 343.1118; found 343.1119.

#### 4.2.7 (5b) Potassium 7-chloro-6-hydroxy-3,3-difluorobenzo-1,2,3-siloxaborolate


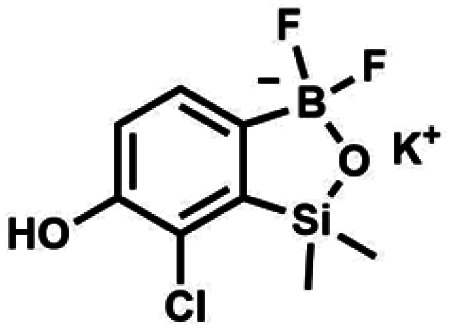
A solution of KHF_2_ in water (5 M, 115 mL, 574 mmol, 6.7 eq.) was added to a stirred solution of 4b (29.3 g, 85.5 mmol, e. 1.0) in MeOH (200 mL) in a sealed polypropylene beaker. After 24 h of stirring at room temperature it was concentrated under reduced pressure and obtained residue was suspended in DMF. It was filtered and concentrated under reduced pressure. The obtained white solid was dried *in vacuo* at 70 °C, mp 248–249 °C. Yield 19.0 g (77%). ^1^H NMR (400 MHz, DMSO-*d*_6_) *δ* 9.29 (s, 1H, OH), 6.97 (d, *J* = 7.5 Hz, 1H, Ar), 6.75 (d, *J* = 7.5 Hz, 1H, Ar), 0.15 (s, 6H, SiMe_2_) ppm. ^13^C NMR (101 MHz, DMSO-*d*_6_) *δ* 162.3, 150.0, 146.3, 127.7 (t, *J* = 3.0 Hz), 121.3, 117.4, 1.0 ppm. ^19^F NMR (376 MHz, DMSO-*d*_6_) *δ* −133.65 ppm. ^11^B NMR (96 MHz, DMSO) *δ* 5.8 ppm. HRMS (ESI, negative ion mode): calcd for C_8_H_9_BClF_2_O_2_Si^−^ [M − K]^−^ 249.0127; found 249.0125.

#### 4.2.8 (6) 7-Chloro-6-methoxy-1,1-dimethyl-3-hydroxybenzo-1,2,3-siloxaborole


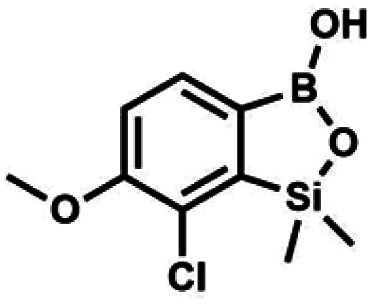
A suspension of NaH in mineral oil (60%, 0.30 g, 7.50 mmol, 2.1 eq.) under argon atmosphere was washed with anhydrous hexane (5 mL) twice and anhydrous DMF (10 mL) was added and stirred for 10 min. Then it was cooled to 0 °C and 5b (1.00 g, 3.50 mmol, 1.0 eq.) was added. After *ca.* 30 min stirring in 0 °C, iodomethane (0.45 mL, 7.00 mmol, 2.0 eq.) was added to the white suspension. It was stirred for 1.0 h at room temperature and concentrated by a simple distillation under reduced pressure. The solid residue was treated with 1.5 M aq. H_2_SO_4_ to reach the pH = 2–3. The obtained white solid was filtered and washed with water (5 mL) and hexane (2 × 5 mL) and dried to give the product, mp 84–85 °C. Yield 0.25 g (30%). ^1^H NMR (400 MHz, CDCl_3_) *δ* 7.68 (d, *J* = 8.0 Hz, 1H, Ar), 7.02 (d, *J* = 8.0 Hz, 1H, Ar), 3.94 (s, 3H, OMe), 0.50 (s, 6H, SiMe_2_) ppm. ^13^C NMR (101 MHz, CDCl_3_) *δ* 156.9, 151.5, 131.6, 125.1, 114.2, 56.3, −1.3 ppm. HRMS (ESI, positive ion mode): calcd for C_9_H_13_BClO_3_Si^+^ [MH]^+^ 243.0410; found 243.0412.

#### 4.2.9 (7) 7-Chloro-6-(*N*,*N*-diethylcarbamoyloxy)-1,1-dimethyl-3-hydroxybenzo-1,2,3-siloxaborole


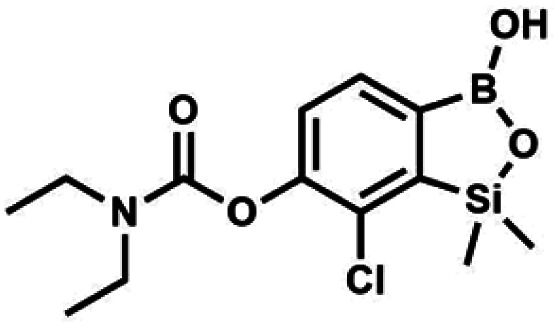
This compound was obtained using the protocol described for 6 using suspension of NaH in mineral oil (60%, 0.14 g, 3.50 mmol, 2.0 eq.), 5b (0.5 g, 1.7 mmol, 1.0 eq.) and *N*,*N*-diethylcarbamoyl chloride (0.44 mL, 3.5 mmol, 2.1 eq.) as the starting materials. It was obtained as a white powder, mp 115–117 °C. Yield 0.35 g (63%). ^1^H NMR (300 MHz, CDCl_3_) *δ* 7.66 (d, *J* = 7.8 Hz, 1H, Ar), 7.29 (d, *J* = 7.8 Hz, 1H, Ar), 5.21 (s, 1H, OH), 3.52 (q, *J* = 7.0 Hz, 2H, CH_2_), 3.43 (q, *J* = 7.0 Hz, 2H, CH_2_), 1.33 (t, *J* = 7.0 Hz, 3H, Me), 1.25 (t, *J* = 7.0 Hz, 3H, Me), 0.49 (s, 6H, SiMe_2_) ppm. ^13^C NMR (101 MHz, CDCl_3_) *δ* 153.1, 151.7, 149.4, 130.9, 126.2, 42.47, 42.1, 14.1, 13.3, −1.4 ppm. ^11^B NMR (96 MHz, CDCl_3_) *δ* 31.4 ppm. HRMS (ESI, positive ion mode): calcd for C_13_H_20_BClNO_4_Si^+^ [MH]^+^ 328.0938; found 328.0940.

#### 4.2.10 (8a) 7-Chloro-6-benzoyloxy-1,1-dimethyl-3-hydroxybenzo-1,2,3-siloxaborole


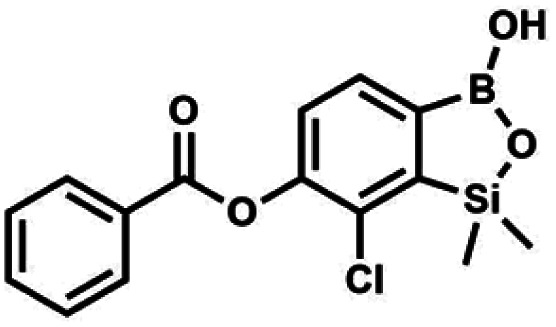
This compound was obtained using the protocol described for 6 using suspension of NaH in mineral oil (60%, 0.10 g, 2.50 mmol, 1.8 eq.), 5b (0.5 g, 1.7 mmol, 1.0 eq.) and benzoyl chloride (0.24 mL, 2.0 mmol, 1.2 eq.) as the starting materials. It was obtained as a white powder. mp 150–152 °C. Yield 0.07 g (12%). ^1^H NMR (400 MHz, CDCl_3_) *δ* 8.24 (d, *J* = 7.8 Hz, 2H, Ar^C^), 7.75 (d, *J* = 7.7 Hz, 1H, Ar^B^), 7.67 (t, *J* = 7.5 Hz, 1H, Ar^C^), 7.54 (t, *J* = 7.6 Hz, 2H, Ar^C^), 7.36 (d, *J* = 7.5 Hz, 1H, Ar^B^), 0.52 (s, 6H, SiMe_2_) ppm. ^13^C NMR (101 MHz, CDCl_3_) *δ* 164.4, 151.9, 149.2, 134.1, 131.3, 130.5, 130.3, 129.8, 128.8, 128.6, 126.1, −1.2 ppm. HRMS (ESI, positive ion mode): calcd for C_15_H_15_BClO_4_Si^+^ [MH]^+^ 333.0516; found 333.0518.

#### 4.2.11 (8b) 7-Chloro-6-(4′-chlorobenzoyloxy)-1,1-dimethyl-3-hydroxybenzo-1,2,3-siloxaborole


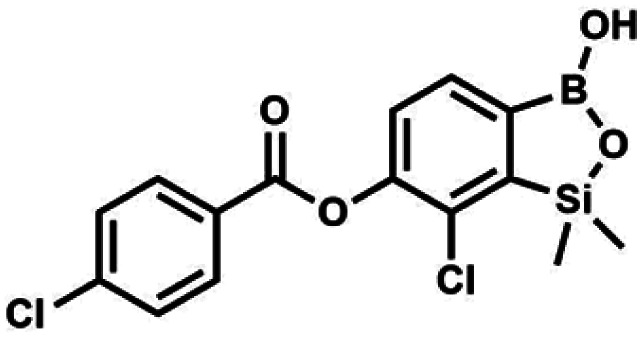
This compound was obtained using the protocol described for 8d using suspension of NaH in mineral oil (60%, 0.14 g, 3.50 mmol, 2.0 eq.), 5b (0.5 g, 1.70 mmol, 1.0 eq.) and 4-chlorobenzoyl chloride (0.22 mL, 1.70 mmol, 1.0 eq.) as the starting materials. It was obtained as a white powder. mp 161–164 °C. Yield 0.32 g (52%). ^1^H NMR (400 MHz, CDCl_3_) *δ* 8.18 (d, *J* = 8.5 Hz, 2H, Ar^C^), 7.76 (d, *J* = 7.7 Hz, 1H, Ar^B^), 7.52 (d, *J* = 8.5 Hz, 2H, Ar^C^), 7.36 (d, *J* = 7.7 Hz, 1H, Ar^B^), 5.48 (s, 1H, OH), 0.53 (s, 6H, SiMe_2_). ^13^C NMR (101 MHz, CDCl_3_) *δ* 163.4, 152.0, 148.8, 140.6, 131.7, 131.2, 129.5, 129.1, 127.2, 125.8, −1.4 ppm. HRMS (ESI, positive ion mode): calcd for C_15_H_14_BCl_2_O_4_Si^+^ [MH]^+^ 367.0126; found 367.0126.

#### 4.2.12 (8c) 7-Chloro-6-(3′-bromobenzoyloxy)-1,1-dimethyl-3-hydroxybenzo-1,2,3-siloxaborole


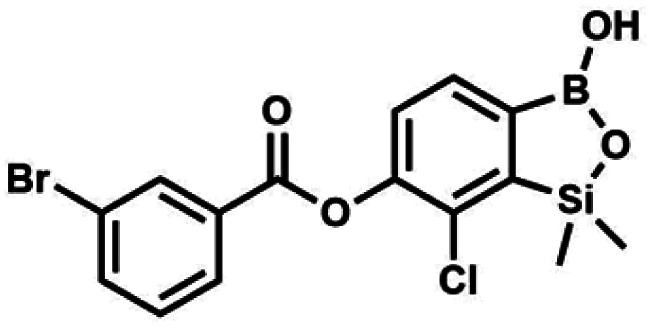
This compound was obtained using the protocol described for 8d using suspension of NaH in mineral oil (60%, 0.14 g, 3.50 mmol, 2.0 eq.), 5b (0.5 g, 1.70 mmol, 1.0 eq.) and 3-bromobenzoyl chloride (0.24 mL, 1.70 mmol, 1.0 eq.) as the starting materials. It was obtained as a white powder. Yield 0.25 g (38%). ^1^H NMR (400 MHz, CDCl_3_) *δ* 8.38 (s, 1H, Ar^C^), 8.17 (d, *J* = 7.8 Hz, 1H, Ar^C^), 7.80 (d, *J* = 7.8 Hz, 1H, Ar^C^), 7.75 (d, *J* = 7.7 Hz, 1H, Ar^B^), 7.42 (t, *J* = 7.9 Hz, 1H, Ar^C^), 7.35 (d, *J* = 7.7 Hz, 1H, Ar^B^), 4.86 (s, 1H), 0.52 (s, 6H, SiMe_2_) ppm. ^13^C NMR (101 MHz, CDCl_3_) *δ* 162.9, 152.3, 148.7, 136.9, 133.3, 131.1, 130.7, 130.2, 129.5, 128.9, 125.7, 122.7, −1.4 ppm. HRMS (ESI, positive ion mode): calcd for C_15_H_14_BBrClO_4_Si^+^ [MH]^+^ 410.9621; found 410.9621.

#### 4.2.13 (8d) 7-Chloro-6-(4′-methylbenzoyloxy)-1,1-dimethyl-3-hydroxybenzo-1,2,3-siloxaborole


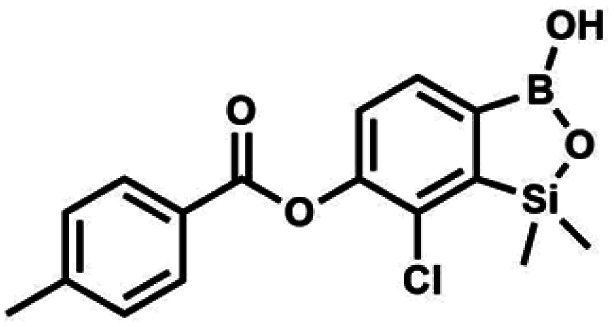
Suspension of NaH in mineral oil (60%, 0.14 g, 3.50 mmol, 2.0 eq.) under argon atmosphere was washed with anhydrous hexane (5 mL) twice and anhydrous DMF (10 mL) was added and stirred for 10 min. Then it was cooled to 0 °C and 5b (0.5 g, 1.70 mmol, 1.0 eq.) was added. After *ca.* 30 min stirring in 0 °C, 4-toluoyl chloride (0.22 mL, 1.70 mmol, 1.0 eq.) was added to the white suspension. It was stirred for another 1.0 h at room temperature, then concentrated by a simple distillation under reduced pressure. The solid residue was treated with 1.5 M aq. H_2_SO_4_ to reach the pH = 2–3. The obtained white solid was filtered and washed with water (2 × 5 mL) and hexane (2 × 5 mL). Aqueous NaHCO_3_ (5 wt% in water, 2 mL) was added and the suspension was stirred for *ca.* 30 min. The white solid product was filtered and washed with water (2 × 2 mL). The product was dried *in vacuo*, mp 140–145 °C. Yield 0.19 g (32%). ^1^H NMR (400 MHz, CDCl_3_) *δ* 8.14 (d, *J* = 8.1 Hz, 2H, Ar^C^), 7.75 (d, *J* = 7.7 Hz, 1H, Ar^B^), 7.38–7.32 (m, 3H, Ar^C^ + Ar^B^), 5.35 (s, 1H, OH), 2.47 (s, 3H, Me), 0.53 (s, 6H, SiMe_2_) ppm. ^13^C NMR (101 MHz, CDCl_3_) *δ* 164.3, 151.9, 149.1, 131.1, 130.5, 130.2, 129.7, 129.4, 129.2, 126.01, 125.99, 21.8, −1.4 ppm. HRMS (ESI, positive ion mode): calcd for C_16_H_17_BClO_4_Si^+^ [MH]^+^ 347.0672; found 347.0674.

#### 4.2.14 (8e) 7-Chloro-6-[4′-(*tert*-butyl)benzoyloxy]-1,1-dimethyl-3-hydroxybenzo-1,2,3-siloxaborole


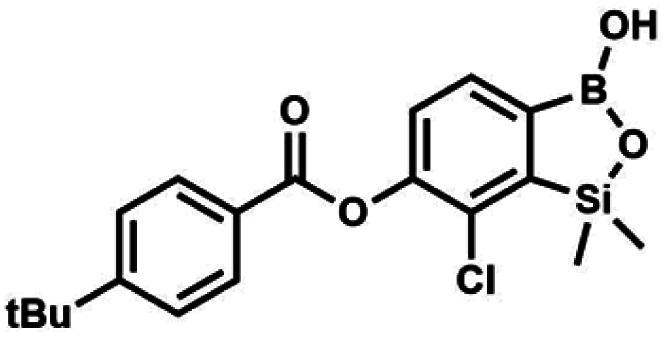
A suspension of NaH in mineral oil (60%, 0.14 g, 3.50 mmol, 2.0 eq.) under argon atmosphere was washed twice with anhydrous hexane (5 mL) and anhydrous DMF (10 mL) was added. The mixture was stirred for 10 min, cooled to 0 °C and 5b (0.50 g, 1.70 mmol, 1.0 eq.) was added. After *ca.* 30 min stirring in 0 °C, 4-*tert-*butylbenzoyl chloride (0.34 mL, 1.70 mmol, 1.0 eq.) was added to the white suspension. It was stirred for another 1.0 h at room temperature, and concentrated by a simple distillation under reduced pressure. The solid residue was treated with 1.5 M aq. H_2_SO_4_ to reach the pH = 2–3. Et_2_O (15 mL) and brine (10 mL) were added, and the aqueous phase was separated followed by the extraction with Et_2_O (2 × 10 mL). The extracts were added to the organic phase and dried with anhydrous MgSO_4_. Then it was concentrated under reduced pressure. The solid was filtered and washed with water (2 × 5 mL) and hexane (2 × 5 mL). Aqueous NaHCO_3_ (5 wt%, 2 mL) was added and the suspension was stirred for *ca.* 30 min. The white solid was filtered, washed with water (2 × 2 mL) and dried *in vacuo*. Yield 0.36 g (56%). ^1^H NMR (400 MHz, CDCl_3_) *δ* 8.18 (d, *J* = 8.8 Hz, 2H, Ar^C^), 7.76 (d, *J* = 7.7 Hz, 1H, Ar^B^), 7.56 (d, *J* = 8.9 Hz, 2H, Ar^C^), 7.35 (d, *J* = 7.7 Hz, 1H, Ar^B^), 5.46 (s, 1H, OH), 1.38 (s, 9H, *t*-Bu), 0.53 (s, 6H, SiMe_2_) ppm. ^13^C NMR (101 MHz, CDCl_3_) *δ* 164.2, 157.8, 151.9, 149.1, 131.1, 130.3, 129.7, 127.0, 126.0, 125.9, 125.7, 125.2, 35.3, 31.1, −1.4 ppm. HRMS (ESI, positive ion mode): calcd for C_19_H_23_BClO_4_Si^+^ [MH]^+^: 389.1142; found 389.1142.

#### 4.2.15 (8f) 7-Chloro-6-(4′-cyanobenzoyloxy)-1,1-dimethyl-3-hydroxybenzo-1,2,3-siloxaborole


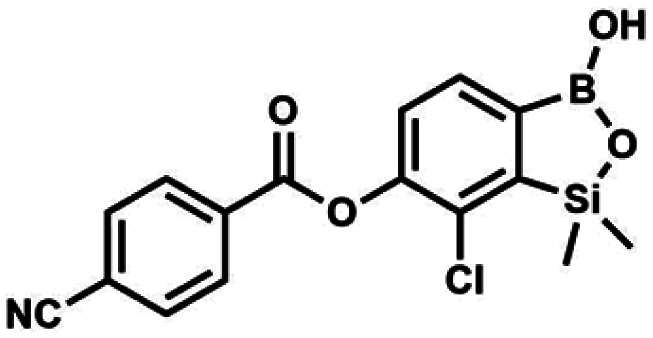
This compound was obtained using the protocol described for 8d using suspension of NaH in mineral oil (60%, 0.14 g, 3.50 mmol, 2.0 eq.), 5b (0.50 g, 1.70 mmol, 1.0 eq.) and 4-cyanobenzoyl chloride (0.28 g, 1.70 mmol, 1.0 eq.) as the starting materials. It was obtained as a white powder. mp 160–165 °C. Yield 0.12 g (20%). ^1^H NMR (400 MHz, CDCl_3_) *δ* 8.35 (d, *J* = 8.0 Hz, 2H, Ar^C^), 7.85 (d, *J* = 8.0 Hz, 2H, Ar^C^), 7.77 (d, *J* = 7.8 Hz, 1H, Ar^B^), 7.36 (d, *J* = 7.7 Hz, 1H, Ar^B^), 4.96 (s, 1H, O̲*H*), 0.52 (s, 6H, SiMe_2_) ppm. ^13^C NMR (101 MHz, CDCl_3_) *δ* 162.7, 152.4, 148.5, 132.6, 132.5, 131.2, 130.8, 129.3, 127.7, 125.5, 117.7, 117.3, −1.4 ppm. HRMS (ESI, negative ion mode) calcd for C_16_H_12_BClNO_4_Si^−^ [M − H]^−^ 356.0323; found 356.0321.

#### 4.2.16 (8g) 7-Chloro-6-(2′,6′-dichloroisonicotinoyl)-1,1-dimethyl-3-hydroxybenzo-1,2,3-siloxaborole


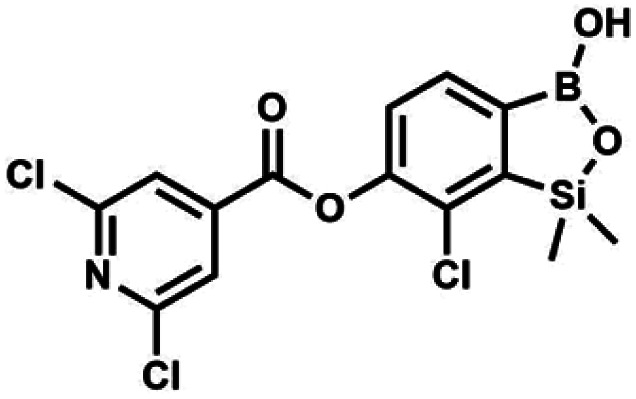
This compound was obtained using the protocol described for 6 using suspension of NaH in mineral oil (60%, 0.10 g, 2.50 mmol, 1.8 eq.), 5b (0.4 g, 1.4 mmol, 1.0 eq.) and 2,6-dichloroisonicotinoyl chloride (0.23 mL, 1.7 mmol, 1.2 eq.) as the starting materials. It was obtained as a white powder, mp 113–114 °C. Yield 0.22 g (39%). ^1^H NMR (400 MHz, acetone-*d*_6_) *δ* 7.89 (s, 2H, Py), 7.60 (d, *J* = 7.8 Hz, 1H, Ar^B^), 7.08 (d, *J* = 7.8 Hz, 1H, Ar^B^), 0.43 (s, 6H, SiMe_2_) ppm. ^13^C NMR (101 MHz, acetone-*d*_6_) *δ* 163.5, 155.2, 151.3, 144.3, 131.8, 123.3, 119.2, −1.8 ppm. HRMS (ESI, positive ion mode): calcd for C_14_H_12_BCl_3_NO_4_Si^+^ [MH]^+^ 401.9689; found 401.9689.

#### 4.2.17 (9a) 7-Chloro-6-phenylsulfonyloxy-1,1-dimethyl-3-hydroxybenzo-1,2,3-siloxaborole


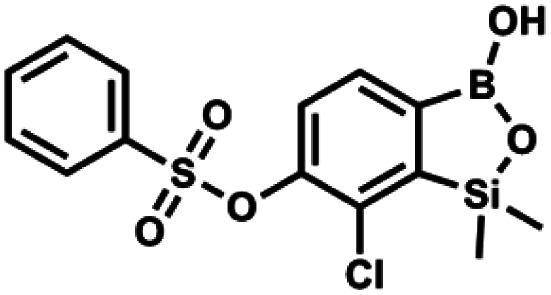
A suspension of NaH in mineral oil (60%, 0.10 g, 2.48 mmol, 2.5 eq.) under argon atmosphere was washed with anhydrous hexane (5 mL) twice and anhydrous DMF (10 mL) was added and stirred for 10 min. Then it was cooled to 0 °C and 5b (0.5 g, 1.7 mmol, 1.0 eq.) was added. After *ca.* 30 min stirring in 0 °C, benzenesulfonyl chloride (0.24 mL, 2.0 mmol, 1.2 eq.) was added to the white suspension. It was stirred for another 1.5 h at room temperature, then it was concentrated by a simple distillation under reduced pressure and was quenched with water (5 mL) and then with 1.5 M aq. H_2_SO_4_ to reach the pH = 2–3. Et_2_O (15 mL) and brine (10 mL) were added, then the aqueous phase was separated followed by the extraction with Et_2_O (2 × 10 mL). The extracts were added to the organic phase and dried under anhydrous MgSO_4_. Then it was concentrated under reduced pressure. Hexane (5 mL) and acetone (0.5 mL) were added to obtained oily residue and the mixture was stirred for 24 h. Then, precipitated white solid was filtered and washed with hexane (2 × 2 mL). The product was dried *in vacuo*, mp 150–152 °C. Yield 0.28 g (48%). ^1^H NMR (400 MHz, CDCl_3_) *δ* 7.93–7.89 (m, 2H, Ar^S^), 7.72–7.69 (m, 1H, Ar^S^), 7.66 (d, *J* = 7.8 Hz, 1H, ArB), 7.56–7.51 (m, 2H, Ar^S^), 7.36 (d, *J* = 7.8 Hz, 1H, ArB), 0.44 (s, 6H, SiMe_2_) ppm. ^13^C NMR (101 MHz, CDCl_3_) *δ* 152.7, 147.3, 135.8, 134.6, 131.2, 130.3, 129.8, 129.3, 128.8, 126.2, −1.4 ppm. ^11^B NMR (96 MHz, CDCl_3_) *δ* 30.6 ppm. HRMS (ESI, negative ion mode): calcd for C_14_H_14_BClO_5_SSi^−^ [M − H]^−^ 367.0040; found 367.0039.

#### 4.2.18 (9b) 7-Chloro-6-(2′-fluorophenylsulfonyloxy)-1,1-dimethyl-3-hydroxybenzo-1,2,3-siloxaborole


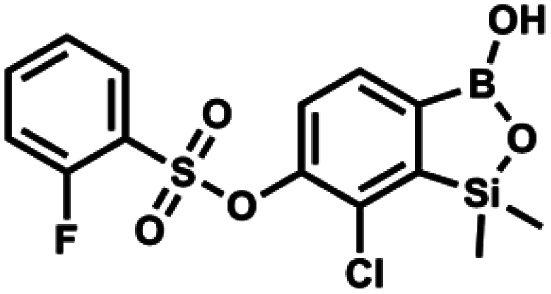
This compound was obtained using the protocol described for 9a using suspension of NaH in mineral oil (60%, 0.14 g, 3.5 mmol, 2.0 eq.), 5b (0.51 g, 1.8 mmol, 1.0 eq.) and 2-fluorobenzenesulfonyl chloride (0.23 mL, 1.7 mmol, 1.0 eq.) as the starting materials. It was obtained as a white powder, mp 158–161 °C. Yield 0.30 g (45%). ^1^H NMR (400 MHz, CDCl_3_) *δ* 7.92–7.82 (m, 1H, Ar^S^), 7.75–7.71 (m, 1H, Ar^S^), 7.67 (d, *J* = 7.9 Hz, 1H, Ar^B^), 7.36 (d, *J* = 7.9 Hz, 1H, Ar^S^), 7.30 (d, *J* = 7.9 Hz, 1H, Ar^B^), 7.28–7.26 (m, 1H, Ar^S^), 0.45 (s, 6H, SiMe_2_) ppm. ^13^C NMR (101 MHz, CDCl_3_) *δ* 159.7 (d, *J* = 261.3 Hz), 152.6, 147.1, 137.0 (d, *J* = 8.6 Hz), 131.4, 131.2 (d, *J* = 6.8 Hz), 129.9, 126.0, 124.4 (d, *J* = 4.1 Hz), 124.2 (d, *J* = 13.7 Hz), 117.6 (d, *J* = 20.9 Hz), 114.0, −1.6 ppm. ^19^F NMR (376 MHz, CDCl_3_) *δ* −100.52 (m) ppm. HRMS (ESI, negative ion mode): calcd C_14_H_13_BClFO_5_SSi^−^ for [M − H] ^−^ 384.9946; found 384.9949.

#### 4.2.19 (9c) 7-Chloro-6-(4′-fluorophenylsulfonyloxy)-1,1-dimethyl-3-hydroxybenzo-1,2,3-siloxaborole


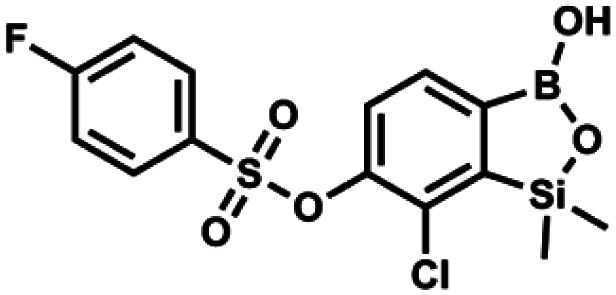
This compound was obtained using the protocol described for 9a using suspension of NaH in mineral oil (60%, 0.14 g, 3.5 mmol, 2.0 eq.), 5b (0.51 g, 1.8 mmol, 1.0 eq.) and 4-fluorobenzenesulfonyl chloride (0.36 g, 1.8 mmol, 1.0 eq.) as the starting materials. It was obtained as a white powder, mp 142–144 °C. Yield 0.40 g (42%). ^1^H NMR (400 MHz, CDCl_3_) *δ* 7.95–7.90 (m, 2H, Ar^S^), 7.69 (d, *J* = 7.9 Hz, 1H, Ar^B^), 7.40 (d, *J* = 7.9 Hz, 1H, Ar^B^), 7.24–7.18 (m, 2H, Ar^S^), 5.44 (s, 1H, OH), 0.45 (s, 6H, SiMe_2_) ppm. ^13^C NMR (101 MHz, CDCl_3_) *δ* 166.4 (d, *J* = 257.9 Hz), 152.7, 147.2, 132.1 (d, *J* = 1.6 Hz), 131.7 (d, *J* = 9.8 Hz), 131.7, 131.4, 130.1, 126.3, 116.7 (d, *J* = 22.9 Hz), −1.4 ppm. ^19^F NMR (376 MHz, CDCl_3_) *δ* −101.53 to −101.67 (m) ppm. HRMS (ESI, negative ion mode): calcd for C_14_H_13_BClFO_5_SSi^−^ [M − H]^−^ 384.9946; found 384.9949.

#### 4.2.20 (9d) 7-Chloro-6-(4′-chlorophenylsulfonyloxy)-1,1-dimethyl-3-hydroxybenzo-1,2,3-siloxaborole


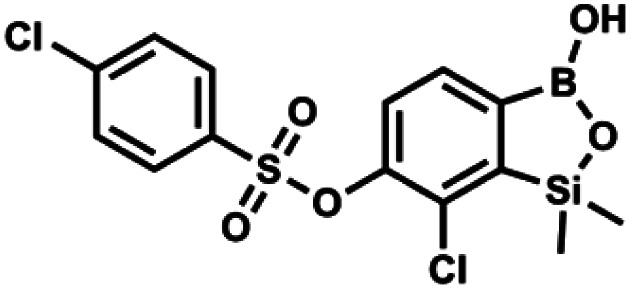
This compound was obtained using the protocol described for 9a using suspension of NaH in mineral oil (60%, 0.20 g, 4.96 mmol, 2.0 eq.), 5b (0.72 g, 2.48 mmol, 1.0 eq.) and 4-chlorobenzenesulfonyl chloride (0.52 g, 2.48 mmol, 1.0 eq.) as the starting materials. It was obtained as a white powder. mp 121–123 °C. Yield 0.46 g (46%). ^1^H NMR (400 MHz, CDCl_3_) *δ* 7.84 (d, *J* = 8.7 Hz, 2H, Ar^S^), 7.69 (d, *J* = 7.8 Hz, 1H, Ar^B^), 7.51 (d, *J* = 8.7 Hz, 2H, Ar^S^), 7.38 (d, *J* = 7.8 Hz, 1H, Ar^B^), 5.47 (s, 1H, OH), 0.45 (s, 6H, SiMe_2_) ppm. ^13^C NMR (101 MHz, CDCl_3_) *δ* 152.7, 147.0, 141.4, 134.0, 131.2, 130.1, 130.0, 129.5, 126.0, −1.6 ppm. HRMS (ESI): calcd for C_14_H_13_BCl_2_O_5_SSi [M − H]^−^ 400.9650; found 400.9652.

#### 4.2.21 (9e) 7-Chloro-6-(4′-bromophenylsulfonyloxy)-1,1-dimethyl-3-hydroxybenzo-1,2,3-siloxaborole


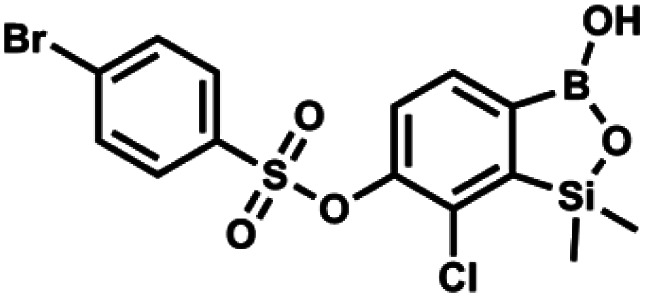
This compound was obtained using the protocol described for 9a using suspension of NaH in mineral oil (60%, 0.20 g, 4.96 mmol, 2.0 eq.), 5b (0.72 g, 2.48 mmol, 1.0 eq.) and 4-bromobenzenesulfonyl chloride (0.64 g, 2.48 mmol, 1.0 eq.) as the starting materials. It was obtained as a white powder, mp 108–111 °C. Yield 0.45 g (41%). ^1^H NMR (400 MHz, CDCl_3_) *δ* 7.74–7.72 (m, 2H, Ar^S^), 7.69–7.66 (m, 3H, Ar^S^ + Ar^B^), 7.38 (d, *J* = 7.8 Hz, 1H, Ar^B^), 0.45 (s, 6H, SiMe_2_) ppm. ^13^C NMR (101 MHz, CDCl_3_) *δ* 152.6, 147.0, 134.6, 132.5, 131.3, 130.1, 130.0, 127.8, 126.0, −1.6 ppm. HRMS (ESI): calcd for C_14_H_13_BBrClO_5_SSi [M − H]^−^ 444.9145; found 444.9147.

#### 4.2.22 (9f) 7-Chloro-6-(4′-iodophenylsulfonyloxy)-1,1-dimethyl-3-hydroxybenzo-1,2,3-siloxaborole


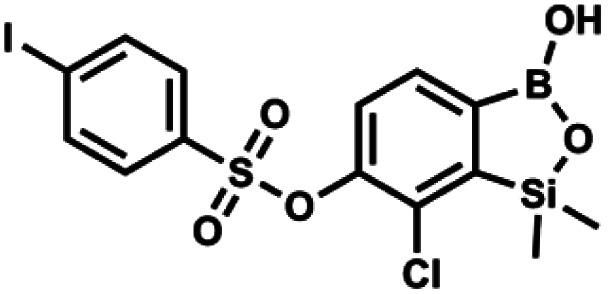
This compound was obtained using the protocol described for 9a using suspension of NaH in mineral oil (60%, 0.20 g, 4.96 mmol, 2.0 eq.), 5b (0.72 g, 2.48 mmol, 1.0 eq.) and 4-iodobenzenesulfonyl chloride (0.74 g, 2.48 mmol, 1.0 eq.) as the starting materials. It was obtained as a white powder, mp 112–116 °C. Yield 0.50 g (41%). ^1^H NMR (400 MHz, CDCl_3_) *δ* 7.90 (d, *J* = 8.7 Hz, 2H), 7.67 (d, *J* = 7.8 Hz, 1H), 7.59 (d, *J* = 8.7 Hz, 2H), 7.37 (d, *J* = 7.8 Hz, 1H), 0.44 (s, 6H, SiMe_2_) ppm. ^13^C NMR (101 MHz, CDCl_3_) *δ* 152.7, 147.0, 138.5, 135.2, 131.2, 130.0, 129.8, 126.0, 102.6, −1.6 ppm. HRMS (ESI): calcd for C_14_H_13_BClIO_5_SSi^−^ [M − H]^−^ 492.9006; found 492.9010.

#### 4.2.23 (9g) 7-Chloro-6-[2′-(trifluoromethyl)phenylsulfonyloxy]-1,1-dimethyl-3-hydroxybenzo-1,2,3-siloxaborole


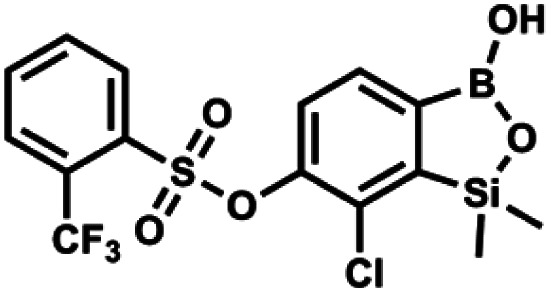
This compound was obtained using the protocol described for 9a using suspension of NaH in mineral oil (60%, 0.14 g, 3.5 mmol, 2.0 eq.), 5b (0.50 g, 1.8 mmol, 1.0 eq.) and 2-(trifluoromethyl)benzenesulfonyl chloride (0.26 mL, 1.7 mmol, 1.0 eq.) as the starting materials. It was obtained as a white powder, mp 160–162 °C. Yield 0.14 g (19%). ^1^H NMR (400 MHz, CDCl_3_) *δ* 8.13 (d, *J* = 7.3 Hz, 1H, Ar^S^), 8.00 (d, *J* = 7.2 Hz, 1H, Ar^S^), 7.84 (t, *J* = 7.7 Hz, 1H, Ar^S^), 7.72 (t, *J* = 7.2 Hz, 1H, Ar^S^), 7.66 (d, *J* = 7.9 Hz, 1H, Ar^B^), 7.29 (d, *J* = 7.9 Hz, 1H, Ar^B^), 5.26 (s, 1H, OH), 0.45 (s, 6H, SiMe_2_) ppm. ^13^C NMR (101 MHz, CDCl_3_) *δ* 152.8, 147.2, 134.7, 134.5, 132.4, 132.2, 131.1, 129.9, 129.5 (q, *J* = 34.1 Hz), 128.8 (q, *J* = 6.1 Hz), 127.7, 126.2, 122.3 (q, *J* = 273 Hz), −1.6 ppm. ^19^F NMR (376 MHz, CDCl_3_) *δ* −58.11 ppm. HRMS (ESI, negative ion mode): calcd for C_15_H_13_BClF_3_O_5_SSi^−^ [M − H]^−^ 434.9914; found 434.9915.

#### 4.2.24 (9h) 7-Chloro-6-[4′-(trifluoromethyl)phenylsulfonyloxy]-1,1-dimethyl-3-hydroxybenzo-1,2,3-siloxaborole


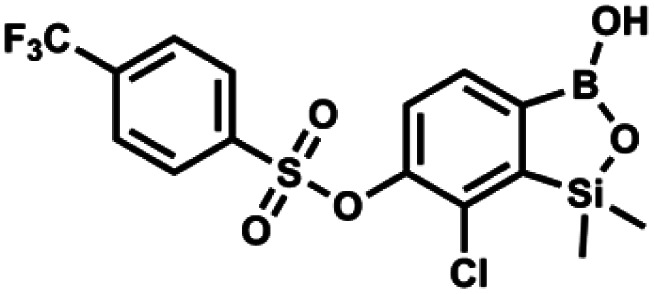
This compound was obtained using the protocol described for 9a using suspension of NaH in mineral oil (60%, 0.14 g, 3.5 mmol, 2.0 eq.), 5b (0.51 g, 1.8 mmol, 1.0 eq.) and 4-(trifluoromethyl)benzenesulfonyl chloride (0.43 g, 1.8 mmol, 1.0 eq.) as the starting materials. It was obtained as a white powder. Yield 0.15 g (20%). ^1^H NMR (400 MHz, CDCl_3_) *δ* 8.04 (dt, *J* = 8.2, 0.7 Hz, 1H, Ar^S^), 7.80 (dt, *J* = 8.2, 0.7 Hz, 2H, Ar^S^), 7.70 (d, *J* = 7.8 Hz, 1H, Ar^B^), 7.42 (d, *J* = 7.9 Hz, 1H, Ar^B^), 5.18 (s, 1H, OH), 0.43 (s, 6H, SiMe_2_) ppm. ^13^C NMR (101 MHz, CDCl_3_) *δ* 152.9, 146.8, 139.1, 136.1 (q, *J* = 33.2 Hz), 131.3, 129.2, 126.2 (q, *J* = 3.8 Hz), 126.0, 125.7 (q, *J* = 271.7 Hz), −1.7 ppm. ^19^F NMR (376 MHz, CDCl_3_) *δ* −63.3 ppm. HRMS (ESI, negative ion mode): calcd for C_15_H_13_BClF_3_O_5_SSi^−^ [M − H]^−^ 434.9914; found 434.9913.

#### 4.2.25 (9i) 7-Chloro-6-(4′-methylphenylsulfonyloxy)-1,1-dimethyl-3-hydroxybenzo-1,2,3-siloxaborole


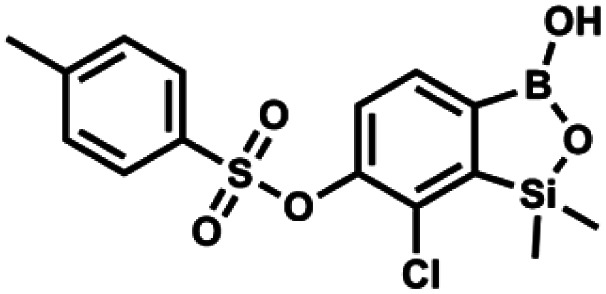
A suspension of NaH in mineral oil (60%, 0.20 g, 4.96 mmol, 2.0 eq.) under argon atmosphere was washed with anhydrous hexane (5 mL) twice and anhydrous DMF (10 mL) was added and stirred for 10 min. Then it was cooled to 0 °C and 5b (0.72 g, 2.48 mmol, 1.0 eq.) was added. After *ca.* 30 min stirring in 0 °C, tosyl chloride (0.47 g, 2.48 mmol, 1.0 eq.) was added to the white suspension. It was stirred for another 1.5 h at room temperature, then it was concentrated by a simple distillation under reduced pressure and was quenched with water (5 mL) and then with 1.5 M aq. H_2_SO_4_ to reach the pH = 2–3. Et_2_O (15 mL) and brine (10 mL) were added, then the aqueous phase was separated followed by the extraction with Et_2_O (2 × 10 mL). The extracts were added to the organic phase and dried under anhydrous MgSO_4_. Then it was concentrated under reduced pressure. 5% NaHCO_3_/H_2_O (10 mL) was added to obtained oily residue and was stirred for 15 min pending the precipitation of white solid. It was filtered, washed with 1.5 M H_2_SO_4_ (10 mL) and washed several times with water. The product was dried *in vacuo*, mp 109–122 °C. Yield 0.98 g (96%). ^1^H NMR (400 MHz, CDCl_3_) *δ* 7.78 (d, *J* = 8.4 Hz, 1H, Ar^S^), 7.64 (d, *J* = 7.8 Hz, 1H, Ar^B^), 7.35–7.30 (m, 3H, Ar^S^ + Ar^B^), 5.18 (s, 1H, OH), 2.45 (s, 3H, Me), 0.43 (s, 6H, SiMe_2_) ppm. ^13^C NMR (101 MHz, CDCl_3_) *δ* 152.6, 147.2, 145.7, 132.6, 131.0, 130.2, 130.0, 129.7, 128.7, 128.5, 125.9, 21.7, −1.6 ppm. HRMS (ESI, negative ion mode): calcd for C_15_H_16_BClO_5_SSi^−^ [M − H]^−^ 381.0197; found 381.0200.

#### 4.2.26 (9j) 7-Chloro-6-(3′-methylphenylsulfonyloxy)-1,1-dimethyl-3-hydroxybenzo-1,2,3-siloxaborole


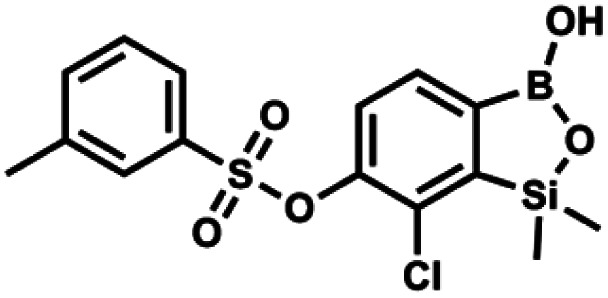
This compound was obtained using the protocol described for 9i using suspension of NaH in mineral oil (60%, 0.20 g, 4.96 mmol, 2.0 eq.), 5b (0.72 g, 2.48 mmol, 1.0 eq.) and 3-methylbenzenesulfonyl chloride (0.47 g, 2.48 mmol, 1.0 eq.) as the starting materials. It was obtained as a white powder, mp 116–120 °C. Yield 0.60 g (63%). ^1^H NMR (400 MHz, CDCl_3_) *δ* 7.76–7.62 (m, 3H, Ar^S^ + Ar^B^), 7.55–7.46 (m, 1H, Ar^S^), 7.41 (t, *J* = 7.7 Hz, 1H, Ar^S^), 7.34 (d, *J* = 7.8 Hz, 1H, Ar^B^), 2.42 (s, 3H, Me), 0.45 (s, 6H, SiMe_2_). ^13^C NMR (101 MHz, CDCl_3_) *δ* 152.7, 147.4, 139.7, 135.6, 135.4, 131.2, 130.3, 129.1, 129.0, 126.2, 125.9, 21.4, −1.4 ppm. HRMS (ESI, negative ion mode): calcd for C_15_H_16_BClO_5_SSi^−^ [M − H]^−^ 381.0197; found 381.0198.

#### 4.2.27 (9k) 7-Chloro-6-[2′,4′,4′-trimethylphenylsulfonyloxy]-1,1-dimethyl-3-hydroxybenzo-1,2,3-siloxaborole


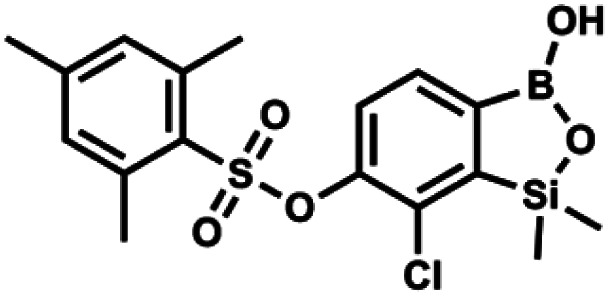
This compound was obtained using the protocol described for 9i using suspension of NaH in mineral oil (60%, 0.20 g, 4.96 mmol, 2.0 eq.), 5b (0.72 g, 2.48 mmol, 1.0 eq.) and 2,4,6-trimethylbenzenesulfonyl chloride (0.54 g, 2.48 mmol, 1.0 eq.) as the starting materials. It was obtained as a white powder, mp 162–169 °C. Yield 0.68 g (67%). ^1^H NMR (400 MHz, CDCl_3_) *δ* 7.59 (d, *J* = 7.8 Hz, 1H, Ar^B^), 7.12 (d, *J* = 7.8 Hz, 1H, Ar^B^), 7.00 (s, 2H, Ar^S^), 2.61 (s, 6H, *o*-Me), 2.34 (s, 3H, *p*-Me), 0.47 (s, 6H, SiMe_2_) ppm. ^13^C NMR (101 MHz, CDCl_3_) *δ* 152.6, 147.4, 144.1, 140.5, 131.8, 131.4, 130.9, 130.3, 125.5, 22.3, 21.1, −1.5 ppm. HRMS (ESI, negative ion mode): calcd for C_17_H_20_BClO_5_SSi^−^ [M − H]^−^ 409.0510; found 409.0513.

#### 4.2.28 (9l) 7-Chloro-6-[4′-(*tert*-butyl)phenylsulfonyloxy]-1,1-dimethyl-3-hydroxybenzo-1,2,3-siloxaborole


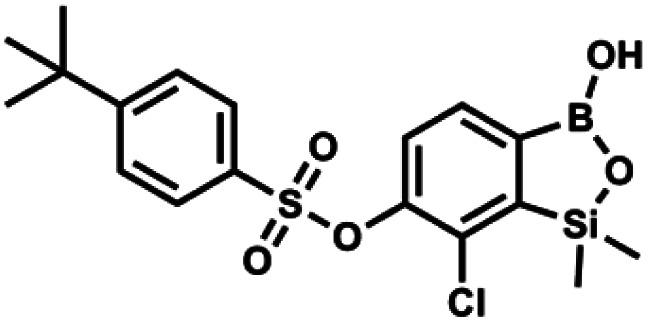
This compound was obtained using the protocol described for 9a using suspension of NaH in mineral oil (60%, 0.20 g, 4.96 mmol, 2.0 eq.), 5b (0.72 g, 2.48 mmol, 1.0 eq.) and 4-*tert*-butylbenzenesulfonyl chloride (0.58 g, 2.48 mmol, 1.0 eq.) as the starting materials. It was obtained as a white powder, mp 122–124 °C. Yield 0.75 g (71%). ^1^H NMR (400 MHz, CDCl_3_) *δ* 7.81–7.77 (m, 2H, Ar^S^), 7.67 (d, *J* = 7.8 Hz, 1H, Ar^B^), 7.53–7.50 (m, 2H, Ar^S^), 7.40 (d, *J* = 7.8 Hz, 1H, Ar^B^), 1.34 (s, 9H, *t*-Bu), 0.43 (s, 6H, SiMe_2_) ppm. ^13^C NMR (101 MHz, CDCl_3_) *δ* 158.8, 152.5, 147.2, 132.3, 131.1, 130.3, 128.6, 126.2, 126.1, 35.4, 31.0, −1.6 ppm. HRMS (ESI, negative ion mode): calcd for C_18_H_22_BClO_5_SSi^−^ [M − H]^−^ 423.0666; found 423.0670.

#### 4.2.29 (9m) 7-Chloro-6-(4′-acetylphenylsulfonyloxy)-1,1-dimethyl-3-hydroxybenzo-1,2,3-siloxaborole


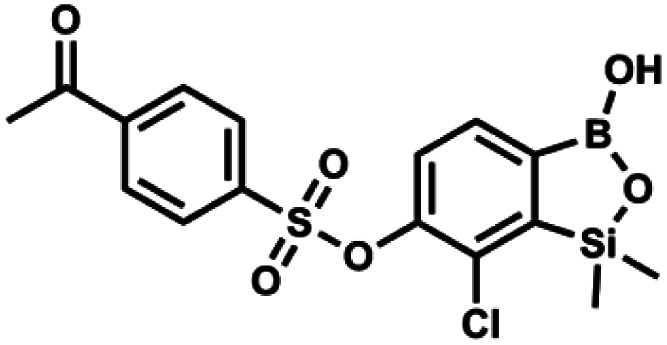
This compound was obtained using the protocol described for 9a using suspension of NaH in mineral oil (60%, 0.20 g, 4.96 mmol, 2.0 eq.), 5b (0.72 g, 2.48 mmol, 1.0 eq.) and 4-acetylbenzenesulfonyl chloride (0.53 g, 2.48 mmol, 1.0 eq.) as the starting materials. It was obtained as a white powder, mp 113–115 °C. Yield 0.63 g (62%). ^1^H NMR (400 MHz, CDCl_3_) *δ* 8.09 (d, *J* = 8.2 Hz, 2H, Ar^S^), 8.01 (d, *J* = 8.1 Hz, 2H, Ar^S^), 7.68 (d, *J* = 7.8 Hz, 1H, Ar^B^), 7.37 (d, *J* = 7.9 Hz, 1H, Ar^B^), 2.67 (s, 3H, MeCO), 0.43 (s, 6H, SiMe_2_) ppm. ^13^C NMR (101 MHz, CDCl_3_) *δ* 196.6, 152.8, 147.0, 141.3, 139.4, 131.2, 129.9, 129.0, 129.0, 126.0, 26.9, −1.6 ppm. HRMS (ESI, negative ion mode): calcd for C_16_H_16_BClO_6_SSi^−^ [M − H]^−^ 409.0146; found 409.0146.

#### 4.2.30 (9n) 7-Chloro-6-(4′-methoxyphenylsulfonyloxy)-1,1-dimethyl-3-hydroxybenzo-1,2,3-siloxaborole


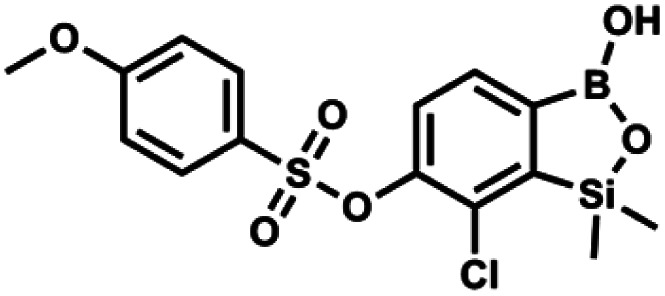
This compound was obtained using the protocol described for 9a using suspension of NaH in mineral oil (60%, 0.20 g, 4.96 mmol, 2.0 eq.), 5b (0.72 g, 2.48 mmol, 1.0 eq.) and 4-methoxybenzenesulfonyl chloride (0.53 g, 2.48 mmol, 1.0 eq.) as the starting materials. It was obtained as a white powder, mp 120–124 °C. Yield 0.62 g (63%). ^1^H NMR (400 MHz, CDCl_3_) *δ* 7.84–7.79 (m, 2H, Ar^S^), 7.65 (d, *J* = 7.8 Hz, 1H, Ar^S^), 7.36 (d, *J* = 7.9 Hz, 1H, Ar^B^), 7.00–6.95 (m, 2H, Ar^S^), 3.89 (s, 3H, OMe), 0.44 (s, 6H, SiMe_2_). ^13^C NMR (101 MHz, CDCl_3_) *δ* 164.4, 152.6, 147.3, 131.0, 131.0, 127.0, 126.1, 55.8, −1.6 ppm. HRMS (ESI, negative ion mode): calcd for C_15_H_16_BClO_6_SSi [M − H]^−^ 397.0146; found 397.0146.

#### 4.2.31 (9o) 7-Chloro-6-(4′-nitrophenylsulfonyloxy)-1,1-dimethyl-3-hydroxybenzo-1,2,3-siloxaborole


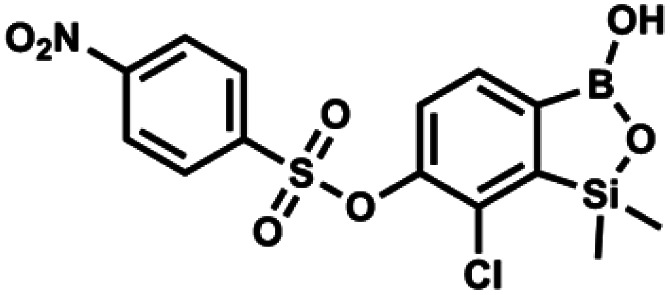
This compound was obtained using the protocol described for 9a using suspension of NaH in mineral oil (60%, 0.20 g, 4.96 mmol, 2.0 eq.), 5b (0.72 g, 2.48 mmol, 1.0 eq.) and 4-nitrobenzenesulfonyl chloride (0.55 g, 2.48 mmol, 1.0 eq.) as the starting materials. It was obtained as a white powder, mp 146–148 °C. Yield 0.39 g (38%). ^1^H NMR (400 MHz, CDCl_3_) *δ* 8.39 (d, *J* = 8.8 Hz, 2H, Ar^S^), 8.12 (d, *J* = 8.9 Hz, 2H, Ar^S^), 7.72 (d, *J* = 7.8 Hz, 1H, Ar^B^), 7.42 (d, *J* = 7.9 Hz, 1H, Ar^B^), 5.68 (s, 1H, OH), 0.44 (s, 6H, SiMe_2_) ppm. ^13^C NMR (101 MHz, CDCl_3_) *δ* 152.9, 151.2, 146.7, 141.3, 131.5, 130.0, 129.6, 126.0, 124.3, 118.6, −1.6 ppm. HRMS (ESI, negative ion mode): calcd for C_14_H_13_BClNO_7_SSi [M − H]^−^ 411.9891; found 411.9894.

#### 4.2.32 (9p) 7-Chloro-6-(4′-chloro-3′-nitrophenylsulfonyloxy)-1,1-dimethyl-3-hydroxybenzo-1,2,3-siloxaborole


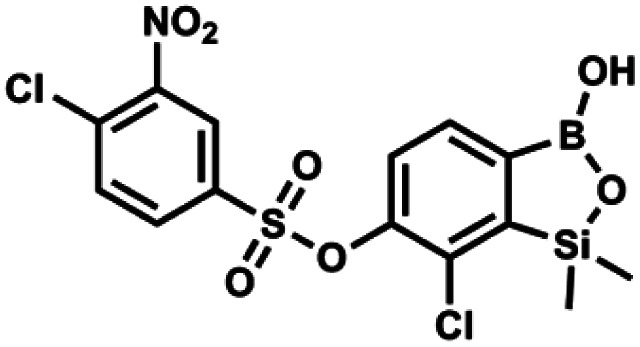
This compound was obtained using the protocol described for 9a using suspension of NaH in mineral oil (60%, 0.20 g, 4.96 mmol, 2.0 eq.), 5b (0.72 g, 2.48 mmol, 1.0 eq.) and 4-chloro-3-nitrobenzenesulfonyl chloride (0.63 g, 2.48 mmol, 1.0 eq.) as the starting materials. It was obtained as a white powder, mp 96–98 °C. Yield 0.55 g (50%). ^1^H NMR (400 MHz, DMSO-*d*_6_) *δ* 8.57 (dd, *J* = 2.0, 0.6 Hz, 1H, Ar^S^), 8.11–8.05 (m, 2H, Ar^S^), 7.21 (d, *J* = 7.7 Hz, 1H, Ar^B^), 7.00 (d, *J* = 7.6 Hz, 1H, Ar^B^), 0.13 (s, 6H, SiMe_2_) ppm. ^13^C NMR (101 MHz, DMSO-*d*_6_) *δ* 148.6, 148.2, 142.5, 135.3, 133.9, 133.2, 132.4, 128.9, 127.7, 126.0, 124.0, 1.0 ppm. HRMS (ESI, negative ion mode): calcd for C_14_H_12_BCl_2_NO_7_SSi [M − H]^−^ 445.9501; found 445.9502.

#### 4.2.33 (9q) 7-Chloro-6-(3′,4′-dichlorophenylsulfonyloxy)-1,1-dimethyl-3-hydroxybenzo-1,2,3-siloxaborole


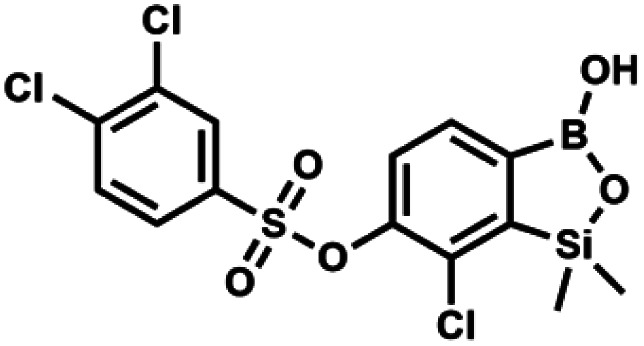
This compound was obtained using the protocol described for 9i using suspension of NaH in mineral oil (60%, 0.20 g, 4.96 mmol, 2.0 eq.), 5b (0.72 g, 2.48 mmol, 1.0 eq.) and 3,4-dichlorobenzenesulfonyl chloride (0.61 g, 2.48 mmol, 1.0 eq.) as the starting materials. It was obtained as a white powder, mp 95–100 °C. Yield 0.58 g (53%). ^1^H NMR (400 MHz, CDCl_3_) *δ* 7.96 (d, *J* = 2.2 Hz, 1H, Ar^S^), 7.75–7.69 (m, 2H, Ar^S^ + Ar^B^), 7.62 (d, *J* = 8.5 Hz, 1H, Ar^S^), 7.40 (d, *J* = 7.8 Hz, 1H, Ar^B^), 5.58 (s, 1H, OH), 0.46 (s, 6H, SiMe_2_). ^13^C NMR (101 MHz, CDCl_3_) *δ* 152.8, 146.8, 139.7, 135.2, 134.0, 131.4, 131.2, 130.4, 129.8, 127.5, 126.1, 121.8, −1.6 ppm. HRMS (ESI, negative ion mode): calcd for C_14_H_12_BCl_3_O_5_SSi [M − H]^−^ 434.9261; found 434.9261.

#### 4.2.34 (9r) 7-Chloro-6-[4′-chloro-3′-(trifluoromethyl)phenylsulfonyloxy]-1,1-dimethyl-3-hydroxybenzo-1,2,3-siloxaborole


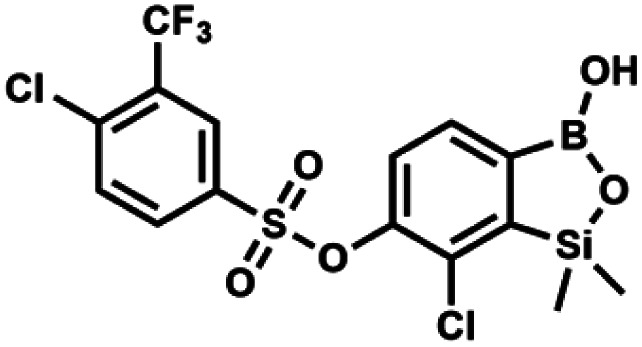
This compound was obtained using the protocol described for 9i using suspension of NaH in mineral oil (60%, 0.20 g, 4.96 mmol, 2.0 eq.), 5b (0.72 g, 2.48 mmol, 1.0 eq.) and 4-chloro-3-(trifluoromethyl)-benzenesulfonyl chloride (0.53 g, 2.48 mmol, 1.0 eq.) as the starting materials. It was obtained as a white powder, mp 124–126 °C. Yield 0.85 g (73%). ^1^H NMR (400 MHz, CDCl_3_) *δ* 8.18 (d, *J* = 2.1 Hz, 1H, Ar^S^), 8.04–8.00 (m, 1H, Ar^S^), 7.74–7.68 (m, 2H, Ar^S^ + Ar^B^), 7.45 (d, *J* = 7.9 Hz, 1H, Ar^B^), 5.09 (s, 1H, OH), 0.44 (s, 6H, SiMe_2_) ppm. ^13^C NMR (101 MHz, CDCl_3_) *δ* 153.0, 146.7, 139.4, 134.7, 132.7, 132.6 (q, *J* = 14.6 Hz), 129.8 (q, *J* = 32.8 Hz), 129.5, 128.00 (q, *J* = 5.4 Hz), 124.6 (q, *J* = 274.3 Hz), −1.7 ppm. ^19^F NMR (376 MHz, chloroform-*d*) *δ* −63.22 ppm. HRMS (ESI, negative ion mode): calcd for C_15_H_12_BCl_2_F_3_O_5_SSi [M − H]^−^ 468.9524; found 468.9525.

#### 4.2.35 (11a) 2-(4-Bromo-2-fluorophenoxy)-3-chloropyridine


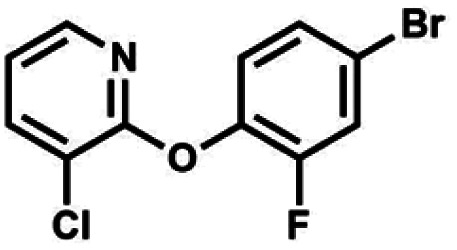
This compound was obtained using the protocol described below for 11b using 4-bromo-2-fluorophenol (12 ml, 110 mmol, 1.1 eq.), NaOH (4.40 g, 110 mmol, 1.1 eq.) and 2,3-dichloropyridine (14.80 g, 100 mmol, 1.0 eq.) as the starting materials. However, the temperature was maintained at 150 °C during the reaction. The product was obtained as a yellow solid. mp 71–73 °C. Yield 16.6 g (55%). ^1^H NMR (400 MHz, CDCl_3_) *δ* 7.98 (dd, *J* = 4.9, 1.7 Hz, 1H, Py), 7.77 (dd, *J* = 7.7, 1.7 Hz, 1H, Py), 7.37 (dd, *J* = 9.6, 2.3 Hz, 1H, Ar), 7.33 (ddd, *J* = 8.6, 2.3, 1.3 Hz, 1H, Ar), 7.15 (dd, *J* = 8.5, 8.1 Hz, 1H, Ar), 6.99 (dd, *J* = 7.7, 4.9 Hz, 1H, Py) ppm. ^13^C NMR (101 MHz, CDCl_3_) *δ* 158.0, 154.7 (d, *J* = 254.1 Hz), 145.1, 140.1 (d, *J* = 12.2 Hz), 139.6, 128.0 (d, *J* = 3.8 Hz), 125.4 (d, *J* = 1.6 Hz), 120.6 (d, *J* = 21.4 Hz), 119.9, 118.47 (d, *J* = 2.3 Hz), 118.4 ppm. ^19^F NMR (376 MHz, CDCl_3_) *δ* −123.69 to −124.40 (m) ppm. Anal. calcd for C_11_H_6_BrClFNO (302.53): C 43.67, H 2.00, N 4.63; found C 43.57, H 1.93, N 4.60.

#### 4.2.36 (11b) 2-(4-Bromo-2-fluorophenoxy)-6-chloropyridine


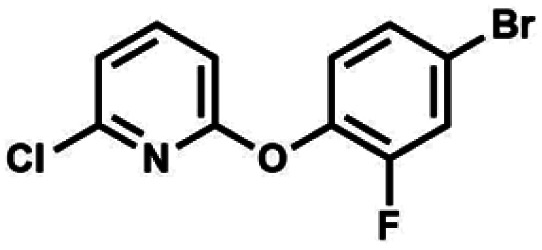
NaOH (4.40 g, 110 mmol, 1.1 eq.) was dissolved in DMSO (100 mL) in 80 °C and obtained solution was cooled to 50 °C. 4-Bromo-2-fluorophenol (12 ml, 110 mmol, 1.1 eq.) and 2,6-dichloropyridine (14.8 g, 100 mmol, 1.0 eq.) was added to the stirred solution of NaOH in DMSO in 50 °C. It was heated in 110 °C for 24 h, then it was cooled to the room temperature and AcOEt (20 mL) was added and stirred for *ca.* 20 min. It was filtered and concentrated to dryness under reduced pressure. Obtained solid was washed with water (100 mL) and it was filtered under reduce pressure. Then Et_2_O (70 mL) was added and obtained suspension was filtered under reduce pressure. The filtrate was concentrated to dryness and obtained solid residue was crystallized in heptane. The light brown solid was filtered and washed several times with cold heptane. The product was dried *in vacuo*, mp 69–72 °C. Yield 20.1 g (66%). ^1^H NMR (400 MHz, CDCl_3_) *δ* 7.65 (dd, *J* = 8.1, 7.6 Hz, 1H, Py), 7.36 (dd, *J* = 9.7, 2.3 Hz, 1H, Ar), 7.30 (ddd, *J* = 8.6, 2.3, 1.4 Hz, 1H, Ar), 7.14–7.10 (m, 1H, Ar), 7.05 (dd, *J* = 7.6, 0.6 Hz, 1H, Py), 6.89 (dd, *J* = 8.1, 0.7 Hz, 1H, Py) ppm. ^13^C NMR (101 MHz, CDCl_3_) *δ* 161.89, 154.65 (d, *J* = 254.0 Hz), 148.9, 141.7, 140.0 (d, *J* = 11.9 Hz), 128.0 (d, *J* = 3.8 Hz), 125.1 (d, *J* = 1.8 Hz), 120.6 (d, *J* = 21.4 Hz), 119.1, 118.1 (d, *J* = 8.3 Hz), 109.0 ppm. ^19^F NMR (376 MHz, CDCl_3_) *δ* −124.50 to −124.76 (m) ppm. Anal. calcd for C_11_H_6_BrClFNO (302.53): C 43.67, H 2.00, N 4.63; found C 43.53, H 1.92, N 4.58.

#### 4.2.37 (12a) 2-[4-Bromo-3-(dimethylsilyl)-2-fluorophenoxy]-3-chloropyridine


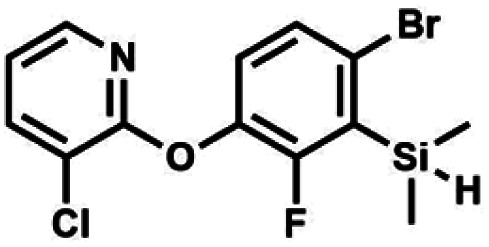
This compound was obtained using the protocol described for 3b using 11a (1.01 g, 3.3 mmol, 1.0 eq.), *n*-BuLi (1.6 M, 2.3 mL, 3.7 mmol, 1.1 eq.), *i*Pr_2_NH (0.6 mL, 4.3 mmol, 1.3 eq.) and Me_2_SiHCl (0.5 mL, 4.5 mmol, 1.4 eq.) as the starting materials. It was obtained as a yellow oil. Yield 0.87 g (73%). ^1^H NMR (300 MHz, CDCl_3_) *δ* 8.02 (dd, *J* = 4.9, 1.7 Hz, 1H, Py), 7.79 (dd, *J* = 7.7, 1.7 Hz, 1H, Py), 7.43 (dd, *J* = 8.5, 1.4 Hz, 1H, Ar), 7.16 (t, *J* = 8.5 Hz, 1H, Ar), 7.02 (dd, *J* = 7.7, 4.9 Hz, 1H, Py), 4.79–4.74 (m, 1H, SiH), 0.48 (dd, *J* = 3.9, 1.8 Hz, 6H, SiMe_2_) ppm. ^13^C NMR (101 MHz, CDCl_3_) *δ* 158.6 (d, *J* = 248.4 Hz), 158.0, 145.0, 139.7 (d, *J* = 16.7 Hz), 139.4, 129.4 (d, *J* = 3.5 Hz), 127.4 (d, *J* = 29.4 Hz), 126.3 (d, *J* = 2.2 Hz), 125.8 (d, *J* = 10.8 Hz), 119.7, −3.2 (d, *J* = 4.3 Hz) ppm. ^19^F NMR (282 MHz, CDCl_3_) *δ* −110.72 to −110.80 (m) ppm. Anal. calcd for C_13_H_12_BrClFNOSi (360.68): C 43.29, H 3.35, N 3.88; found C 43.13, H 3.28, N 3.87.

#### 4.2.38 (12b) 2-[4-Bromo-3-(dimethylsilyl)-2-fluorophenoxy]-6-chloropyridine


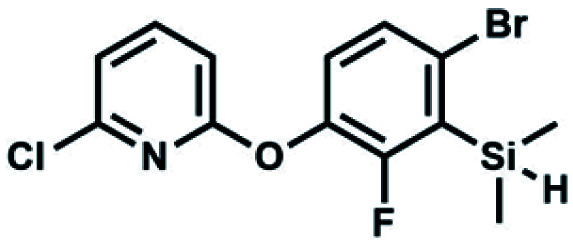
This compound was obtained using the protocol described for 3b using 11b (3.03 g, 10.0 mmol, 1.0 eq.), *n*-BuLi (8 M, 1.1 mL, 9.0 mmol, 0.9 eq.), *i*Pr_2_NH (1.7 mL, 12.0 mmol, 1.2 eq.) and Me_2_SiHCl (1.4 mL, 13.0 mmol, 1.3 eq.) as the starting materials. It was obtained as a yellow oil. Yield 3.53 g (98%). ^1^H NMR (400 MHz, CDCl_3_) *δ* 7.65 (dd, *J* = 8.4, 7.6 Hz, 1H, Py), 7.37 (dd, *J* = 8.6, 1.4 Hz, 1H, Ar), 7.11 (t, *J* = 8.5 Hz, 1H, Ar), 7.04 (dd, *J* = 7.6, 0.7 Hz, 1H, Py), 6.86 (dd, *J* = 8.1, 0.7 Hz, 1H, Py), 4.79–4.71 (m, 1H, SiH), 0.46 (dd, *J* = 4.0, 1.8 Hz, 6H, SiMe_2_) ppm. ^13^C NMR (101 MHz, CDCl_3_) *δ* 161.8, 158.5 (d, *J* = 248.0 Hz), 148.8, 141.5, 139.5 (d, *J* = 16.4 Hz), 129.3 (d, *J* = 3.8 Hz), 127.4 (d, *J* = 29.6 Hz), 125.9 (d, *J* = 2.3 Hz), 125.5 (d, *J* = 11.0 Hz), 120.5 (d, *J* = 21.4 Hz), 118.8, −3.3 (d, *J* = 4.2 Hz) ppm. ^19^F NMR (376 MHz, CDCl_3_) *δ* −109.94 to −111.02 (m) ppm. Anal. calcd for C_13_H_12_BrClFNOSi (360.68): C 43.29, H 3.35, N 3.88; found C 43.22, H 3.22, N 3.85.

#### 4.2.39 (13a) 7-Fluoro-6-(3-chloropyridin-2-oxy)-1,1-dimethyl-3-hydroxybenzo-1,2,3-siloxaborole


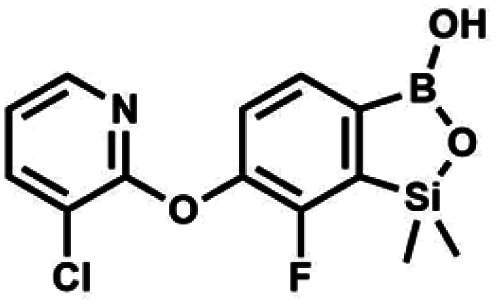
This compound was obtained using the protocol described for 4b using 12a (0.87 g, 2.4 mmol, 1.0 eq.), *t*-BuLi (1.9 M, 3.0 mL, 5.7 mmol, 2.4 eq.) and B(O*i*Pr)_3_ (2.9 mL, 13.0 mmol, 5.4 eq.) as the starting materials. However, the temperature was maintained below −90 °C during the reactions. It was obtained as a yellow powder, mp 134–141 °C. Yield 0.18 g (23%). ^1^H NMR (400 MHz, CDCl_3_) *δ* 8.01 (dd, *J* = 4.8, 1.7 Hz, 1H, Py), 7.78 (dd, *J* = 7.6, 1.7 Hz, 1H, Py), 7.63 (d, *J* = 7.7 Hz, 1H, Ar^B^), 7.36 (t, *J* = 7.5 Hz, 1H, Ar^B^), 6.99 (dd, *J* = 7.7, 4.9 Hz, 1H, Py), 0.50 (s, 6H, SiMe_2_) ppm. ^13^C NMR (101 MHz, CDCl_3_) *δ* 158.5, 155.9 (d, *J* = 248.5 Hz), 145.3, 142.7 (d, *J* = 14.9 Hz), 139.5, 137.2 (d, *J* = 28.2 Hz), 128.7 (d, *J* = 3.4 Hz), 126.7, 119.8, 118.7, −0.5. ^19^F NMR (376 MHz, CDCl_3_) *δ* −119.78 (d, *J* = 7.4 Hz) ppm. HRMS (ESI, negative ion mode): calcd for C_13_H_11_BClFNO_3_Si^−^ [M − H]^−^ 322.0279; found 322.0282.

#### 4.2.40 (13b) 7-Fluoro-6-(6-chloropyridin-2-oxy)-1,1-dimethyl-3-hydroxybenzo-1,2,3-siloxaborole


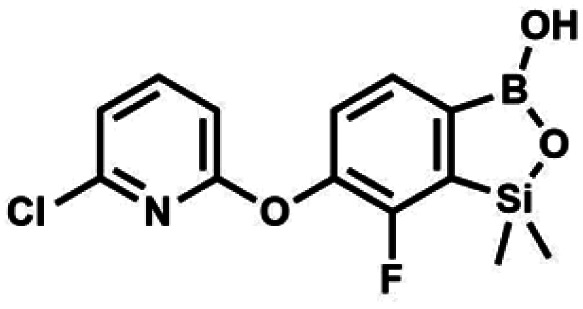
This compound was obtained using the protocol described for 4b using 12b (3.53 g, 9.8 mmol, 1.0 eq.), *t*-BuLi (1.9 M, 11 mL, 21.0 mmol, 2.1 eq.) and B(O*i*Pr)_3_ (3.4 mL, 15.0 mmol, 1.5 eq.) as the starting materials. It was obtained as a pale brown solid, mp 94–104 °C. Yield 0.41 g (20%). ^1^H NMR (400 MHz, DMSO-*d*_6_) *δ* 9.35 (s, 1H, OH), 7.94 (t, *J* = 8.0 Hz, 1H, Py), 7.70 (d, *J* = 7.7 Hz, 1H, Ar^B^), 7.43 (t, *J* = 7.6 Hz, 1H, Ar^B^), 7.28 (dd, *J* = 7.7, 0.6 Hz, 1H, Py), 7.14 (dd, *J* = 8.1, 0.6 Hz, 1H, Py), 0.43 (s, 6H, SiMe_2_) ppm. ^13^C NMR (101 MHz, DMSO-*d*_6_) *δ* 162.1, 155.5 (d, *J* = 246.2 Hz), 147.8, 143.80, 141.9 (d, *J* = 14.9 Hz), 137.0 (d, *J* = 28.3 Hz), 129.5 (d, *J* = 3.2 Hz), 126.8, 119.7, 110.1, −0.2 ppm. ^19^F NMR (376 MHz, DMSO-*d*_6_) *δ* −120.86 (d, *J* = 7.6 Hz) ppm. HRMS (ESI, negative ion mode): calcd for C_13_H_11_BClFNO_3_Si^−^ [M − H]^−^ 322.0279; found 322.0281.

### 4.3. Antimicrobial activity

#### 4.3.1 Bacterial and fungal strains and their growth conditions

The following standard strains to determine the direct antimicrobial activity were used in the study: (1) Gram-positive cocci: *Staphylococcus aureus* ATCC 6538P MSSA, *S. aureus* subsp. *aureus* ATCC 43300 MRSA, *S. epidermidis* ATCC 12228, *Enterococcus faecalis* ATCC 29212, *E. faecium* ATCC 6057, *Bacillus subtilis* ATCC 6633; (2) Gram-negative bacteria from *Enterobacteriales* order: *Escherichia coli* ATCC 25922, *Klebsiella pneumoniae* ATCC 13883, *Proteus mirabilis* ATCC 12453, *Enterobacter cloacae* DSM 6234, *Serratia marcescens* ATCC 13880; (3) Gram-negative non-fermentative rods: *Pseudomonas aeruginosa* ATCC 27853, *Acinetobacter baumannii* ATCC 19606, *Stenotrophomonas maltophilia* ATCC 12714, *S. maltophilia* ATCC 13637, *Burkholderia cepacia* ATCC 25416, *Bordetella bronchiseptica* ATCC 4617; (4) yeasts: *Candida albicans* ATCC 90028, *C. parapsilosis* ATCC 22019, *C. tropicalis* IBA 171, *C. tropicalis* (Castellani) Berkhout ATCC 750, *C. guilliermondii* IBA 155, *C. krusei* ATCC 6258 and *Saccharomyces cerevisiae* ATCC 9763.

All strains were stored at −80 °C. Prior to testing, each bacterial strain was subcultured twice on tryptic soy agar TSA (bioMerieux) medium and yeast strains on Sabouraud dextrose agar (bioMerieux) for 24–48 h at 30 °C to ensure viability.

#### 4.3.2 Determination of antimicrobial activity

Direct antimicrobial activity against yeast, Gram-positive and Gram-negative bacterial strains was examined by the disc-diffusion test and the MIC determination assays according to the EUCAST^49,50^ and CLSI^51–53^ recommendations. Additionally, in the study of antimicrobial activity of new benzosiloxaboroles three following reference agents were used: fluconazole (in the case of fungi), linezolid (for Gram-positive bacteria) and nitrofurantoin (for Gram-negative rods). The MIC value of fluconazole was tested by Etest method.^54,55^ Determination of MIC/MBC values of linezolid and nitrofurantoin was done using the CLSI methods,^38,53^ however, its concentration range was compatible with Etest. The solutions of all tested benzosiloxaboroles were prepared in DMSO (Sigma). The disc-diffusion test was determined on Mueller–Hinton II agar medium (MHA) (Becton Dickinson) for bacteria and on MHA supplemented with 2% glucose and 0.5 mg L^−1^ methylene blue dye (Sigma) (MHA + GMB medium) for yeasts. The MIC determination was performed in Mueller–Hinton II broth medium (MHB) (Becton Dickinson) for bacteria and in RPMI 1640 broth medium (Sigma) with 2% glucose (Sigma) for yeasts. Results of antimicrobial activity were evaluated after incubation at 35 °C for 18 h (bacteria) and 24 h (yeasts). Determination of bactericidal (MBC) and fungicidal (MFC) activity was performed according to the CLSI recommendations.^38,56^

#### 4.3.3 Determination of the MICs of agents in the presence of PAβN

To determine the ability of the Gram-negative bacterial strains to remove newly synthesis compounds by MDR efflux pumps, the MIC values of studied agents, with or without the pump inhibitor, PAβN (20 mg L^−1^) (Sigma) were evaluated.^45^ The MIC determination was performed in MHB with 1 mM MgSO_4_ (Sigma), using 2-fold serial dilutions, according to the CLSI guidelines^53^ in order to compare these two assays. The presence of 1 mM MgSO_4_ stabilizes the outer membrane.^44^ At least a 4-fold decrease in the MIC value after the addition of PAβN was considered significant.^42,43^

### 4.4. Cytotoxicity studies

#### 4.4.1 Culture method

MRC-5 pd30 human fibroblasts were cultured in MEME, Minimum Essential Medium Eagle (Sigma-Aldrich) supplemented with 10% fetal bovine serum (Sigma-Aldrich), 2 mM l-glutamine, antibiotics (100 U mL^−1^ penicillin, 100 μg mL^−1^ streptomycin, Sigma-Aldrich) and 1% non-essential amino-acids (Sigma-Aldrich). Cells were grown in 75 cm^2^ cell culture flasks (Sarstedt), in a humidified atmosphere of CO_2_/air (5/95%) at 37 °C.

#### 4.4.2 MTT-based viability assay

Stock solutions of the tested compounds were prepared in DMSO, so the final concentration of vehicle was 0.5% in each case. For the cytotoxicity studies 2-fold serial dilutions were prepared in the proper medium containing 0.5% DMSO. All the experiments were performed in exponentially growing cultures. Before the treatment MRC-5 cells were trypsinized in 0.25% trypsin–EDTA solution (Sigma-Aldrich) and seeded into 96-well microplates (Sarstedt) at a density of 6 × 10^3^ cells per well. Cells were treated with the tested compounds or DMSO (0.5%) at the appropriate concentrations 18 h after plating. After 72 h incubation with the compounds, the supernatants were discarded, and subsequently MTT stock solution (Sigma-Aldrich) was added to each well to the final concentration of 1 mg mL^−1^. After 1 h incubation at 37 °C, water-insoluble dark blue formazan crystals were dissolved in DMSO (100 μL) (37 °C/10 min incubation). Optical densities were measured at 570 nm using BioTek microplate reader. All measurements were carried out in three replicates and the results expressed as a percent of viable cells *versus* control cells.

## Author contributions

Conceptualization: S. L.; A. E. L., funding acquisition: S. L.; investigation: P. P., J. K., P. W., J. D., U. G., J. M., K. D., K. W., project administration: S. L.; supervision: S. L., A. E. L., visualization: P. P., J. K., P. W., writing – original draft – P. P., J. K., P. W., S. L., A. E. L. writing – review & editing – P. P., S. L., A. E. L.

## Conflicts of interest

There are no conflicts to declare.

## Supplementary Material

RA-011-D1RA04127D-s001

RA-011-D1RA04127D-s002
