# Supplementary material for: Development of structurally extended benzosiloxaboroles – synthesis and in vitro biological evaluation
Source: RSC Adv. 2021 Jul 20;11(41):25104–21. doi: 10.1039/d1ra04127d (PMC9037100; doi:10.1039/d1ra04127d)
Supplement: RA-011-D1RA04127D-s001 [file RA-011-D1RA04127D-s001.pdf]

Electronic Supplementary Material (ESI) for RSC Advances.
This journal is © The Royal Society of Chemistry 2021

Supporting Information

Development of structurally extended benzosiloxaboroles – synthesis and *in vitro* biological evaluation

P. Pacholak,[a,b] J. Krajewska,[c] P. Wińska,[a] J. Dunikowska,[a] U. Gogowska,[a] J. Mierzejewska,[a] K. Durka,[a] K. Woźniak,[b] A. E. Laudy**,[c] S. Luliński*[a]

[a] *Faculty of Chemistry, Warsaw University of Technology, Noakowskiego 3, 00-664 Warsaw, Poland*

[b] *University of Warsaw, Faculty of Chemistry, Warsaw, Poland, Pasteura 1, 02-093*

[c] *Department of Pharmaceutical Microbiology, Medical University of Warsaw, Oczki 3, 02-007 Warsaw, Poland*

*Corresponding author responsible for chemical research.
Email address: sergiusz.lulinski@pw.edu.pl (S. Luliński).
**Corresponding author responsible for microbiological research.
Email address: alaudy@wp.pl (A. E. Laudy).

Keywords: benzosiloxaboroles, antifungal activity, antibacterial activity, molecular docking, efflux pump, Eagle effect

Table of content

1. NMR spectra 2
2. Single-crystal X-ray diffraction analysis 43
3. Antimicrobial activity. 46
4. Cytotoxic activity 50
5. References 53

1. NMR Spectra

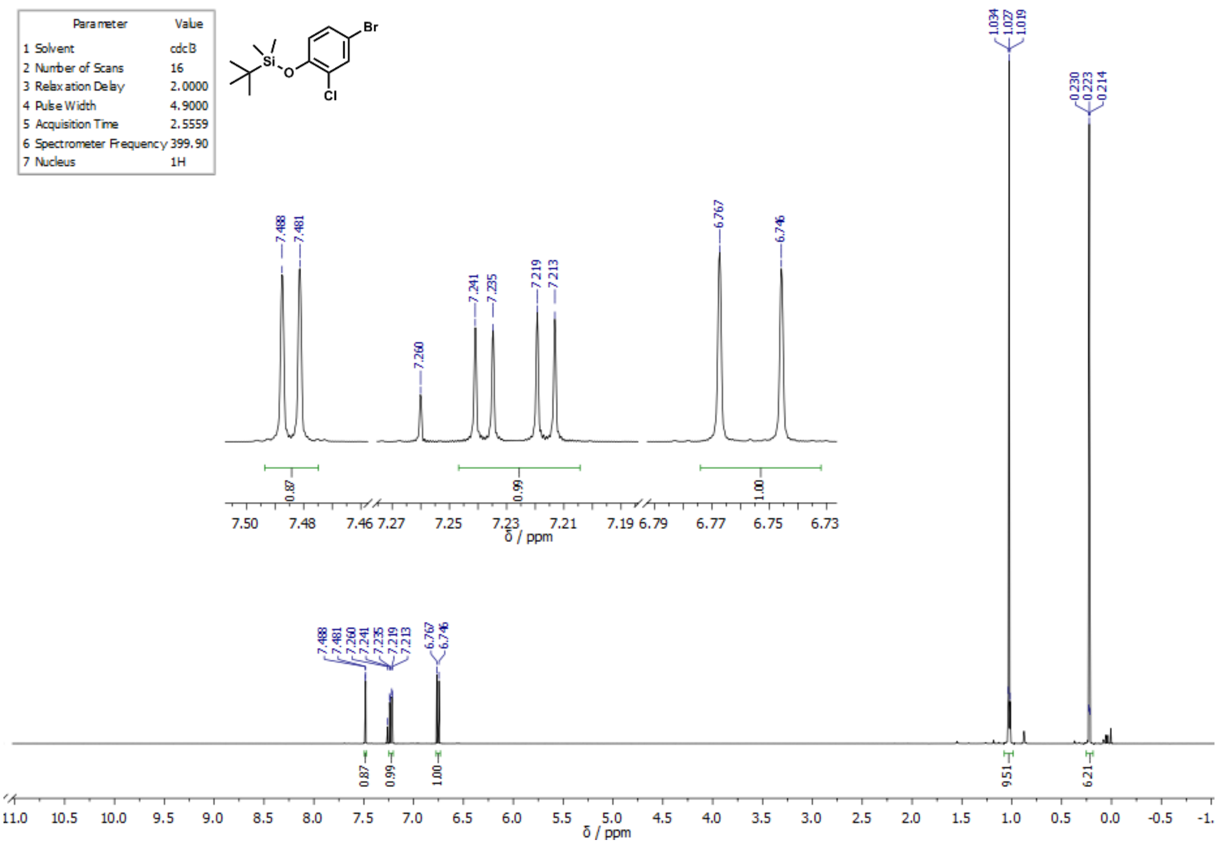

Figure S1. ^{1}H NMR spectrum of **2b** in $CDCl_3$.

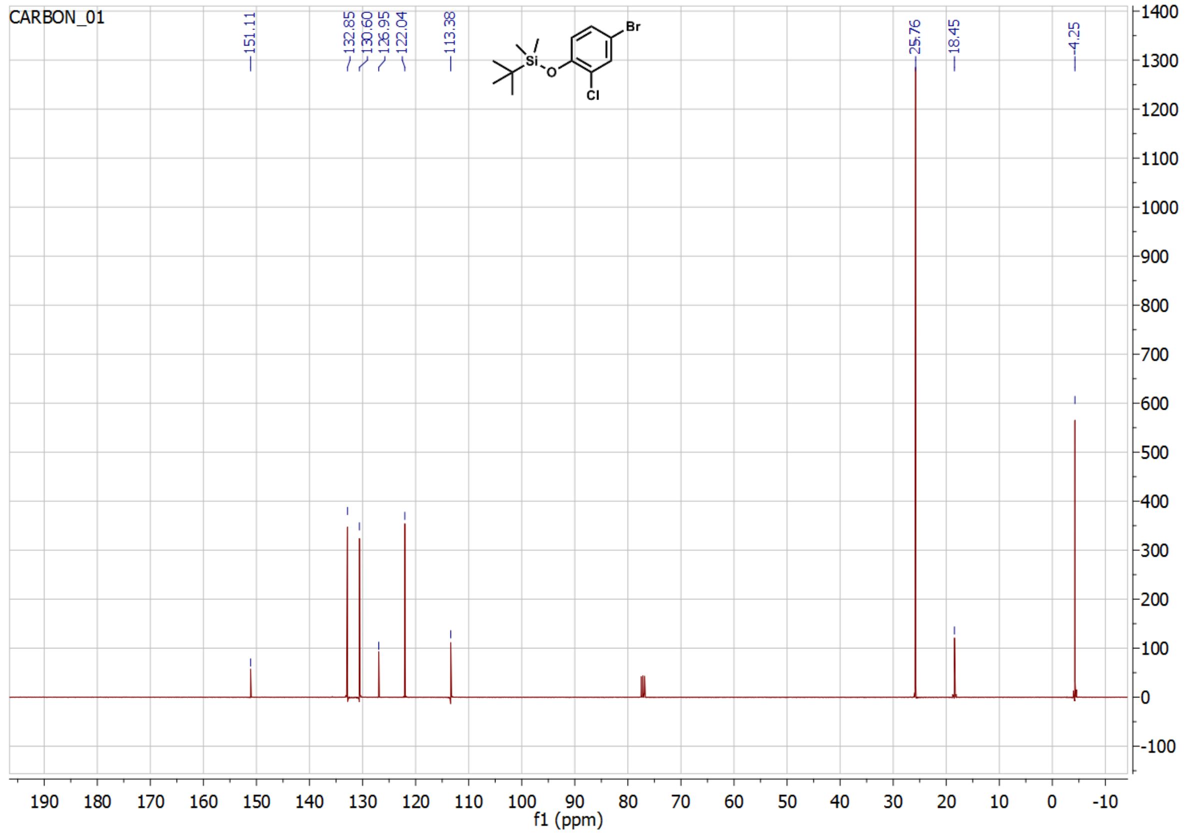

Figure S2. ^{13}C NMR spectrum of **2b** in $CDCl_3$.

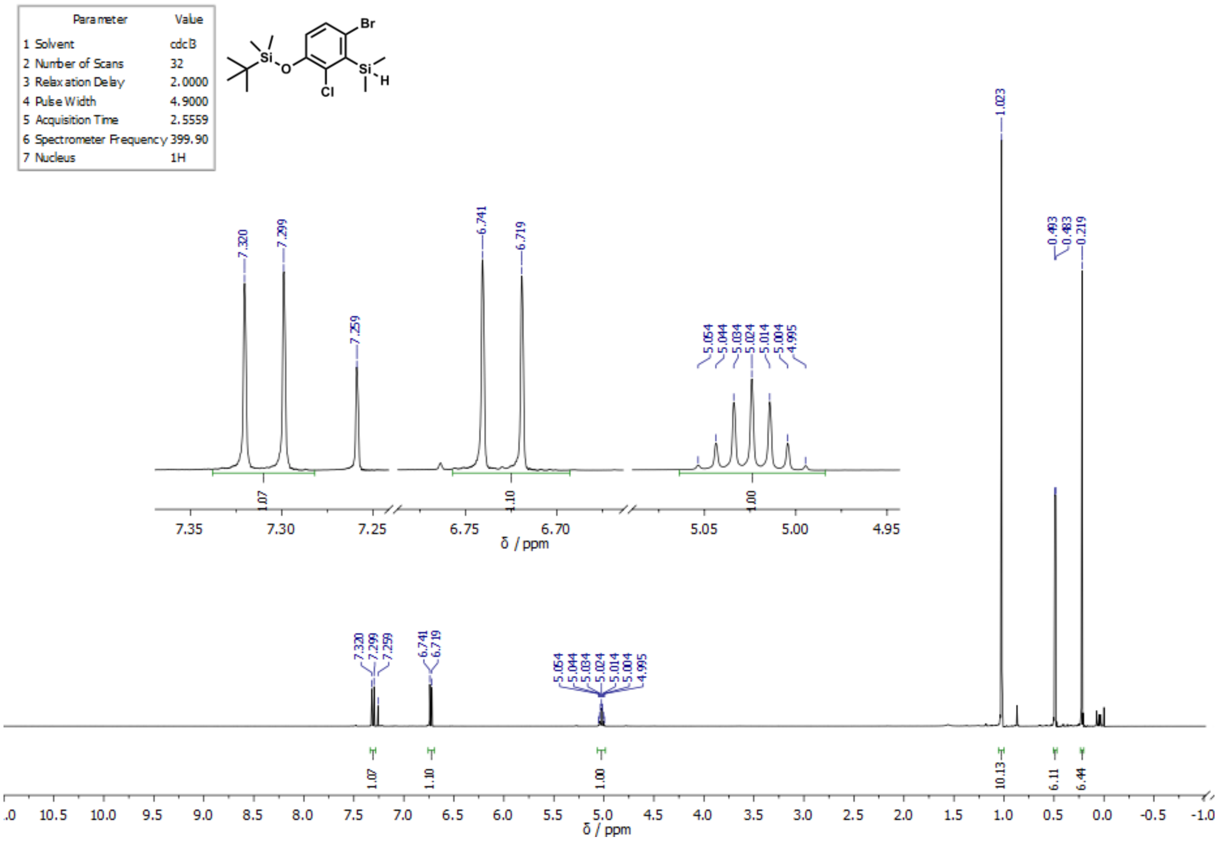

Figure S3. ^{1}H NMR spectrum of **3b** in $CDCl_3$.

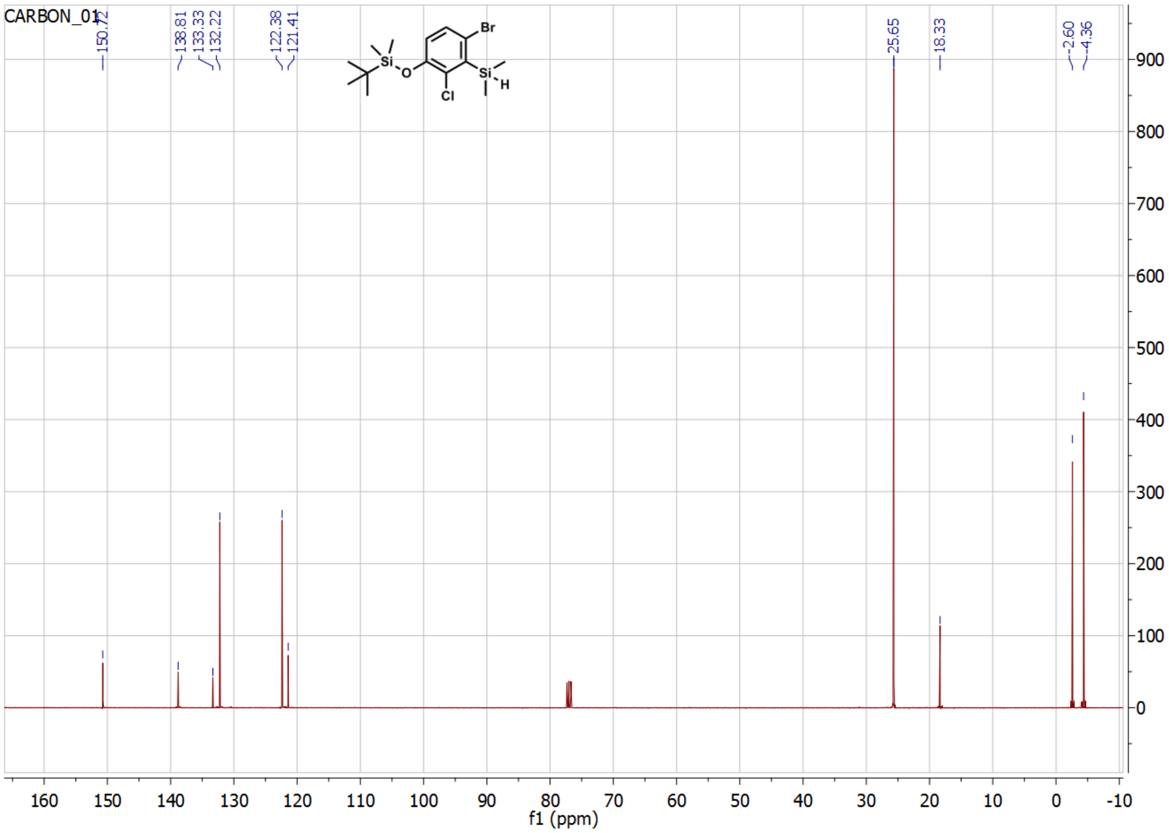

Figure S4. ^{13}C NMR spectrum of **3b** in $CDCl_3$.

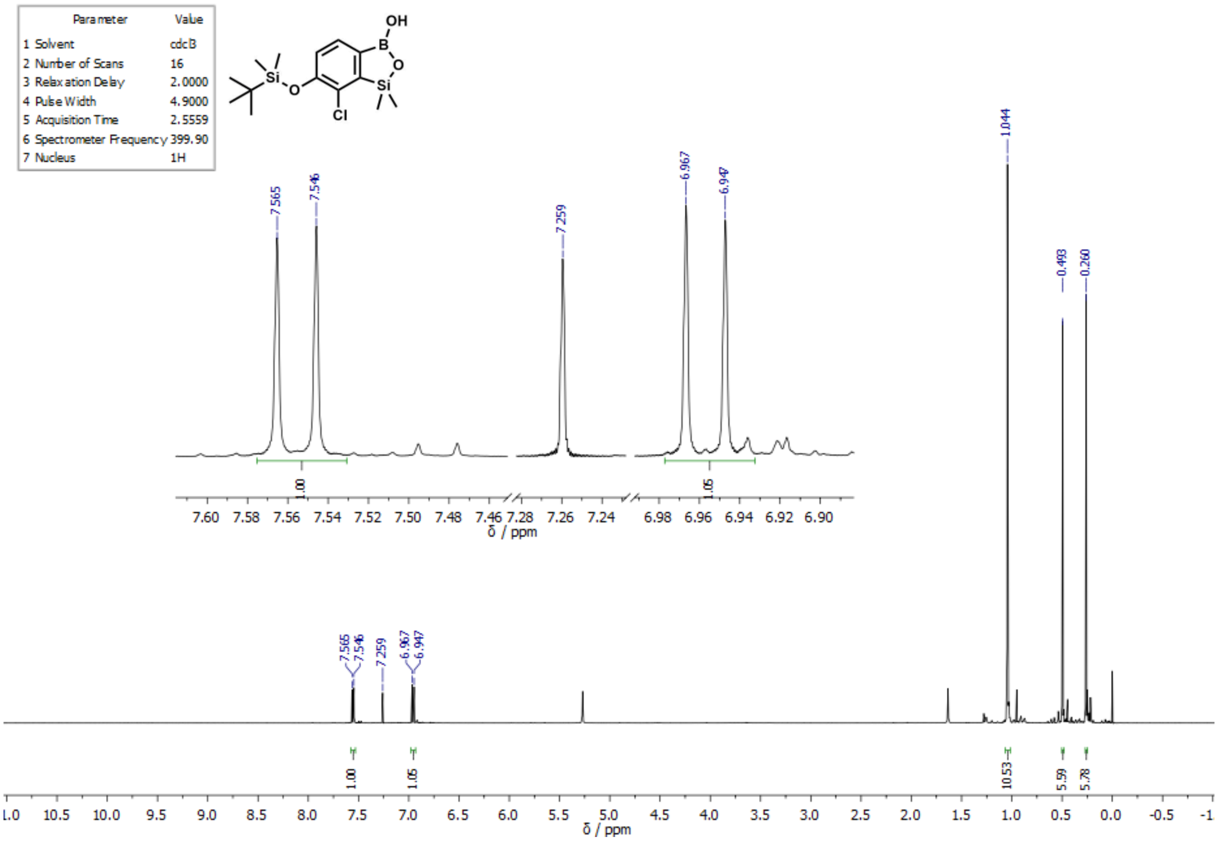

Figure S5. ^{1}H NMR spectrum of **4b** in $CDCl_3$.

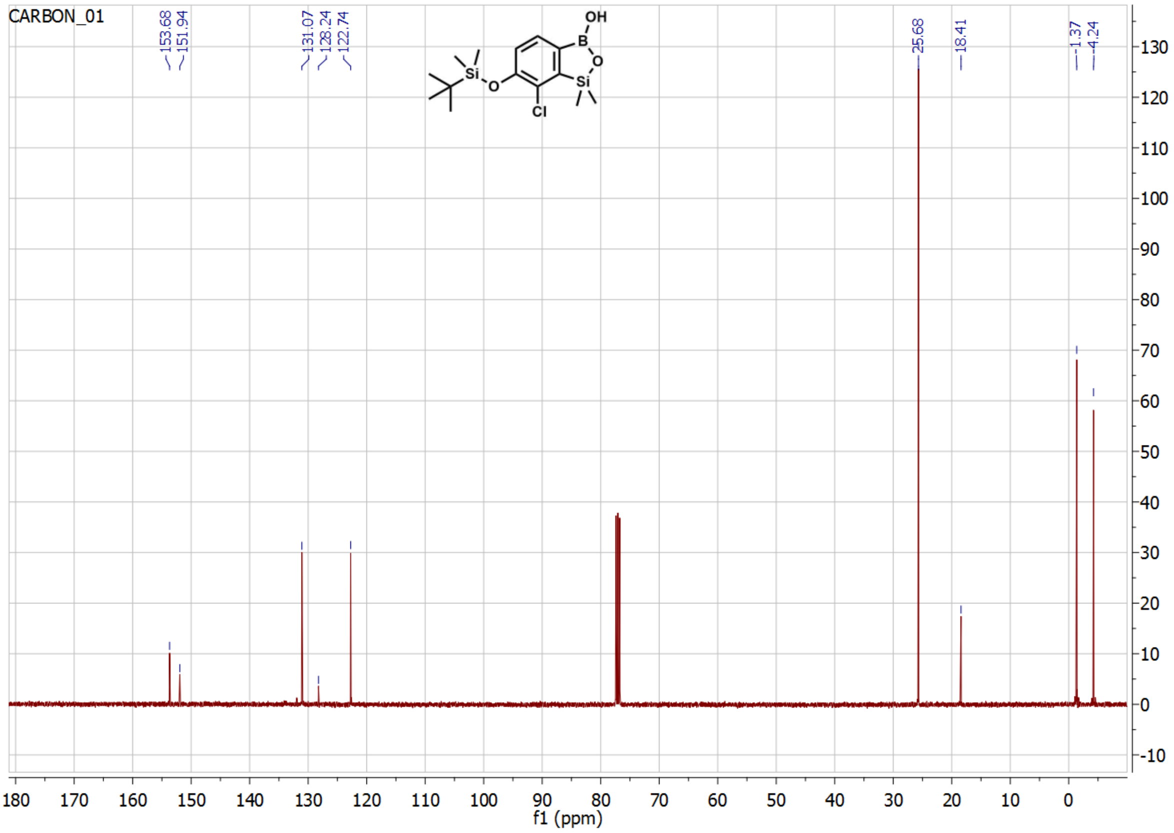

Figure S6. ^{13}C NMR spectrum of **4b** in $CDCl_3$.

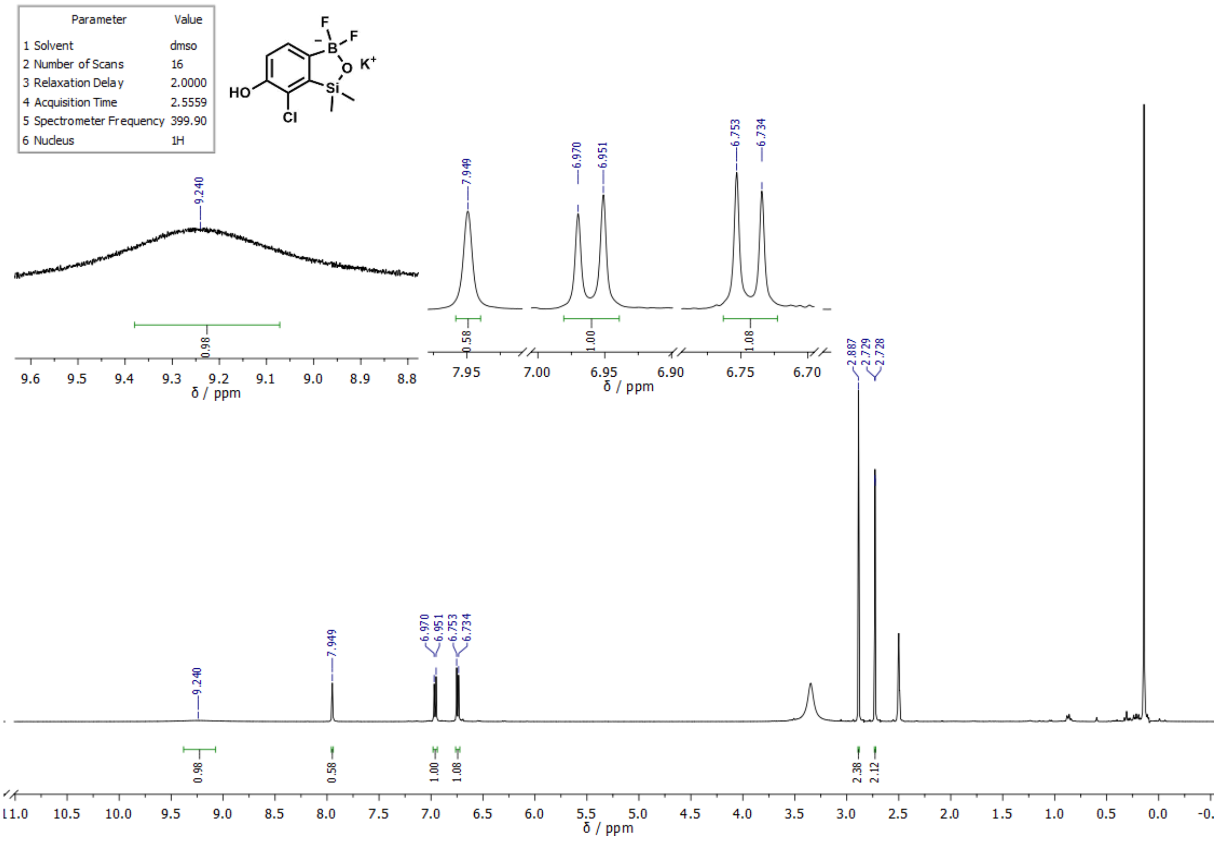

Figure S7. ^{1}H NMR spectrum of **5b** (non-stoichiometric DMF solvate (molar ratio **5b** : DMF = 3:2) in DMSO.

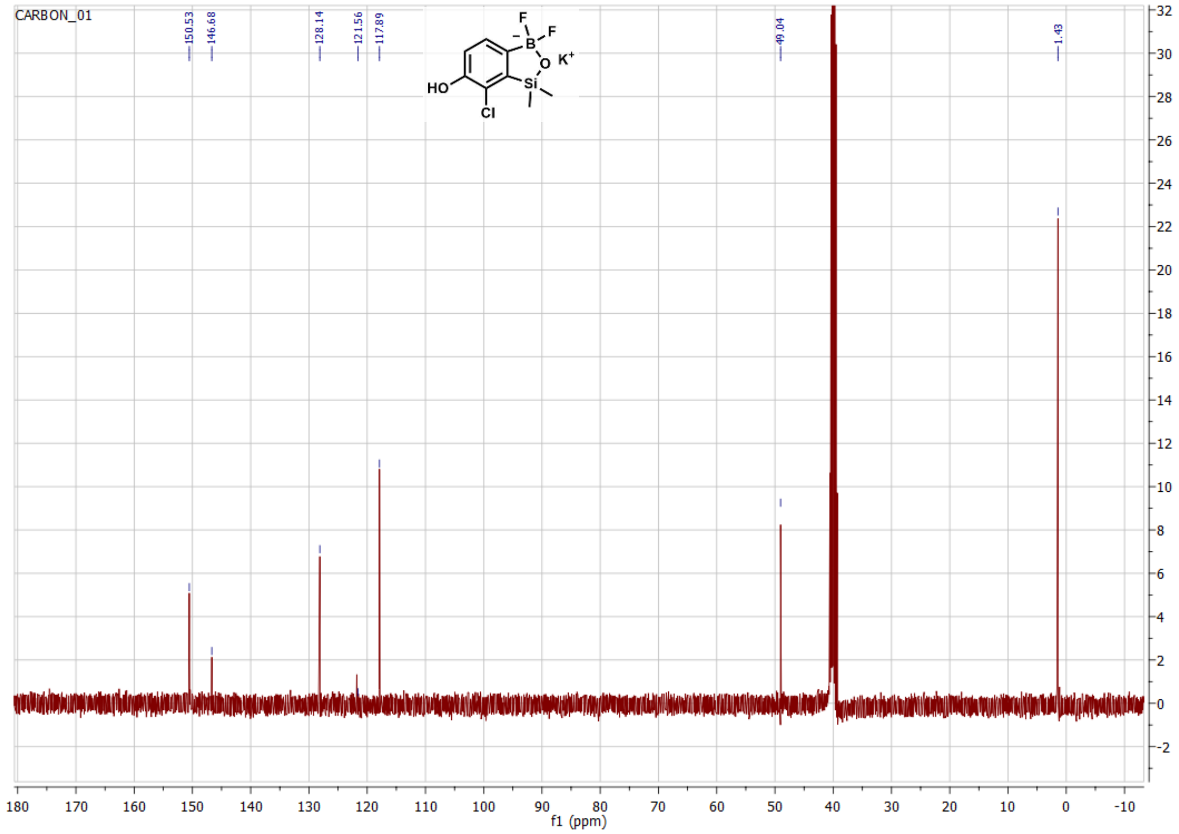

Figure S8. ^{13}C NMR spectrum of **5b** in DMSO.

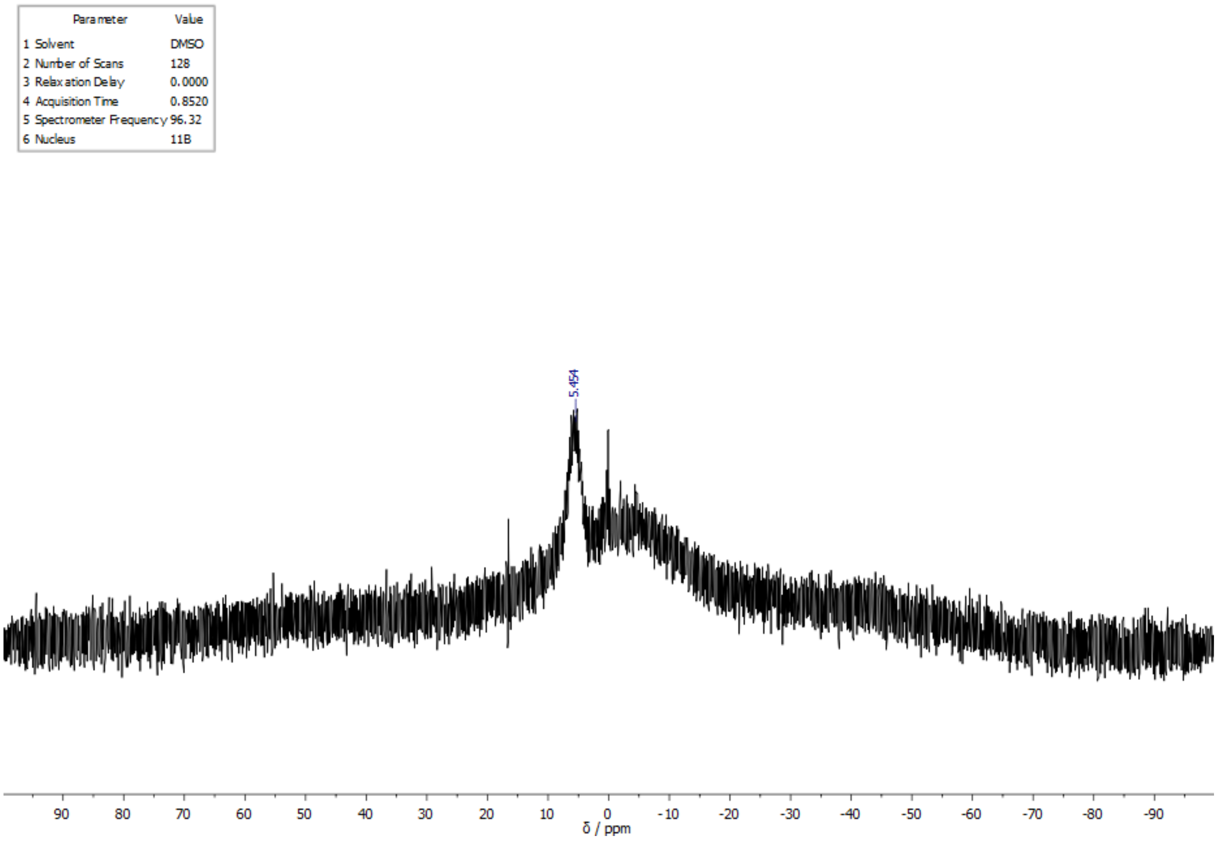

Figure S9. ^{11}B NMR spectrum of **5b** in DMSO.

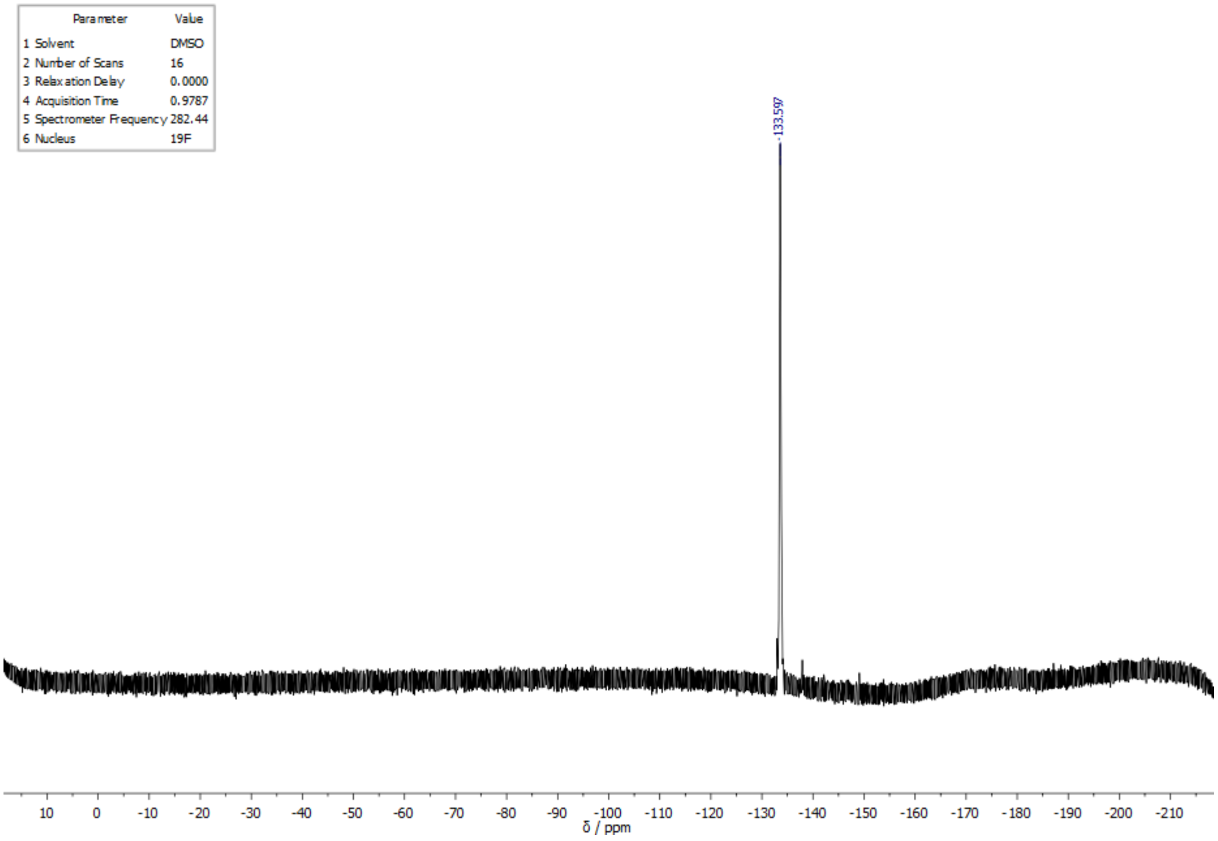

Figure S10. ^{19}F NMR spectrum of **5b** in DMSO.

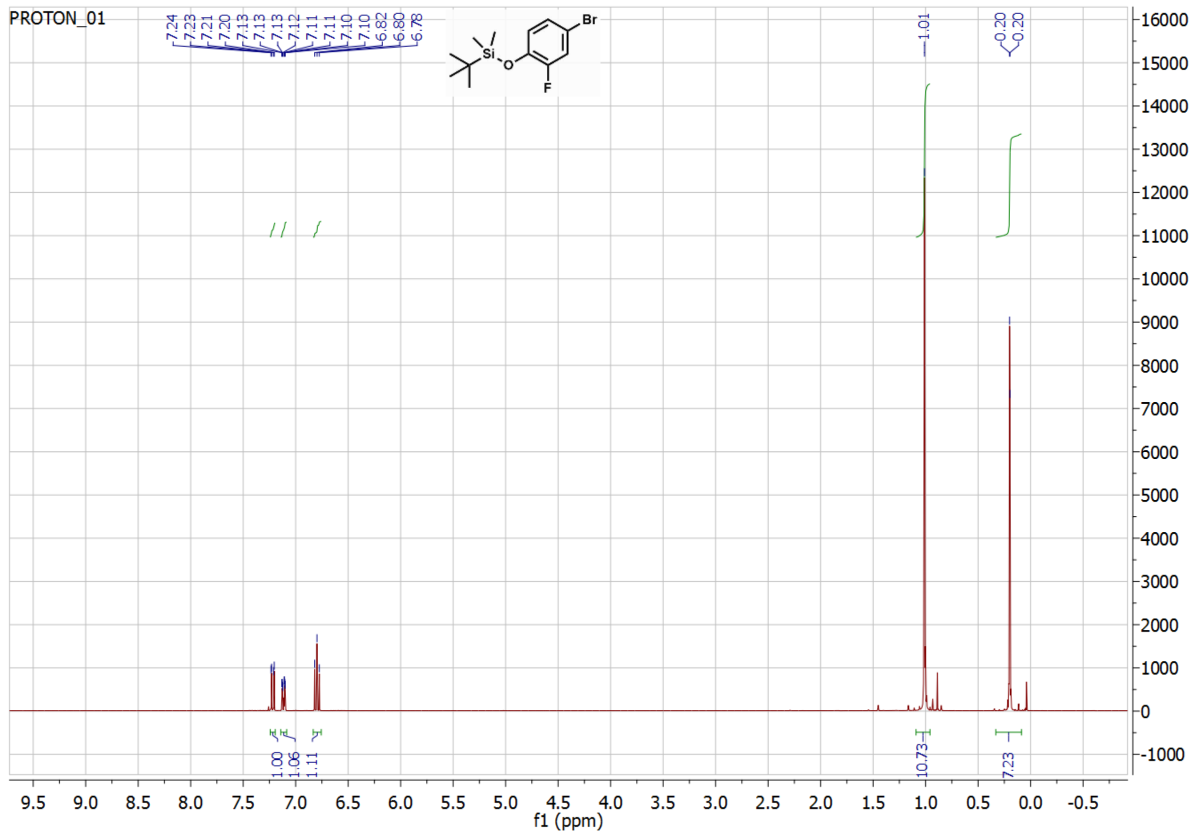

Figure S11. ^{1}H NMR spectrum of **2a** in $CDCl_3$.

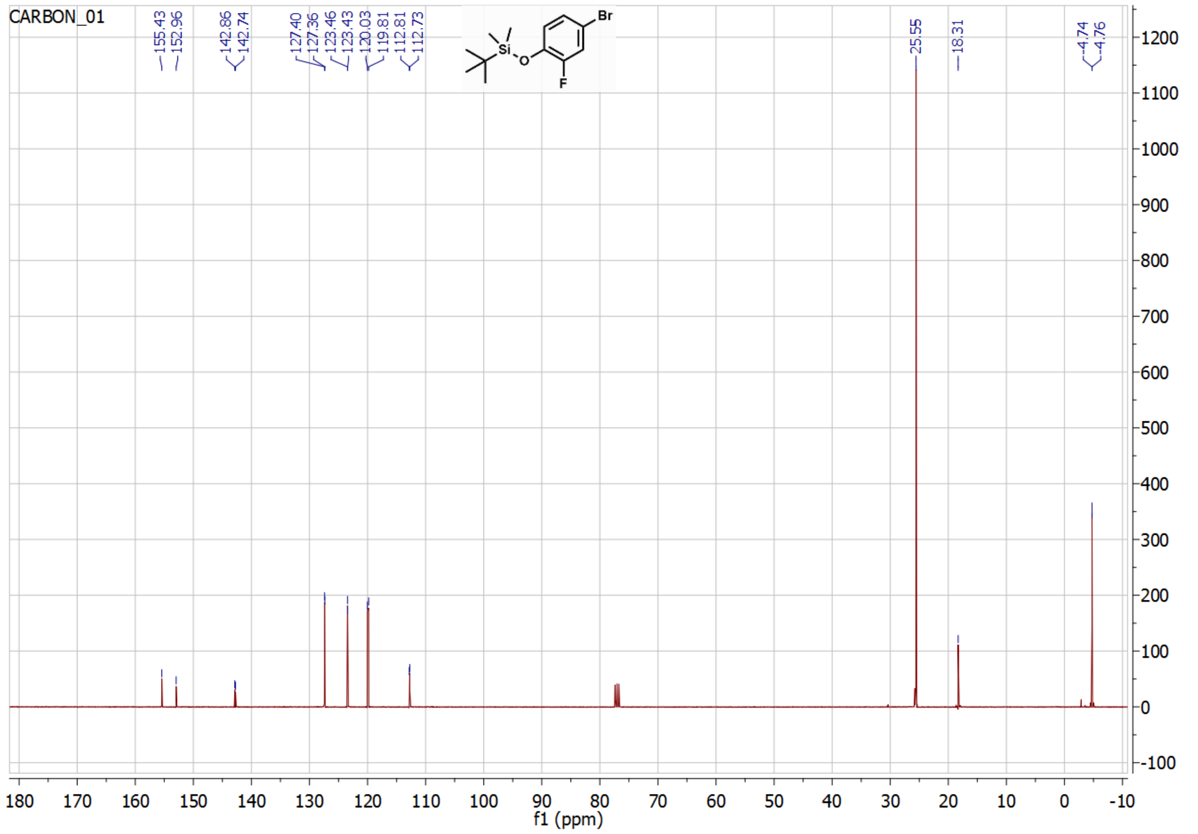

Figure S12. ^{13}C NMR spectrum of **2a** in $CDCl_3$.

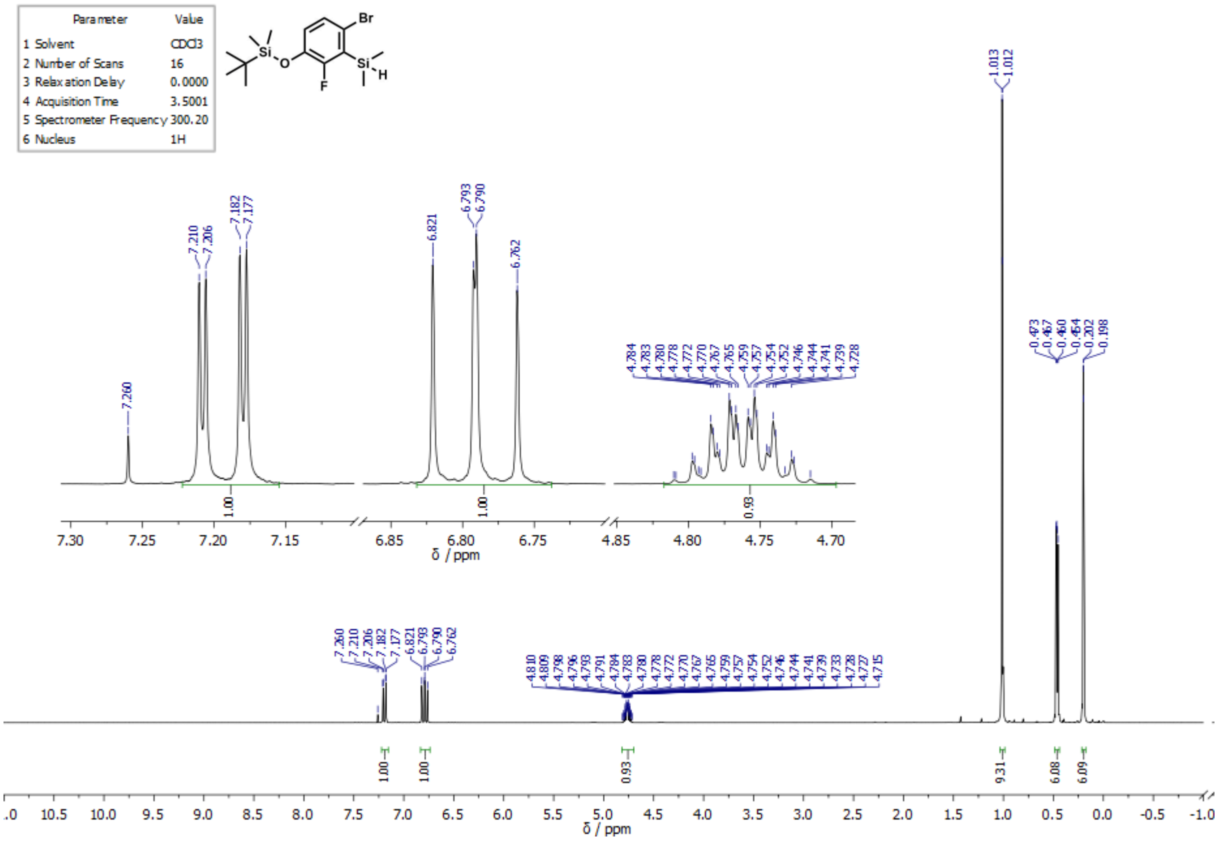

Figure S13. ^{1}H NMR spectrum of **3a** in $CDCl_3$.

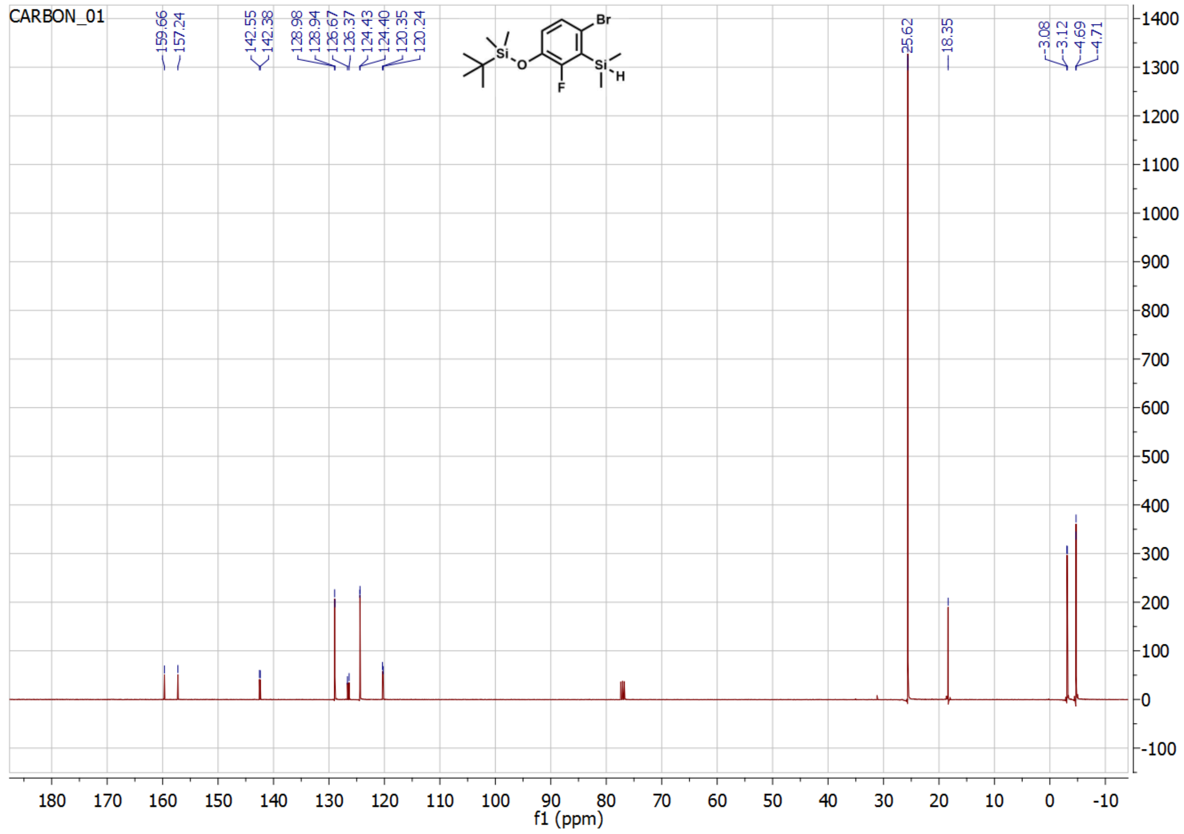

Figure S14. ^{13}C NMR spectrum of **3a** in $CDCl_3$.

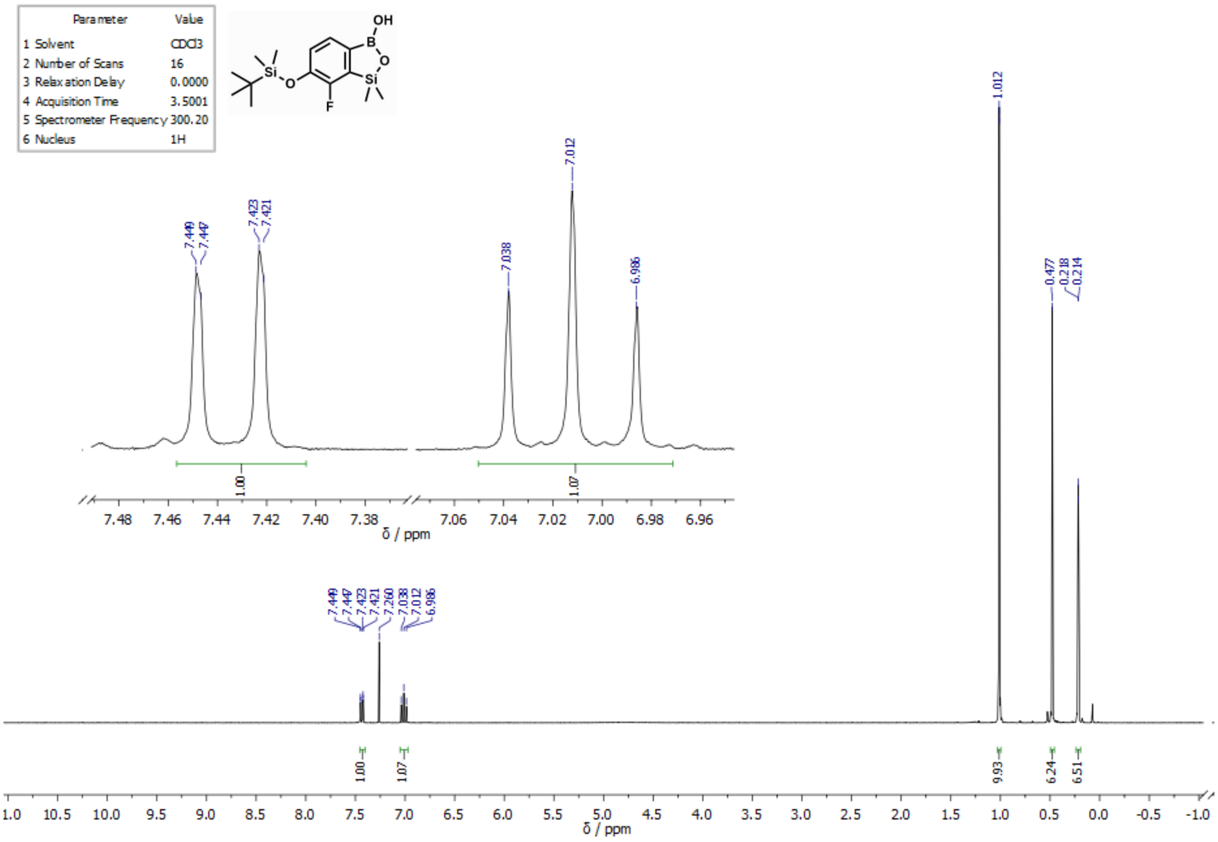

Figure S15. ^{1}H NMR spectrum of **4a** in $CDCl_3$.

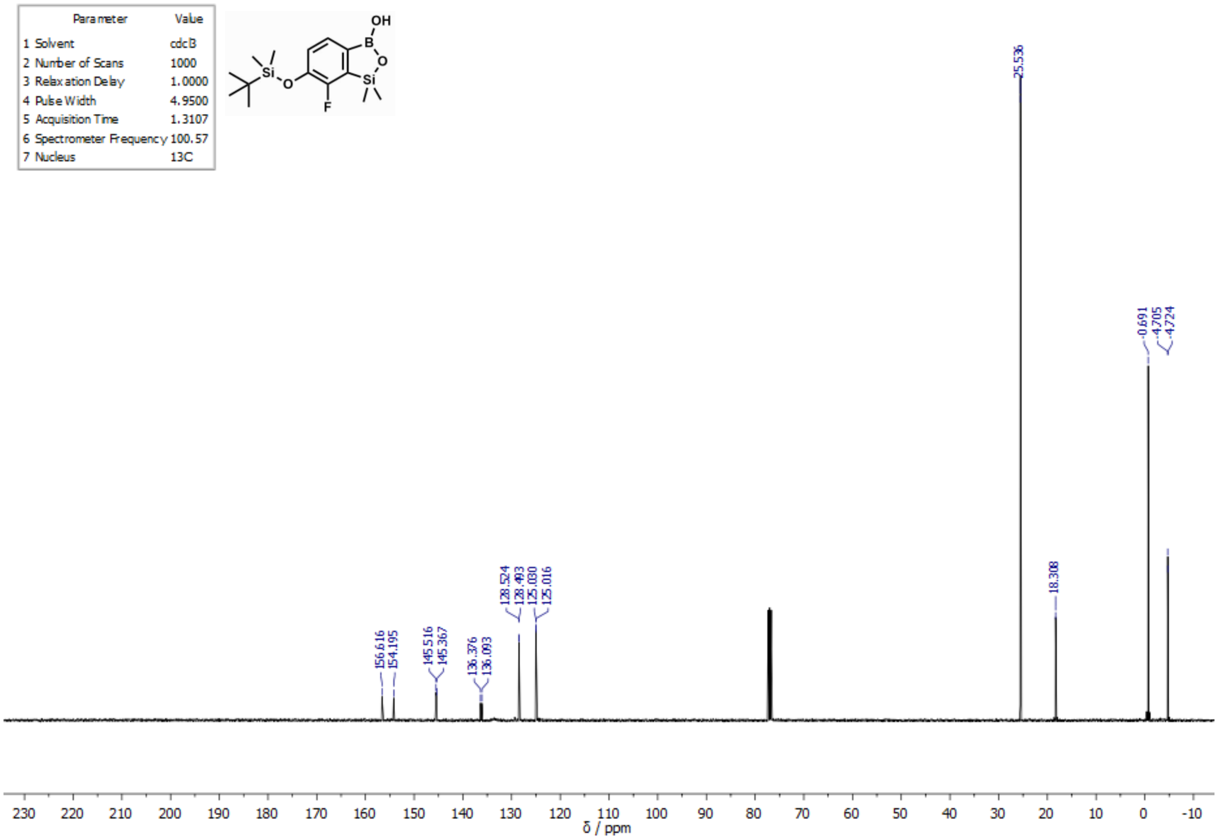

Figure S16. ^{13}C NMR spectrum of **4a** in $CDCl_3$.

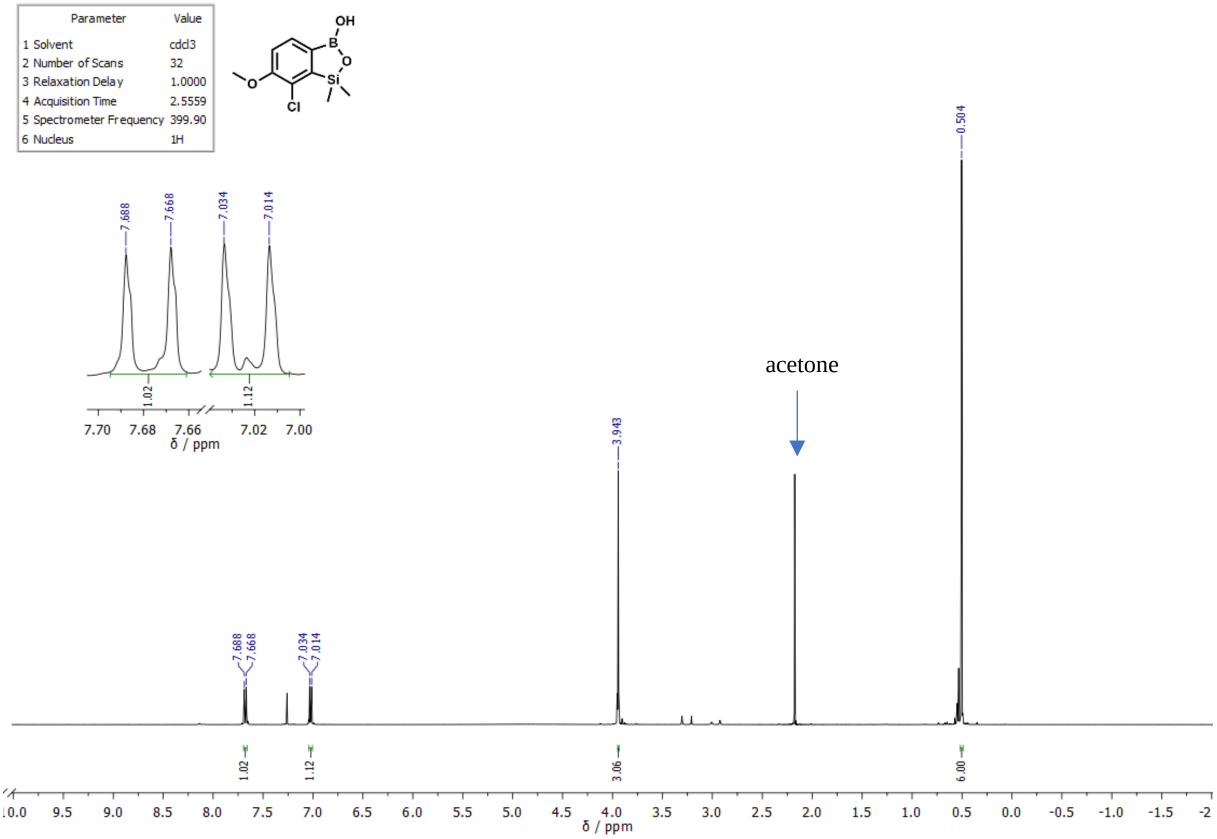

Figure S17. ^{1}H NMR spectrum of **6** in $CDCl_3$.

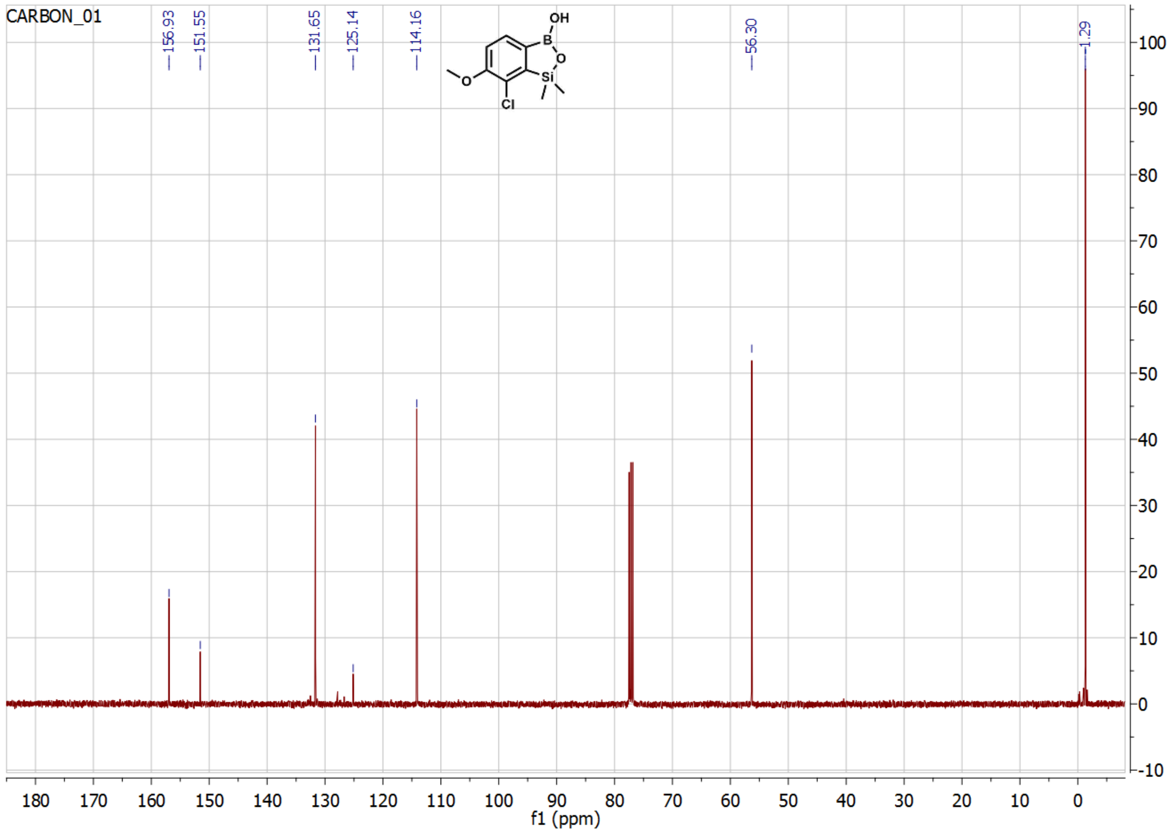

Figure S18. ^{13}C NMR spectrum of **6** in $CDCl_3$.

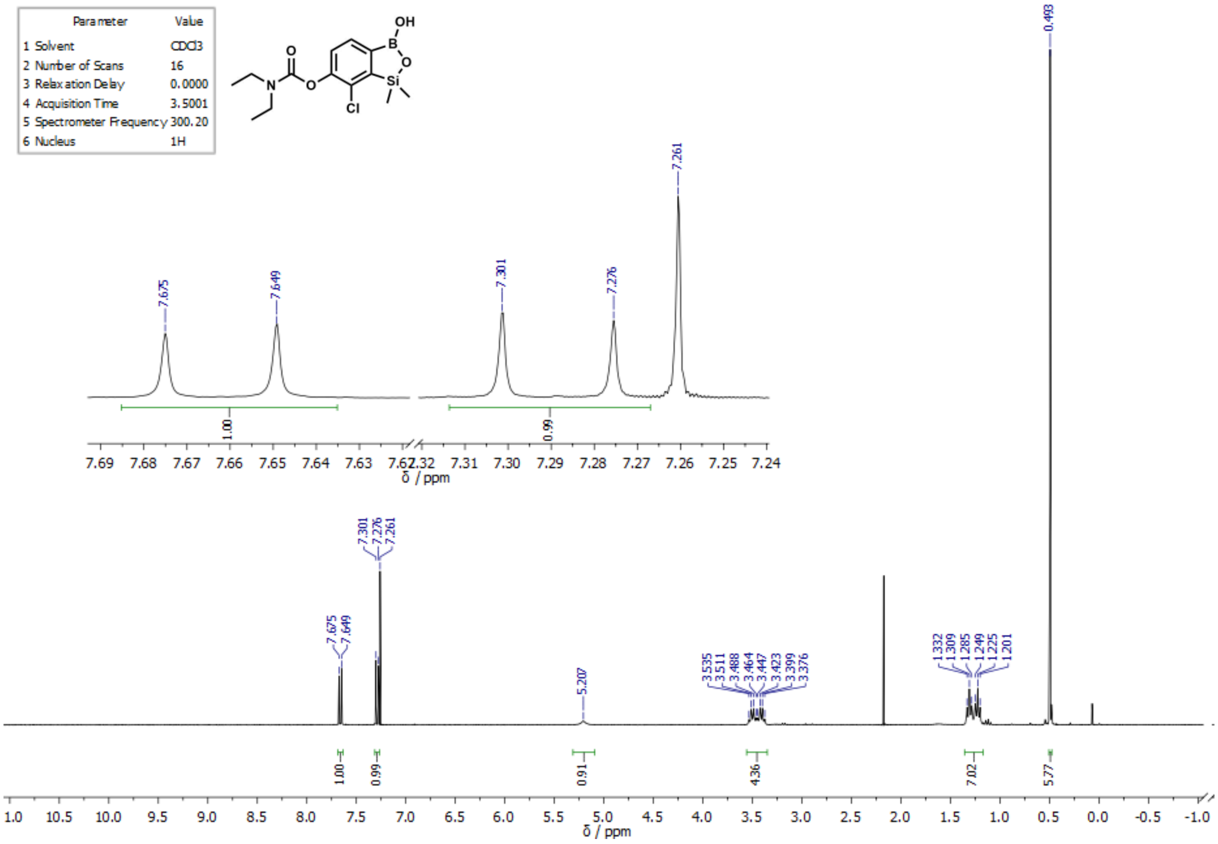

Figure S19. ^{1}H NMR spectrum of **7** in $CDCl_3$.

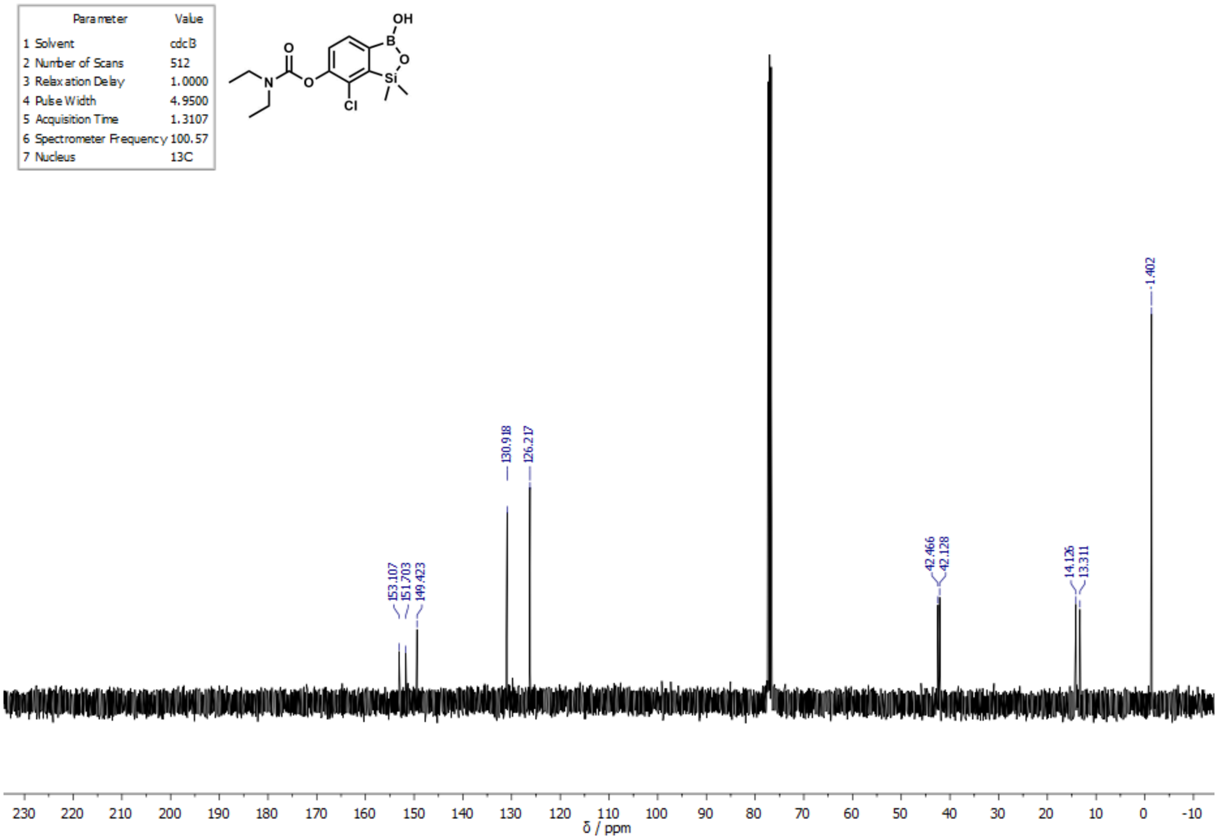

Figure S20. ^{13}C NMR spectrum of **7** in $CDCl_3$.

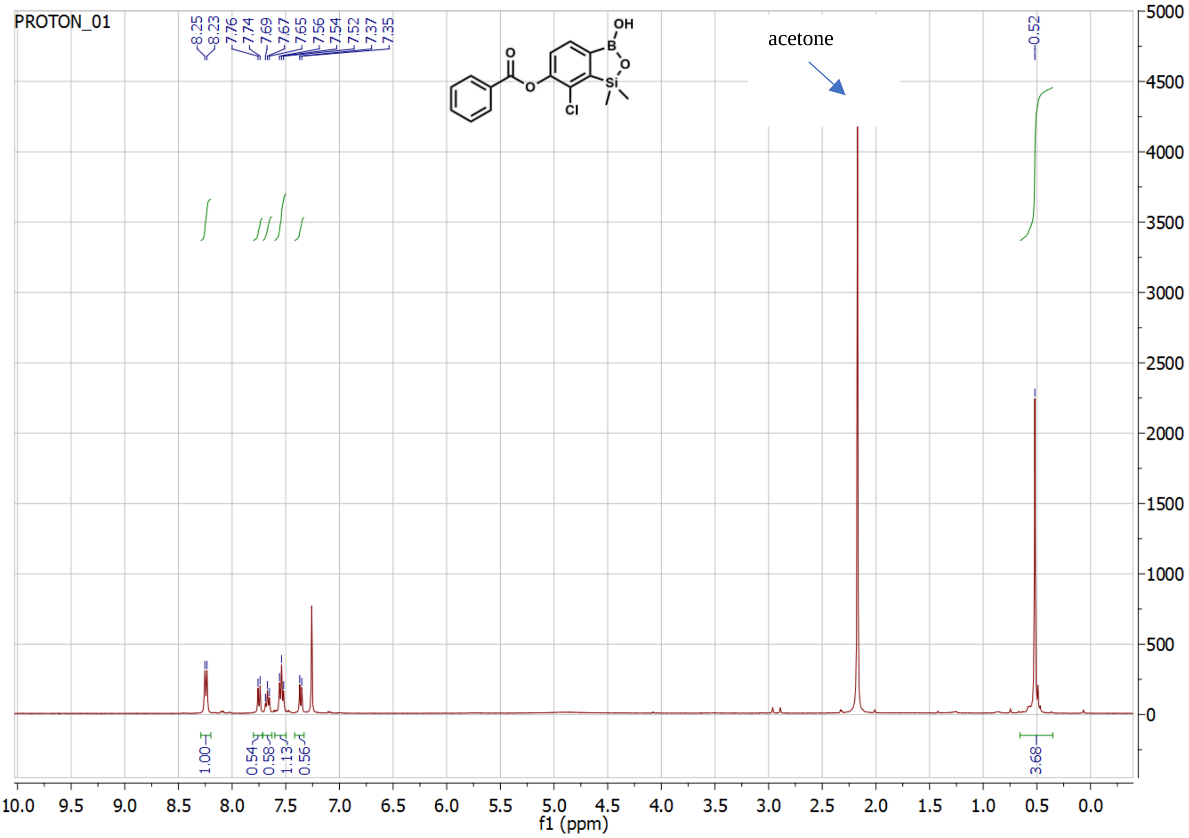

Figure S21. ^{1}H NMR spectrum of **8a** in $CDCl_3$.

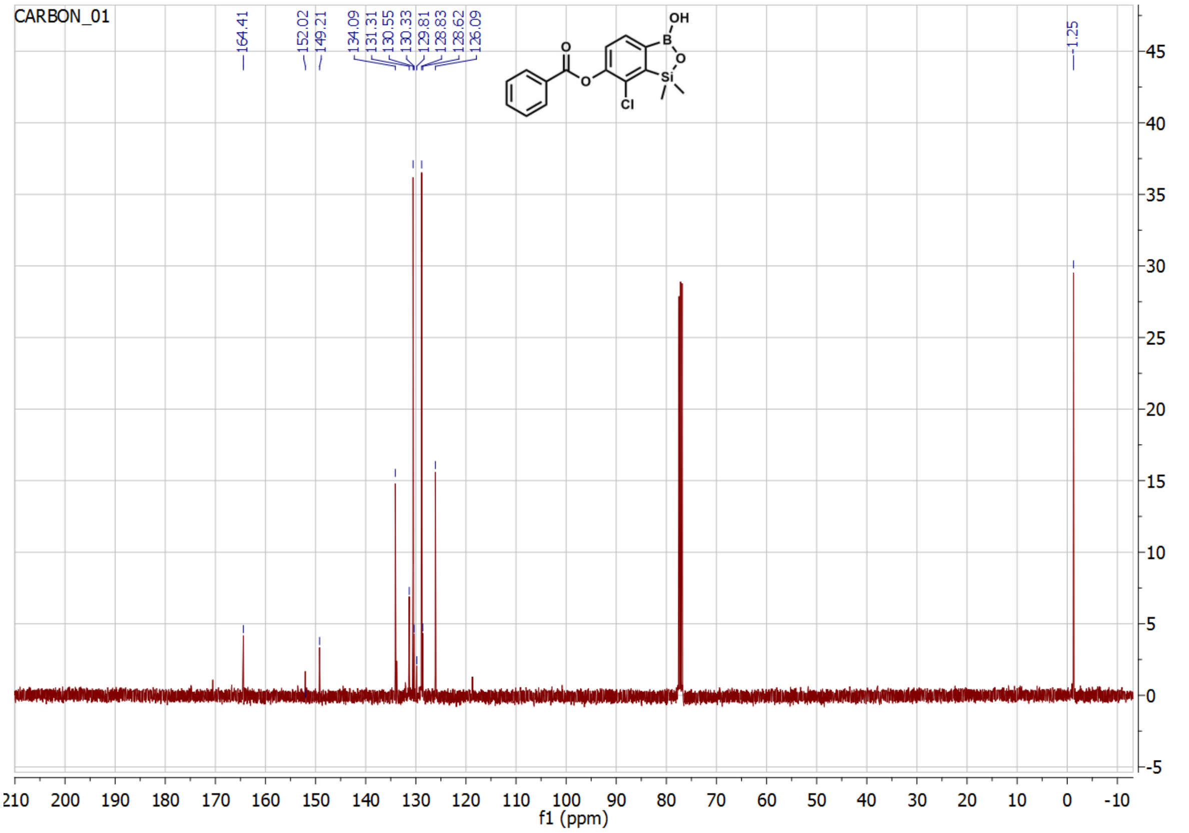

Figure S22. ^{13}C NMR spectrum of **8a** in $CDCl_3$.

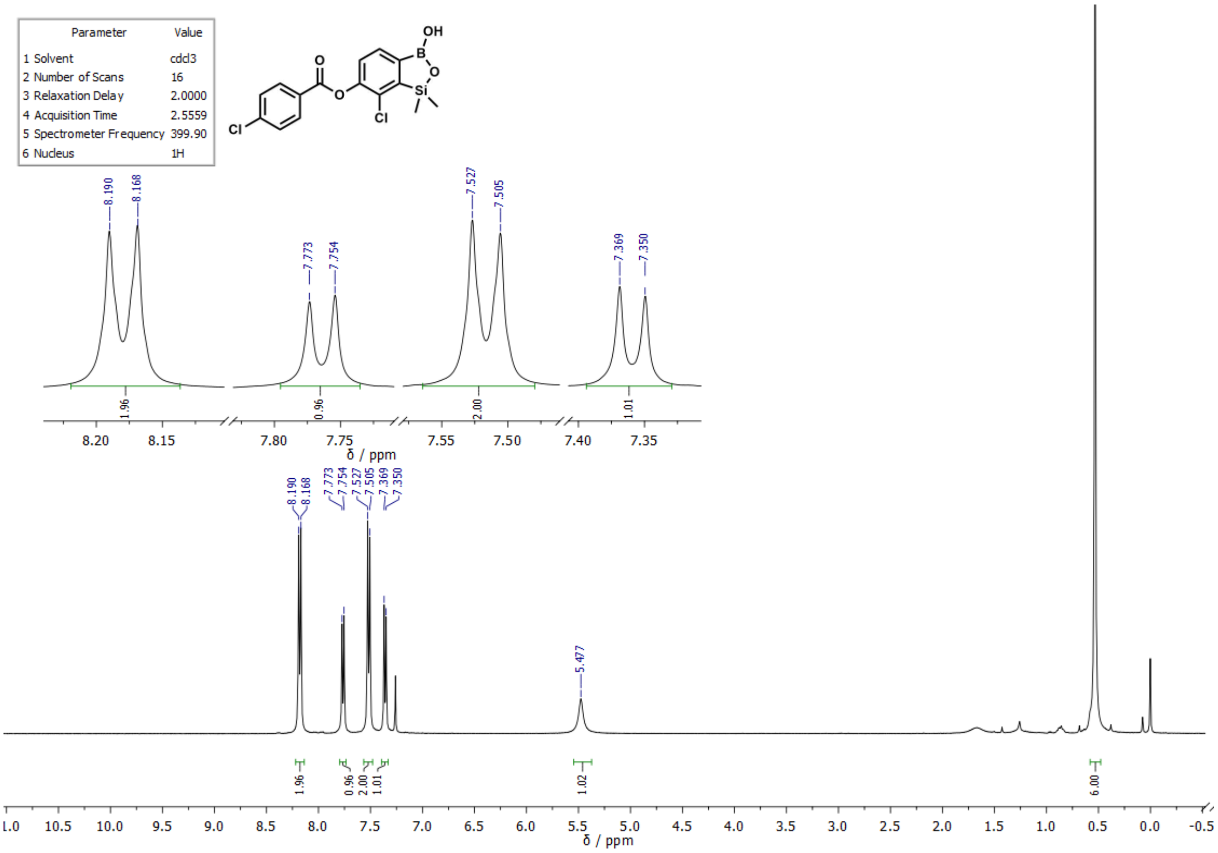

Figure S23. ^{1}H NMR spectrum of **8b** in $CDCl_3$.

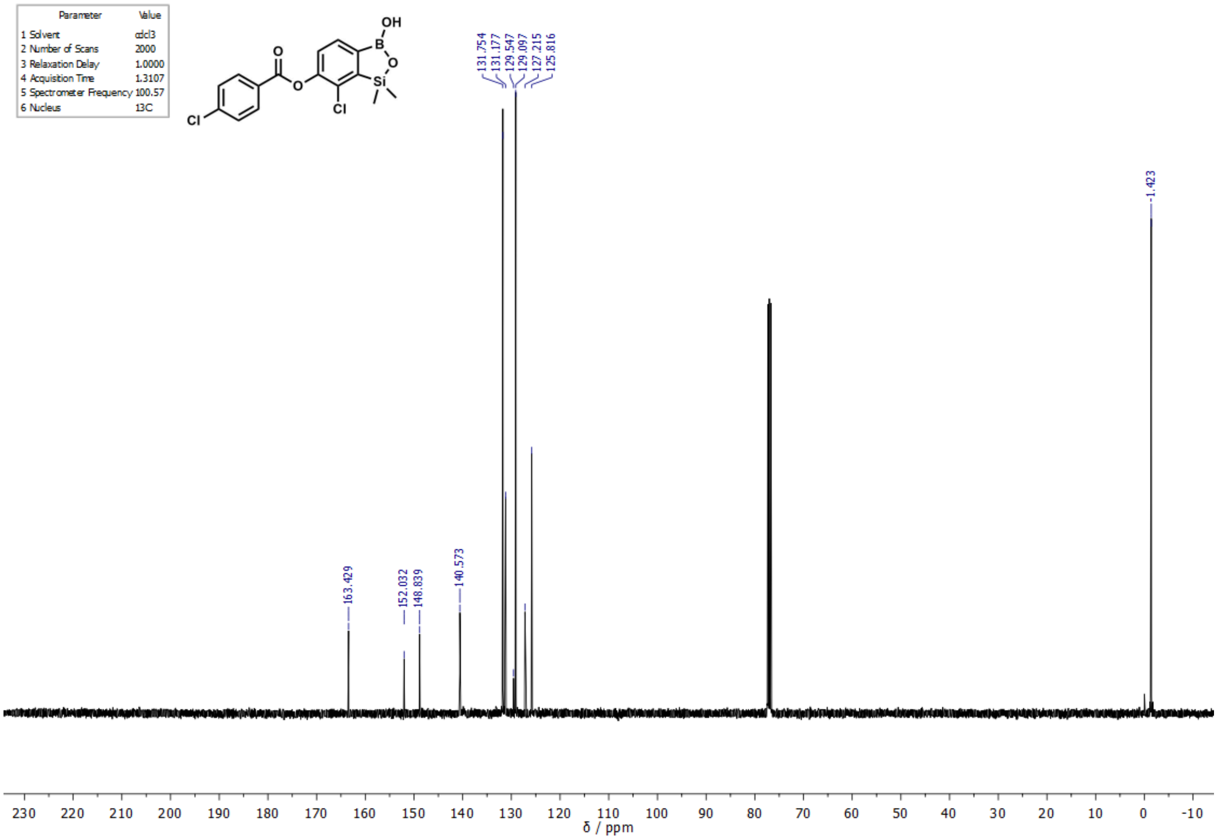

Figure S24. ^{13}C NMR spectrum of **8b** in $CDCl_3$.

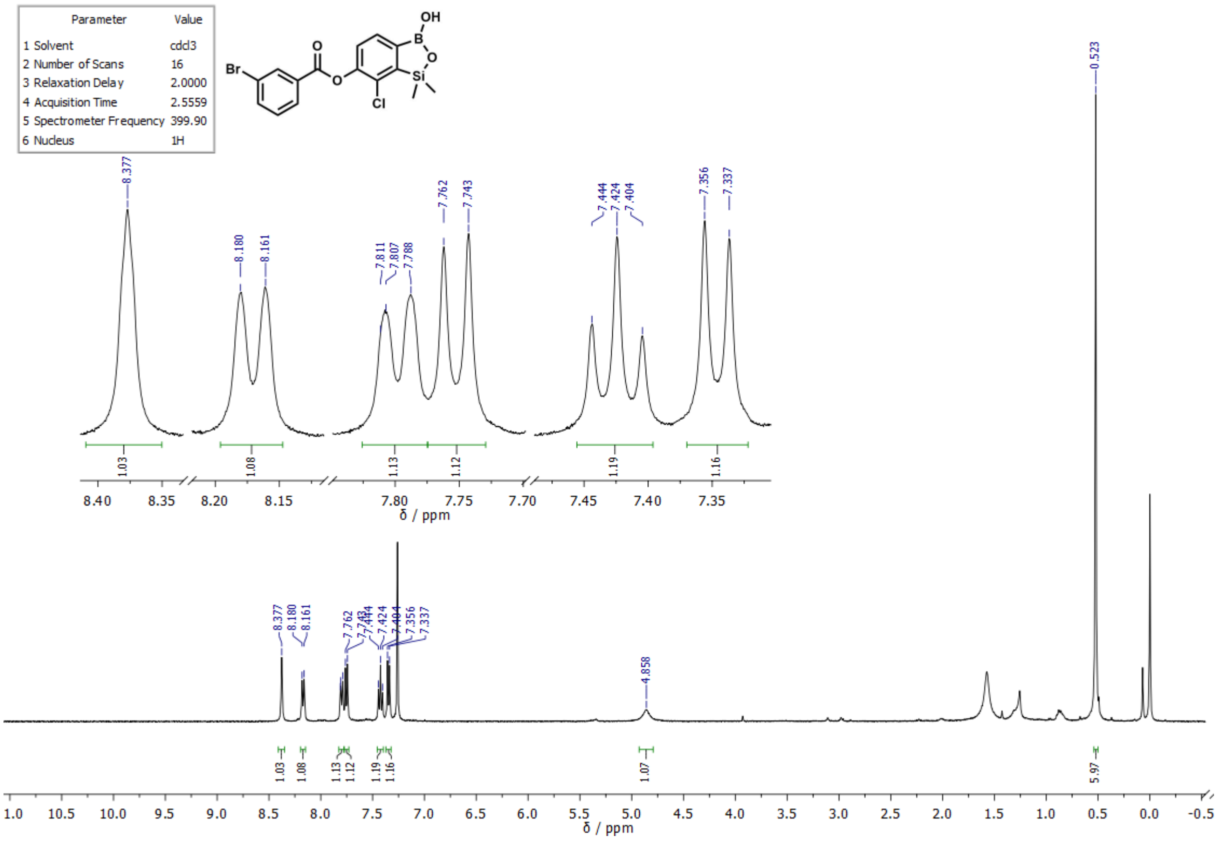

Figure S25. ^{1}H NMR spectrum of **8c** in $CDCl_3$.

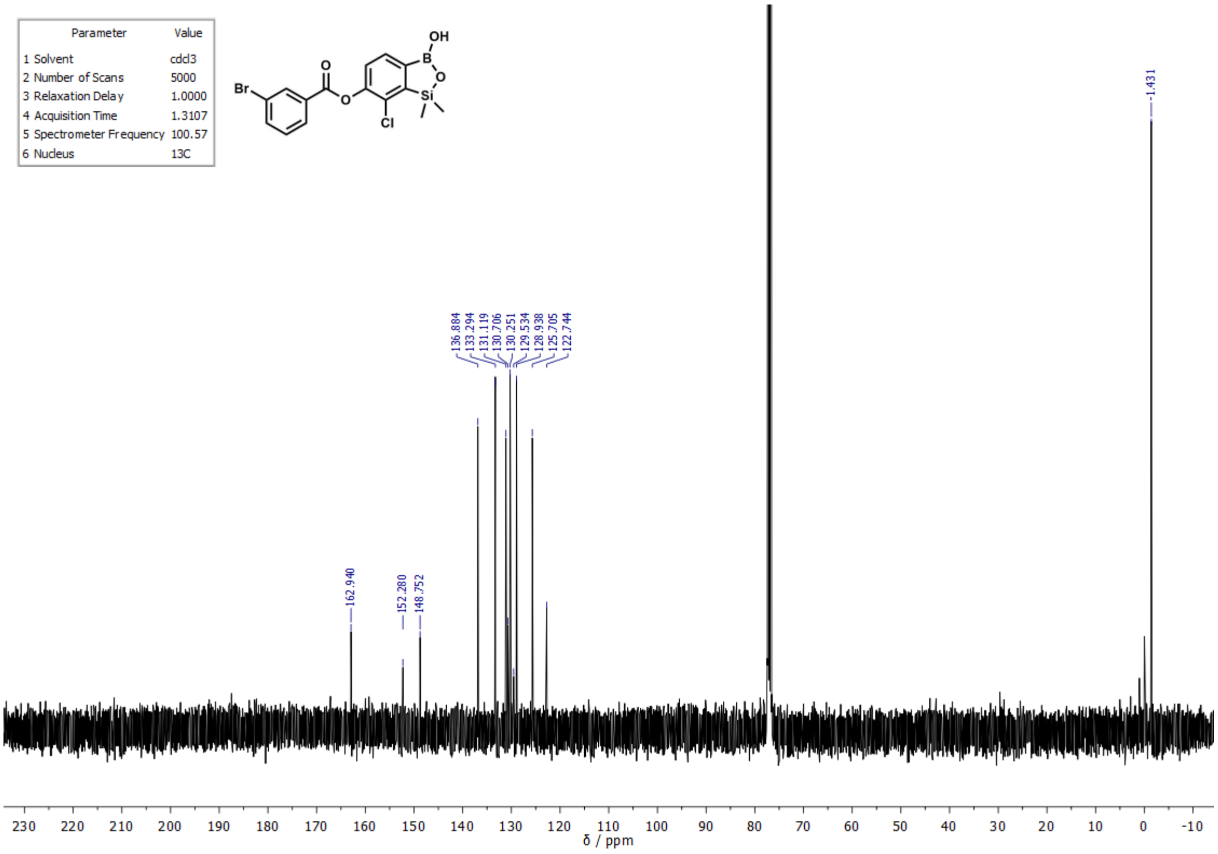

Figure S26. ^{13}C NMR spectrum of **8c** in $CDCl_3$.

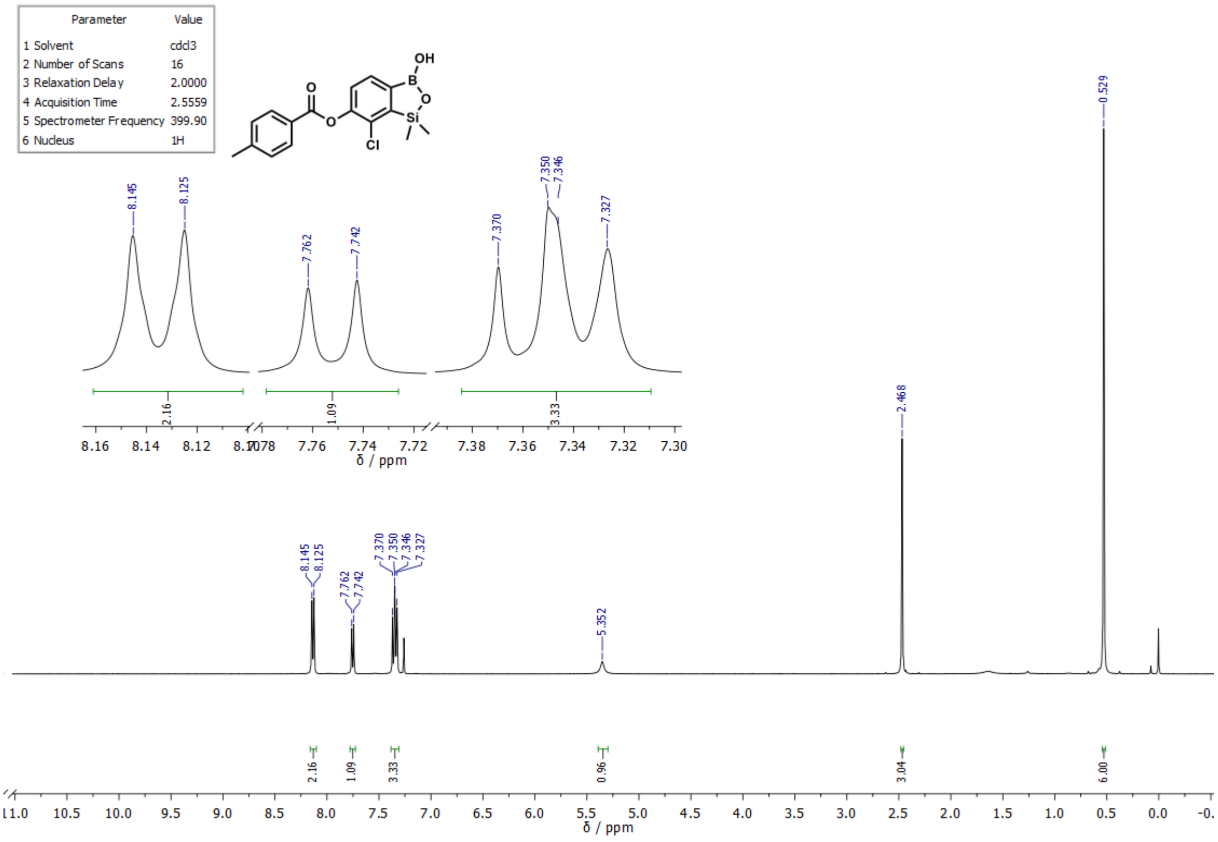

Figure S27. ^{1}H NMR spectrum of **8d** in $CDCl_3$.

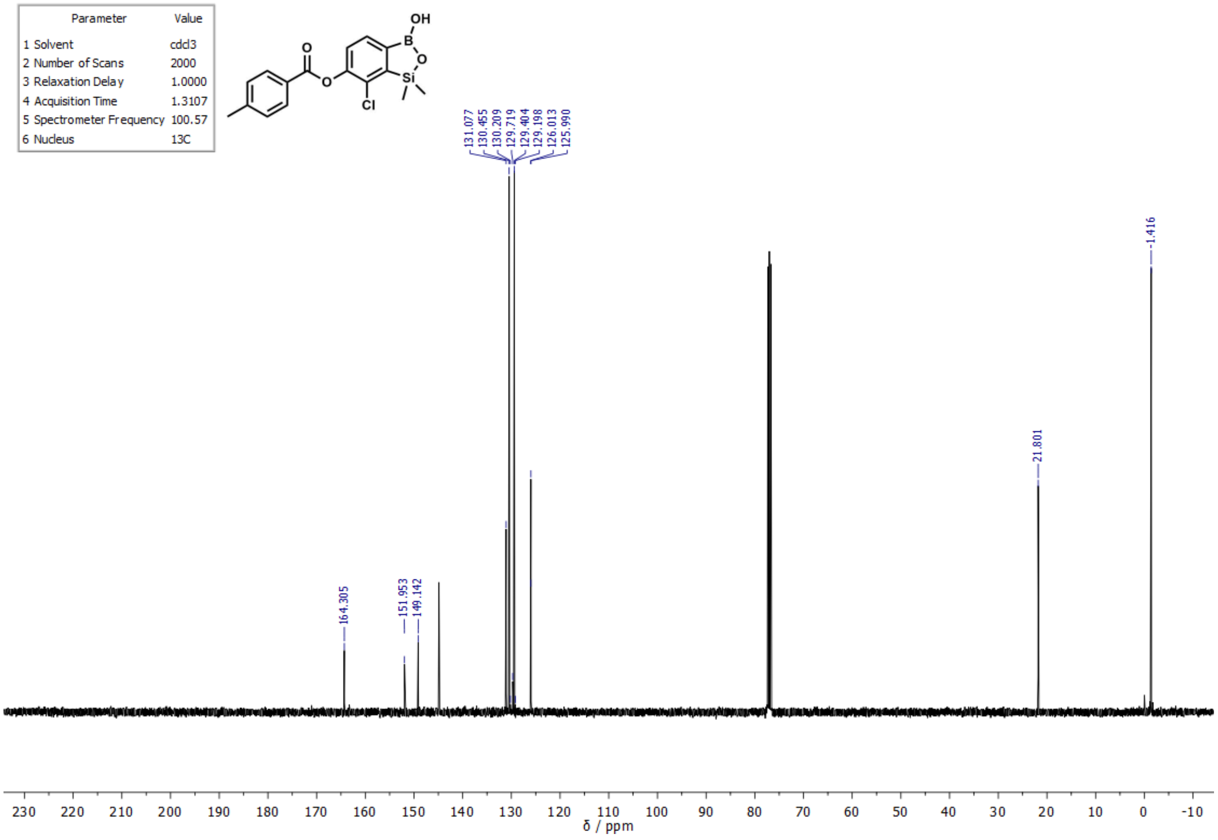

Figure S28. ^{13}C NMR spectrum of **8d** in $CDCl_3$.

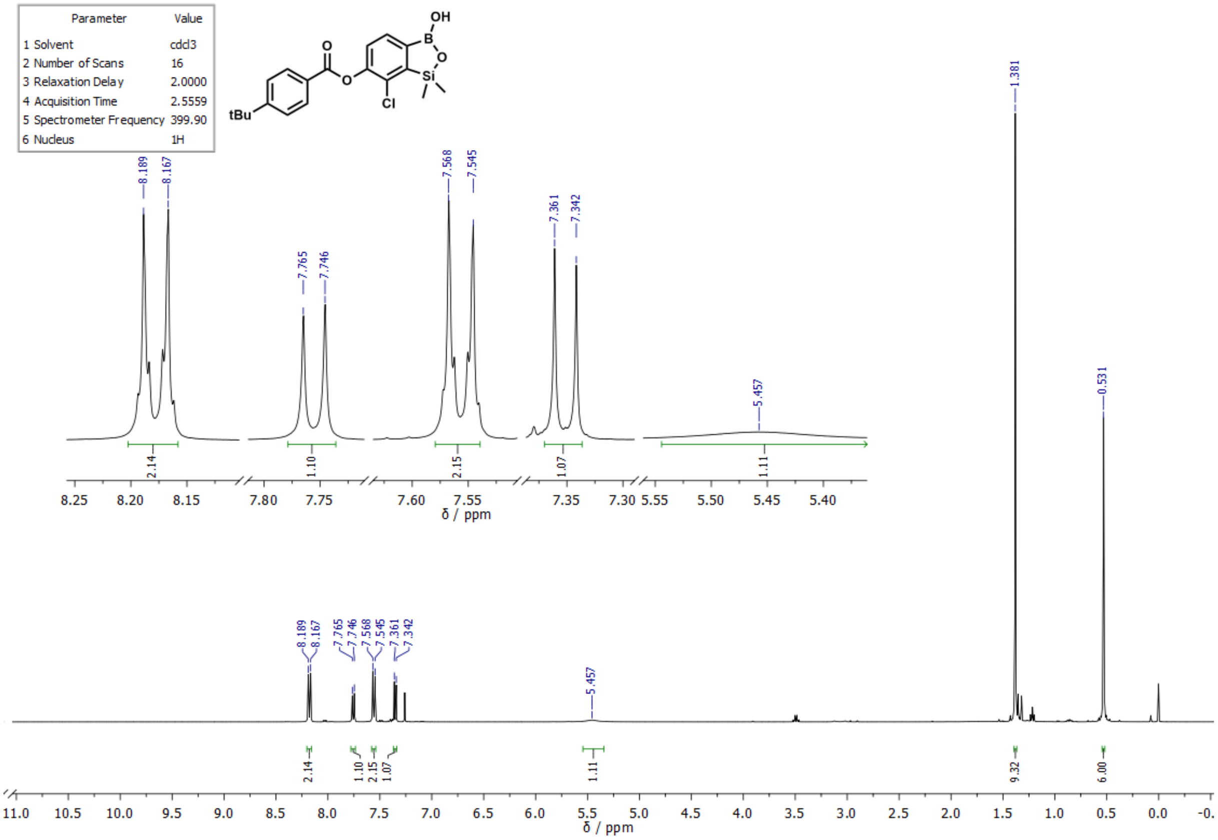

Figure S29. ^{1}H NMR spectrum of **8e** in $CDCl_3$.

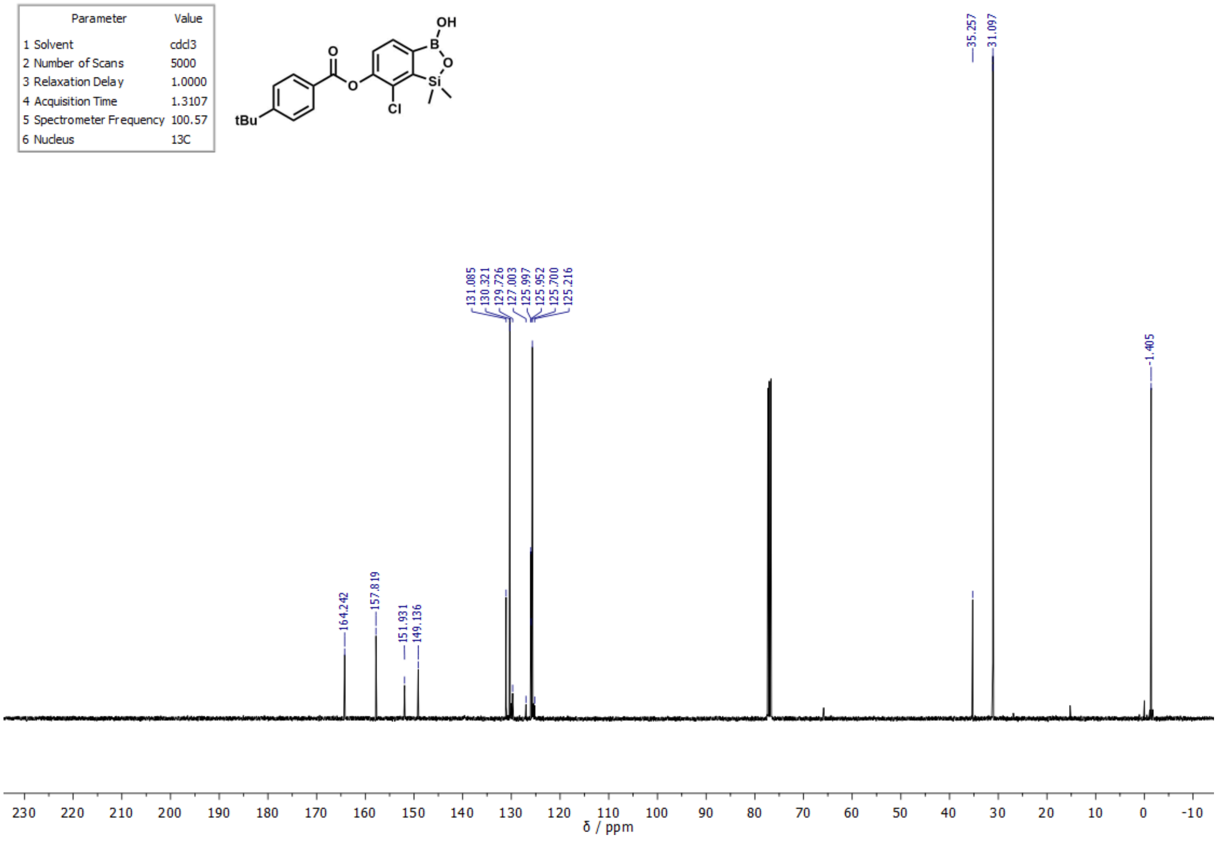

Figure S30. ^{13}C NMR spectrum of **8e** in $CDCl_3$.

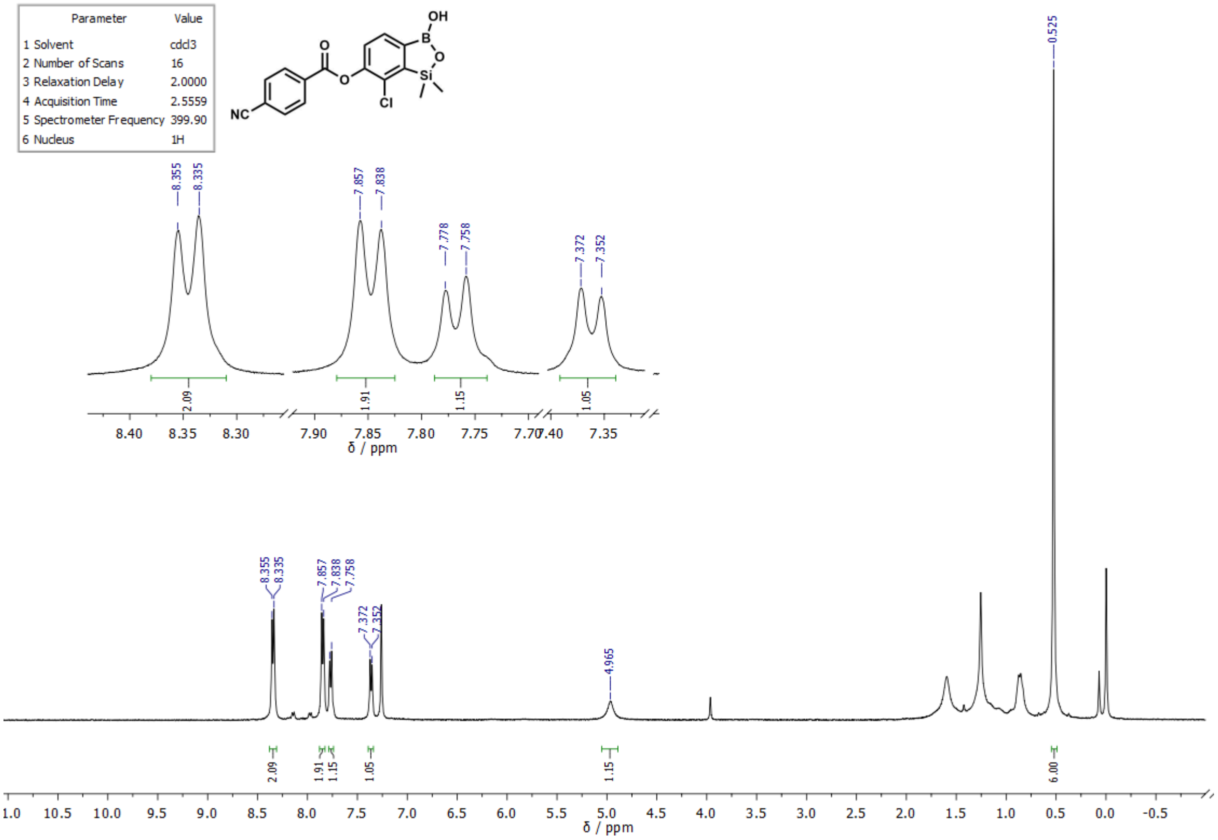

Figure S31. ^{1}H NMR spectrum of **8f** in acetone-d_6.

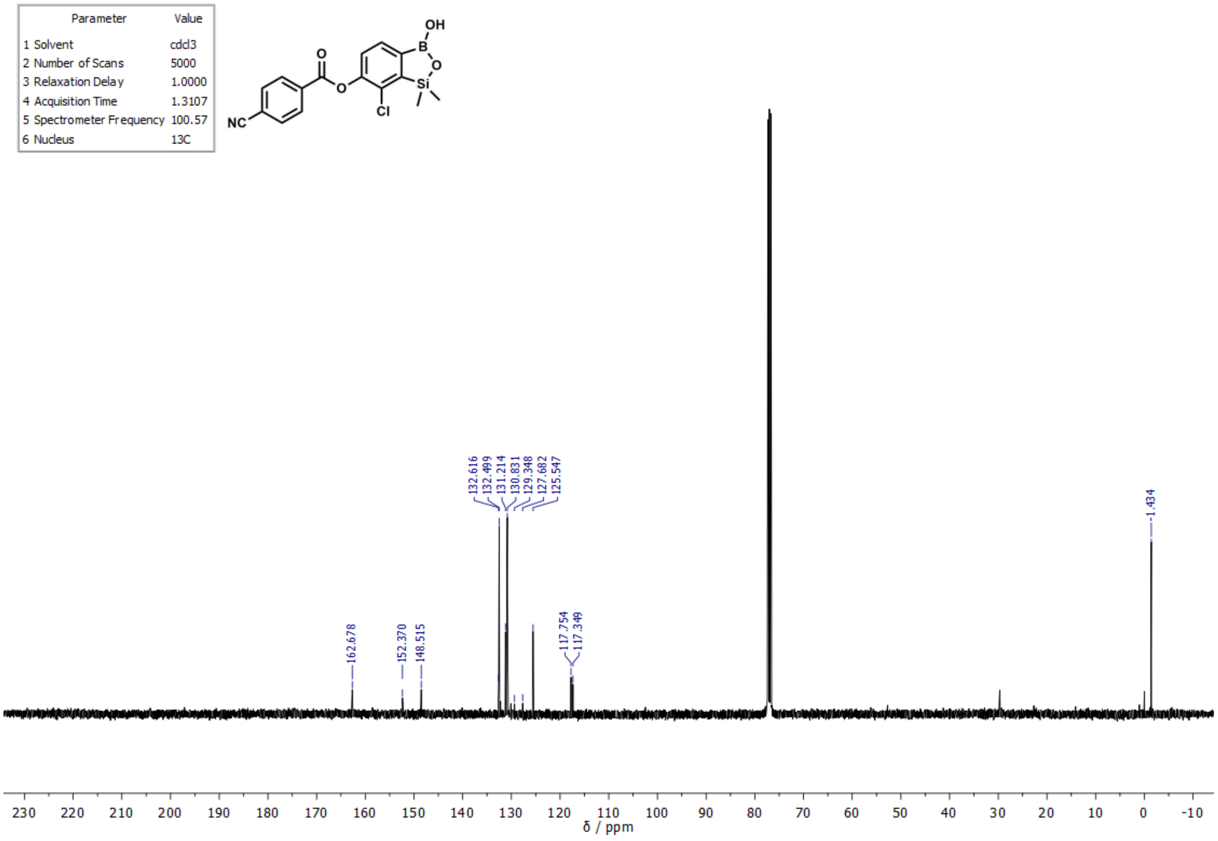

Figure S32. ^{13}C NMR spectrum of **8f** in acetone-d_6.

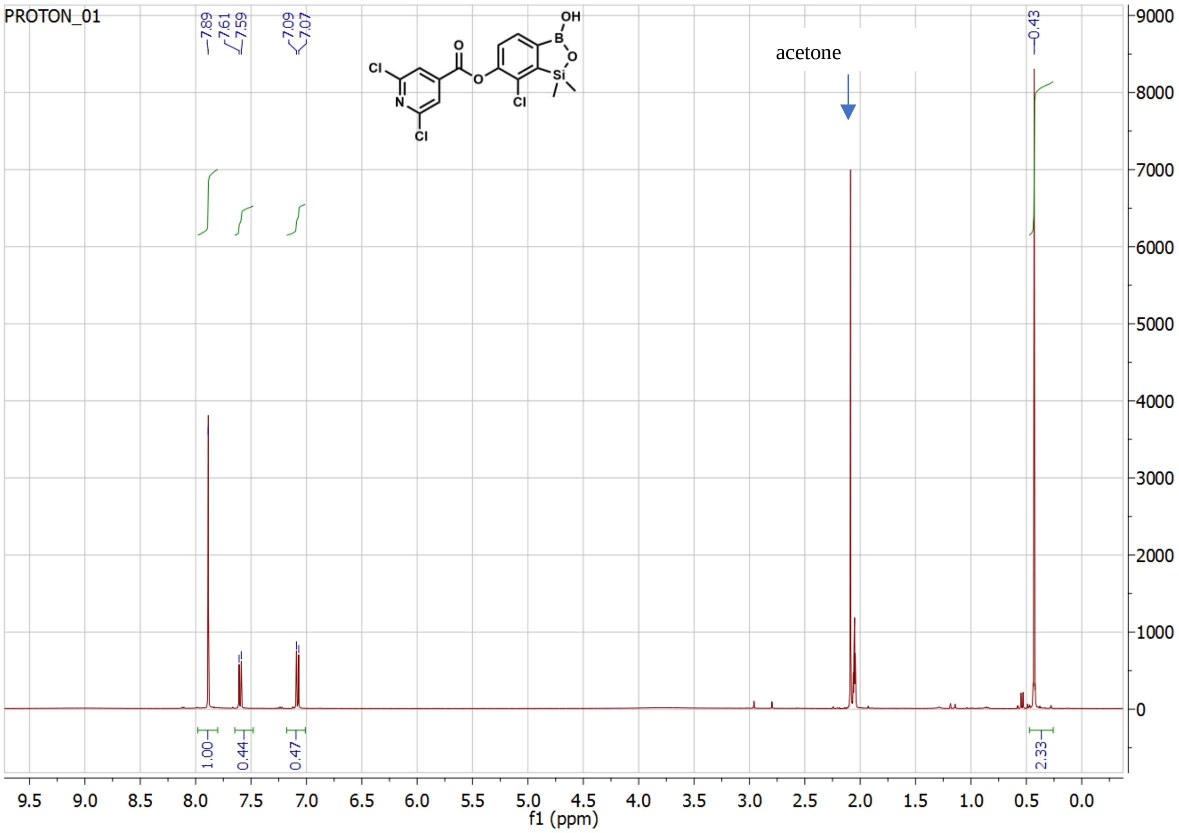

Figure S33. ^{1}H NMR spectrum of **8g** in acetone-d_6.

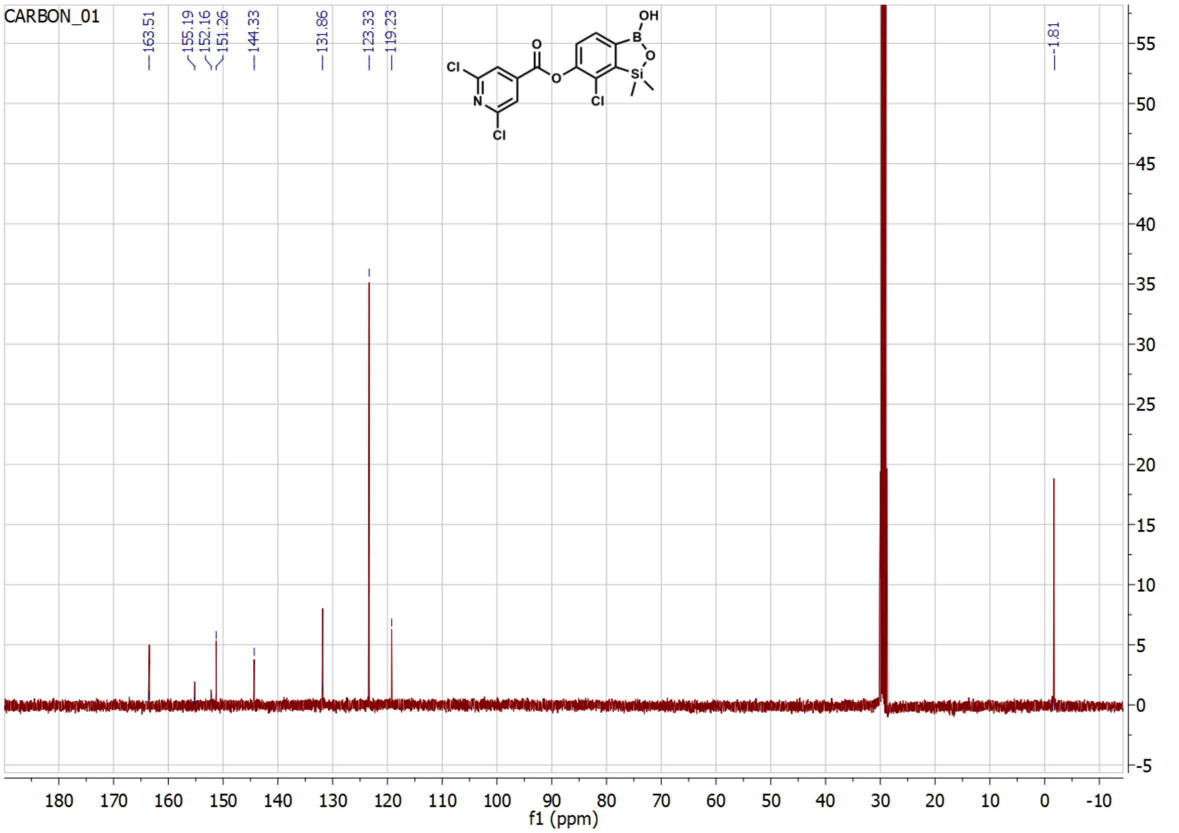

Figure S34. ^{13}C NMR spectrum of **8g** in acetone-d_6.

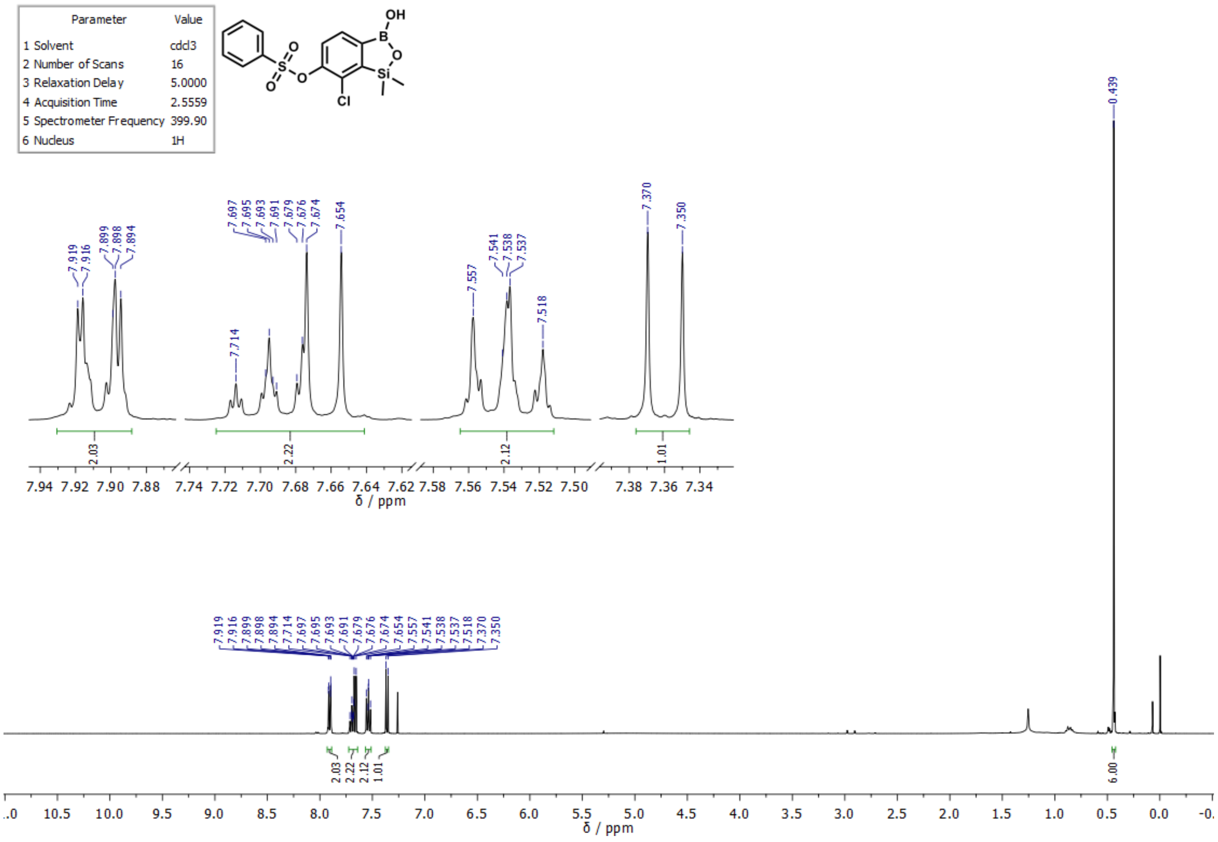

Figure S35. ^{1}H NMR spectrum of **9a** in $CDCl_3$.

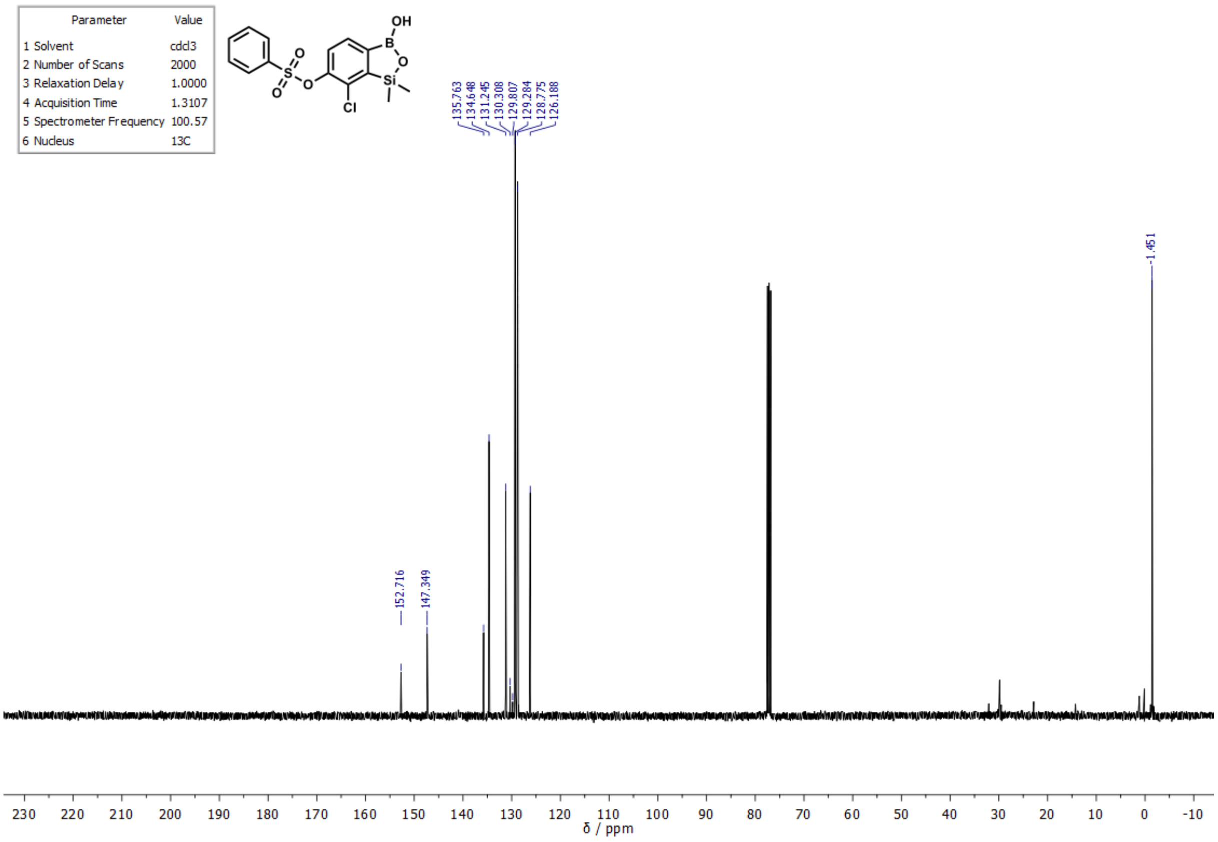

Figure S36. ^{13}C NMR spectrum of **9a** in $CDCl_3$

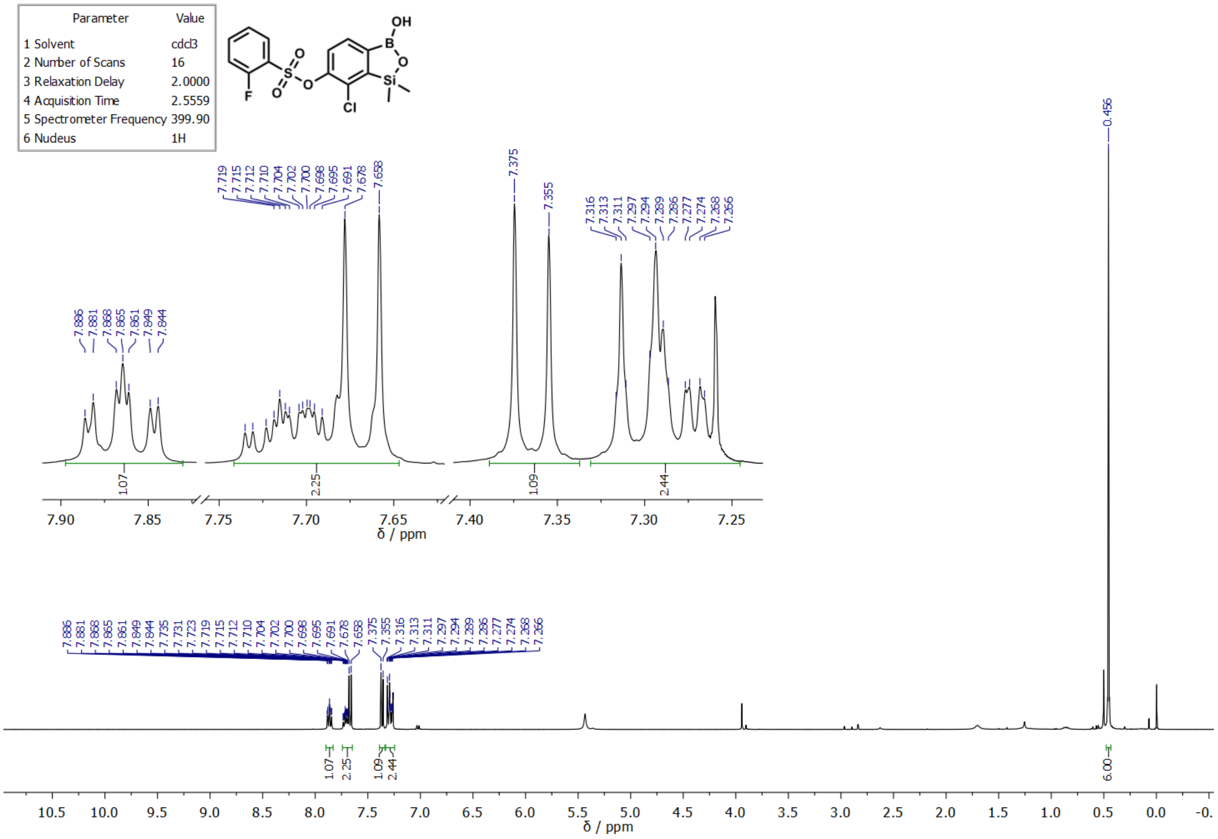

Figure S37. ^{1}H NMR spectrum of **9b** in $CDCl_3$.

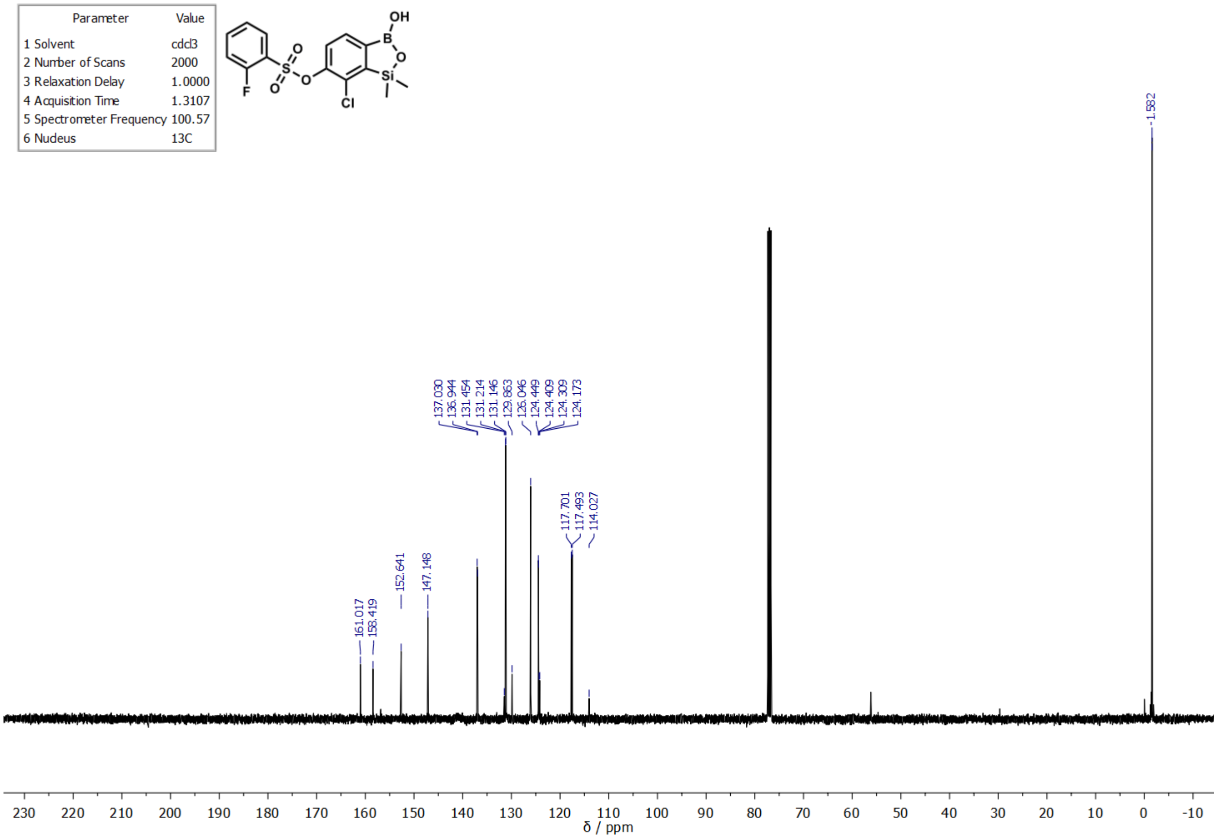

Figure S38. ^{13}C NMR spectrum of **9b** in $CDCl_3$.

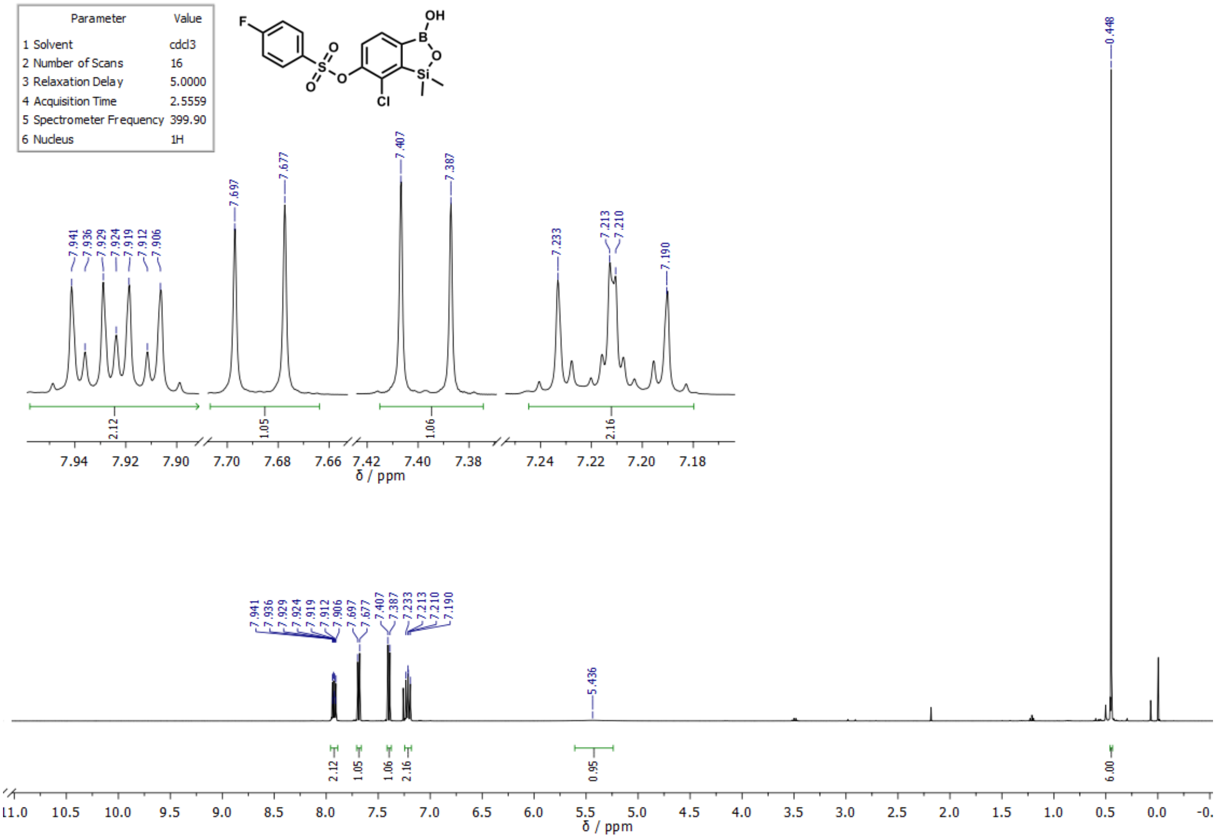

Figure S39. ^{1}H NMR spectrum of **9c** in $CDCl_3$.

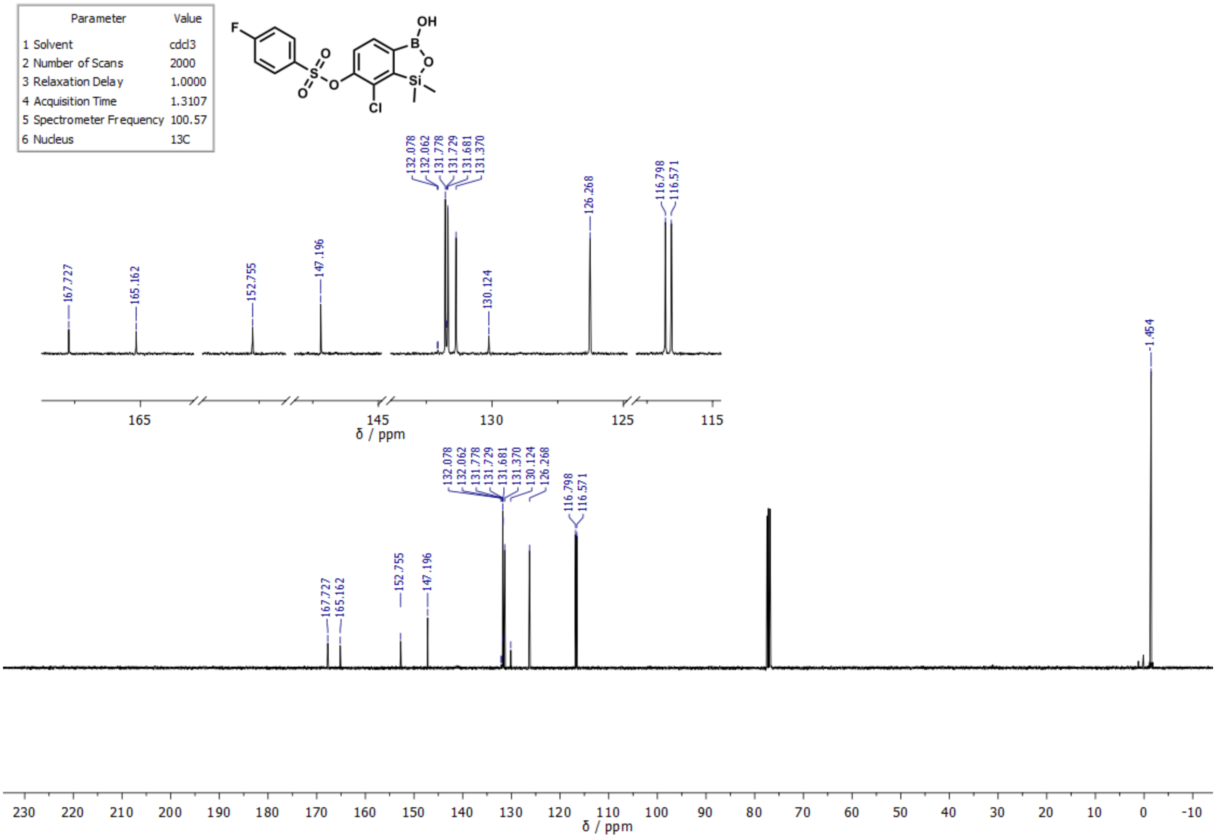

Figure S40. ^{13}C NMR spectrum of **9c** in $CDCl_3$.

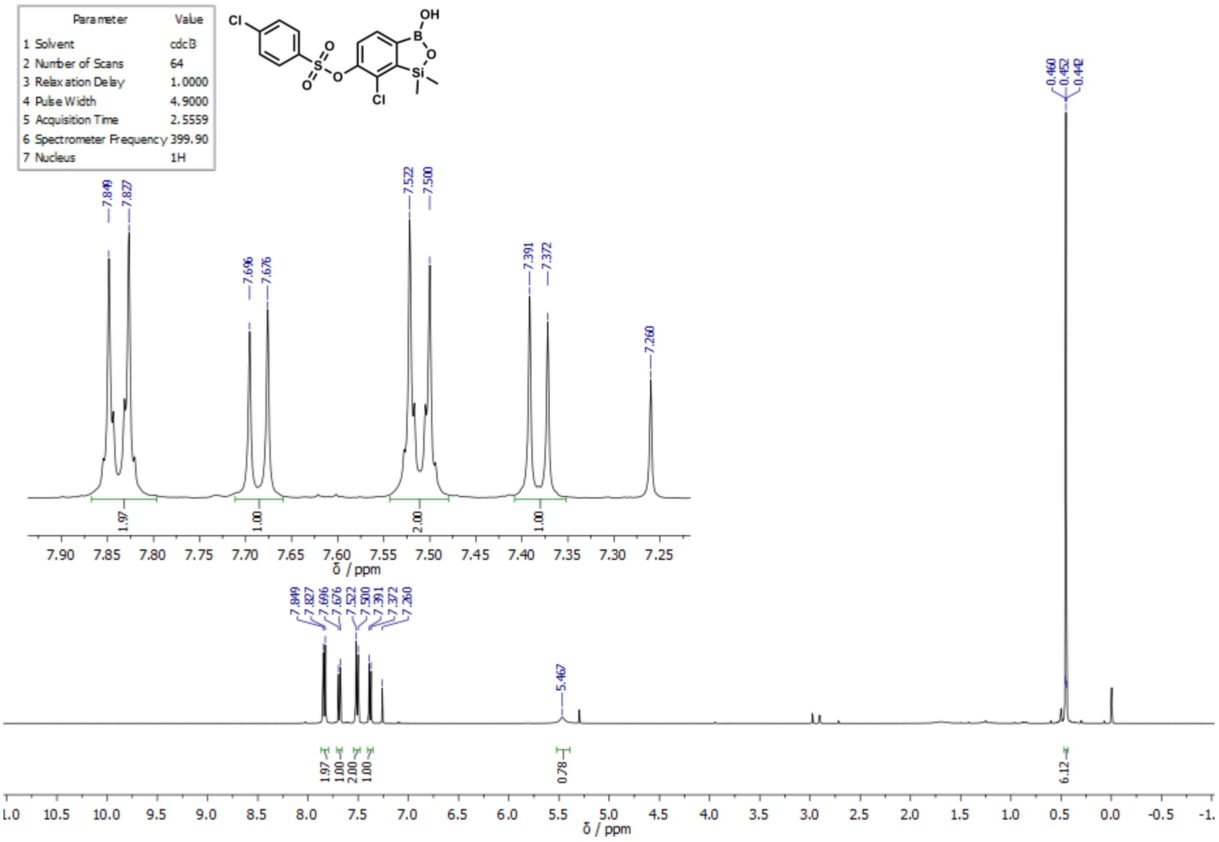

Figure S41. ^{1}H NMR spectrum of **9d** in $CDCl_3$.

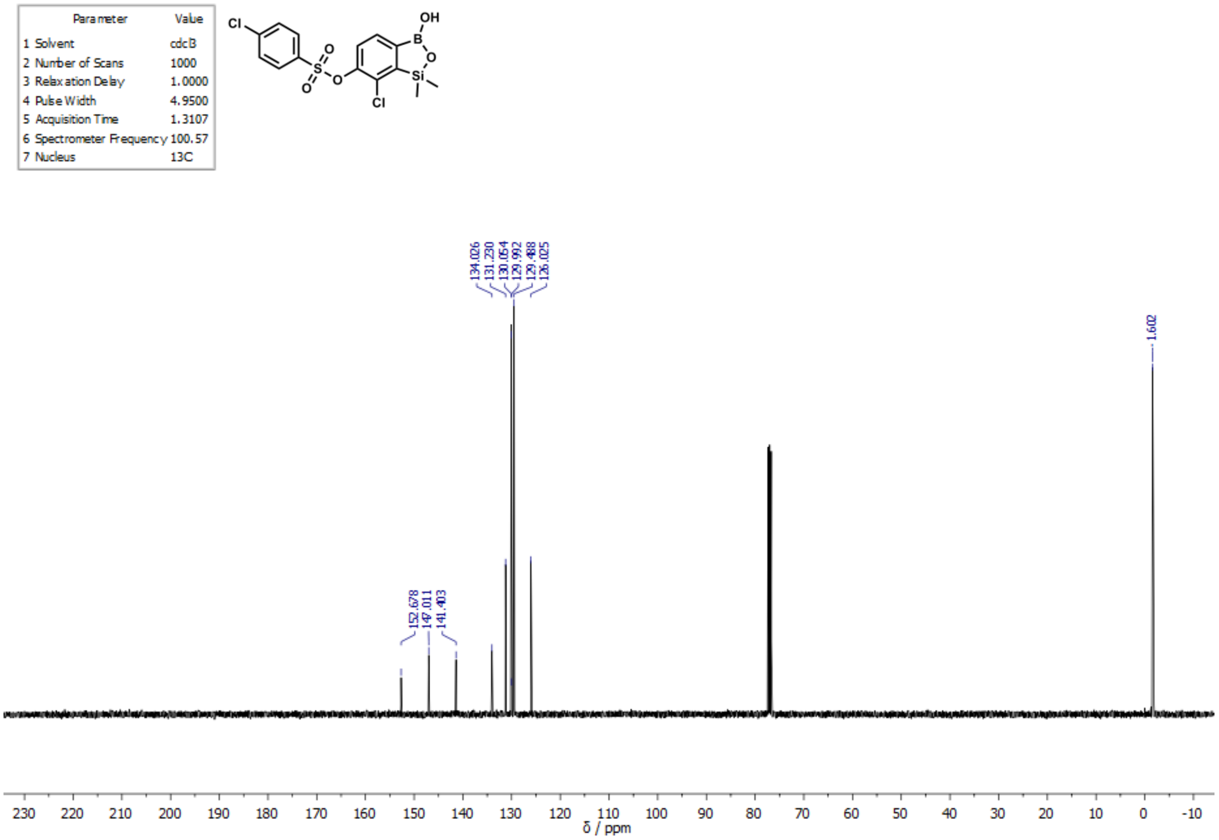

Figure S42. ^{13}C NMR spectrum of **9d** in $CDCl_3$.

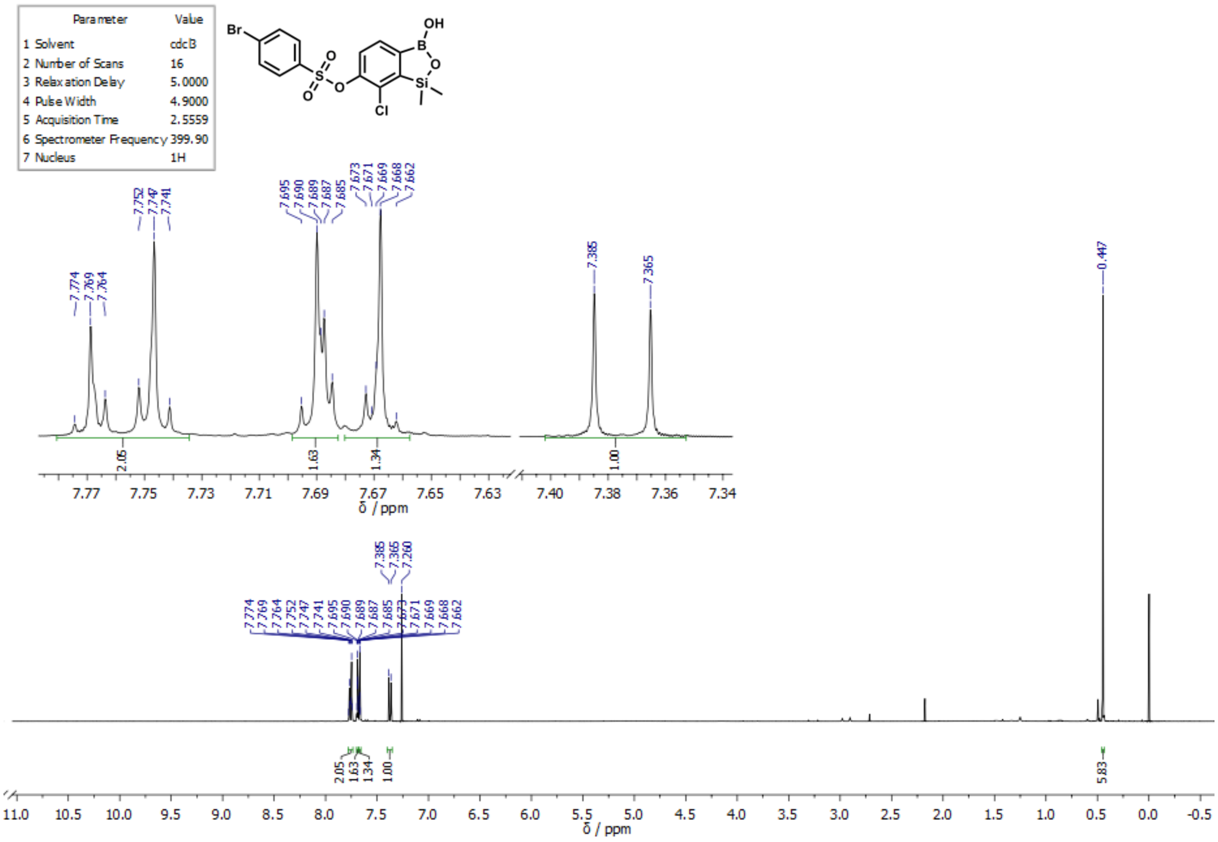

Figure S43. ^{1}H NMR spectrum of **9e** in $CDCl_3$.

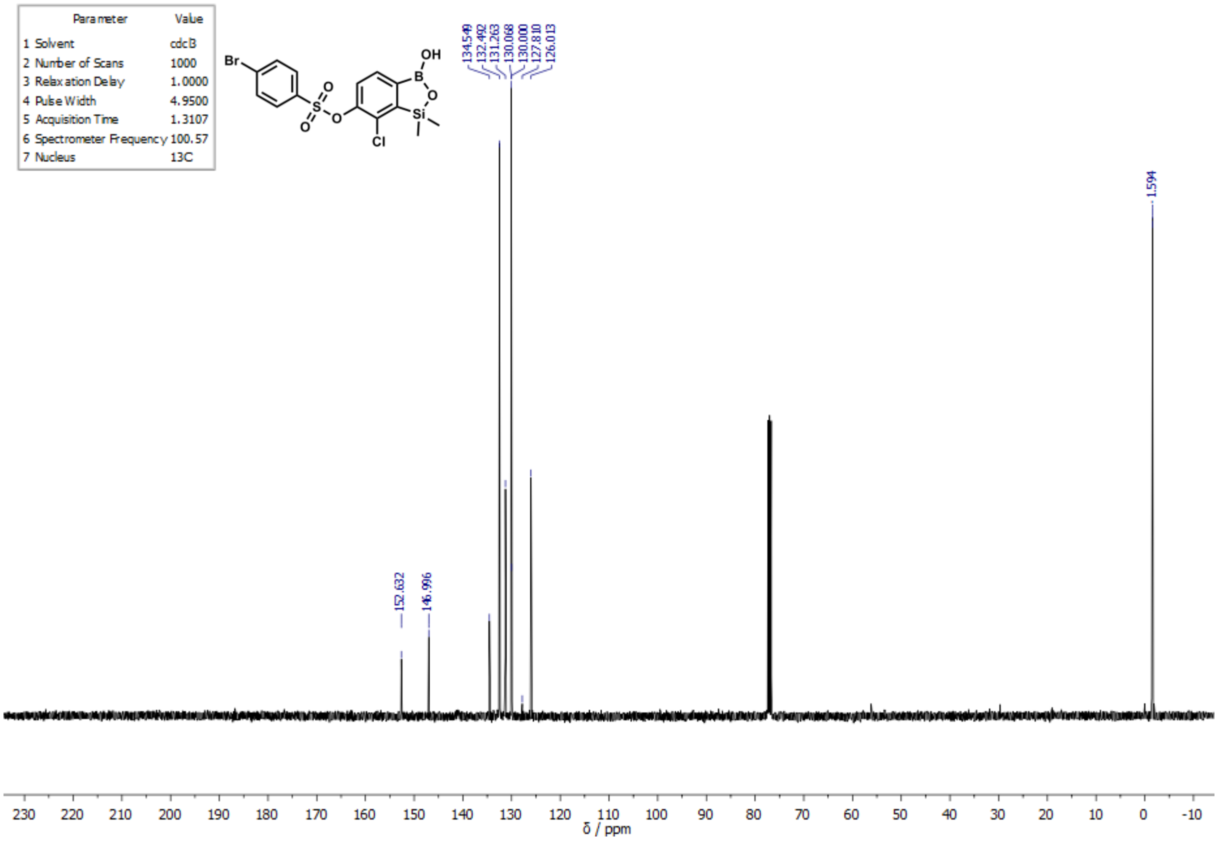

Figure S44. ^{13}C NMR spectrum of **9e** in $CDCl_3$.

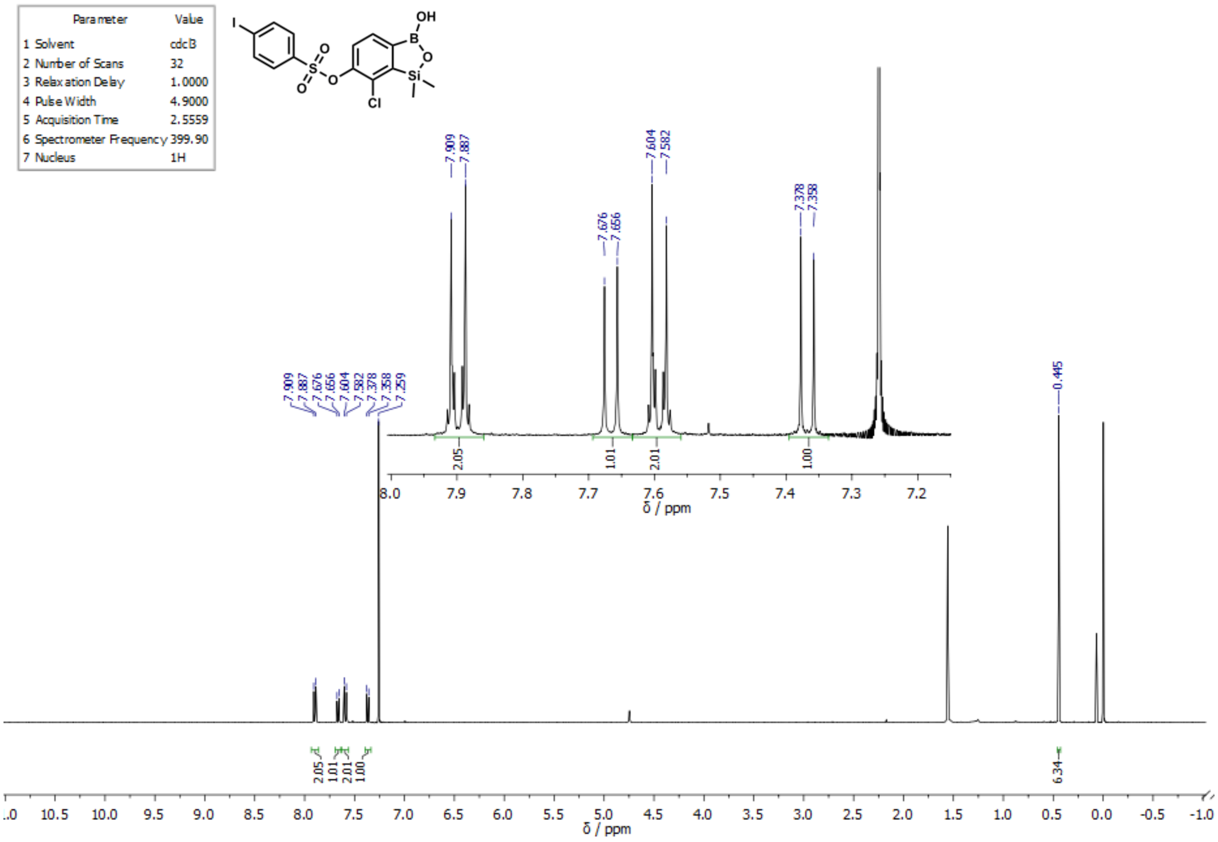

Figure S45. ^{1}H NMR spectrum of **9f** in $CDCl_3$.

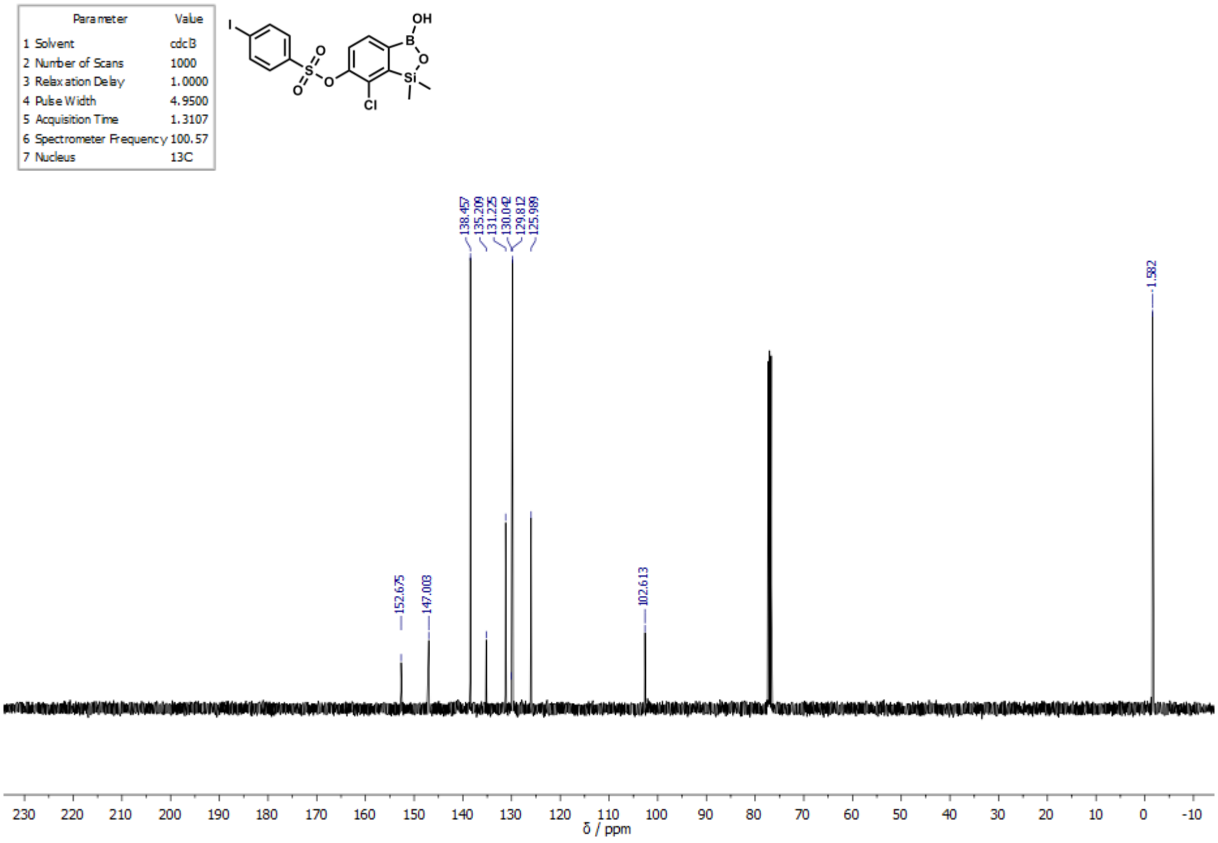

Figure S46. ^{13}C NMR spectrum of **9f** in $CDCl_3$.

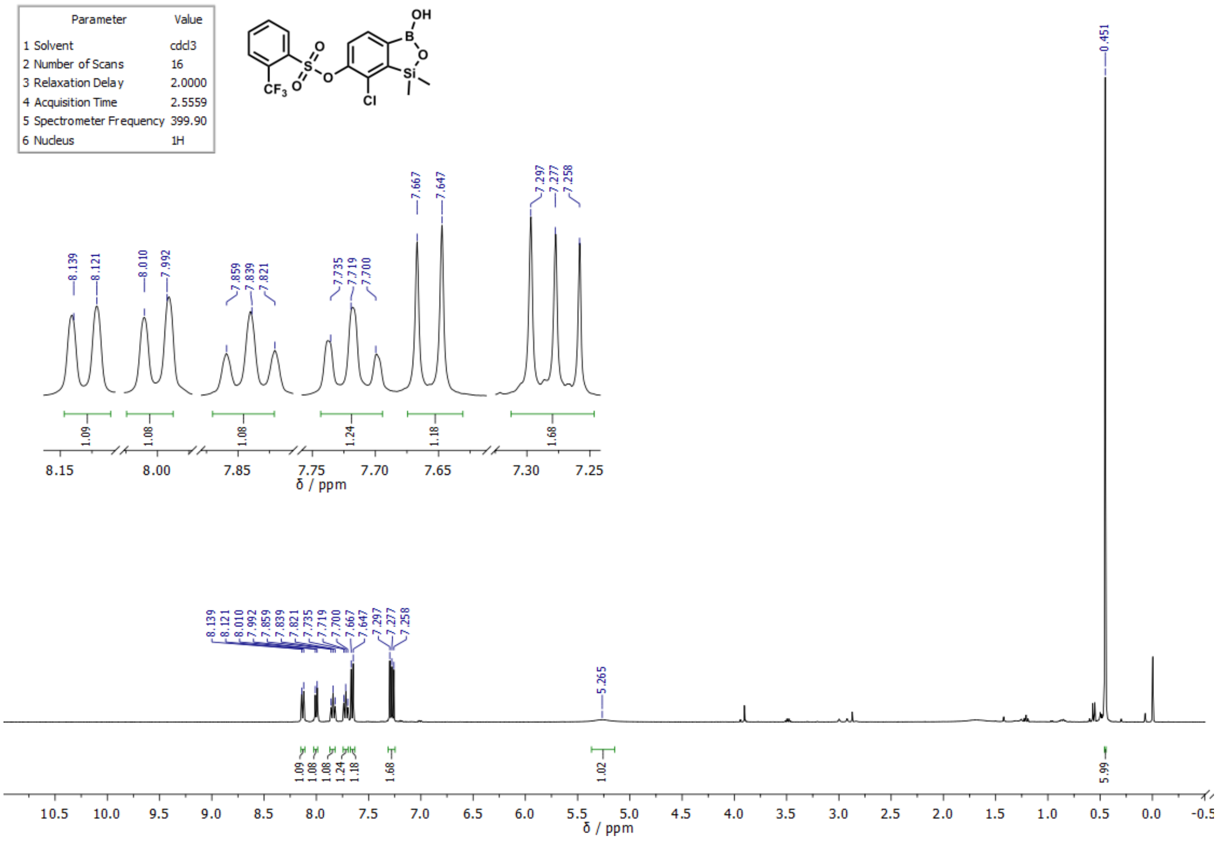

Figure S47. ^{1}H NMR spectrum of **9g** in $CDCl_3$.

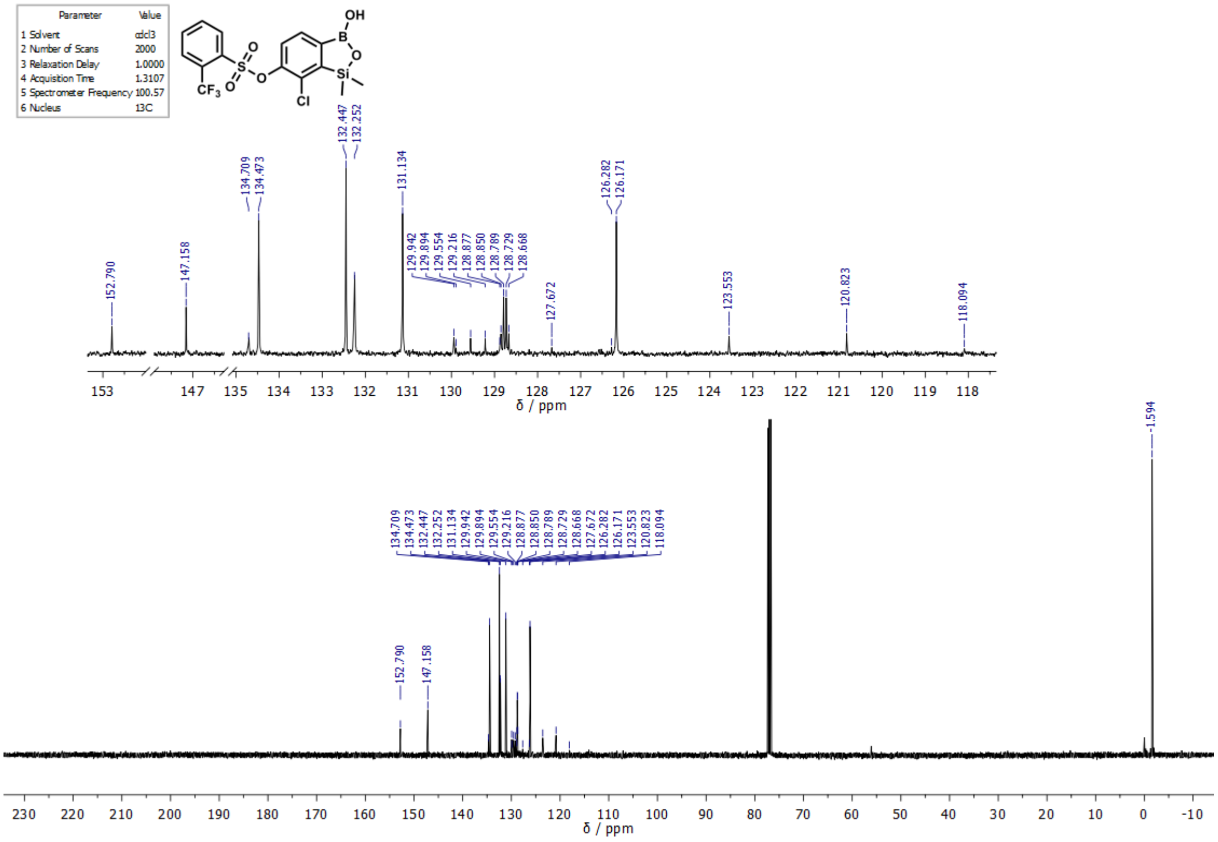

Figure S48. ^{13}C NMR spectrum of 9g in $CDCl_3$.

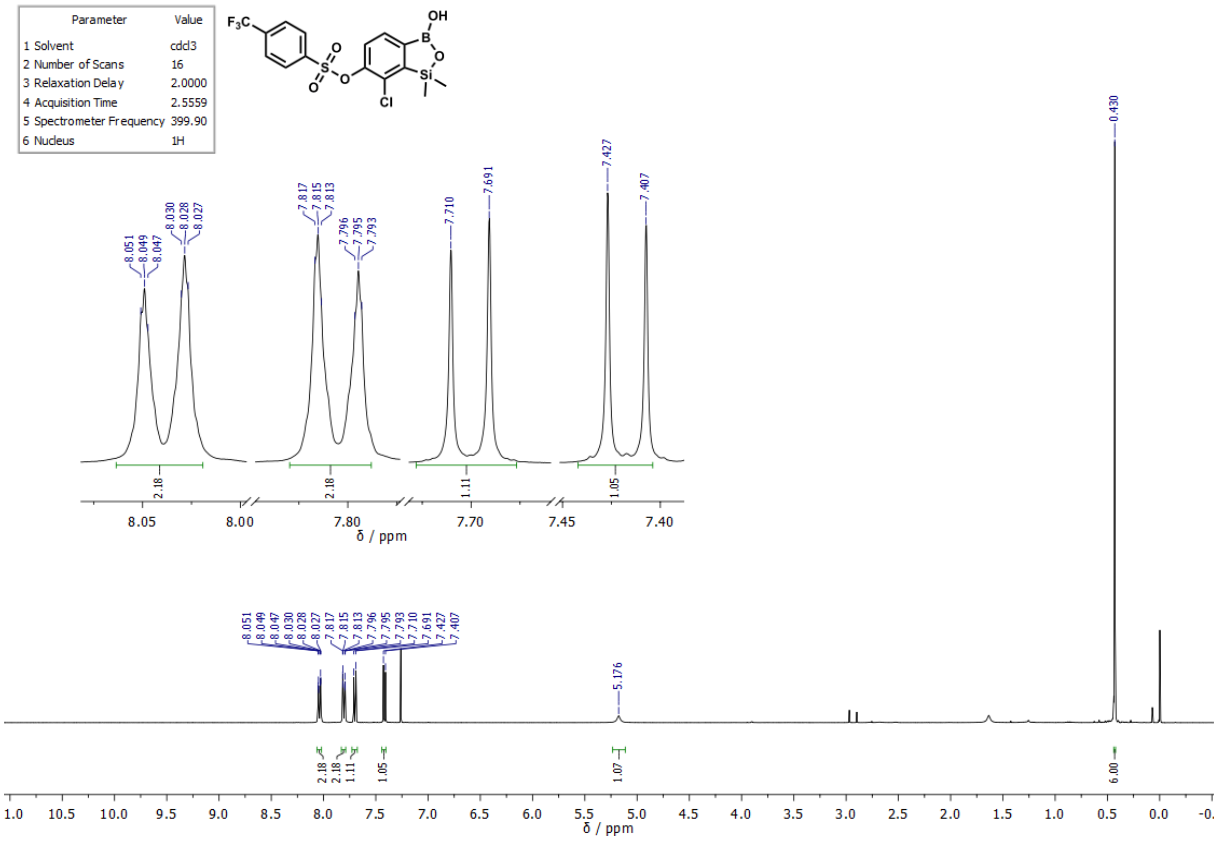

Figure S49. ^{1}H NMR spectrum of **9h** in $CDCl_3$.

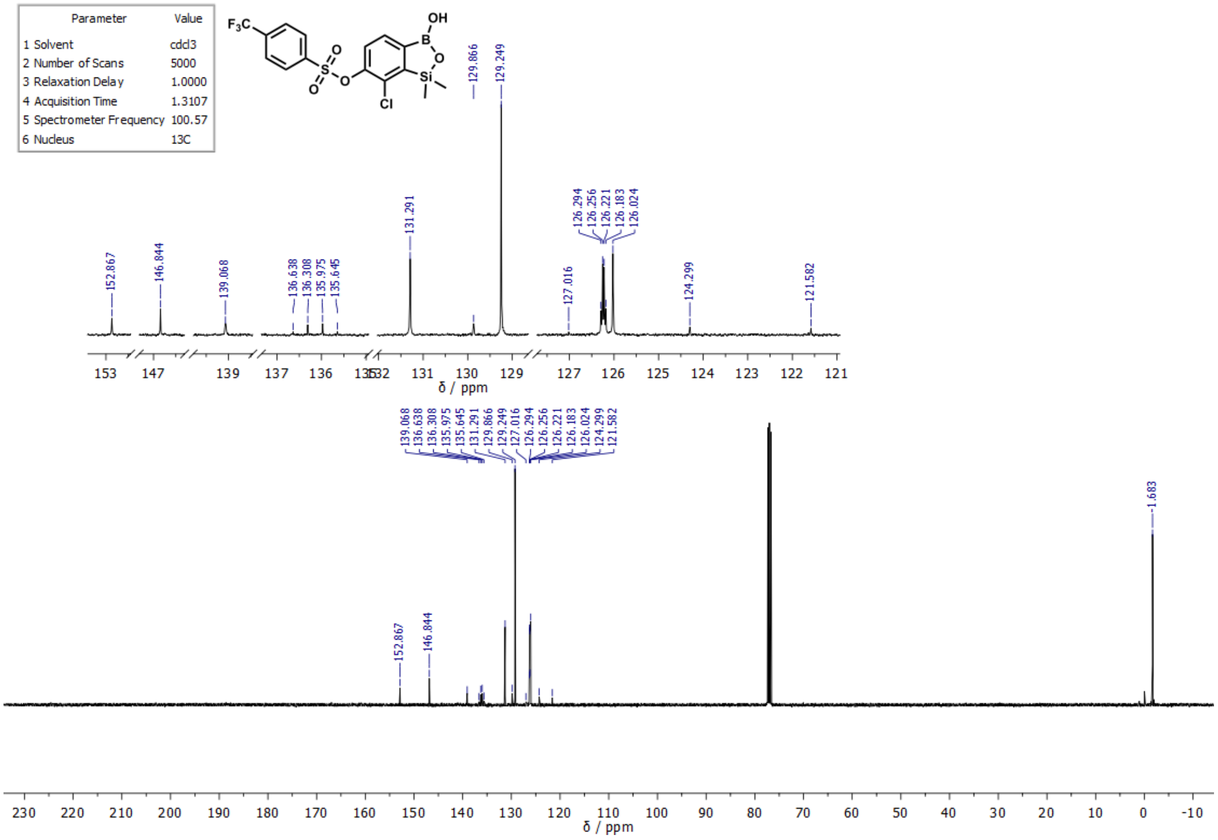

Figure S50.^{13}C NMR spectrum of **9h** in $CDCl_3$.

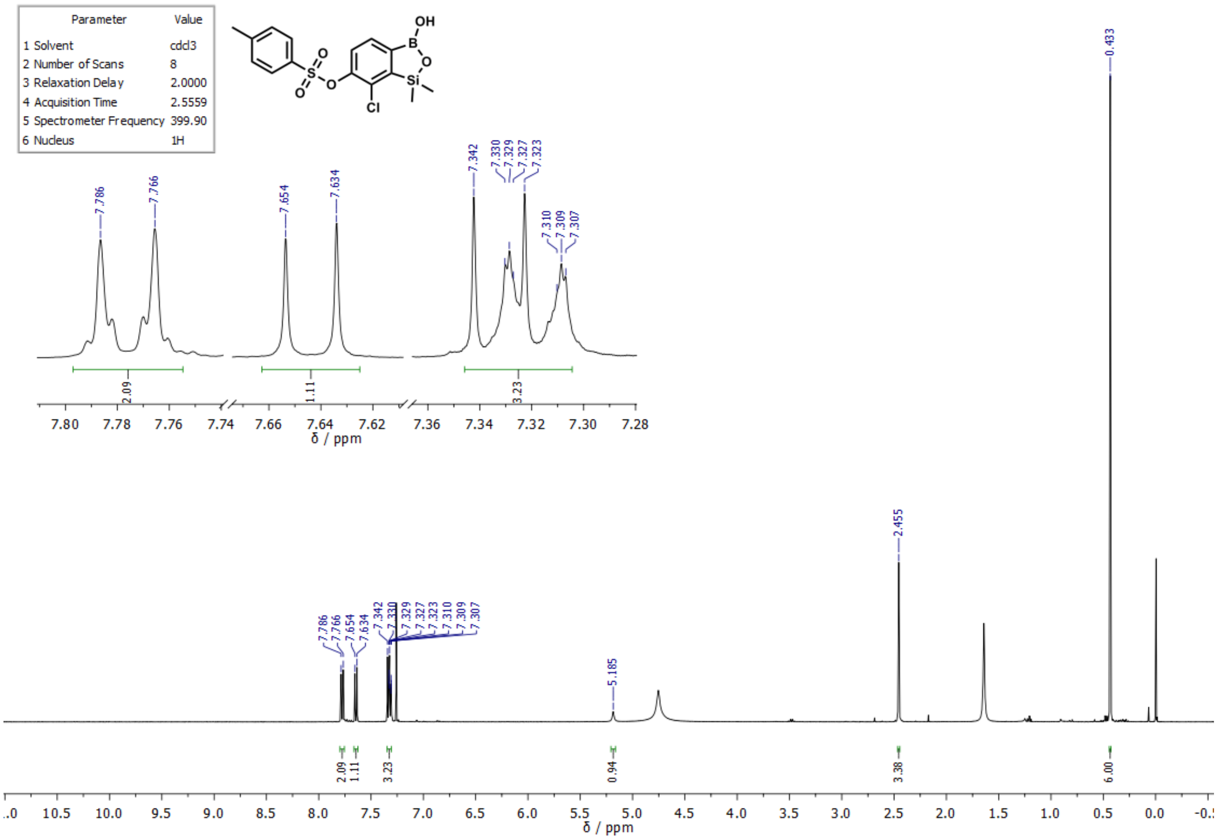

Figure S51. ^{1}H NMR spectrum of **9i** in $CDCl_3$.

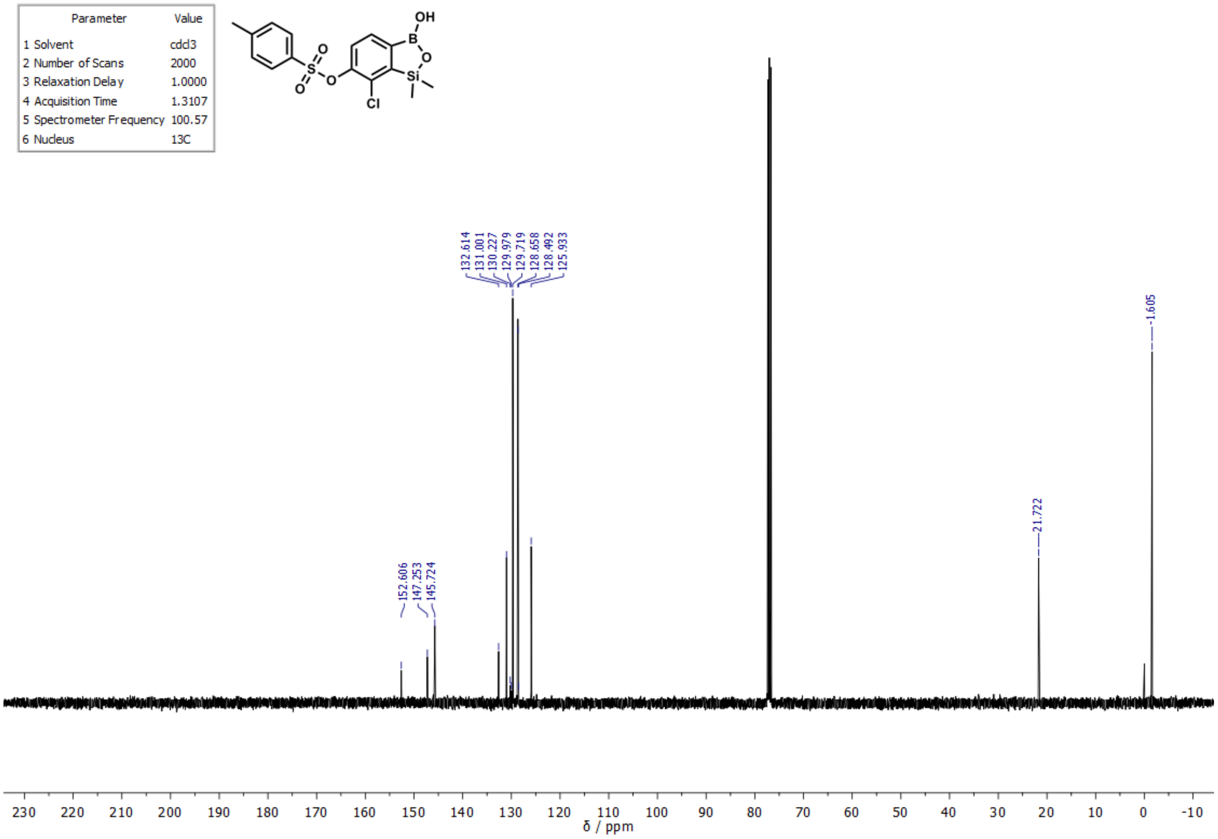

Figure S52. ^{13}C NMR spectrum of **9i** in $CDCl_3$.

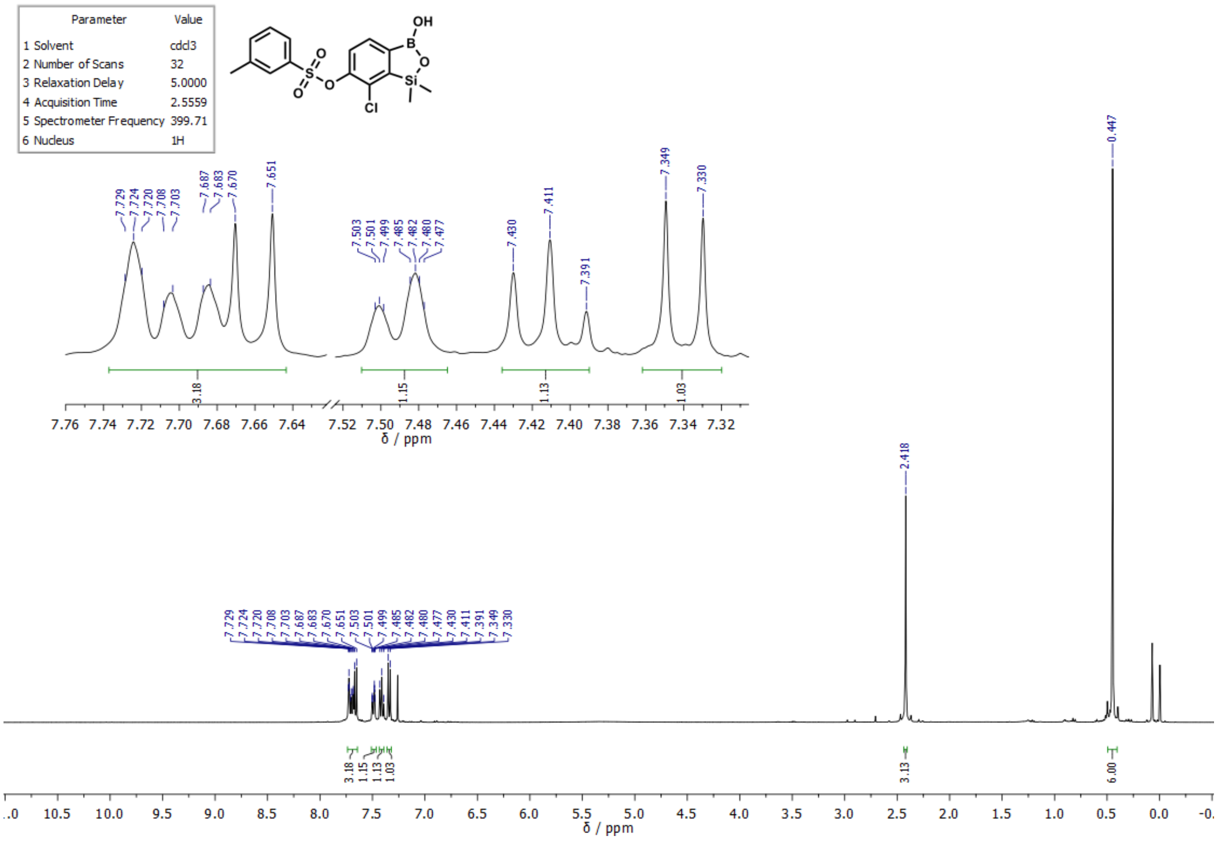

Figure S53. ^{1}H NMR spectrum of **9j** in $CDCl_3$.

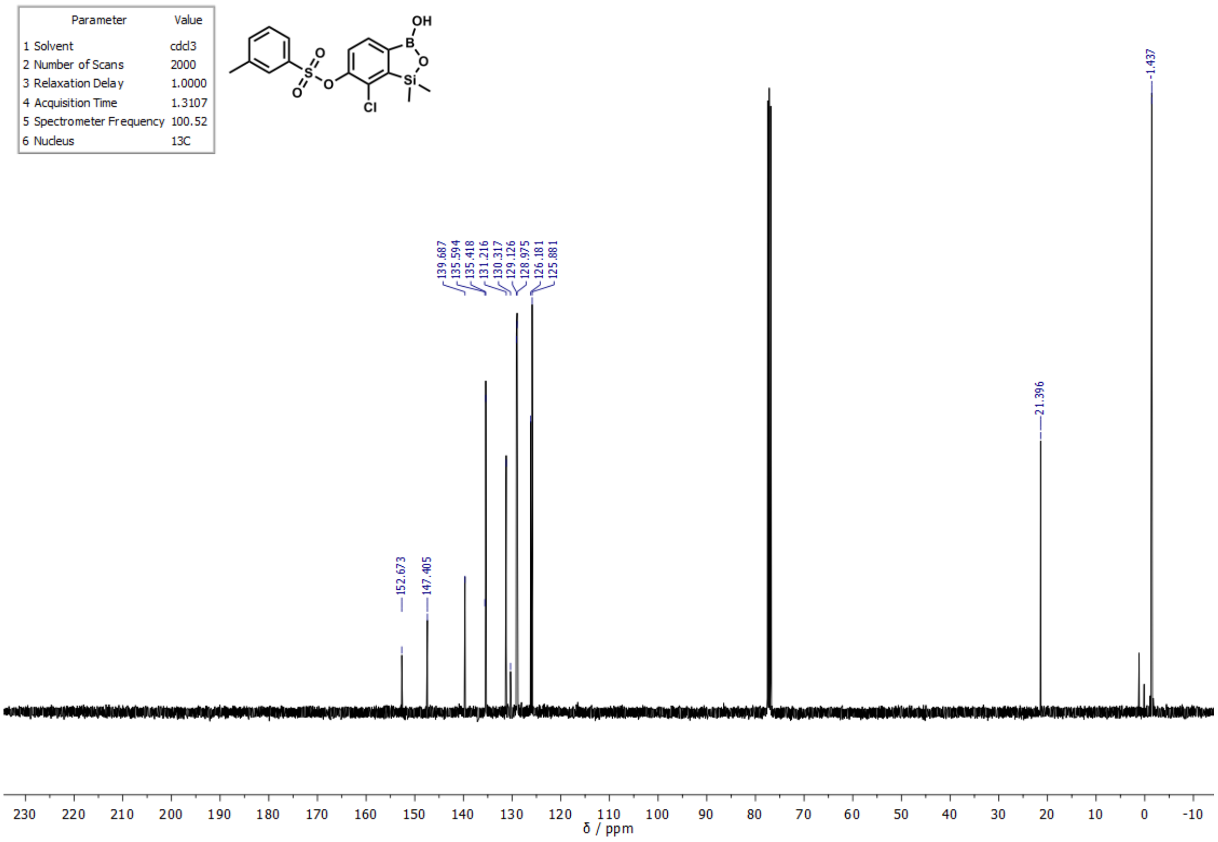

Figure S54. ^{13}C NMR spectrum of **9j** in $CDCl_3$.

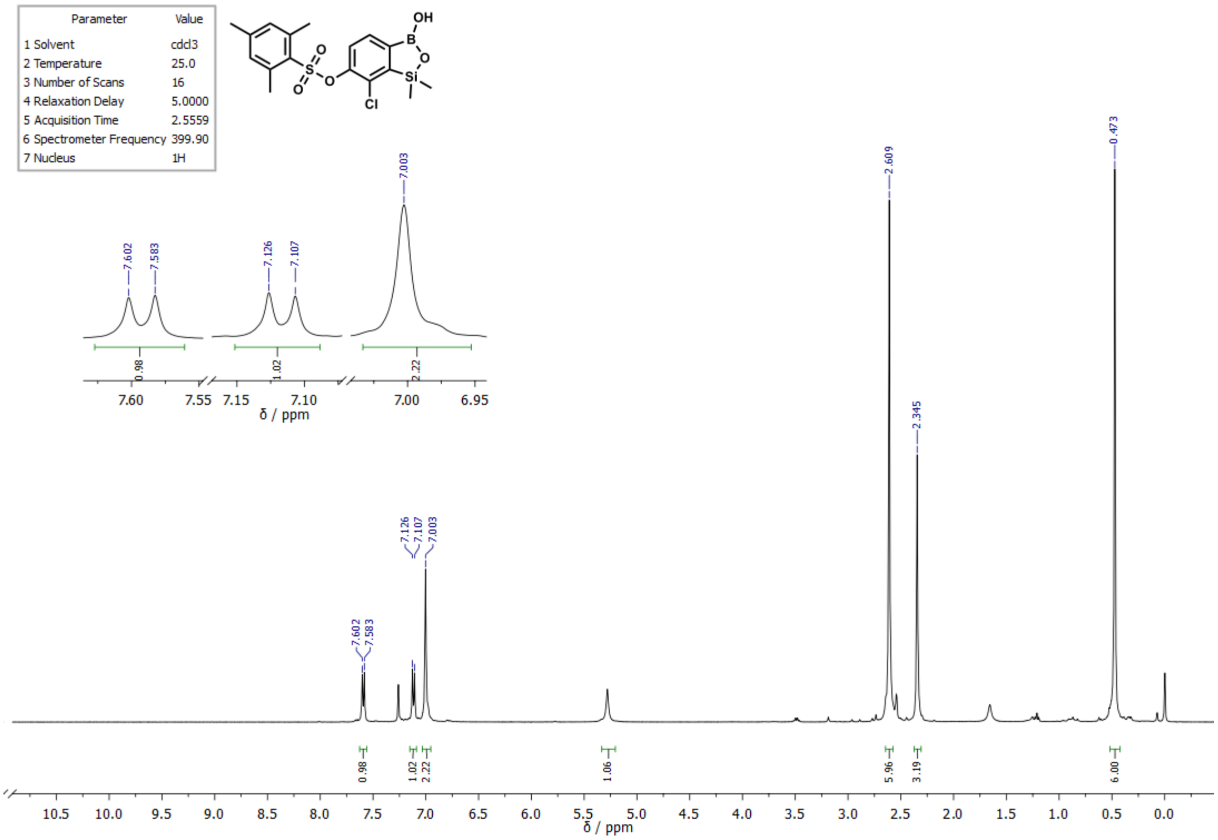

Figure S55. ^{1}H NMR spectrum of **9k** in $CDCl_3$.

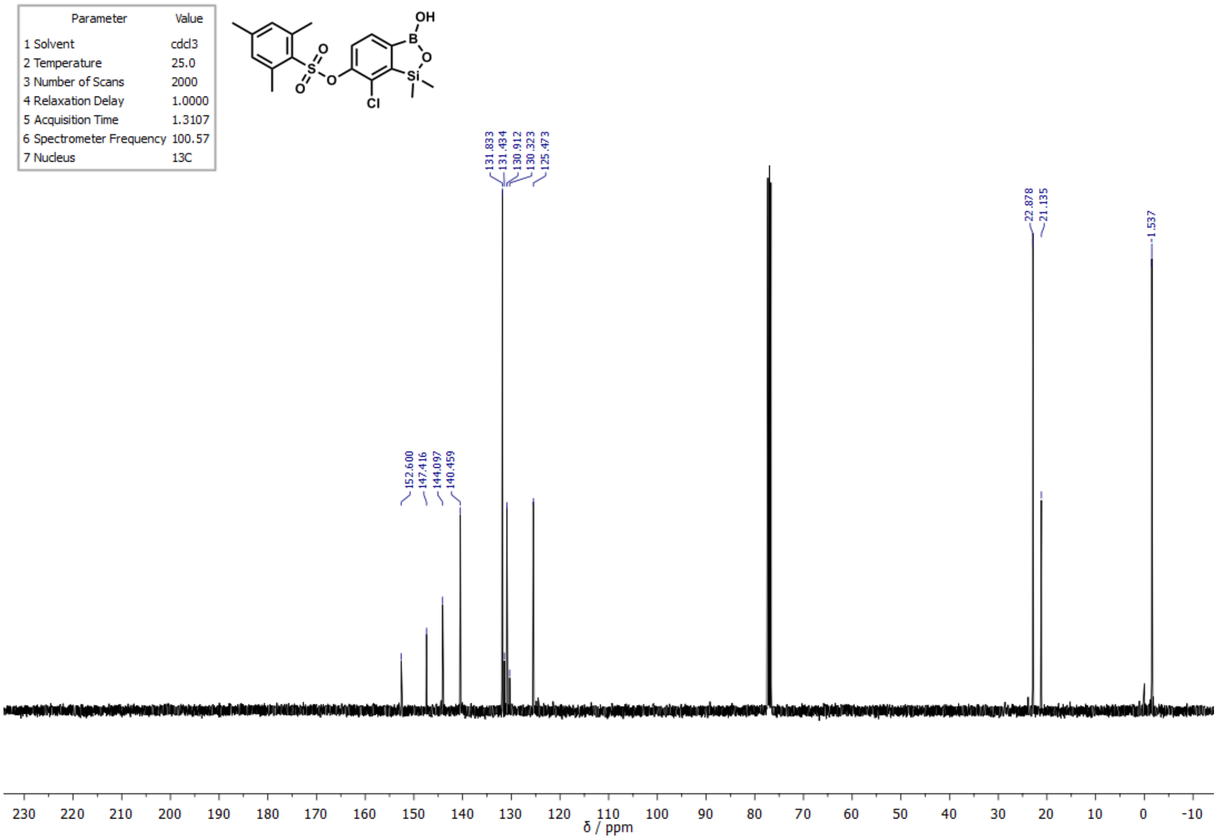

Figure S56. ^{13}C NMR spectrum of **9k** in $CDCl_3$.

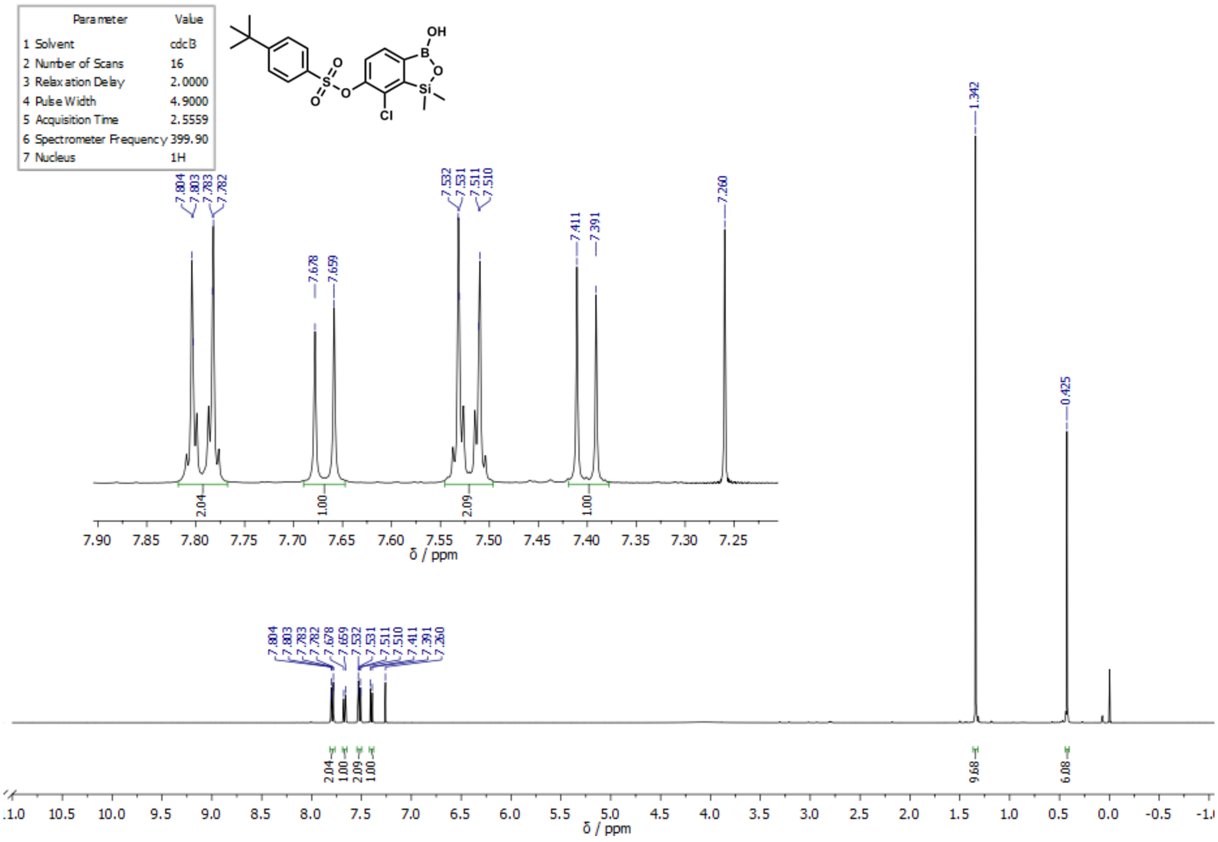

Figure S57. ^{1}H NMR spectrum of **9l** in $CDCl_3$.

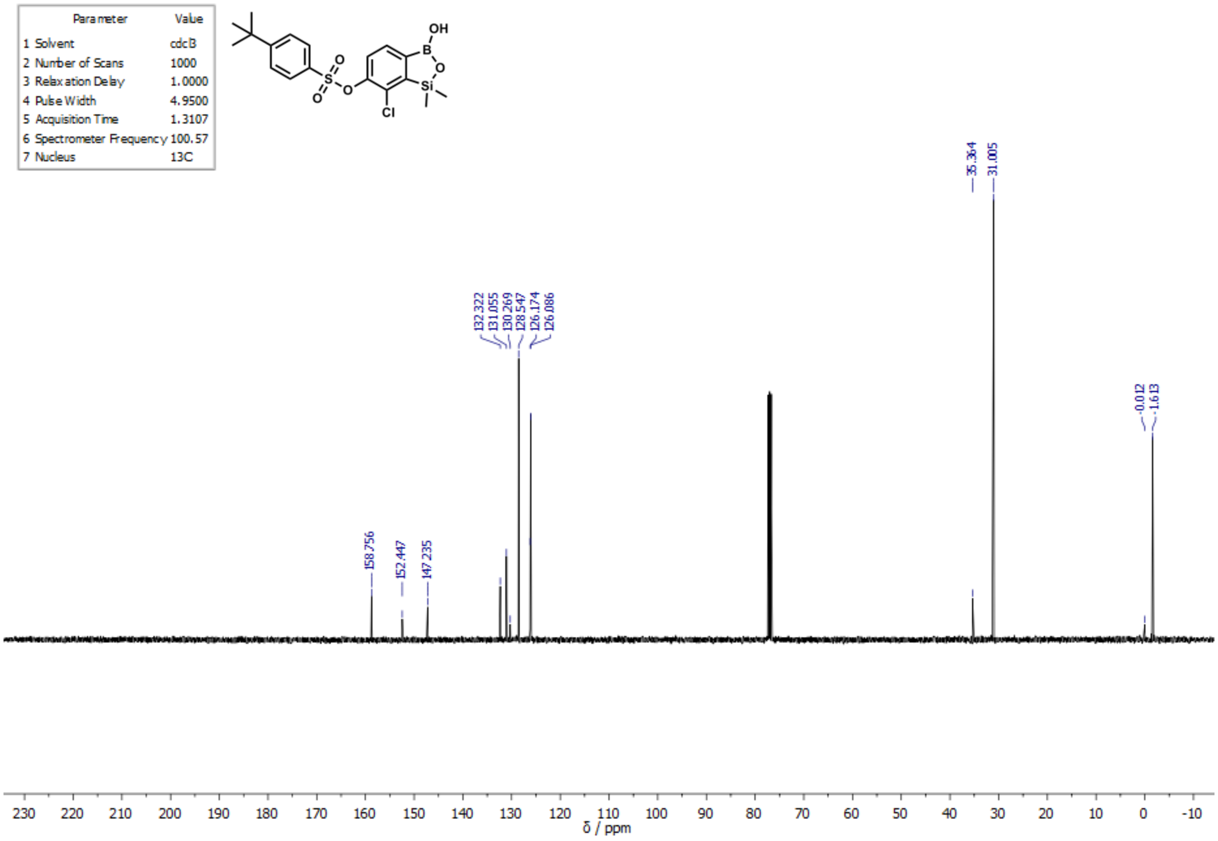

Figure S58. ^{13}C NMR spectrum of **9l** in $CDCl_3$

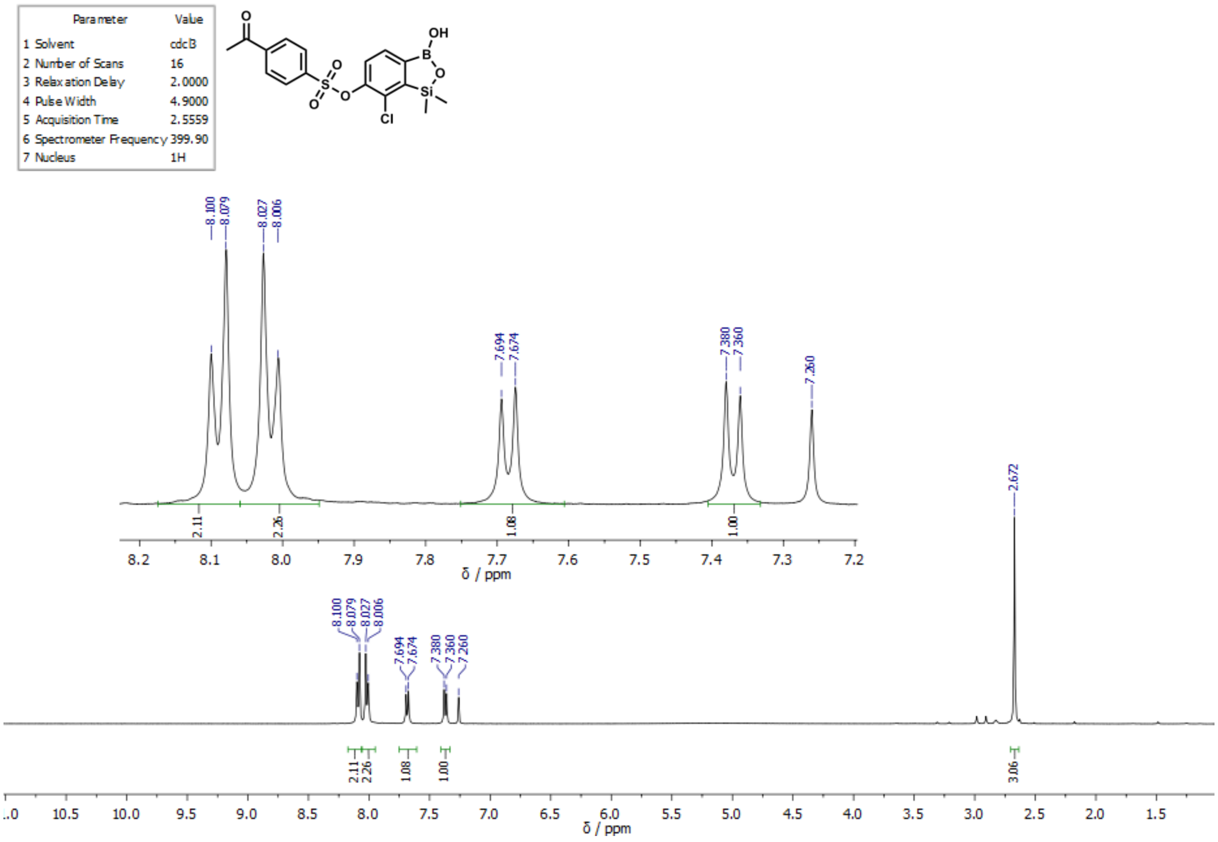

Figure S59. ^{1}H NMR spectrum of **9m** in $CDCl_3$.

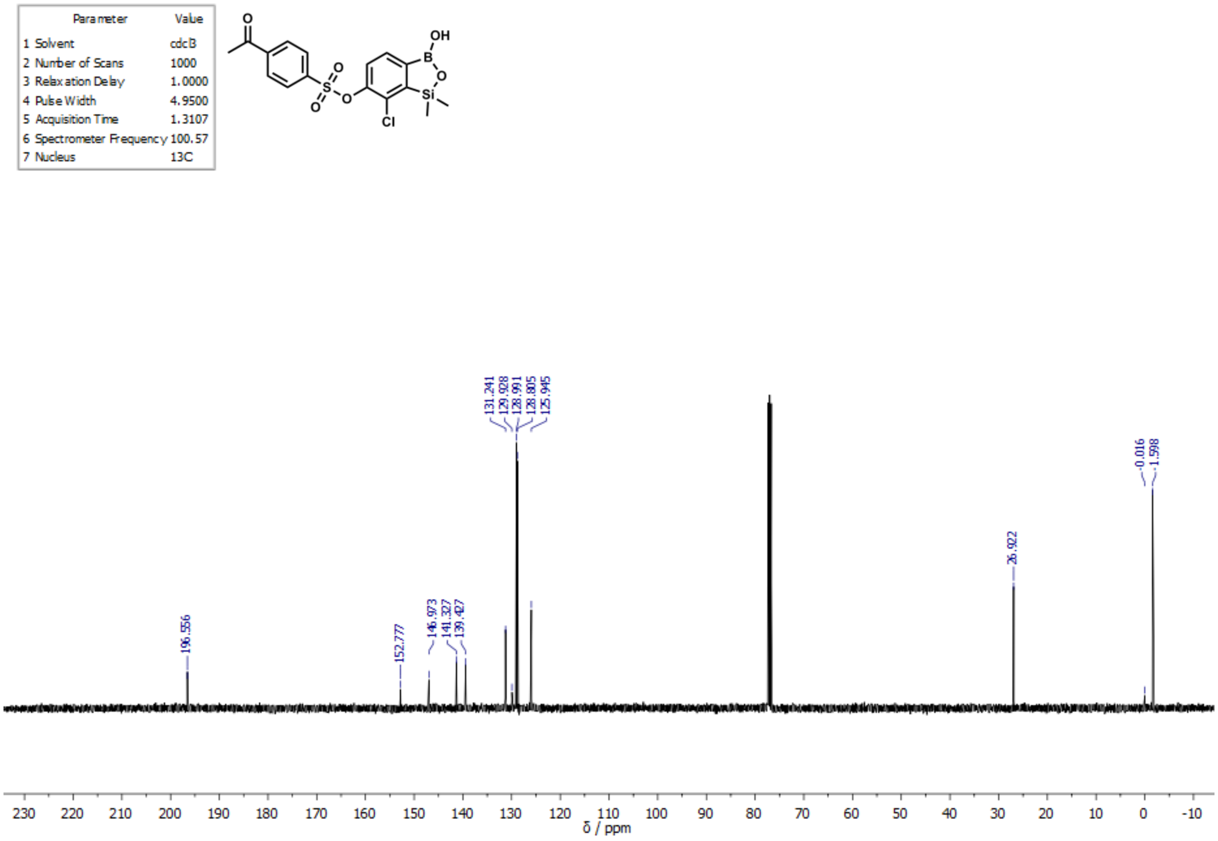

Figure S60. ^{13}C NMR spectrum of **9m** in $CDCl_3$

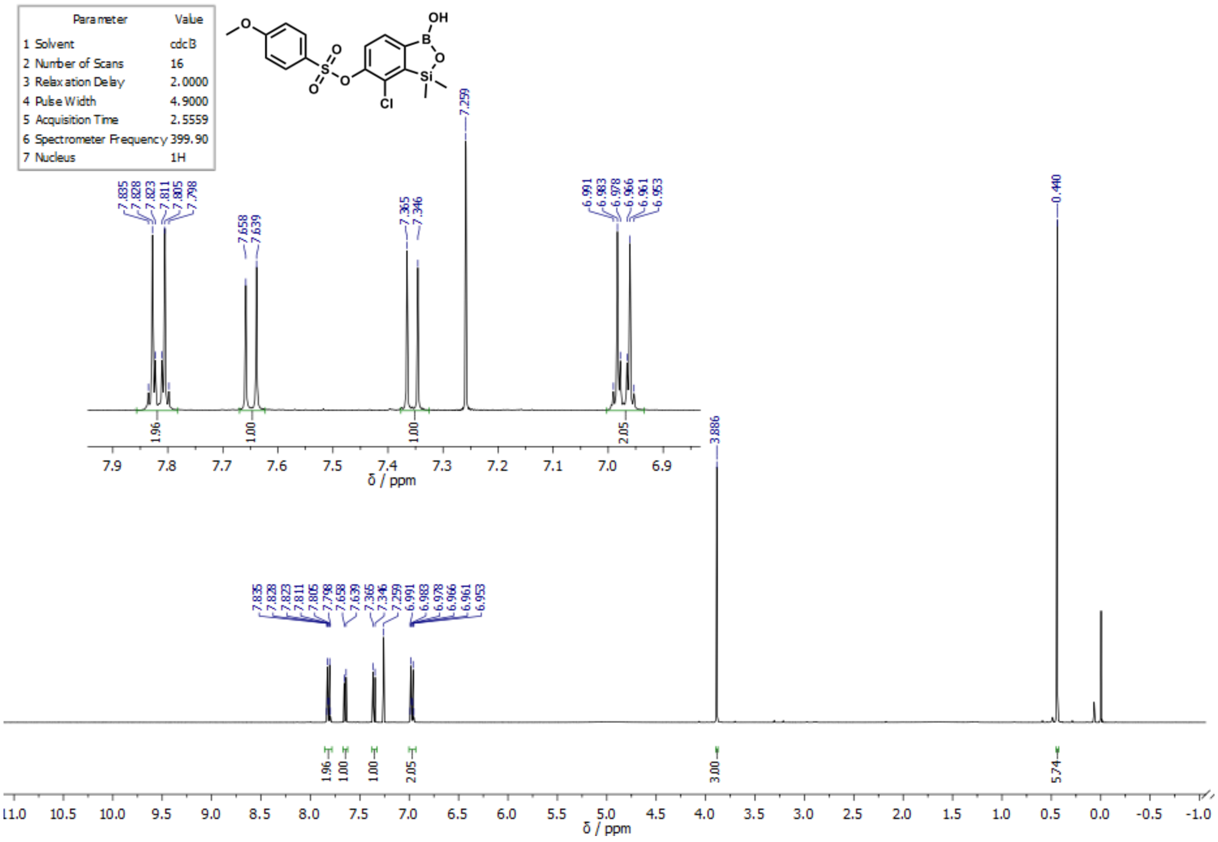

Figure S61. ^{1}H NMR spectrum of **9n** in $CDCl_3$.

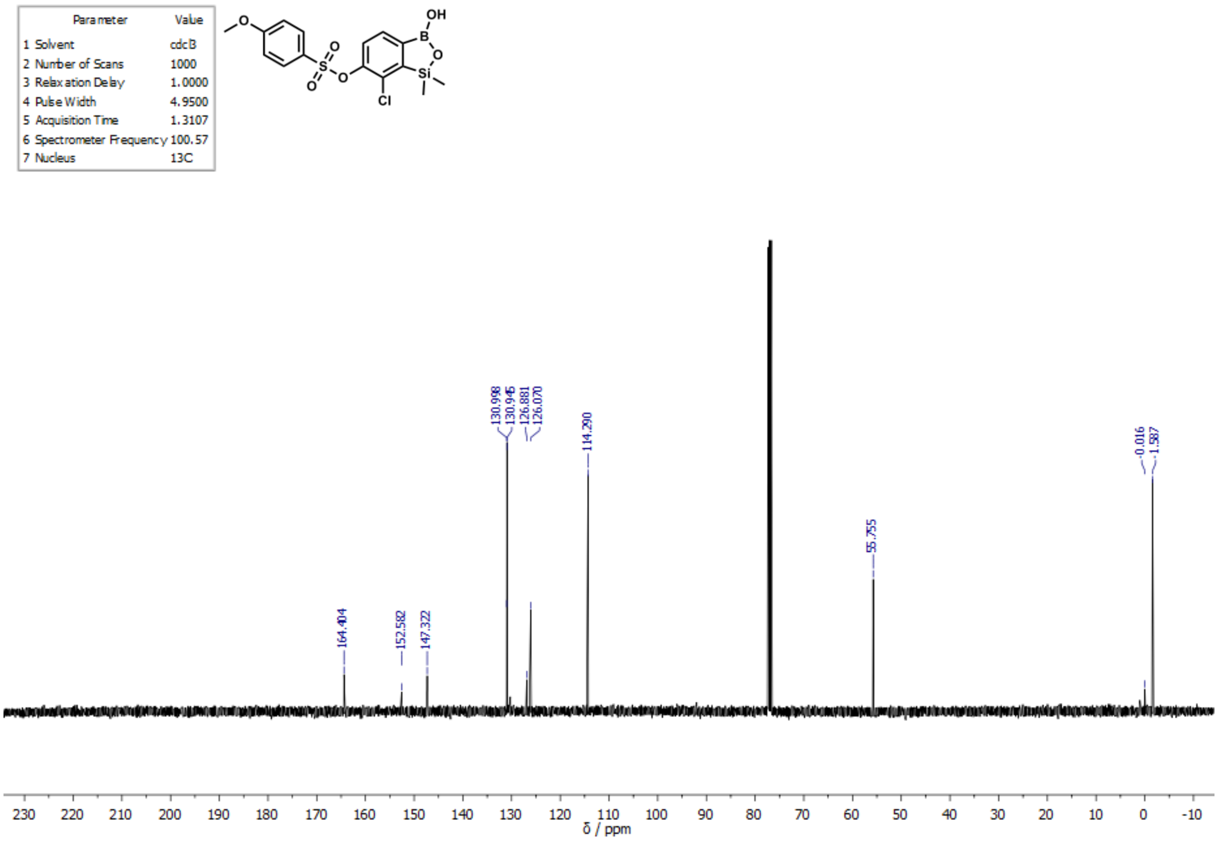

Figure S62. ^{13}C NMR spectrum of **9n** in $CDCl_3$

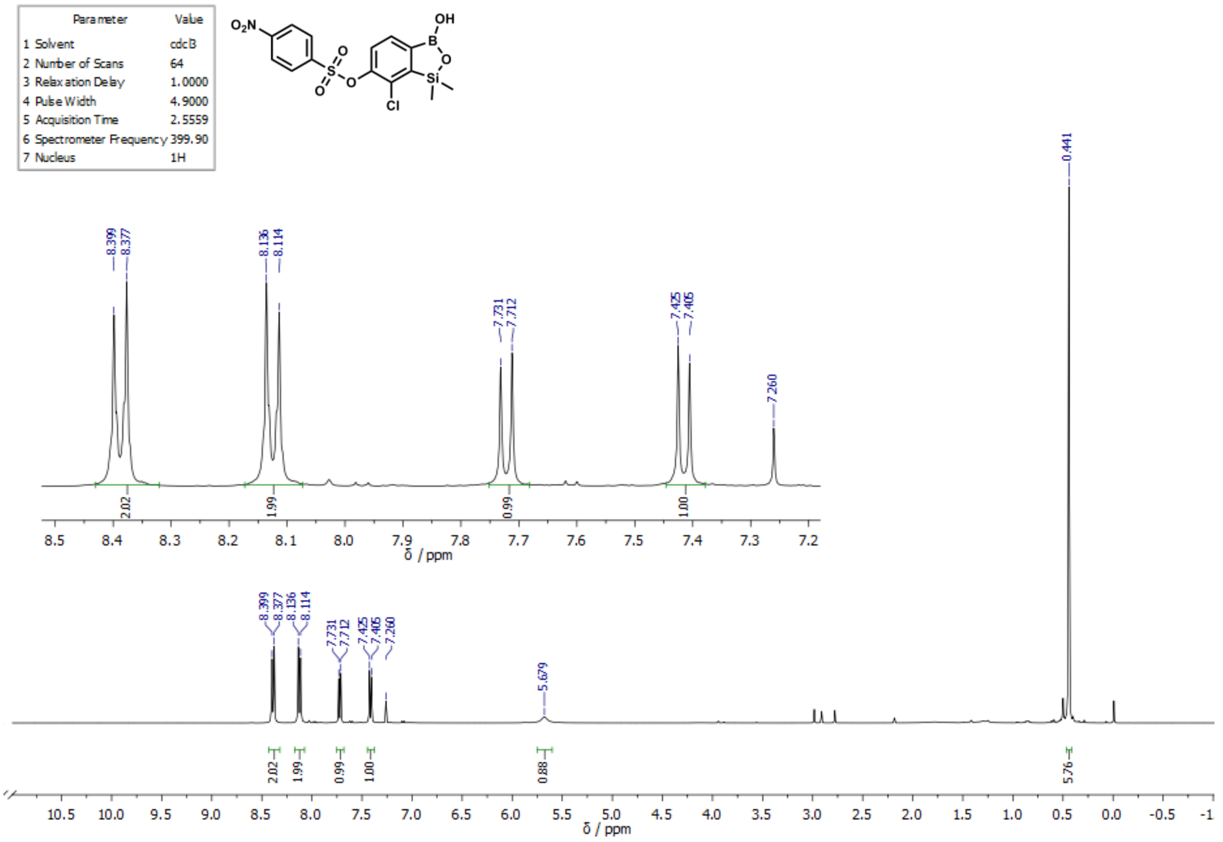

Figure S63. ^{1}H NMR spectrum of **9o** in $CDCl_3$.

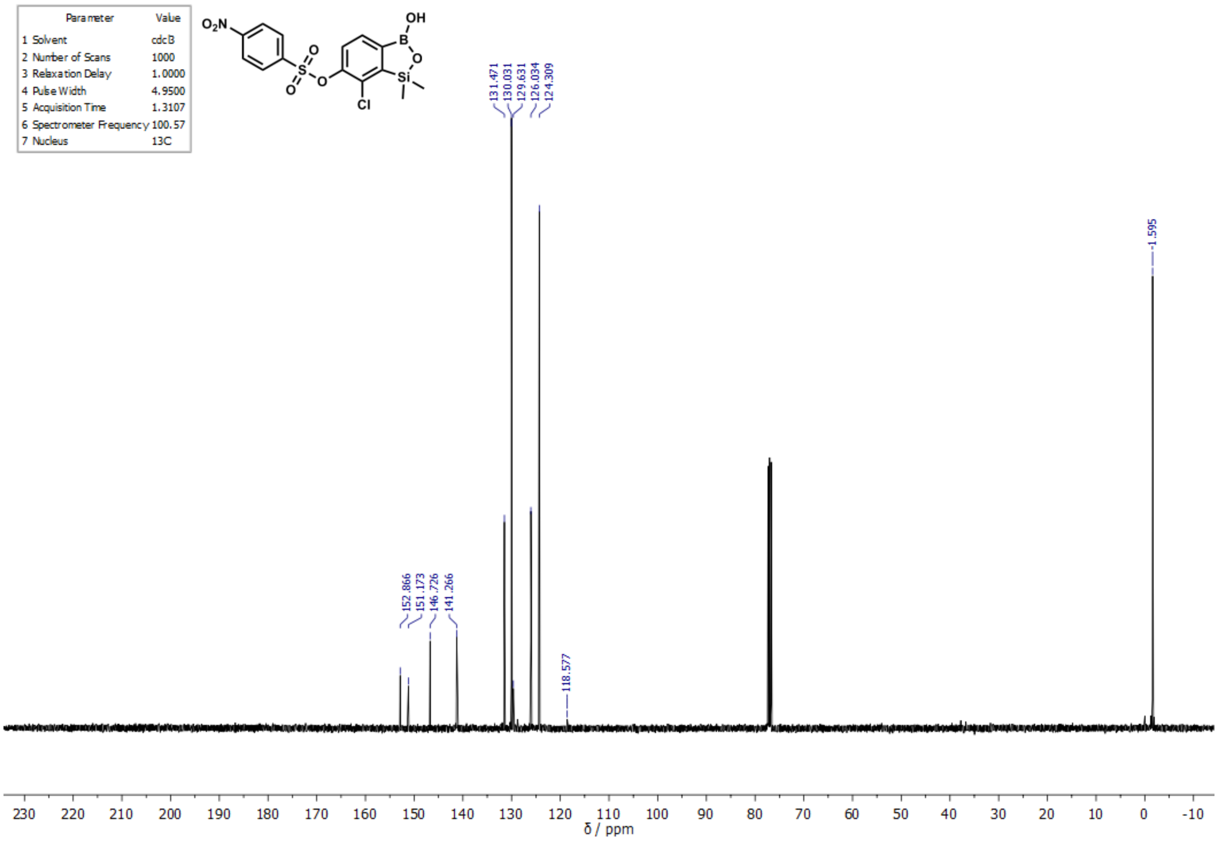

Figure S64. ^{13}C NMR spectrum of **9o** in $CDCl_3$.

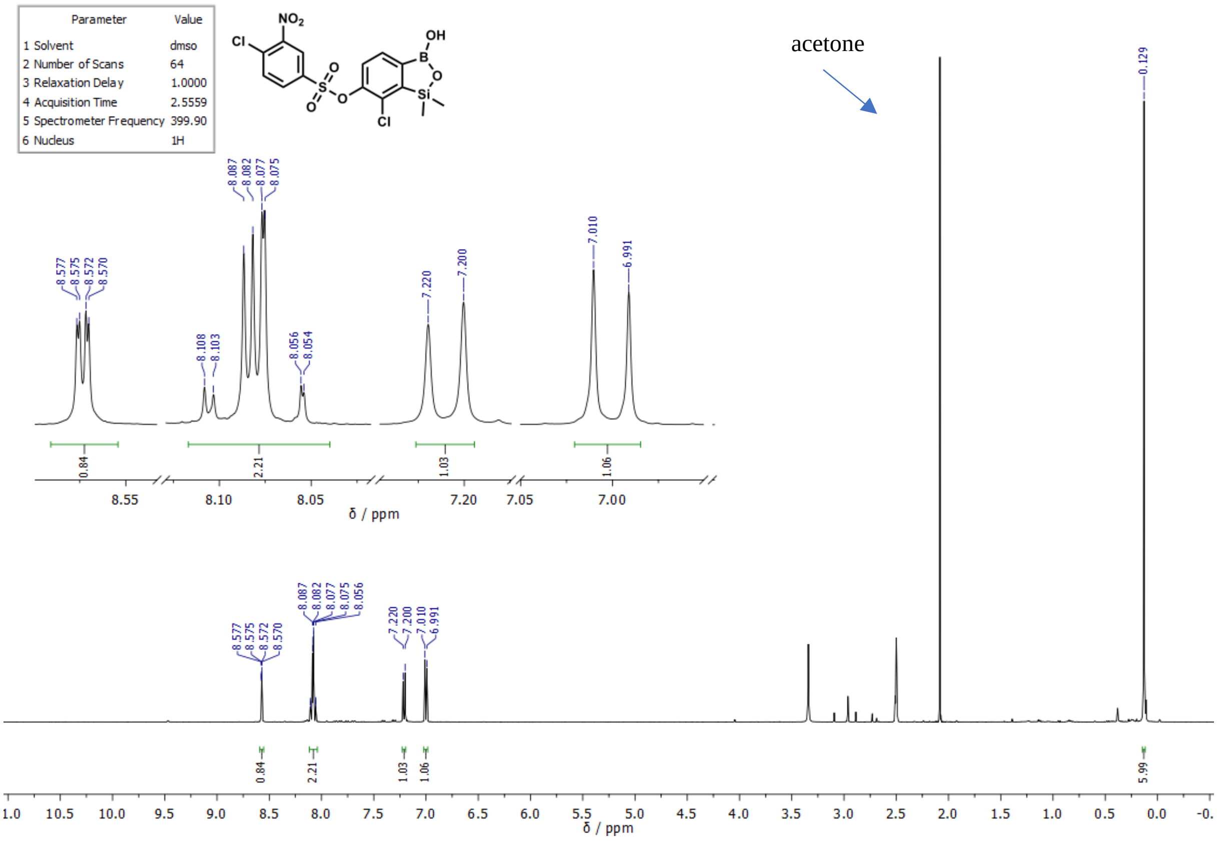

Figure S65. ^{1}H NMR spectrum of **9p** in DMSO.

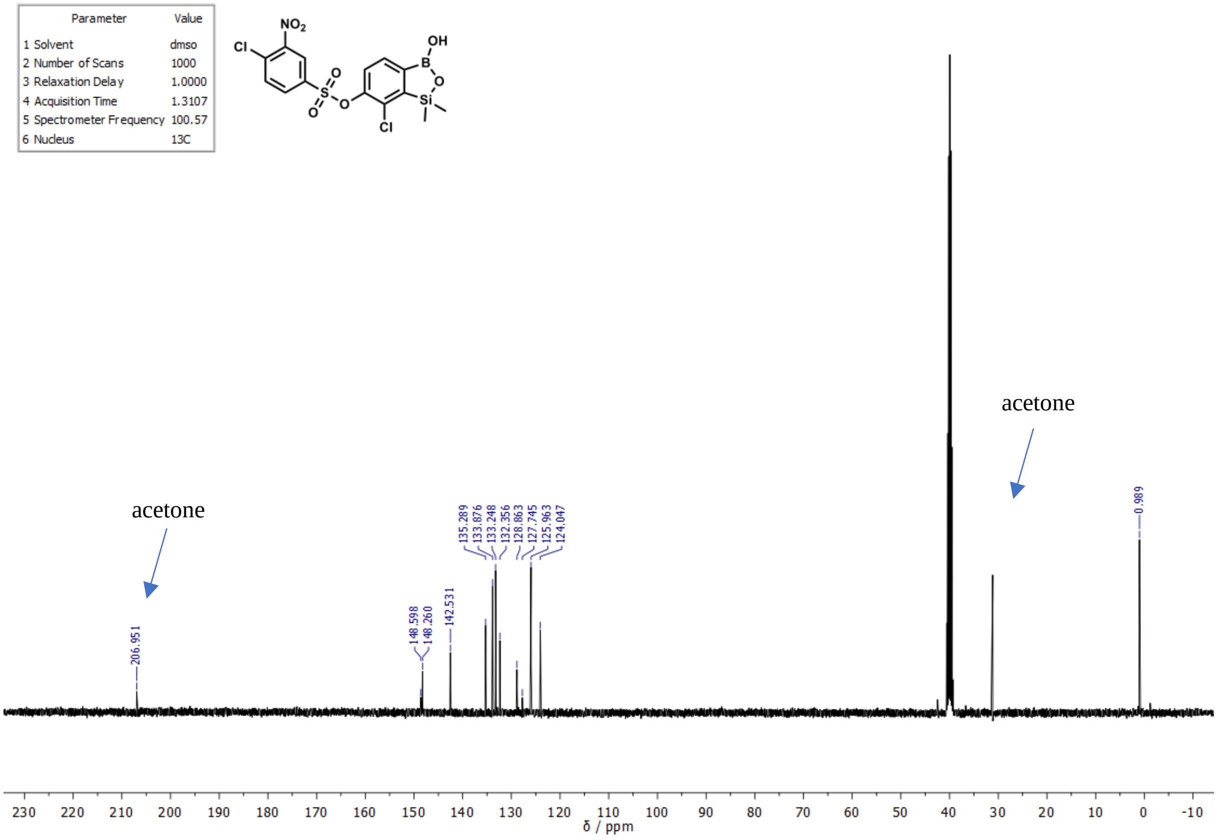

Figure S66. ^{13}C NMR spectrum of **9p** in DMSO.

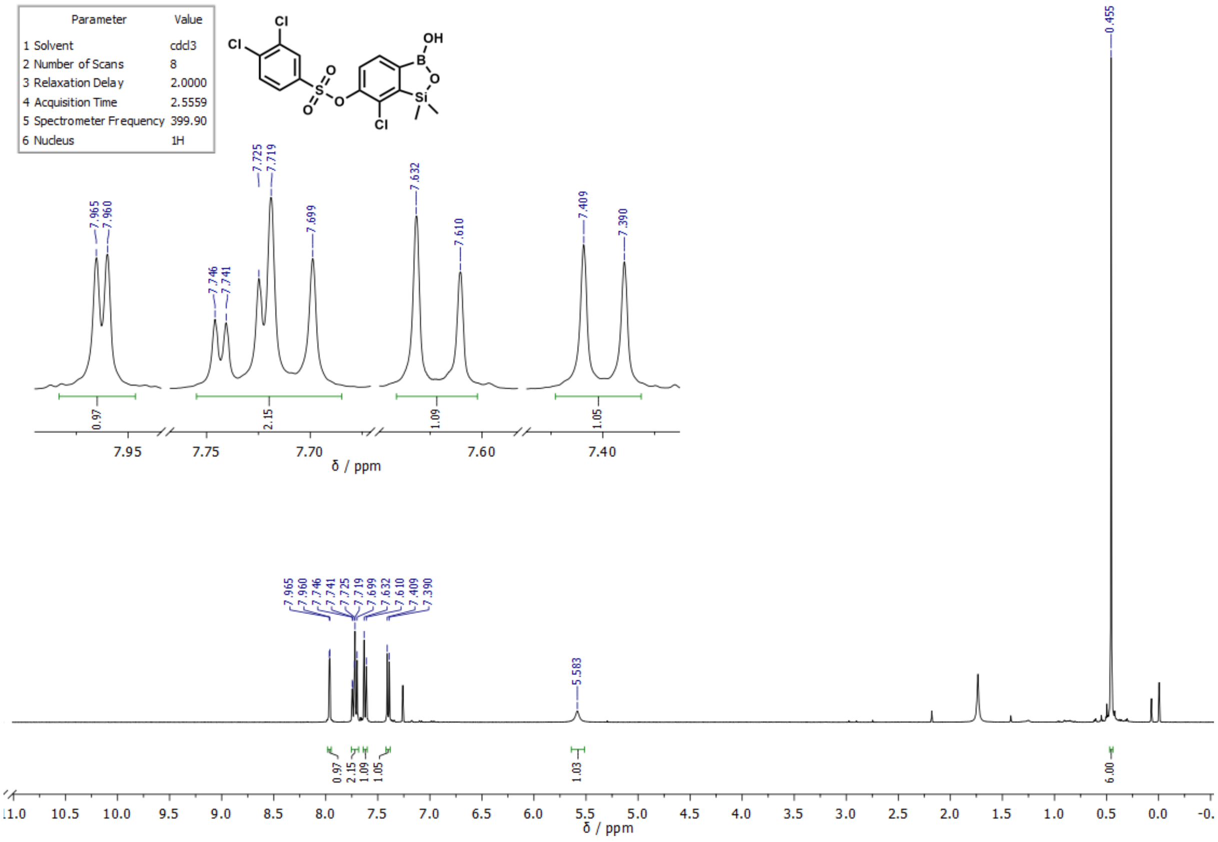

Figure S67. ^{1}H NMR spectrum of **9q** in $CDCl_3$.

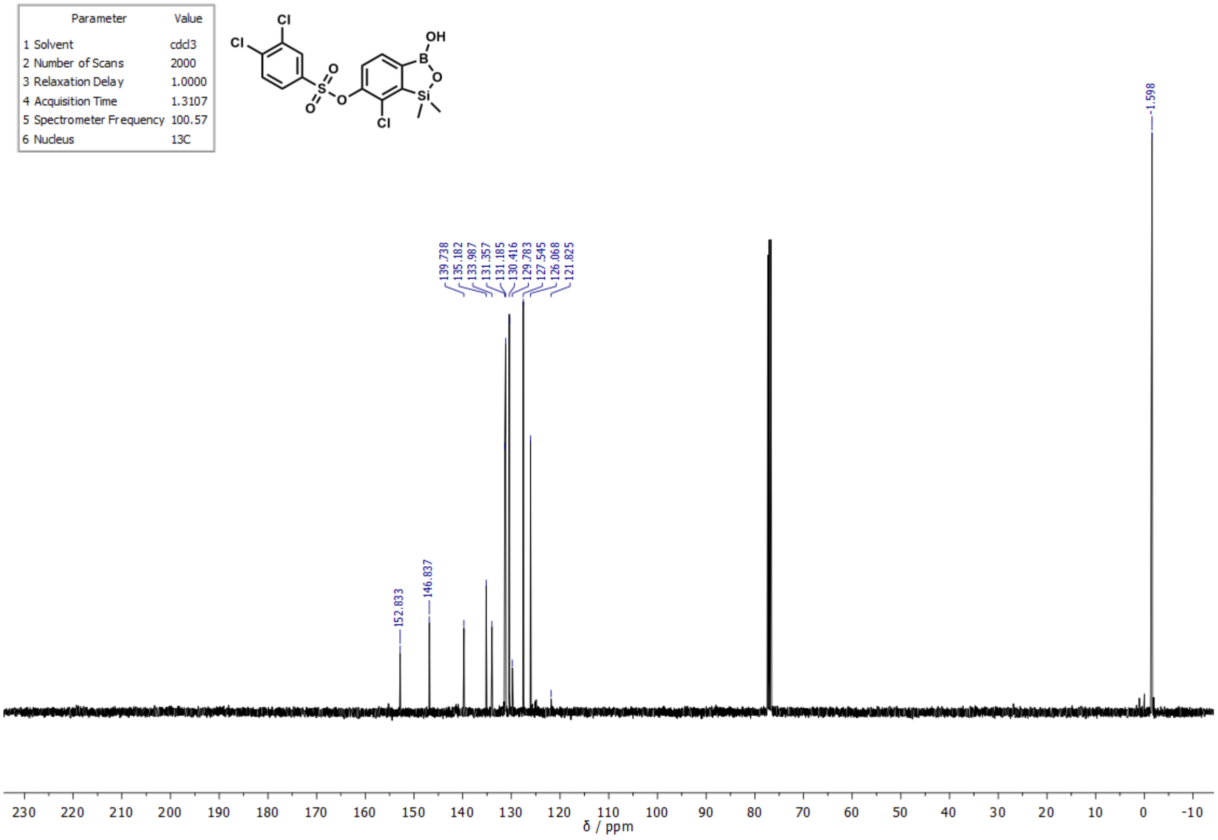

Figure S68. ^{13}C NMR spectrum of **9q** in $CDCl_3$.

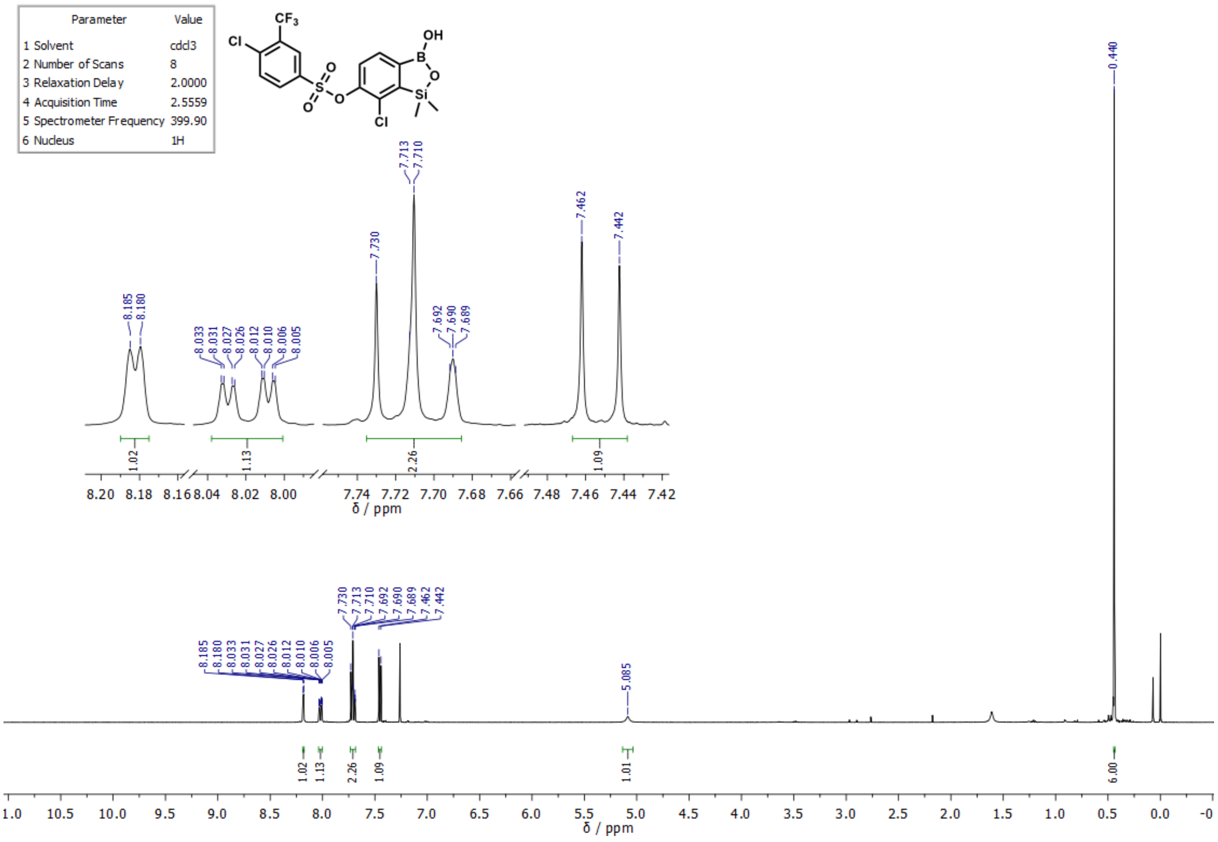

Figure S69. ^{1}H NMR spectrum of **9r** in $CDCl_3$.

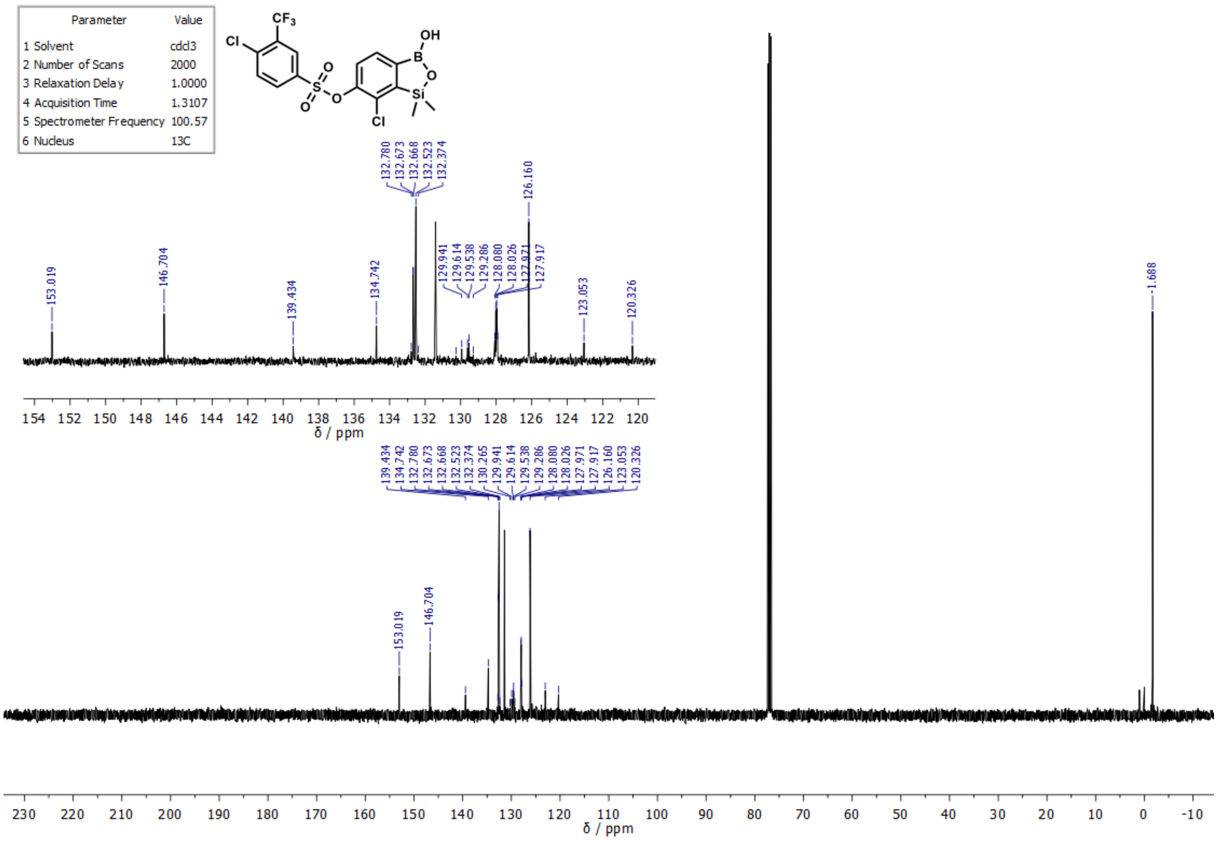

Figure S70. ^{13}C NMR spectrum of **9r** in $CDCl_3$.

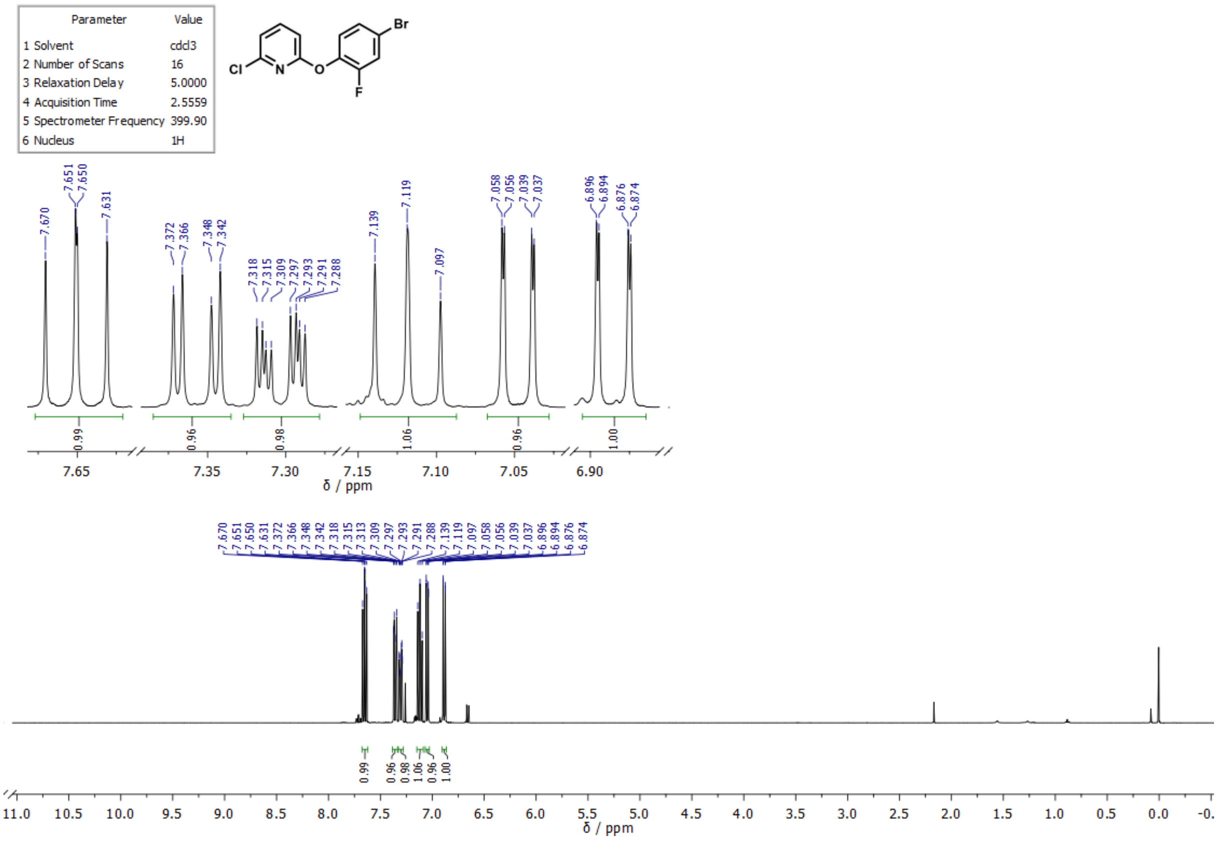

Figure S71. ^{1}H NMR spectrum of **11b** in $CDCl_3$.

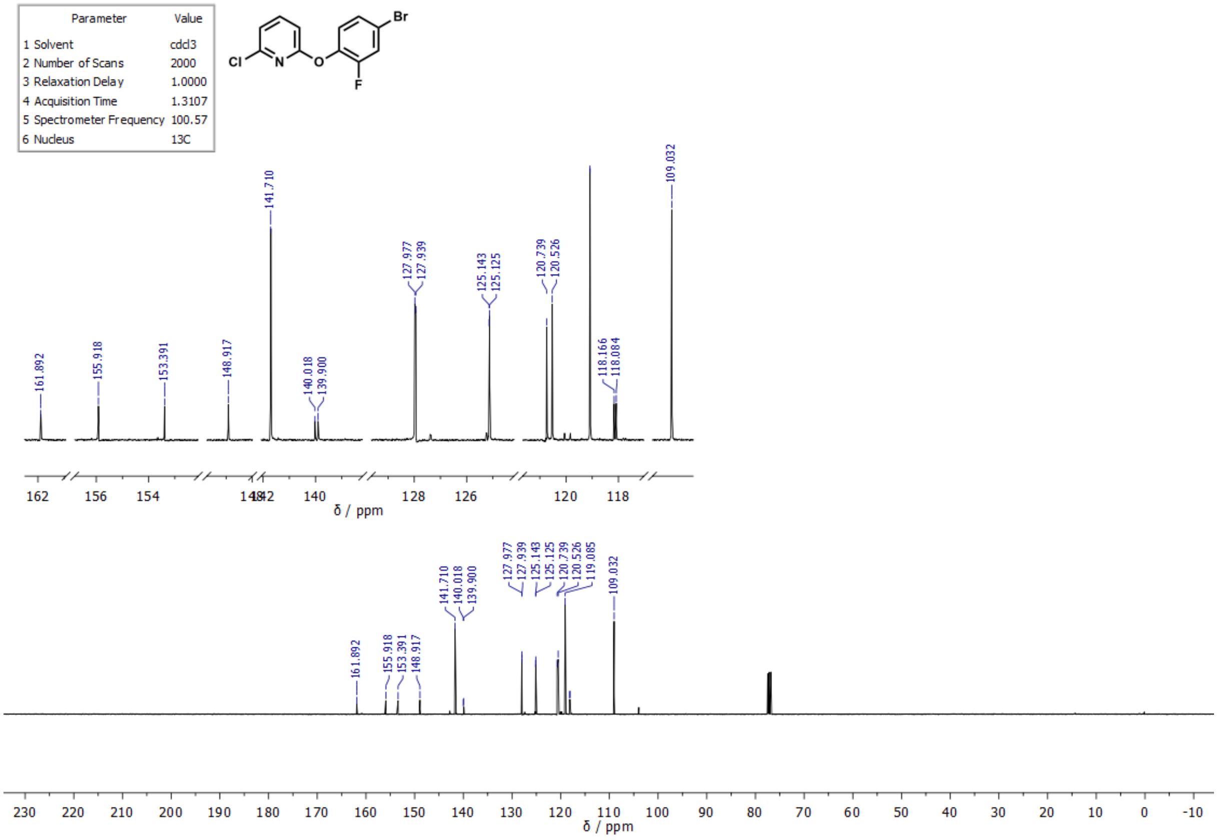

Figure S72. ^{13}C NMR spectrum of **11b** in $CDCl_3$.

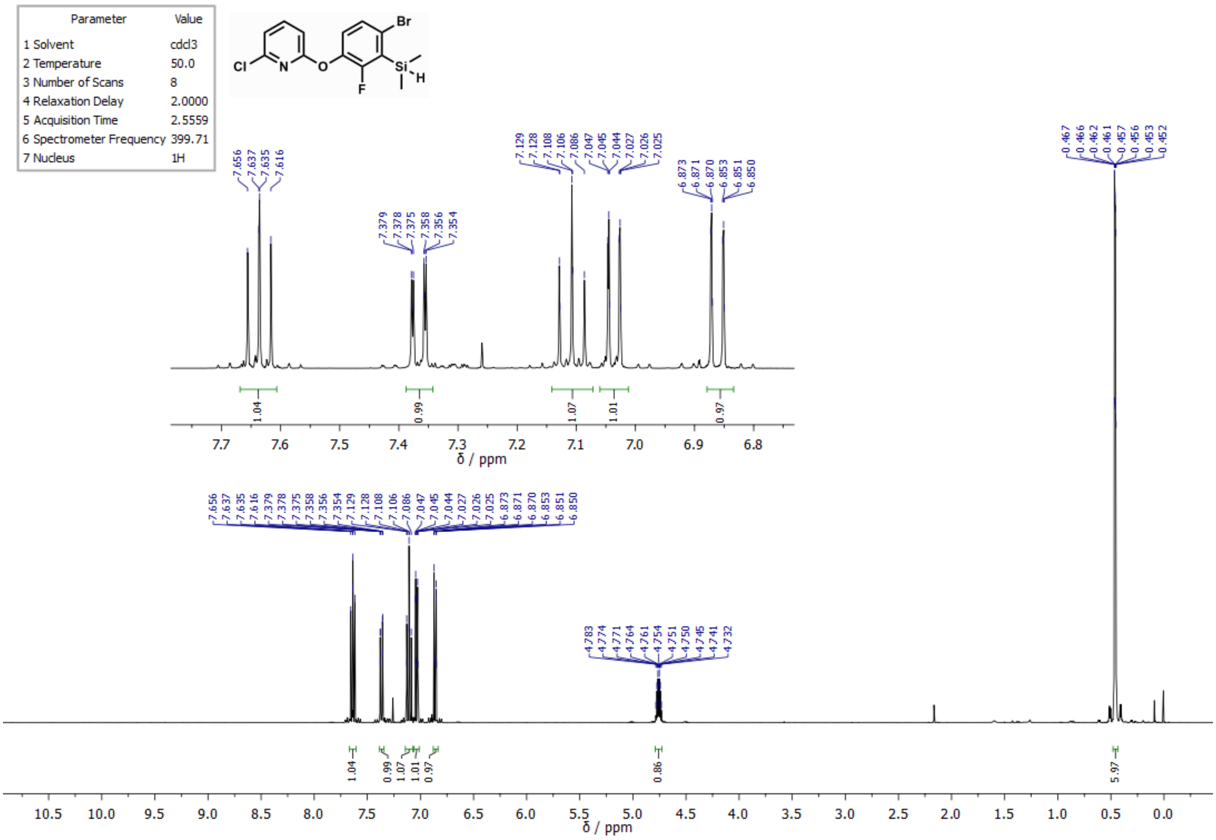

Figure S73. ^{1}H NMR spectrum of **12b** in $CDCl_3$.

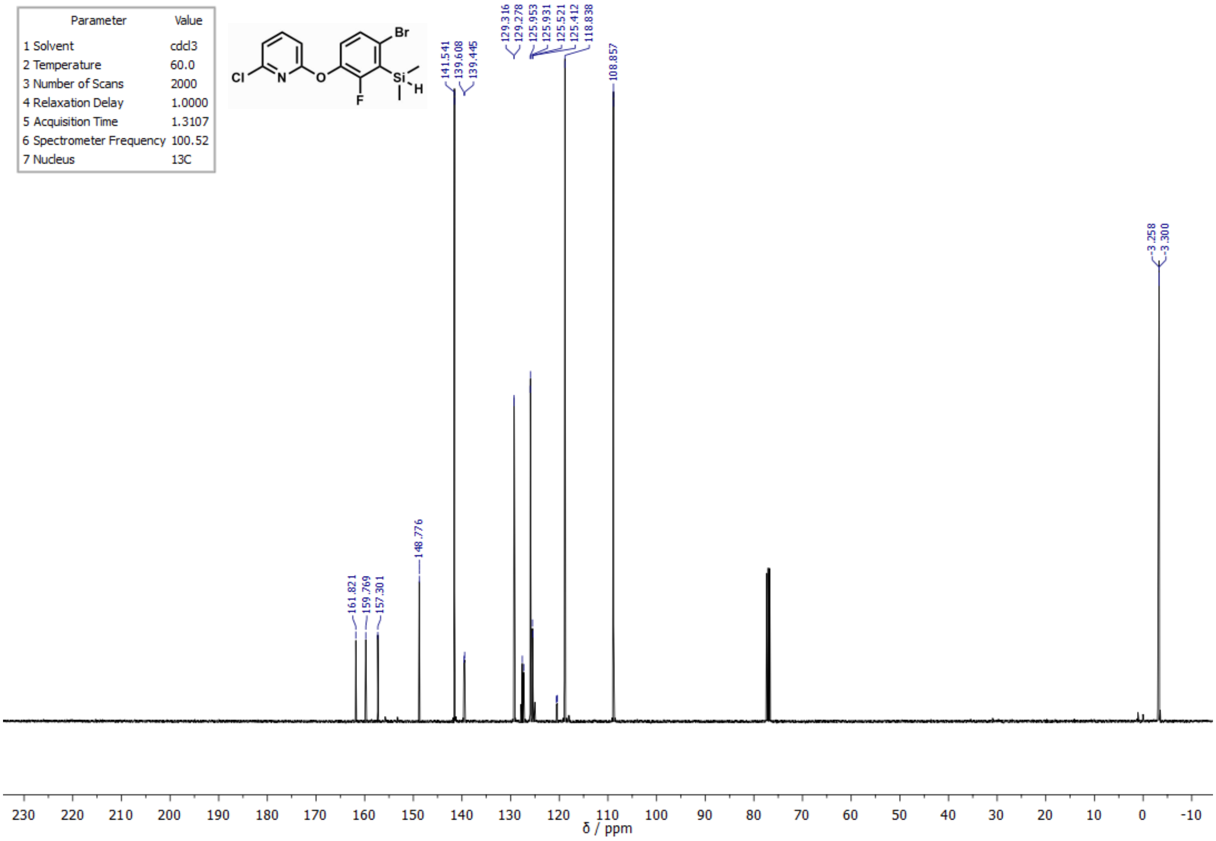

Figure S74. ^{13}C NMR spectrum of **12b** in $CDCl_3$.

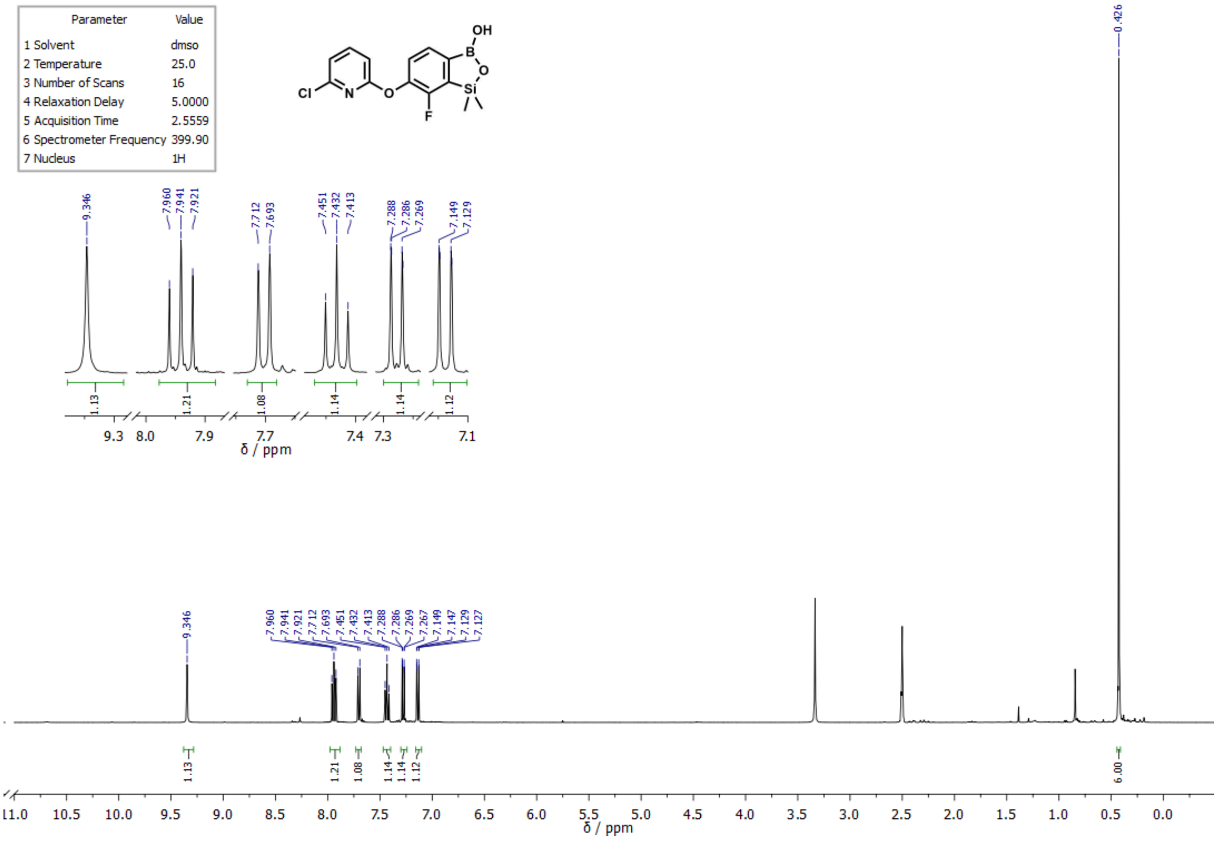

Figure S75. ^{1}H NMR spectrum of **13b** in DMSO.

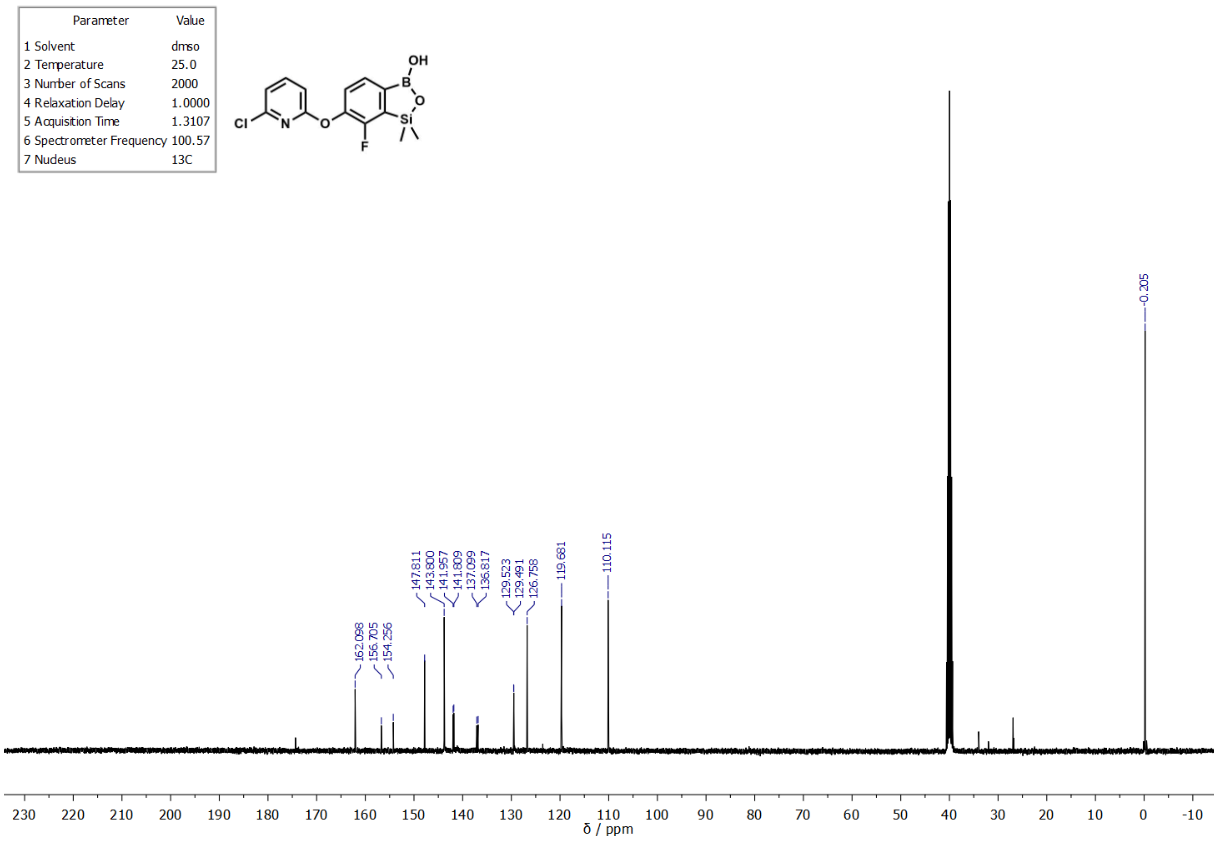

Figure S76. ^{13}C NMR spectrum of **13b** in DMSO.

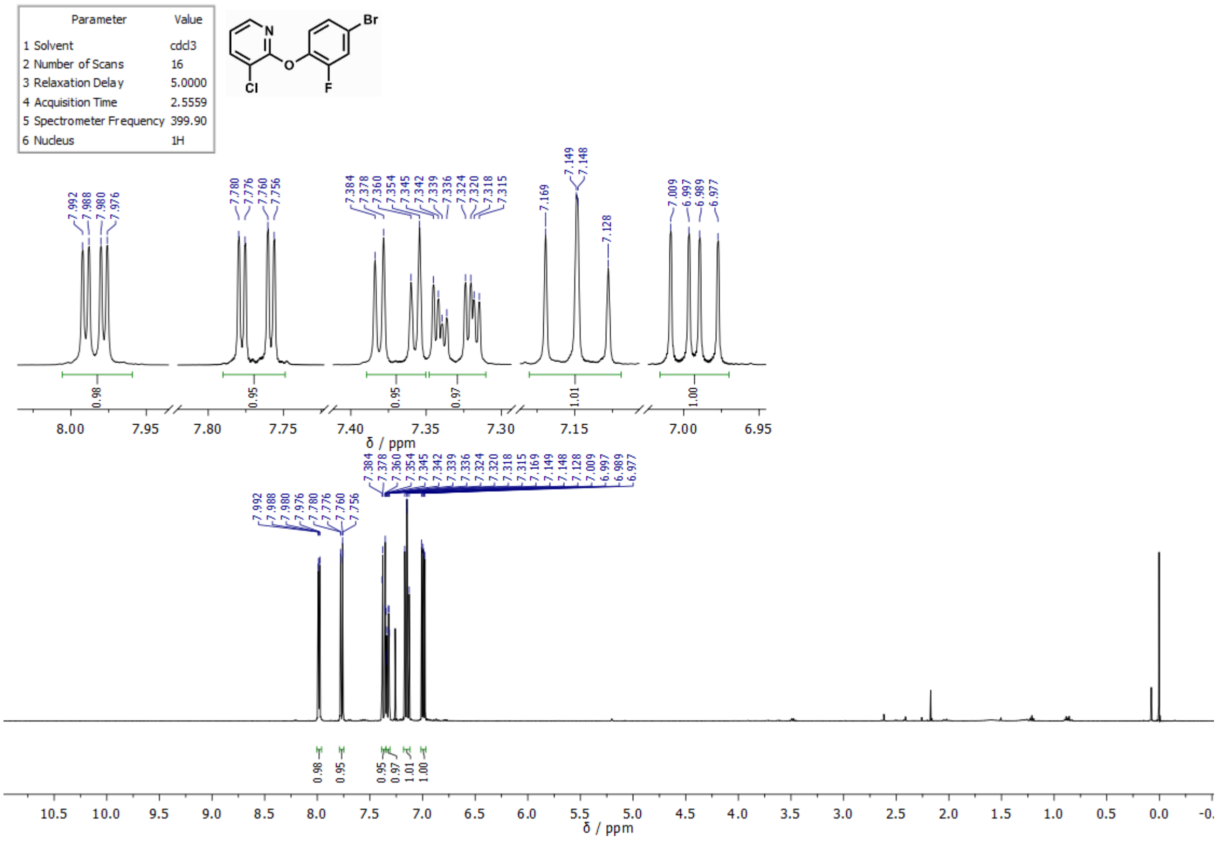

Figure S77. ^{1}H NMR spectrum of **11a** in $CDCl_3$.

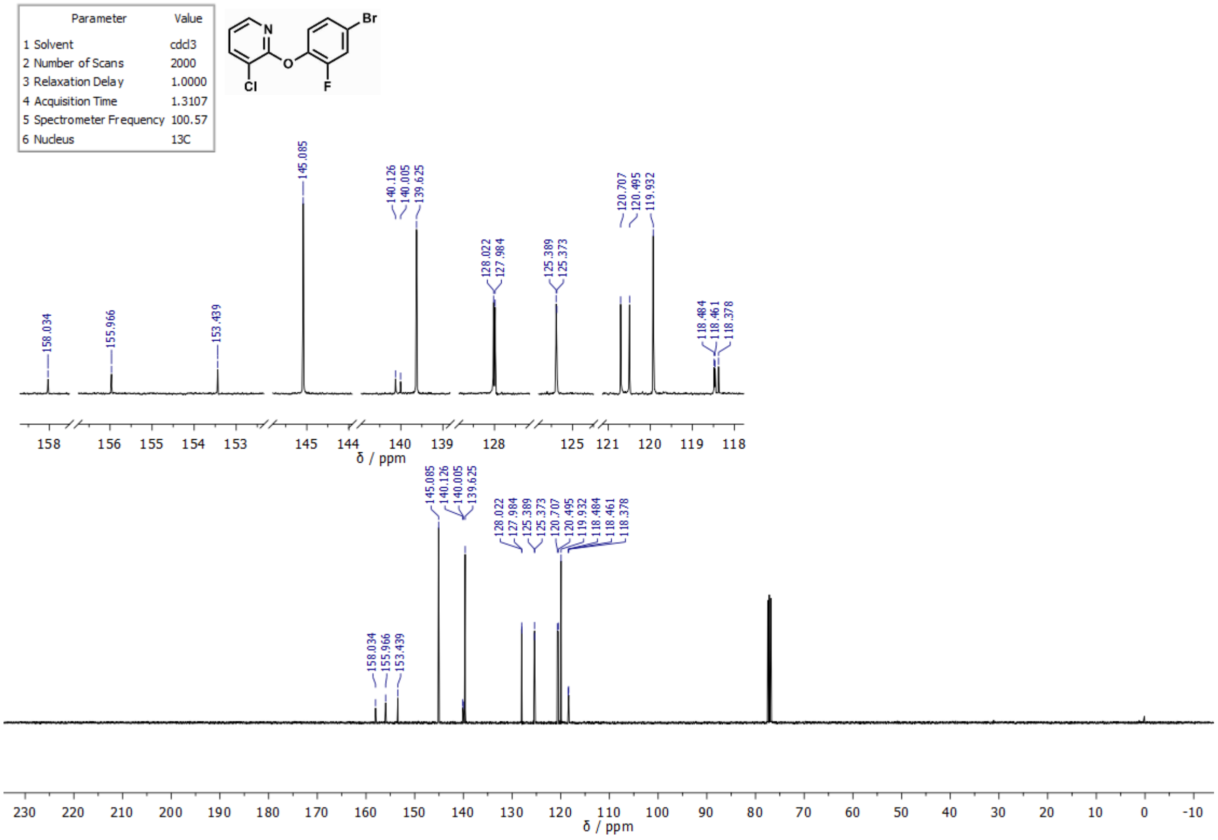

Figure S78. ^{13}C NMR spectrum of **11a** in $CDCl_3$.

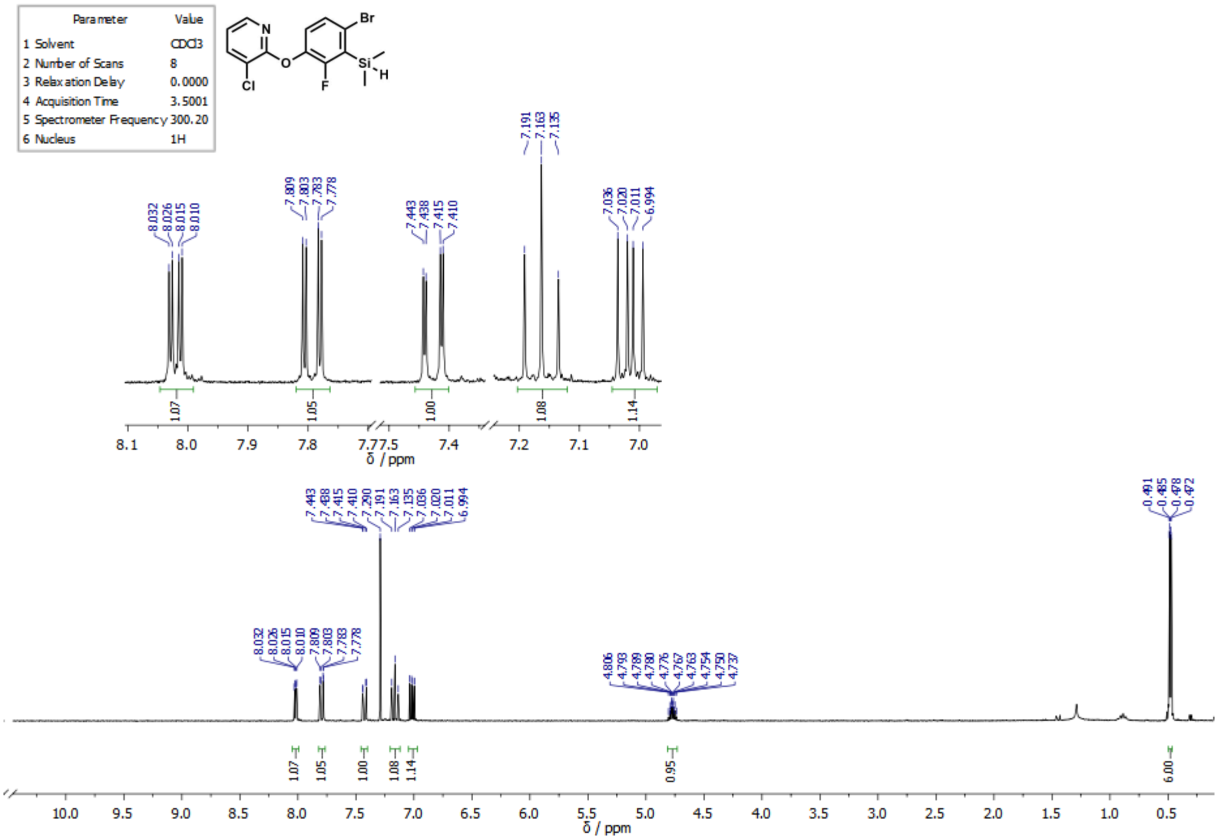

Figure S79. ^{1}H NMR spectrum of **12a** in $CDCl_3$.

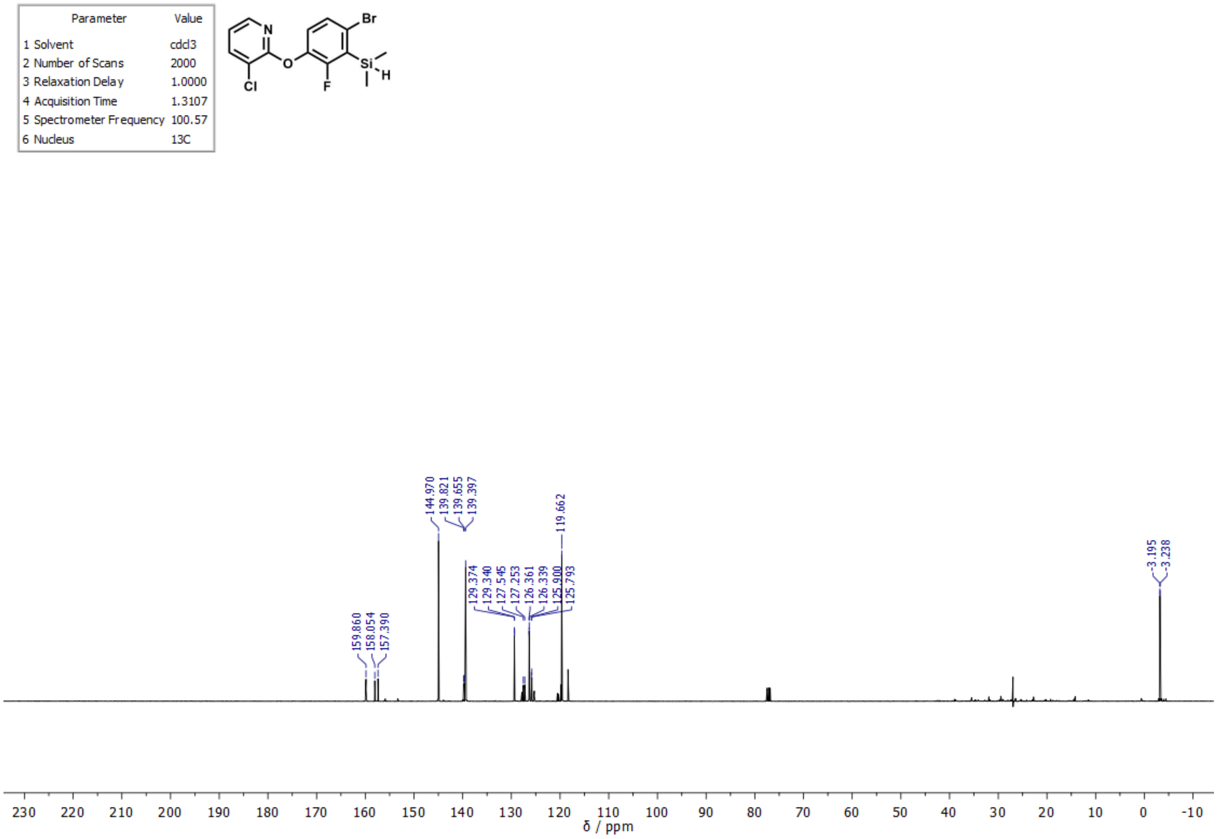

Figure S80. ^{13}C NMR spectrum of **12a** in $CDCl_3$.

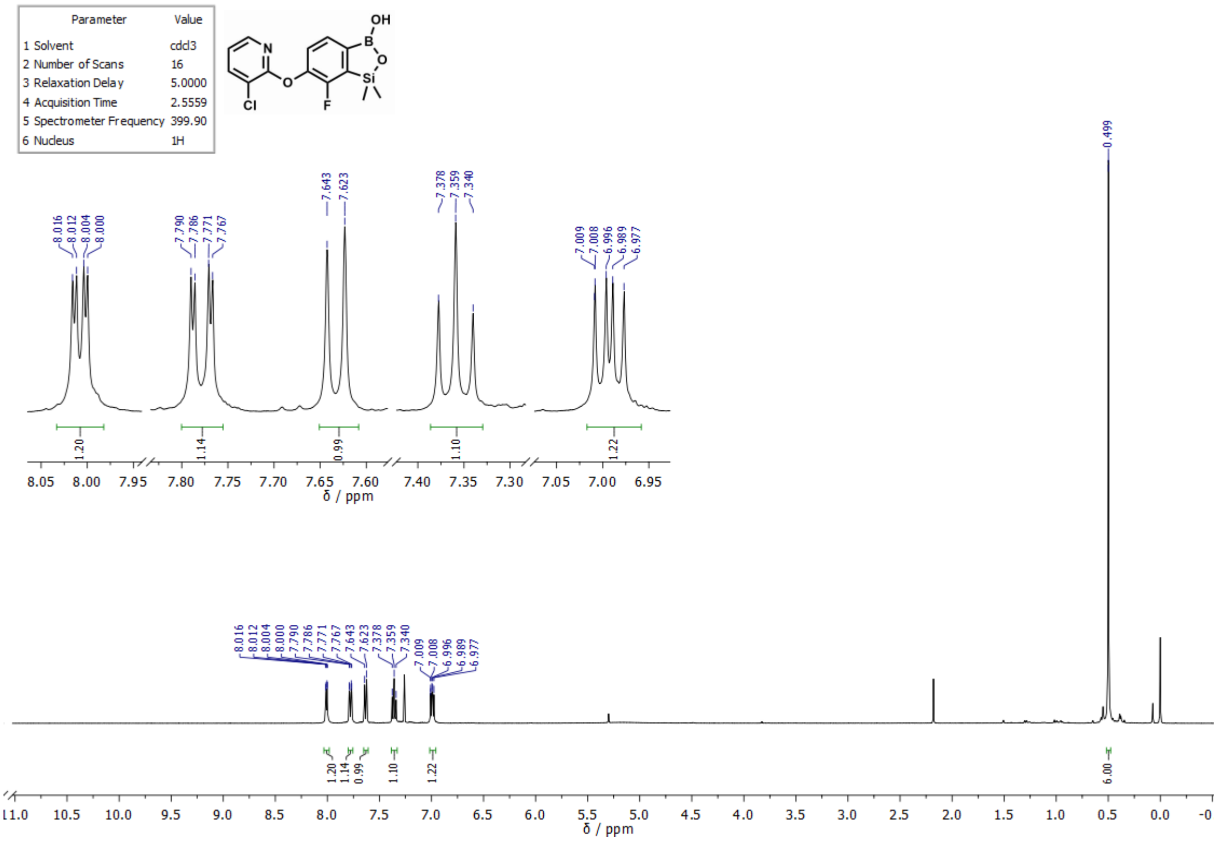

Figure S81. ^{1}H NMR spectrum of **13a** in $CDCl_3$.

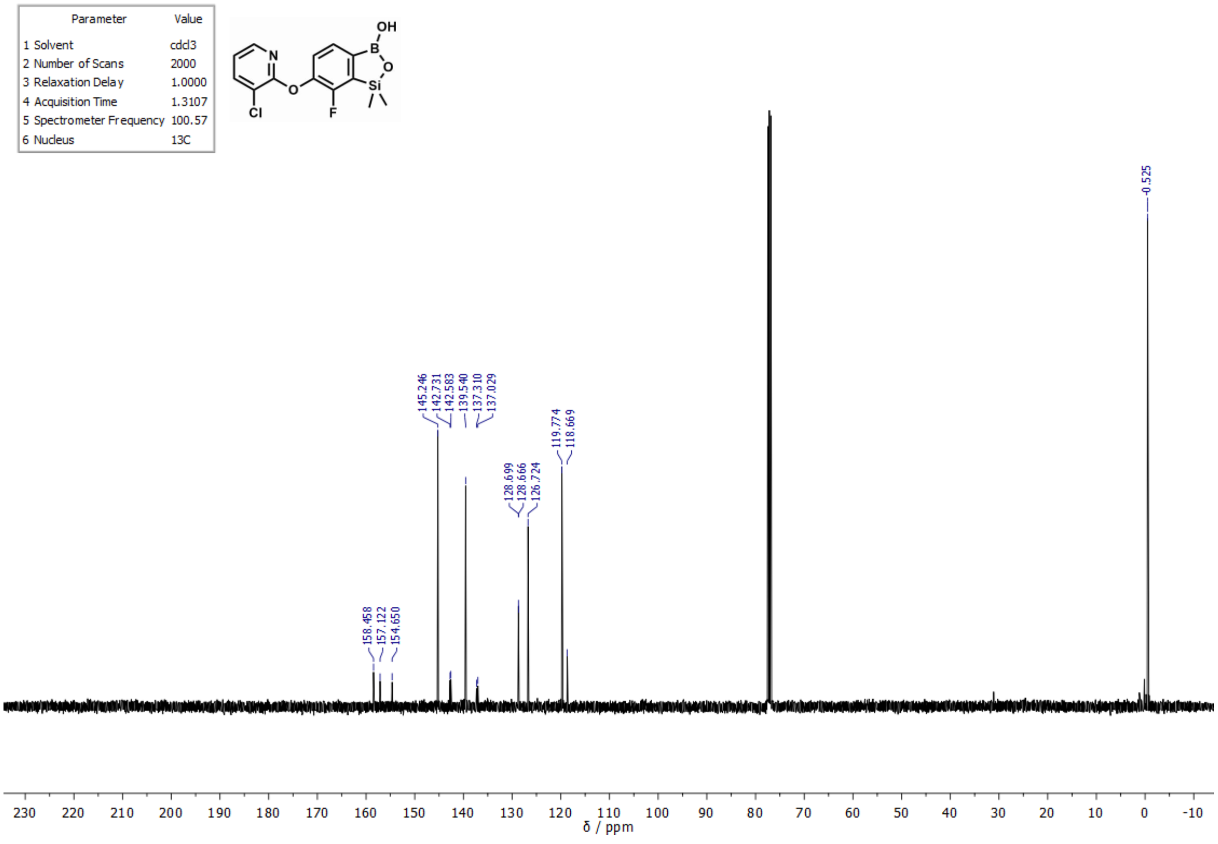

Figure S82. ^{13}C NMR spectrum of **13a** in $CDCl_3$.

2. Single-crystal X-ray diffraction analysis

Crystal structure determination. X-ray diffraction data sets for single crystals of **4b**, **5b**, **8a**, **9a**, **9h** and **13a** were collected at 100 K on a SuperNova diffractometer equipped with an Atlas detector (Cu-*K*α radiation, λ = 1.5418 Å). Data reduction and analysis were carried out with the *CrysAlisPro* program. [1] All structures were solved by direct methods using *SHELXS-97* [2] and refined using *SHELXL-2014.* [3] All non-hydrogen atoms were refined anisotropically. Crystallographic Information Files (CIFs) have been deposited with the Cambridge Crystallographic Data Centre as supplementary publications no. 2068345 (**4b**), 2068347 (**5b**), 2069477 (**6**), 2068346 (**8a**), 2068348 (**9a**), 2077619 (**9h**) and 2068349 (**13a**). Relevant crystallographic data is provided in **Table S1**.

Table S1. Selected crystal data, data collection and refinement parameters for crystal structures **4b, 5b, 6.**

	**4b**	**5b**	**6**
Formula	$C_{14}H_{24}BClO_3Si_2$	$(C_8H_9BClF_2KO_3Si)\cdot(H_2O)$	$C_9H_{12}BClO_3Si$
Molecular mass, M_r / a.u.	342.77	324.38	242.54
Temperature, T / K	100(1)	100(1)	100(1)
Crystal system	monoclinic	tetragonal	triclinic
Space group	$P2_1/n$	$P4_2/m$	P-1
a / Å	7.7496(3)	6.7599(4)	8.8909(2)
b / Å	21.2931(4)	16.7599(4)	13.0768(3)
c / Å	11.3262(6)	19.4746(9)	21.5996(8)
α / °	90	90	105.046(2)
β / °	96.315(6)	90	100.815(3)
γ / °	90	90	90.790(2)
Volume, V / Å^3	1857.63(13)	5470.3(4)	2376.92(12)
d_{calc} / gcm^{-3}	1.226	1.575	1.355
F(000)	728	2620	1008
Absorption coefficient, μ / mm^{-1}	3.102	6.968	3.696
No. of measured / independent reflections	14025 / 3013	21796 / 5221	31651 / 7081
R_{int} / %	0.0341	0.0792	0.0434
GooF	1.056	1.189	1.012
$R[F]$ / $wR[F^2]$ (I> 2σ(I)) / %	0.0322 / 0.0794	0.2600 / 0.4690	0.0444 / 0.1107
Max. and min. residual density / eÅ^{-3}	+0.411 / -0.223	+1.482 / -2.189	+ 0.624 / -0.357

Table S2. Selected crystal data, data collection and refinement parameters for crystal structures **8a, 9a, 13a.**

	**8a**	**9a**	**13a**
Formula	$C_{15}H_{14}BClO_4Si$	$C_{14}H_{14}BClO_5SSi$	$C_{13}H_{12}BClFNO_3Si$
Molecular mass, M_r / a.u.	332.61	368.66	323.59
Temperature, T / K	100(1)	100(1)	100(1)
Crystal system	monoclinic	monoclinic	monoclinic
Space group	$P2_1/c$	$P2_1/c$	$P2_1/n$
a / Å	10.1341(1)	11.1357(1)	8.4560(6)
b / Å	12.5199(2)	7.9341(1)	18.7604(7)
c / Å	12.6193(2)	18.6956(2)	9.4974(13)
α / °	90	90	90
β / °	91.705(1)	93.418(1)	93.128(14)
γ / °	90	90	90
Volume, V / Å^3	1600.40(4)	1648.85(3)	1504.4(2)
d_{calc} / gcm^{-3}	1.380	1.485	1.429
F(000)	688	760	664
Absorption coefficient, μ / mm^{-1}	2.956	4.126	3.194
No. of measured / independent reflections	13335 / 3073	13776 / 3262	10600 / 2420
R_{int} / %	0.0259	0.0282	0.0391
GooF	0.975	1.050	1.080
$R[F]$ / $wR[F^2]$ (I> 2σ(I)) / %	0.0290 / 0.0790	0.0290 / 0.0821	0.0498 / 0.1201
Max. and min. residual density / eÅ^{-3}	+0.354 / -0.282	+0.406 / -0.412	+0.633 / -0.432

Table S3. Selected geometric parameters of crystal structures.

	**4b**	**5b**	**6**	**8a**	**9a**	**13a**
$d_{B\text{-}O(H)}$ / Å	1.341(3)	1.49(2)/ 1.45(2) ^{b}	1.345(4)	1.344(2)	1.342(2)	1.346(4)
$d_{B\text{-}O(Si)}$ / Å	1.400(3)	1.48(2)	1.396(3)	1.394(2)	1.392(2)	1.378(3)
$d_{B\text{-}C}$ / Å	1.570(3)	1.57(3)	1.561(5)	1.575(2)	1.573(2)	1.571(4)
$d_{Si\text{-}O}$ / Å	1.685(1)	1.59(1)	1.690(2)	1.694(1)	1.691(1)	1.674(2)
$d_{Si\text{-}C(ar)}$ / Å	1.872(2)	2.04(2)	1.867(3)	1.868(1)	1.877(1)	1.879(3)
$d_{C(ar)\text{-}O}$ / Å	1.371(2)	1.47(3)	1.354(4)	1.390(2)	1.401(2)	1.401(3)
$\alpha_{C\text{-}Si\text{-}O}$ / °	94.76(7)	96.0(8)	94.9(1)	94.58(5)	94.09(6)	95.5(1)
$\alpha_{Si\text{-}O\text{-}B}$ / °	113.3(1)	113(1)	113.0(2)	113.08(9)	113.81(9)	112.9(2)
$\alpha_{O\text{-}B\text{-}C}$ / °	112.3(2)	113(2)	113.0(2)	112.9(1)	112.5(1)	113.3(2)
$\alpha_{C\text{-}O\text{-}X}{}^a$ / °	124.0(1)	-	117.1(2)	116.2(1)	119.74(9)	117.6(2)

^{a}Valence angle with pendant OR group; ^{b}B-F bond distances are given.

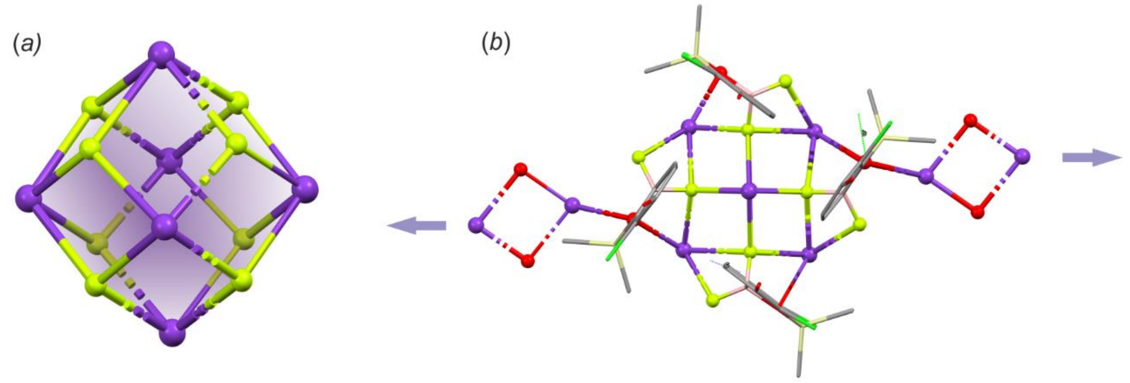

Figure S83. (a) Rhombic dodecahedron formed by potassium and fluorine atoms in crystal structure **5b**. (b) Fragment of 1-D polymeric chain. Blue arrows indicate network propagation.

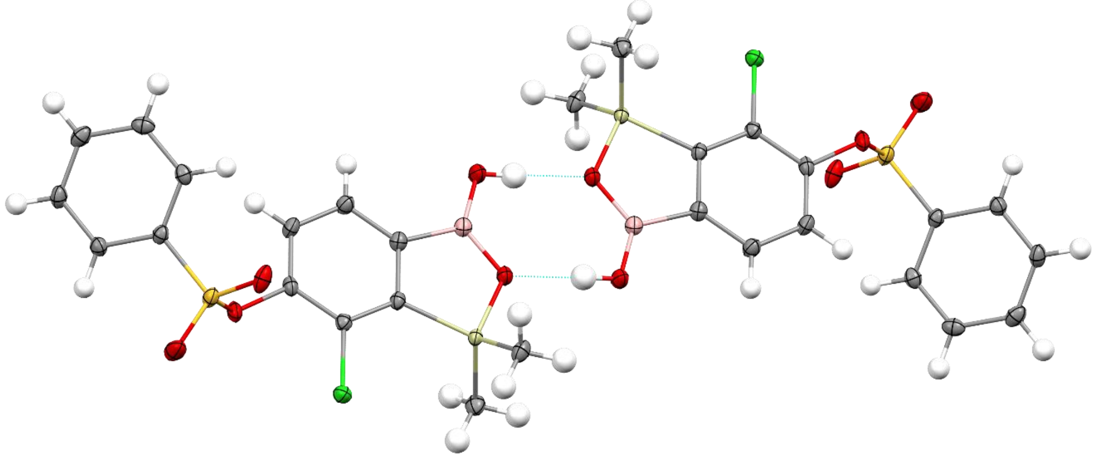

Figure S84. Hydrogen-bonded dimer in **9a.**

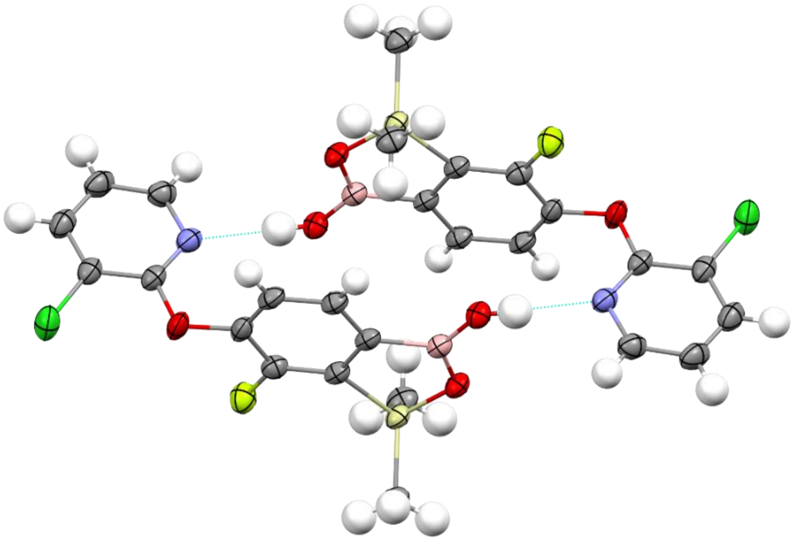

Figure S85. Hydrogen-bonded dimer in **13a.**

3. Antimicrobial activity.

Table S4. The antibacterial activity of tested agents against standard Gram-positive strains.

	**MIC in mg/L [MBC in mg/L][a] (Diameter of inhibition zone in mm)**					
**Agent tested**	***S. aureus*** **ATCC 6538P MSSA**	***S. aureus*** **ATCC 43300 MRSA**	***S. epidermidis*** **ATCC 12228**	***E. faecalis*** **ATCC 29212**	***E. faecium*** **ATCC 6057**	***B. subtilis*** **ATCC 6633[b]**
**4a**	NT (24)	NT (23)	NT (36)	NT (17)	NT (20)	NT (30)
**4b**	NT (21)	NT (21)	NT (29)	NT (15)	NT (16)	NT (26)
**6**	50 (24)	50 (24)	50 (26)	200 (14)	50 (17)	NT (27)
**7**	12.5 (22)	12.5 (22)	50 (26)	200 (14)	200 (15)	NT (30)
**8a**	12.5 (22)	25 [50] (22)	25 [200] (27)	50 [100] (16)	50 [200] (18)	NT (26)
**8b**	NT (19)	NT (18)	NT (24)	NT (16)	NT (17)	NT (23)
**8c**	NT (18)	NT (15)	NT (23)	NT (16)	NT (17)	NT (21)
**8d**	NT (23)	NT (24)	NT (24)	NT (17)	NT (19)	NT (24)
**8e**	NT (15)	NT (14)	NT (18)	NT (14)	NT (13)	NT (17)
**8f[c]**	12.5 [200] (20)	12.5 [100] (21)	12.5 [200] (19)	50 (-)	50 (12)	NT (15)
**8g**	100 (16)	100 (16)	100 (23)	400 (-)	400 (-)	NT (15)
**9a**	**1.56** [*6,25/200*][g](28)	**1.56** [*25/200*] (27)	12.5 [200] (24)	50 [400] (17)	50 (15)	NT (29)
**9b**	**3.12** [200] (26)	**3.12** [*12,5/200*](25)	12.5 [400] (18)	50 (-)	50 (-)	NT (16)
**9c**	**3.12** [*6.25/200*] (26)	**3.12** [*12,5/200*] (27)	12.5 [200] (27)	50 [400] (17)	50 (16)	NT (27)
**9d[c]**	**0.78** [*3.12/50*] (27)	**1,56** [*6,25/200*] (22)	**3.12** [50] (25)	12.5 [200] (16)	12.5 (15)	NT (28)
**9e[d]**	**1,56** [50] (26)	**1,56** [*6,25/50*] (25)	6.25 [100] (25)	12.5 [100] (17)	25 (14)	NT (26)
**9f**	NT (24)	NT (23)	NT (25)	NT (14)	NT (14)	NT (23)
**9g[c]**	**1.56** [*3.12/100*] (27)	**1,56** [*6,25/100*] (20)	6.25 [100] (26)	25 (16)	25 (17)	NT (24)
**9h[d]**	**0.39** [50] (28)	**1,56** [50] (26)	**3.12** [50] (22)	25 [100] (17)	25 (17)	NT (26)
**9i[c]**	**1.56** [*3,12/100*] (23)	**1,56** [*12,5/200*] (26)	6.25 (22)	25 (17)	25 (17)	NT (24)
**9j**	**1.56** [*3,12/100*] (28)	**1,56** [*12,5/200*] (25)	6.25 [200] (25)	25 [200] (17)	25 [200] (16)	NT (24)
**9k[e]**	**0.78** [50] (23)	**1.56** [50] (23)	**3.12** (22)	6.25 (16)	6.25 (16)	NT (21)
**9l**	NT (21)	NT (20)	NT (22)	NT (16)	NT (15)	NT (20)
**9m**	**3.12** [*6.25/200*] (18)	**3.12** [*12,5/200*] (26)	6.25 [400] (24)	50 (15)	25 (14)	NT (26)
**9n**	**1.56** [*6.25/400*] (29)	**1,56** [*12,5/400*] (26)	12.5 (22)	50 (16)	50 (15)	NT (29)
**9o**	**1.56** [50] (26)	**3,12** [50] (25)	**0.78** [50] (28)	50 (16)	12.5 (19)	NT (27)
**9p**	**3.12** [*12.5/50*] (23)	**3.12** [*6.25/50*] (24)	**3.12** [12.5] (22)	25 [400] (17)	25 (20)	NT (24)
**9q[e]**	**0.78** [*1.56/25*] (25)	**0.78** [*3.12/25*] (25)	**3.12** (25)	6.25 [50] (18)	6.25 [50] (20)	NT (24)
**9r[e]**	**0.39** [*0,78/25*] (25)	**0.39** [*1.56/25*] (27)	**3.12** (25)	6.25 [50] (19)	6.25 [50] (18)	NT (25)
**13a**	25 [200] (22)	25 [200] (22)	25 [400] (23)	100 (13)	50 (14)	NT (27)
**13b[c]**	25 [200] (20)	50 (22)	25 (22)	50 (15)	50 (15)	NT (24)
**LIN[f]**	1 [>128] (25)	2 [>128] (25)	1 [>128] (26)	2 [>128] (15)	2 [>128] (14)	NT (30)

The highest activity against Gram-positive bacteria indicated by the low MIC values (≤3.12 mg/L) is shown in boldface.

(-): The inhibition zone was not observed in the disc-diffusion method. The diameter of paper discs was 9 mm. NT: not tested. The MIC determination could not be performed, because tested substance dissolved in DMSO precipitated after implementation into the MHB (Mueller-Hinton II broth) medium.

[a] Only the MBC values ≤400 mg/L are presented.

[b] The growth type of *B. subtilis* in the MHB medium prevented reading the MIC values of tested substances.

[c] The MIC and MBC values of the substance were determined up to 200 mg/L. In the table, only the MBC values ≤200 mg/L are presented. The tested substance dissolved in DMSO precipitated after implementation into the MHB medium at a concentration above 200 mg/L.

[d] The MIC and MBC values of the substance were determined up to 100 mg/L. In the table, only the MBC values ≤100 mg/L are presented. The tested substance dissolved in DMSO precipitated after implementation into the MHB medium at a concentration above 100 mg/L.

[e] The MIC and MBC values of the substance were determined up to 50 mg/L. In the table, only the MBC values ≤50 mg/L are presented. The tested substance dissolved in DMSO precipitated after implementation into the MHB medium at a concentration above 50 mg/L.

[f] LIN, linezolid was used as a reference agent active against Gram-positive bacteria. The diameter of commercial disc containing 0.03 mg of linezolid was 6 mm; the MIC of linezolid was determined according to the CLSI recommendations.[4]

[g] The Eagle effect was observed during the determination of the MBC value of same tested agents against *S. aureus* strains.[5] The Eagle effect is shown in italic face.

Table S5. The antibacterial activity of tested agents against standard Gram-negative strains.

	**MIC in mg/L [MBC in mg/L][a] / x-fold reduction of MIC in the presence of PAßN[b] (Diameter of inhibition zone in mm)**										
**Agent tested**	***E. coli*** **ATCC 25922**	***K. pneumonia*** **ATCC 13883**	***P. mirabilis*** **ATCC 12453**	***E. cloacae*** **DSM 6234**	***S. marcescens*** **ATCC 13880**	***P. aeruginosa*** **ATCC 27853**	***S. maltophilia*** **ATCC 13637**	***S. maltophilia*** **ATCC 12714**	***A. baumannii*** **ATCC 19606**	***B. cepacia*** **ATCC 25416[c]**	***B.bronchiseptica*** **ATCC 4617[c]**
**4a**	NT (-)	NT (-)	NT (-)	NT (-)	NT (-)	NT (-)	NT (-)	NT (-)	NT (-)	NT (-)	NT (-)
**4b**	NT (-)	NT (-)	NT (-)	NT (-)	NT (-)	NT (-)	NT (-)	NT (-)	NT (-)	NT (-)	NT (-)
**6**	>400/**4** (-)	>400/2 (-)	>400 (-)	>400/2 (-)	>400 (-)	>400 (-)	400 [400]/2 (-)	400 [400]/2 (-)	>400/2 (-)	>400 (-)	>400 (-)
**7**	>400 (-)	>400 (-)	>400 (-)	>400 (-)	>400 (-)	>400 (-)	>400 (-)	400 (-)	>400 (-)	>400 (-)	>400 (-)
**8a**	>400 (-)	>400 (-)	>400 (-)	>400 (-)	>400 (-)	>400 (-)	400/2 (-)	200/2 (-)	>400/**4** (-)	>400 (-)	200 (-)
**8b**	NT (-)	NT (-)	NT (-)	NT (-)	NT (-)	NT (-)	NT (-)	NT (-)	NT (-)	NT (-)	NT (-)
**8c**	NT (-)	NT (-)	NT (-)	NT (-)	NT (-)	NT (-)	NT (-)	NT (-)	NT (-)	NT (-)	NT (-)
**8d**	NT (-)	NT (-)	NT (-)	NT (-)	NT (-)	NT (-)	NT (-)	NT (-)	NT (-)	NT (-)	NT (-)
**8e**	NT (-)	NT (-)	NT (-)	NT (-)	NT (-)	NT (-)	NT (-)	NT (-)	NT (-)	NT (-)	NT (-)
**8f[d]**	>200 (-)	>200 (-)	>200 (-)	>200 (-)	>200 (-)	>200 (-)	>200 (-)	>200 (-)	>200 (-)	>200 (-)	>200 (-)
**8g**	>400 (-)	>400/**4** (-)	>400 (-)	>400 (-)	>400 (-)	>400 (-)	400/2 (-)	400 (-)	>400 (-)	>400 (-)	400 (-)
**9a**	>400 (-)	>400 (-)	>400 (-)	>400 (-)	>400 (-)	>400 (-)	>400 (-)	400 (-)	>400 (-)	>400 (-)	400 (-)
**9b**	>400 (-)	>400 (-)	>400 (-)	>400 (-)	>400 (-)	>400 (-)	>400 (-)	>400 (-)	>400 (-)	>400 (-)	>400 (-)
**9c**	>400 (-)	>400 (-)	>400 (-)	>400 (-)	>400 (-)	>400 (-)	>400 (-)	400 (-)	>400 (-)	>400(-)	400 (-)
**9d[d]**	>200 (-)	>200 (-)	>200 (-)	>200 (-)	>200 (-)	>200 (-)	>200 (-)	>200 (-)	>200 (-)	>200 (-)	200 (-)
**9e[e]**	>100 (-)	>100 (-)	>100 (-)	>100 (-)	>100 (-)	>100 (-)	>100 (-)	>100 (-)	>100 (-)	>100 (-)	>100 (-)
**9f**	NT (-)	NT (-)	NT (-)	NT (-)	NT (-)	NT (-)	NT (-)	NT (-)	NT (-)	NT (-)	NT (-)
**9g[d]**	>200 (-)	>200 (-)	>200 (-)	>200 (-)	>200 (-)	>200 (-)	>200 (-)	200 (-)	>200 (-)	>200 (-)	>200 (-)
**9h**	>400 (-)	>400 (-)	>400 (-)	>400 (-)	>400 (-)	>400 (-)	>400 (12)	400 (-)	>400 (-)	>400 (-)	400 (-)
**9i[d]**	>200 (-)	>200 (-)	>200 (-)	>200 (-)	>200 (-)	>200 (-)	>200 (-)	>200 (-)	>200 (-)	>200 (-)	100 (-)
**9j**	>200 (-)	>200 (-)	>200 (-)	>200 (-)	>200 (-)	>200 (-)	>200 (-)	>200 (-)	>200 (-)	>200 (-)	100 (-)
**9k[f]**	>50 (-)	>50 (-)	>50 (-)	>50 (-)	>50 (-)	>50 (-)	>50 (-)	>50 (-)	>50 (-)	>50 (-)	>50 (-)
**9l**	NT (-)	NT (-)	NT (-)	NT (-)	NT (-)	NT (-)	NT (-)	NT (-)	NT (-)	NT (-)	NT (-)
**9m**	>400 (-)	>400 (-)	>400 (-)	>400 (-)	>400 (-)	>400 (-)	>400 (-)	>400 (-)	>400 (-)	>400 (-)	>400 (-)
**9n**	>400 (-)	>400 (-)	>400 (-)	>400 (-)	>400 (-)	>400 (-)	>400 (-)	>400 (-)	>400 (-)	>400 (-)	>400 (-)
**9o[d]**	>200 (-)	>200 (-)	>200 (-)	>200 (-)	>200 (-)	>200 (-)	>200 (-)	>200 (-)	>200 (-)	>200 (-)	>200 (-)
**9p**	>400 (-)	>400 (-)	>400 (-)	>400 (-)	>400 (-)	>400 (-)	>400 (-)	>400 (-)	>400 (-)	>400 (-)	100 (-)
**9q[f]**	>50/2 (-)	>50 (-)	>50 (-)	>50 (-)	>50 (-)	>50 (-)	>50 (-)	>50 (-)	>50 (-)	>50 (-)	50 (-)
**9r[f]**	>50/**4** (-)	>50 (-)	>50 (-)	>50 (-)	>50 (-)	>50 (-)	>50 (-)	>50 (-)	>50 (-)	>50 (-)	50 (-)
**13a**	>400 (-)	>400 (-)	>400 (-)	>400 (-)	>400 (-)	>400 (-)	>400/**4** (-)	>400/**4** (-)	>400 (-)	>400 (-)	>400 (-)

**13b**[d]	>200 (-)	>200 (-)	>200 (-)	>200 (-)	>200 (-)	>200 (-)	>200 (-)	>200 (-)	>200 (-)	>200 (-)	>200 (-)
**Nf**[f]	8 [8] (24)	32 [32] (23)	128 [>128] (9)	32 [32] (17)	128 [>128] (12)	>128 [>128] (-)	128 [>128] (-)	128 [>128] (-)	64 [128] (9)	32 [32] (12)	64 [128] (-)

PAβN , efflux pump inhibitor. The significant decreases (at least a 4-fold) in the MIC values of tested compounds after the addition of PAβN are shown in boldface. The test was performed in the MHB medium supplemented with 1 mM $MgSO_4$.

(-): The inhibition zone was not observed in the disc-diffusion method. The diameter of paper discs was 9 mm.

NT: not tested. The MIC determination could not be performed, because tested substance dissolved in DMSO precipitated after implementation into the MHB (Mueller-Hinton II broth) medium.

[a] Only the MBC values ≤400 mg/L are presented.

[b] In the table, only at least a 2-fold decrease in the MIC values of tested compounds after the addition of PAβN are presented.

[c] The growth of *B. cepacia* ATCC 25416 and *B. bronchiseptica* ATCC 4617 strains was inhibited in the MHB medium supplemented with 1 mM $MgSO_4$ and 20 mg/L PAβN.

[d] The MIC and MBC values of the substance were determined up to 200 mg/L. In the table, only the MBC values ≤200 mg/L are presented. The tested substance dissolved in DMSO precipitated after implementation into the MHB medium at a concentration above 200 mg/L.

[e] The MIC and MBC values of the substance were determined up to 100 mg/L. In the table, only the MBC values ≤100 mg/L are presented. The tested substance dissolved in DMSO precipitated after implementation into the MHB medium at a concentration above 100 mg/L.

[f] The MIC and MBC values of the substance were determined up to 50 mg/L. In the table, only the MBC values ≤50 mg/L are presented. The tested substance dissolved in DMSO precipitated after implementation into the MHB medium at a concentration above 50 mg/L.

[g] Nf, nitrofurantoin was used as a reference agent active against Gram-negative bacteria. The diameter of commercial disc containing 0.3 mg of nitrofurantoin was 6 mm; the MIC of nitrofurantoin was determined according to the CLSI recommendations. [4]

Table S6. The antifungal activity of tested agents against yeasts strains.

	**MIC in mg/L [MFC in mg/L][a] (Diameter of inhibition zone in mm)**						
**Agent tested**	***C. albicans* ATCC 90028**	***C. parapsilosis* ATCC 22019**	***C. tropicalis* IBA 171**	***C. tropicalis* ATCC 750**	***C. guilliermondii* IBA 155**	***C. krusei* ATCC 6258**	***S. cerevisiae* ATCC 9763**
**4a**	>400 (-)	>400 (-)	>400 (-)	>400 (-)	>400 (-)	>400 (-)	>400 (-)
**4b**	NT (-)	NT (12)	NT (11)	NT (-)	NT (19)	NT (-)	NT (-)
**6**	100 (21)	100 (18)	100 [400] (21)	200 (16)	50 [400] (30)	100 (15)	**3.12** [200] (37)
**7**	50 (20)	400 (-)	400 (-)	>400 (-)	200 (12)	200 (22)	**0.78** [6.25] (38)
**8a**	200 (-)	100 (-)	**12.5** [100] (13)	25 (15)	100 (14)	50 (-)	**0.39** [50] (26)
**8b[b]**	50 (-)	**12.5** (-)	**12.5** (14)	50 (13)	50 (14)	**12.5** (13)	**3.12** (20)
**8c[b]**	50 (-)	25 (-)	25 [50] (-)	>50 (-)	>50 (-)	**12.5** (-)	**12.5** [25] (16)
**8d[b]**	50 (-)	25 (12)	**3.12** (17)	**6.25** (17)	50 (-)	**12.5** (13)	**0.19** [12.5] (31)
**8e[b]**	>50 (-)	>50 (-)	**12.5** (-)	>50 (-)	>50 (-)	**12.5** (-)	>50 (-)
**8f**	100 (-)	100 (-)	200 (-)	100 (-)	50 (-)	100 (-)	50 [100] (-)
**8g**	>400 (-)	>400 (-)	>400 (-)	>400 (-)	>400 (-)	>400 (-)	>400 (-)
**9a**	>400 (-)	>400 (-)	400 (-)	400 (-)	>400 (-)	100 (-)	**0.78** [25] (22)
**9b**	>400 (-)	>400 (-)	400 (-)	400 (-)	400 (-)	200 (-)	**0.39** [50] (22)
**9c**	400 (-)	200 (-)	200 [400] (-)	400 (-)	100 (-)	50 (20)	50 [50] (21)
**9d**	200 (-)	100 (-)	50 [100] (-)	100 [200] (-)	25 (-)	25 [400] (17)	**12.5** [50] (20)
**9e**	200 (-)	100 (-)	50 [200] (-)	200 (-)	400 (-)	25 [200] (16)	**12.5** [25] (-)
**9f[b]**	>50 (18)	>50 (-)	>50 (-)	>50 (-)	>50 (-)	**12.5** (13)	**6.25** [25] (20)
**9g**	>400 (-)	>400 (-)	>400 (-)	400 (-)	>400 (-)	100 (-)	**12.5** [25] (23)
**9h**	200 (-)	400 (-)	100 (-)	400 (-)	>400 (-)	25 (13)	25 [50] (-)
**9i**	400 (-)	>400 (-)	100 (-)	400 (-)	200 (-)	100 (16)	**6.25** [50] (30)
**9j**	400 [400] (-)	400 (-)	100 [400] (-)	400 (-)	200 (-)	100 (-)	**1.56** [25] (34)
**9k[c]**	>100 (-)	>100 (-)	>100 (-)	>100 (-)	>100 (-)	50 (-)	25 [100] (15)
**9l[b]**	>50 (-)	>50 (-)	>50 (-)	>50 (-)	>50 (-)	>50 (-)	**0.19** [12.5] (20)
**9m**	>400 (-)	>400 (-)	>400 (-)	>400 (-)	>400 (-)	200 (-)	100 [100] (21)
**9n**	400 (-)	400 (-)	200 (-)	400 (-)	400 (-)	100 (15)	25 [50] (20)
**9o**	400 (-)	200 (-)	100 [400] (-)	200 (-)	>400 (-)	50 [400] (19)	50 [100] (18)
**9p**	100 (26)	100 (-)	50 (-)	100 (-)	>400 (-)	25 [400] (15)	25 [50] (-)
**9q[c]**	>100 (-)	>100 (-)	50 (-)	100 (-)	>100 (-)	25 (15)	**12.5** [50] (20)
**9r[c]**	>100 (-)	>100 (-)	100 (-)	>100 (-)	>100 (-)	>100 (-)	25 [50] (13)
**13a**	50 (-)	100 (-)	25 (16)	200 (-)	50 (-)	100 (18)	100 (16)
**13b**	100 (22)	200 (-)	25 (21)	100 (15)	200 (-)	100 (15)	**1.56** [50] (34)
**Fl[d]**	**1** (43)	**2** (32)	**0.38** (39)	**0.38** (40)	**0.75** (40)	64[e] (16)	16[f] (12)

The highest activity against yeasts indicated by the low MIC values (≤12.5 mg/L) is shown in boldface.

(-): The inhibition zone was not observed in the disc-diffusion method. The diameter of paper discs was 9 mm. NT: not tested. The MIC determination could not be performed, because tested substance dissolved in DMSO precipitated after implementation into the RPMI medium.

[a] Only the MFC values ≤400 mg/L are presented.

[b] The MIC and MFC values of the substance were determined up to 50 mg/L. In the table, only the MFC values ≤50 mg/L are presented. The tested substance dissolved in DMSO precipitated after implementation into the RPMI medium at a concentration above 50 mg/L.

[c] The MIC and MFC values of the substance were determined up to 100 mg/L. In the table, only the MFC values ≤100 mg/L are presented. The tested substance dissolved in DMSO precipitated after implementation into the RPMI medium at a concentration above 100 mg/L.

[d]FL, fluconazole was used as a reference antifungal agent; the diameter of commercial disc containing 0.025 mg of fluconazole was 6 mm; the MIC value of fluconazole was determined by the Etest method. [6]

[e] The ellipse was visible pointing the MIC value 64 mg/L, however, with macro-colonies up to concentration ≥256 mg/L. In accordance with the recommendations for Etest method, the MIC value of fluconazole against *C. krusei* can be also interpreted as ≥256 mg/L. [6,7] *C. krusei* is intrinsically resistant to fluconazole.

[f] The ellipse was visible pointing the MIC value 16 mg/L, with colonies up to concentration ≥256 mg/L. There are no recommendations for Etest method interpretation of the MIC value of fluconazole against *S. cerevisiae*. The obtained MIC 16 mg/L is in line with the published results. [8]

4. Cytotoxic activity

Sigmoidal dose response curves for the tested compounds were determined for human lung fibroblasts MRC-5. Plots were generated by GraphPad Prism after fitting the MTT data to sigmoidal dose response equation.

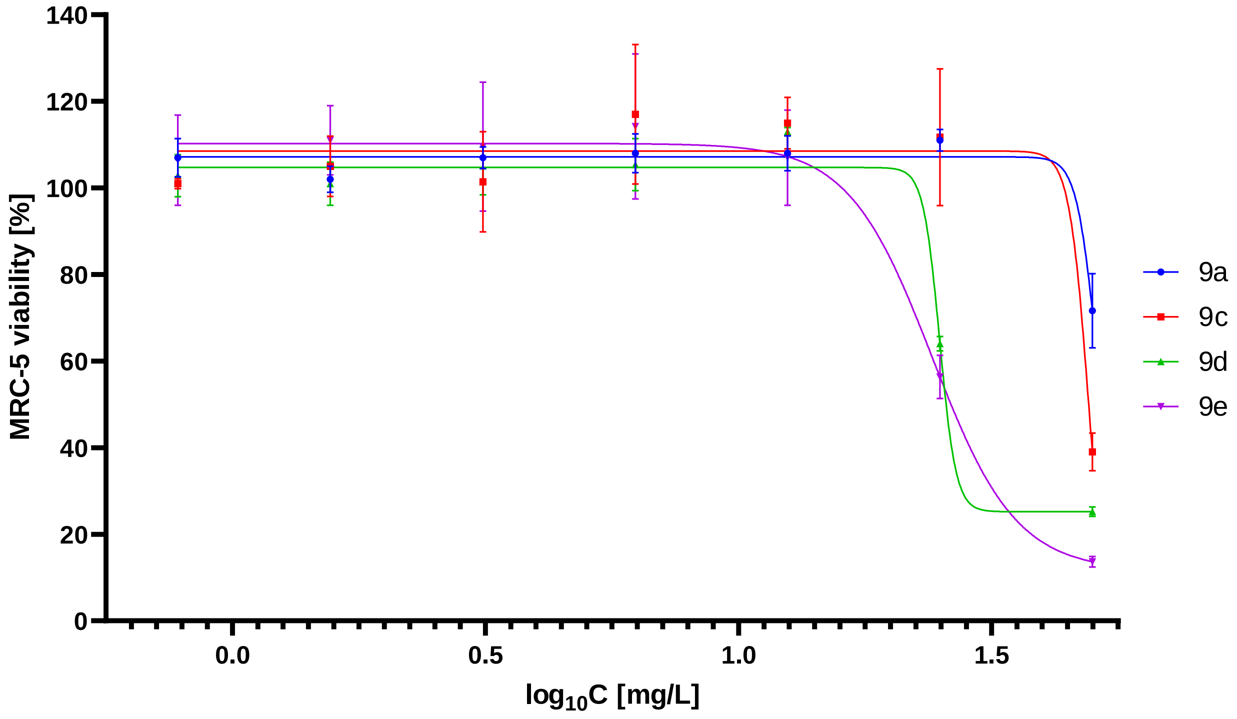

Figure S86. Sigmoidal dose response curves for **9a, 9c-9e** determined for MRC-5.

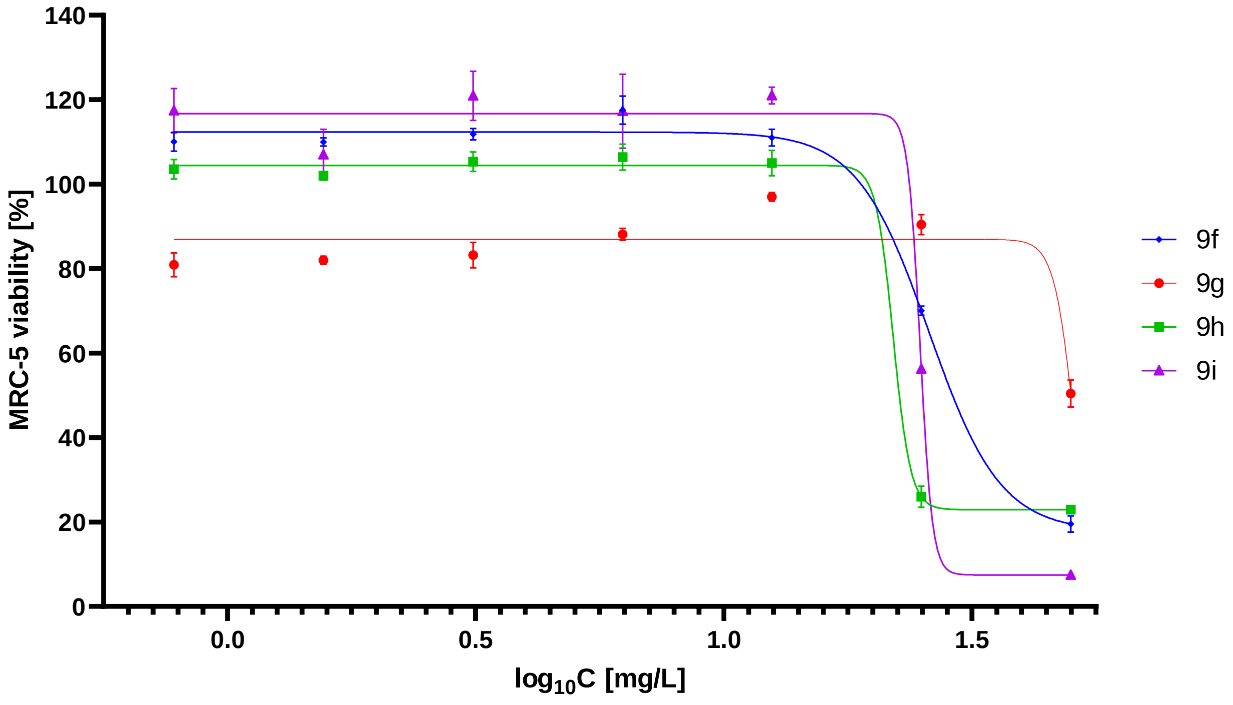

Figure S87. Sigmoidal dose response curves for **9f-9i** determined for MRC-5.

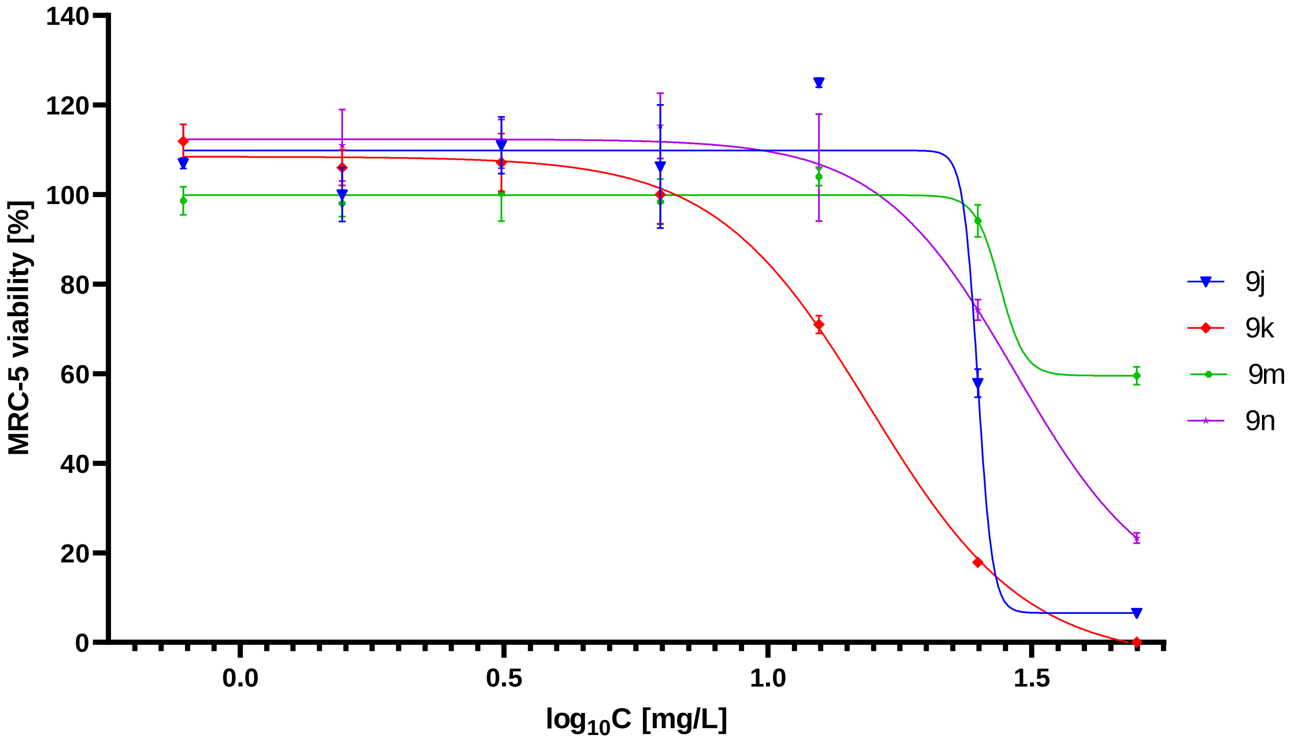

Figure S88. Sigmoidal dose response curves for **9j-9n** determined for MRC-5.

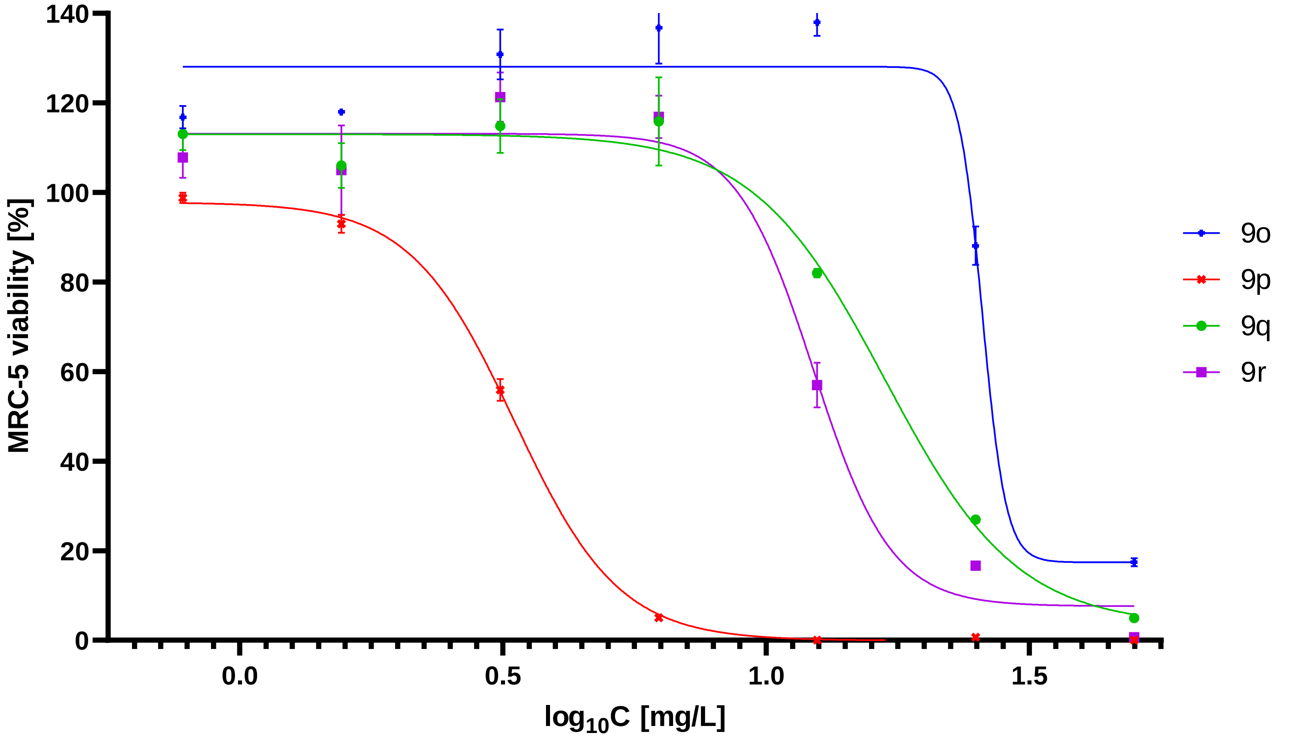

Figure S89. Sigmoidal dose response curves for **9o-9r** determined for MRC-5.

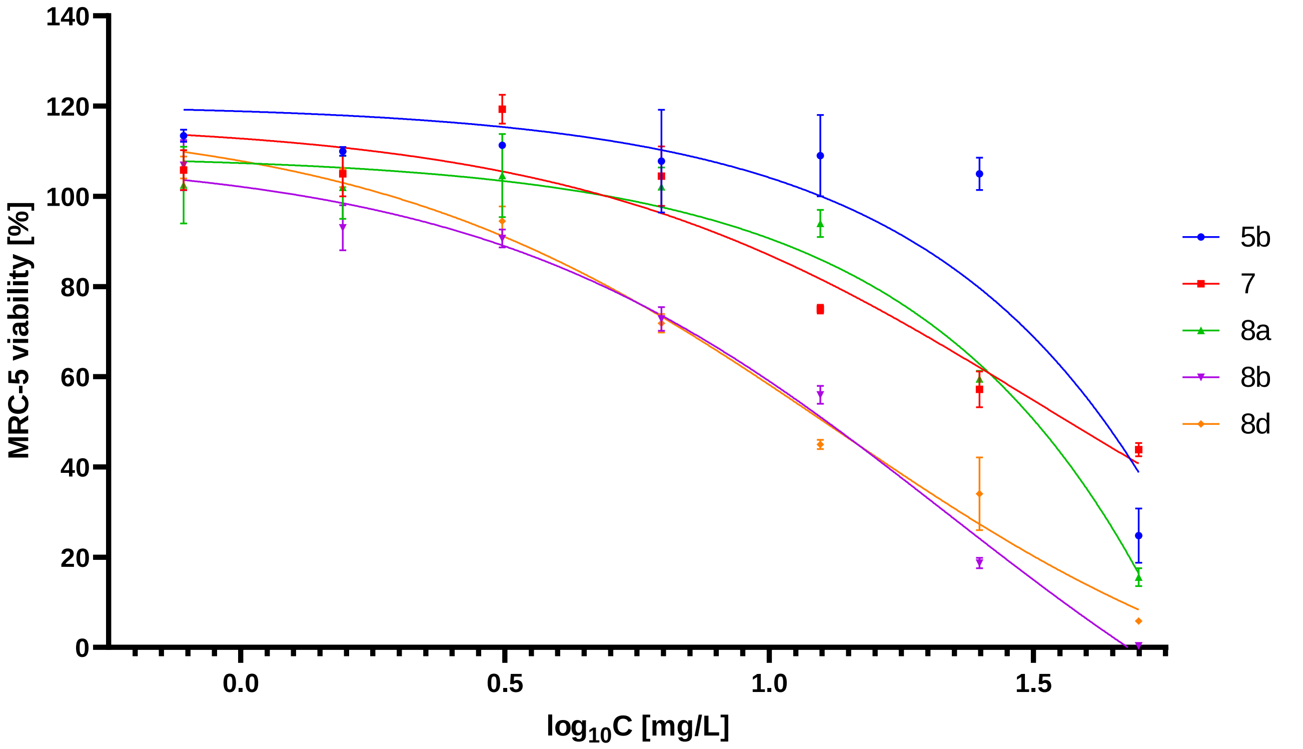

Figure S90. Sigmoidal dose response curves for **5b, 7, 8a, 8b, 8d** determined for MRC-5.

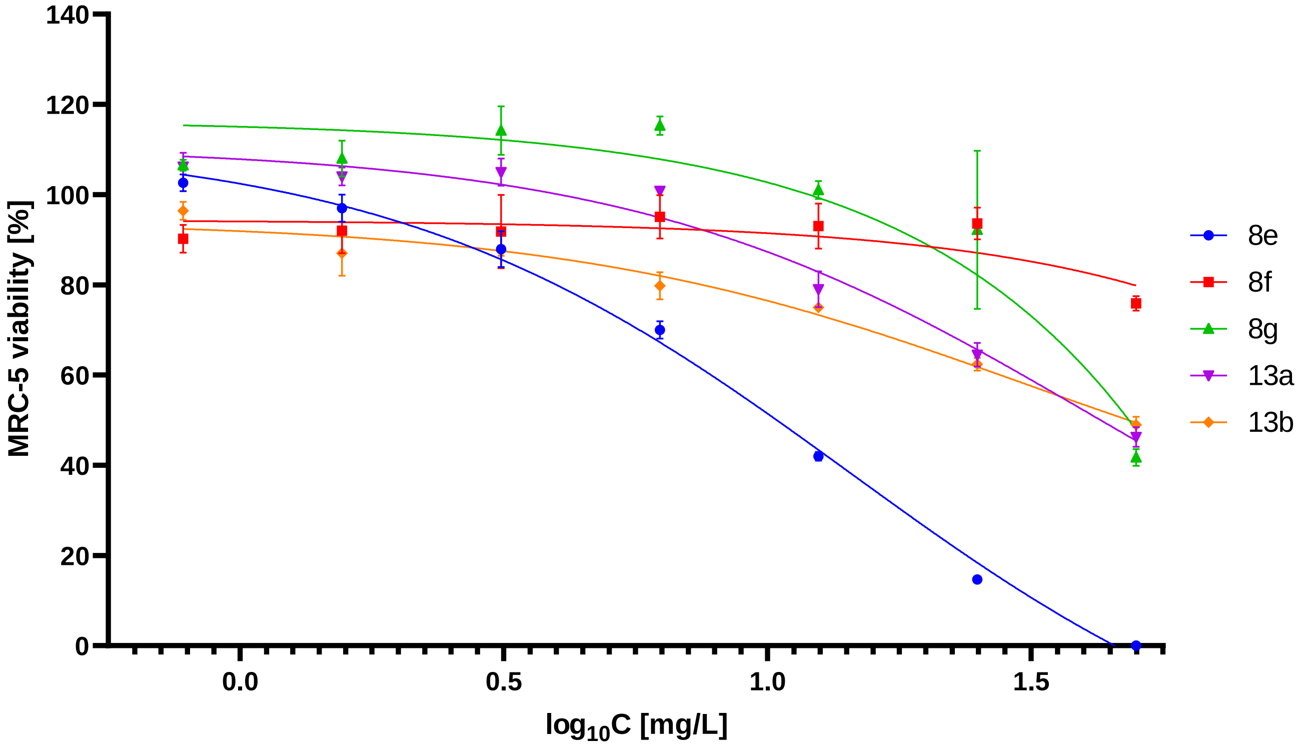

Figure S91. Sigmoidal dose response curves for **8e-8g, 13a, 13b** determined for MRC-5.

5. References

1 *CrysAlis Pro Software*, Oxford Diffraction Ltd, Oxford, U.K., 2010.

2 G. M. Sheldrick, *Acta Crystallogr. A*, 2008, **64**, 112–122.

3 G. M. Sheldrick, *Acta Crystallogr. Sect. C Struct. Chem.*, 2015, **71**, 3–8.

4 Clinical and Laboratory Standards Institute (CLSI), Methods for dilution antimicrobial susceptibility tests for bacteria that grow aerobically, Approved Standard, Document M07-A9, 9th ed., CLSI, 940 West Valley Road, Wayne, Pennsylvania, USA

5 A. Prasetyoputri, A. M. Jarrad, M. A. Cooper and M. A. T. Blaskovich, *Trends Microbiol.*, 2019, **27**, 339–354.

6 ETEST Application guide, BioMerieux. http://www.biomerieux-usa.com/clinical/etest

7 ETEST for antifungal susceptibility testing – research gate, AB BIODISK. https://www.researchgate.net/...fungal.../Etest.pdf,

8 M. A. Pfaller, M. Bale, B. Buschelman, M. Lancaster, A. Espinel-Ingroff, J. H. Rex and M. G. Rinaldi, *J. Clin. Microbiol.*, 1994, **32**, 1650–1653.
